# Projected health and economic impacts of sugar-sweetened beverage taxation in Germany: A cross-validation modelling study

**DOI:** 10.1371/journal.pmed.1004311

**Published:** 2023-11-21

**Authors:** Karl M. F. Emmert-Fees, Ben Amies-Cull, Nina Wawro, Jakob Linseisen, Matthias Staudigel, Annette Peters, Linda J. Cobiac, Martin O’Flaherty, Peter Scarborough, Chris Kypridemos, Michael Laxy

**Affiliations:** 1 Professorship of Public Health and Prevention, School of Medicine and Health, Technical University of Munich, Munich, Germany; 2 Institute for Medical Information Processing, Biometry, and Epidemiology, Pettenkofer School of Public Health LMU Munich, Munich, Germany; 3 Institute of Epidemiology, Helmholtz Zentrum München, Research Center for Environmental Health, Neuherberg, Germany; 4 Nuffield Department of Primary Care Health Sciences, University of Oxford, Oxford, United Kingdom; 5 Oxford Health Biomedical Research Centre, National Institute of Health and Care Research, Oxford, United Kingdom; 6 Epidemiology, University of Augsburg, University Hospital Augsburg, Augsburg, Germany; 7 TUM School of Management, Technical University of Munich, Munich, Germany; 8 School of Medicine and Dentistry, Griffith University, Southport, Australia; 9 Department of Public Health, Policy & Systems, University of Liverpool, Liverpool, United Kingdom; 10 German Center for Diabetes Research (DZD), Neuherberg, Germany; Carolina Population Center, UNITED STATES

## Abstract

**Background:**

Taxes on sugar-sweetened beverages (SSBs) have been implemented globally to reduce the burden of cardiometabolic diseases by disincentivizing consumption through increased prices (e.g., 1 peso/litre tax in Mexico) or incentivizing industry reformulation to reduce SSB sugar content (e.g., tiered structure of the United Kingdom [UK] Soft Drinks Industry Levy [SDIL]). In Germany, where no tax on SSBs is enacted, the health and economic impact of SSB taxation using the experience from internationally implemented tax designs has not been evaluated. The objective of this study was to estimate the health and economic impact of national SSBs taxation scenarios in Germany.

**Methods and findings:**

In this modelling study, we evaluated a 20% ad valorem SSB tax with/without taxation of fruit juice (based on implemented SSB taxes and recommendations) and a tiered tax (based on the UK SDIL) in the German adult population aged 30 to 90 years from 2023 to 2043. We developed a microsimulation model (IMPACT_NCD_ Germany) that captures the demographics, risk factor profile and epidemiology of type 2 diabetes, coronary heart disease (CHD) and stroke in the German population using the best available evidence and national data. For each scenario, we estimated changes in sugar consumption and associated weight change. Resulting cases of cardiometabolic disease prevented/postponed and related quality-adjusted life years (QALYs) and economic impacts from healthcare (medical costs) and societal (medical, patient time, and productivity costs) perspectives were estimated using national cost and health utility data. Additionally, we assessed structural uncertainty regarding direct, body mass index (BMI)-independent cardiometabolic effects of SSBs and cross-validated results with an independently developed cohort model (PRIMEtime). We found that SSB taxation could reduce sugar intake in the German adult population by 1 g/day (95%-uncertainty interval [0.05, 1.65]) for a 20% ad valorem tax on SSBs leading to reduced consumption through increased prices (pass-through of 82%) and 2.34 g/day (95%-UI [2.32, 2.36]) for a tiered tax on SSBs leading to 30% reduction in SSB sugar content via reformulation. Through reductions in obesity, type 2 diabetes, and cardiovascular disease (CVD), 106,000 (95%-UI [57,200, 153,200]) QALYs could be gained with a 20% ad valorem tax and 192,300 (95%-UI [130,100, 254,200]) QALYs with a tiered tax. Respectively, €9.6 billion (95%-UI [4.7, 15.3]) and €16.0 billion (95%-UI [8.1, 25.5]) costs could be saved from a societal perspective over 20 years. Impacts of the 20% ad valorem tax were larger when additionally taxing fruit juice (252,400 QALYs gained, 95%-UI [176,700, 325,800]; €11.8 billion costs saved, 95%-UI [€6.7, €17.9]), but impacts of all scenarios were reduced when excluding direct health effects of SSBs. Cross-validation with PRIMEtime showed similar results. Limitations include remaining uncertainties in the economic and epidemiological evidence and a lack of product-level data.

**Conclusions:**

In this study, we found that SSB taxation in Germany could help to reduce the national burden of noncommunicable diseases and save a substantial amount of societal costs. A tiered tax designed to incentivize reformulation of SSBs towards less sugar might have a larger population-level health and economic impact than an ad valorem tax that incentivizes consumer behaviour change only through increased prices.

## Abstract 2

In a modeling study, Karl MF Emmert-Fees and team estimate the health and economic impact of national sugar-sweetened beverages taxation scenarios in Germany.

## Author summary

**Why was this study done?:**

- Taxation of sugar-sweetened beverages (SSBs), recommended by the World Health Organization (WHO) and implemented in many jurisdictions globally, aims to reduce the noncommunicable disease burden by disincentivizing consumption through increased consumer prices or incentivizing industry reformulation to reduce SSB sugar content.
- No tax on SSBs is currently enacted in Germany and the national government is preparing a new national strategy on food seeking evidence-based recommendations to establish policy priorities until 2050.
- In Germany, the potential long-term health and economic impacts of SSB taxation have not been evaluated.

**What did the researchers do and find?:**

- We developed and validated a microsimulation model based on national data and international evidence to model the impact of SSB taxation on dietary exposure of added sugar from beverages, body mass index (BMI), cardiometabolic diseases, and related economic costs.
- We evaluated 3 SSB taxation scenarios in Germany with the simulation model: (1) 20% ad valorem tax on SSBs; (2) extended 20% ad valorem tax on SSBs and fruit juice; (3) tiered tax leading to reformulation of SSBs towards 30% lower sugar content.
- Taxation of SSBs in Germany could prevent or postpone 132,100 to 244,100 cases of type 2 diabetes, gain 106,000 to 192,300 quality-adjusted life years (QALYs) and save €10.8 to €16.0 billion in societal cost from 2023 to 2043 with the highest impacts estimated for tiered taxation.
- The absolute long-term health impacts are largely dependent on the relevance of direct, BMI-independent cardiometabolic effects of SSBs.

**What do these findings mean?:**

- All modelled SSB taxation scenarios are likely to improve population health and reduce societal costs in Germany by preventing cardiometabolic disease.
- Considering all sources of uncertainty, we find that modelled SSB taxation scenarios that lead to reformulation towards less sugar might have a larger population-level health and economic impact than those that incentivize consumer behaviour change only through increased prices.
- From a public health perspective, taxation of SSBs should be considered as a policy option for German decision-makers to reduce consumption of added sugar and improve population health.

## 1. Introduction

In Central Europe, around 27% of premature deaths in 2017 were associated with dietary risk factors [1]. This is assumed to be a direct consequence of food environments fuelling unhealthy diets characterised by a high intake of energy-dense (often ultra-processed) foods high in fat, salt, and sugar [2]. Sugar-sweetened beverages (SSBs), usually defined as beverages with added caloric sweeteners (mostly high-fructose corn syrup and sucrose), are the main source of added sugars in global diets [3]. Excessive consumption of added sugars has been shown to increase morbidity and mortality, indirectly through excess calorie intake leading to weight gain and directly by increasing the risk for coronary heart disease (CHD), type 2 diabetes mellitus (T2DM), and dental caries [3].

To address the health and socioeconomic burden of unhealthy diets, the taxation of unhealthy foods is an important fiscal policy tool with the aim of disincentivizing consumption by increasing prices [4–6]. In recent years, government regulation of SSBs has been gaining traction and over 45 jurisdictions have implemented fiscal policies of which approximately 70% were enacted since 2015 [7]. Additionally, the World Health Organization (WHO) recommends SSB taxation as a best-buy policy to strengthen noncommunicable disease (NCD) prevention [6].

A recent meta-analysis synthesising ex post evaluations of implemented SSB taxation policies indicated that they indeed increase prices, decrease sales and, if designed with a tiered structure, are particularly effective in promoting reductions in added sugars by the food industry (i.e., via reformulation) [4]. A tiered structure here means that tax levels are differentiated based on beverage sugar content using predefined thresholds (e.g., 0.18 British pound sterling [₤] per litre for drinks containing 5 to 8 grams [g] sugar per 100 millilitre [ml] and ₤0.24 per litre for drinks containing more than 8 g sugar per 100 ml in case of the Soft Drinks Industry Levy [SDIL] in the United Kingdom [UK]). However, although early observational studies indicate that SSB taxes could be effective in preventing obesity, generating robust empirical evidence on their impact on long-term health and economic outcomes is difficult [8,9].

In Germany, over 50% of the adult population are overweight and the prevalence of obesity has been increasing to almost 20% over the last years, following a clear socioeconomic gradient [10]. Assessment of the potentially related dietary risk factors is difficult because no regular surveillance of population dietary patterns exists. However, individual studies have shown that most of the population does not follow diet recommendations and that SSB consumption, particularly among younger age groups, is high [11,12].

To improve unhealthy diets, reduce the national burden of NCDs, and increase sustainability of the national food system, the German government is currently gathering recommendations for a national strategy on food [13]. This strategy will cover policy priorities until 2050 in domains such as procurement standards for meals (e.g., as developed by the German Nutrition Society), diet literacy, and sustainable food production [14]. Moreover, while the reduction of sugar in SSBs is a policy objective of the German government, a recent study found that voluntary industry commitments to reduce sugar in soft drinks were not successful [15]. A tax on SSB might be a suitable policy option which is also discussed by German decision-makers and supported by non-governmental organisations such as the German Alliance on NCDs [15].

Some studies have previously attempted to model the health impact of SSB taxation scenarios in Germany. However, these mainly used cohort modelling methods with lower granularity which are not able to account for complex epidemiological dependencies and population dynamics over time and did further not quantify healthcare cost savings or productivity losses [16–18]. The value of these previous studies in providing concrete policy recommendations might thus be limited. Yet, to promote best-practice, cost-effective policies, decision-makers need contemporary and context-specific evidence. This is highlighted by the political processes surrounding the enactment of SSB fiscal policies in Mexico and the UK for which timely scientific evidence played an important role [19].

In this study, we evaluate the impact of context-relevant SSB taxation scenarios in Germany on stroke, CHD, and T2DM morbidity, mortality and healthcare, patient time, and productivity costs with a new individual-level population health microsimulation model (IMPACT_NCD_ Germany). Additionally, we explore structural uncertainty in the predicted policy impact by assessing the relevance of direct, body mass index (BMI)-independent cardiometabolic effects of SSBs and cross-validate our results with a second independently developed Markov cohort simulation model (PRIMEtime) [20–22].

## 2. Methods

### Modelling overview

We evaluated 3 SSB tax policy scenarios that were chosen based on international scientific consensus recommendation and globally implemented SSB taxes accounting for context-relevant factors:

1. 20% ad valorem tax on SSBs based on scientific recommendations and implemented ad valorem or volumetric taxes in, e.g., Mexico, Chile, and United States (US) legislatures [4,6,7,23];
2. 20% ad valorem tax on SSBs and fruit juice, extending the tax to account for high consumption levels and the caloric content of juices [12];
3. 30% reformulation of SSBs towards lower sugar content based on tiered taxes such as the UK SDIL [4,7].

Further details on all scenarios and related assumptions are described below and in Methods A in S1 Appendix under “Policy module.”

To simulate the health and economic impact of these SSB taxation scenarios, we developed and validated an NCD microsimulation model for Germany based on the UK IMPACT_NCD_ framework (IMPACT_NCD_ Germany; hereafter IMPACT_NCD_). We modelled the German population age 30 to 90 years over 20 years (2023 to 2043) and performed an economic evaluation from healthcare and societal perspectives [24–27]. For this, we created a synthetic population stratified by age and sex that captures the real demographics, exposures, dietary intakes, and disease epidemiology of the actual German population using available national data sources (see below and in Methods A in S1 Appendix under “Epidemiological engine”).

**Fig 1:**
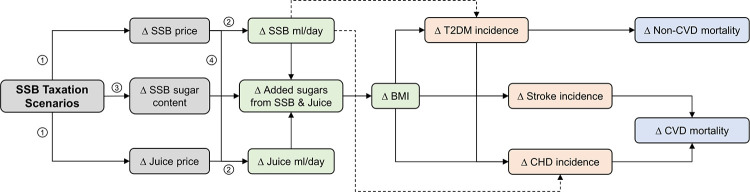
Policy, exposure, and disease pathways of the SSB tax modelling with IMPACT_NCD_ Germany. Complete modelled pathways. Dashed arrows indicate direct effects of SSBs that are excluded in structural uncertainty analyses. Grey boxes indicate modelled policy pathways and corresponding numbered arrows denote underlying modelled mechanisms: (1) Economic theory suggests that SSB producers will pass some proportion of a tax on SSBs along to consumers via increased prices (tax pass-through). (2) After accounting for the tax pass-through rate, economic theory suggests that increased prices of SSBs lead to a change in SSB consumption based on their own-price elasticity of demand. (3) Tiered taxation with different tax rates according to SSB sugar content, incentivizes reformulation towards lower sugar content by producers to avoid the tax burden. (4) After accounting for the tax pass-through rate, economic theory suggests that increased prices of SSBs lead to substitution with similar goods such as fruit juice based on their cross-price elasticity of demand. Green boxes indicate exposures. Orange boxes and blue boxes indicate disease and mortality outcomes, respectively. Δ, “change in”; BMI, body-mass index; Juice, fruit juice; CHD, coronary heart disease; CVD, cardiovascular disease; ml, millilitre; SSB, sugar-sweetened beverages; T2DM, type 2 diabetes mellitus.

The main pathways of our model are founded on widely accepted epidemiological evidence and summarised in Fig 1. Briefly, in our main analysis we assumed that SSB taxation would, depending on the scenario, induce changes in SSB and fruit juice consumption or SSB sugar content guided by economic theory and observations from other countries in which taxes were implemented. We then modelled the effect of changed sugar consumption from SSBs and fruit juice on BMI and consequently BMI and SSB intake as exposures for T2DM, CHD, and stroke. Direct (BMI-independent) effects of SSBs on T2DM and CHD are assumed to be due to the added sugar they contain [21,28]. We also considered T2DM as a risk factor for CHD, stroke, and non-cardiovascular mortality. To analyse structural uncertainty, we (1) re-estimated all scenarios using only BMI-mediated effects, thus excluding the potentially more uncertain estimates of the direct cardiometabolic effects of SSBs from nutritional epidemiological studies [21]; and (2) cross-validated these results with the PRIMEtime cohort model (see below and Methods B and Methods C in S1 Appendix) [29].

Our model choice was guided by a previous review that we conducted during the Policy Evaluation Network project [30]. The technical details of both models have been described previously [20,26,27]. For an overview of all used parameters and data sources see Tables A and G in S1 Appendix. We provide the source code for IMPACT_NCD_ in a public repository at https://github.com/kalleEF/IMPACT-NCD-Germany.

### Sugar-sweetened beverage taxation scenarios

All SSB taxation scenarios including the additional taxation of fruit juice in Germany were modelled compared to a baseline without tax. SSBs were defined as (un-)caffeinated soft drinks or fruit drinks with added sugars (i.e., caloric sweeteners). Fruit juice was defined as 100% fruit juice and nectars or other types of juices that might contain added sugars. Unfortunately, we were not able to distinguish types of juices in detail. However, we consider the resulting bias as negligible because over 80% of consumed fruit juice in Germany does not contain added sugars [31].

The modelled SSB tax policy scenarios were based on globally implemented taxes and international scientific consensus recommendations [6,23]. Three scenarios were selected according to their relevance for the German context and considering limitations of the available beverage consumption data. The modelled scenarios were:

1. 20% ad valorem tax on SSBs (i.e., the tax is calculated as a proportion of the price) based on implemented ad valorem or volumetric taxes as for example in Mexico, Chile, and US legislatures [7]. Due to lack of access to product-level ingredient and price data, we could only approximate volumetric taxes through this scenario. However, most implemented taxes were designed to increase prices by 10% to 20% based on scientific recommendations [6,23]. Evidence has shown that such taxes indeed have increased prices and reduced SSB consumption (hereafter: “ad valorem tax”) [4,6,7,23];
2. 20% ad valorem tax on SSBs and fruit juice that extends SSB taxation to account for the caloric content of fruit juices and the high baseline consumption in Germany (hereafter: “extended ad valorem tax”);
3. tiered tax design with increasing tax rates according to specific sugar thresholds that incentivizes producers to reformulate SSBs towards 30% lower sugar content. This was based on observed reformulation effects under the UK SDIL, which could serve as a blueprint policy for other European countries such as Germany (hereafter: “tiered tax”) [15,32,33].

Further details on the scenarios and their implementation in the model are described below and in Methods A in S1 Appendix under “Policy module.”

### Synthetic German population

To simulate the population-level impact of the policy scenarios, we constructed a synthetic German population. For this we combined (1) data on the exposures BMI and intake of SSBs and fruit juice from 3 waves of the Kooperative Gesundheitsforschung in der Region Augsburg (KORA) study (S4, F4, and FF4; 1999, 2007, and 2014), a population-based cohort in southern Germany, and the nationally representative dietary survey Nationale Verzehrsstudie (NVS) II (2006) [34–36]; (2) national data on the epidemiology of stroke, CHD, and T2DM [37]; and (3) information on death counts, population count estimates, and projections by age and sex from official sources and a de novo mortality forecast using a functional demographic model (Methods A in S1 Appendix under “Mortality calibration”) [38–41]. Detailed step-by-step descriptions of all data sources, applied statistical methods, and validation results for the synthetic population are described in Methods A in S1 Appendix under “Epidemiological engine.”

### Estimating exposure distributions

To estimate exposure distributions conditional on age and sex, we used generalised additive models for location, shape and scale (GAMLSS), which can handle complex relationships between the response variable and its predictors and numerous types of distributions [42]. For the distribution of BMI, we used data from the 3 KORA waves to incorporate time trends. In KORA FF4 usual dietary intakes were calculated using a blended approach consisting of up to three 24-h food lists and a food frequency questionnaire [35]. In NVS II dietary intakes were calculated based on two 24-h dietary recalls [34]. For the estimation of the SSB and fruit juice intake distributions in ml/day, we primarily relied on KORA FF4 and supplemented this with the NVS II due to the underrepresentation of younger age groups in KORA FF4. Our method accounts for non- and high-consumers in the beverage intake distributions via mixture models. We calculated sugar intake from SSBs and fruit juice using information on beverage-specific sugar consumption in g/ml that was available in KORA FF4. We adjusted beverage and sugar intake for misreporting with the residual method (i.e., regressed on energy intake) [43]. Further details are in Methods A in S1 Appendix under “Exposure module.”

### Estimating disease epidemiology

Information on the epidemiology of T2DM was gathered from the most recent data of the German national diabetes surveillance [37]. Due to data limitations for stroke and CHD incidence, we applied SCORE2 risk equations to the KORA data to estimate the yearly incidence of cardiovascular disease by age and sex [44, 45]. Incidence trends were based on the empirical trends between follow-ups S4, F4, and FF4. The proportions of stroke and CHD events in these estimates were based on results from the European Prospective Investigation into Cancer and Nutrition (EPIC) [46]. Finally, all epidemiological data was aligned and smoothed with DISMOD II before application in IMPACT_NCD_ to improve consistency between incidence, prevalence, and mortality data [47]. Further details are in Methods A in S1 Appendix under “Disease module.”

### Effect of SSB taxation scenarios on sugar intake

According to economic theory, taxation can have both demand and supply side effects [48]. In the case of SSBs, taxation leads to increased consumer prices as producers pass some proportion of the tax along (i.e., tax pass-through) [48]. Price changes influence consumption of goods due to own- and cross-price elasticities of demand (i.e., %-change in consumption following price increase of the same or another product category by 1%) [49]. However, SSB taxes (e.g., the UK SDIL) can be designed with tax levels that depend on beverage sugar content and thus incentivize product reformulation, giving producers a way to avoid the tax. These mechanisms have important implications on implementing the analysed policy scenarios in our model. For further details, see Methods A in S1 Appendix under “Policy module.”

In the “ad valorem tax” and “extended ad valorem tax” scenarios, the effect of price increases on SSB and fruit juice consumption was modelled with national price elasticities of demand. We first calculated the change in consumption of SSB and fruit juice and consequently the change in respective sugar intake. In the former we considered substitution from SSBs to fruit juice. In both scenarios, we assumed that the policy immediately affected beverage consumption. Because national elasticities were not available, we estimated de novo uncompensated price elasticities for beverage categories with an Almost Ideal Demand System using data from the German household consumption survey (Einkommens- und Verbrauchsstichprobe 2013 and 2018) (Methods A in S1 Appendix under “Policy module”). We estimated the own-price elasticity of SSBs and fruit juice to be −0.956 (95%-confidence interval [−1.174, −0.738], *p* < 0.001) and −1.106 (95%-CI [−1.397, −0.814], *p* < 0.001), respectively. The cross-price elasticity for SSBs and fruit juice was estimated to be 0.052 (95%-CI [−0.138, 0.242], *p* = 0.593) (Table J in S1 Appendix). Based on a recent meta-analysis, we assumed that the tax pass-through was 82% (95%-CI [66,98]; *p* < 0.001) [4].

To implement the effects of reformulation in the “tiered tax” scenario, we had to make several assumptions. Based on a recent evaluation of the UK SDIL, we assumed that individual intake of sugar from SSBs would be reduced by 30% without changing consumption [15]. We additionally assumed that this reduction would be gradually come into effect over 3 years. The reformulation effect can only be approximated since we did not have access to product-level data in the KORA or NVS studies and were thus unable to directly specify the underlying sugar thresholds. This means we assumed that a tiered tax, replicating the design of the UK SDIL and taking German SSB price levels and sugar content into account, would gradually lead to similar reformulation effects as in the UK.

### Effects of exposures on cardiometabolic risk

For all modelled exposure and disease pathways in Fig 1, we used high-quality evidence from meta-analyses of prospective cohort studies or randomised controlled trials and cohort pooling projects to translate changes in sugar and SSB intake into changes in BMI and the risk for stroke, CHD, and T2DM. For simplicity, we considered the metabolic effects of sugar from SSBs and fruit juice on BMI to be the same [50].

We modelled the ceteris paribus effect of reduced consumption of sugar from beverages (i.e., no compensation behaviour beyond considered beverages) on the long-term reduction in BMI with effect estimates from a meta-analysis of prospective cohorts [51]. The predicted reductions in weight are conservative and generally lower than using traditional energy balance equations [52,53] (for comparisons, see Table G in S1 Appendix).

Like previous diet policy modelling studies, we considered (1) BMI-mediated effects on stroke, CHD, and T2DM risk [27,54,55]; (2) BMI-independent (direct) effects of SSBs on CHD and T2DM risk due to their sugar content [21,50,56]; and (3) T2DM as a risk factor for stroke, CHD, and non-cardiovascular mortality [57]. We included direct effects of SSBs to reflect potential underlying mechanisms related to insulin resistance and inflammation but excluded them in structural uncertainty analyses (see below) [21,50,58,59]. However, we did not model potential direct effects of fruit juice due to higher uncertainty in these estimates [21]. An overview of all used risk parameters can be found in Table G in S1 Appendix.

### IMPACT_NCD_ microsimulation

IMPACT_NCD_ is a dynamic, discrete-time, stochastic, open-cohort microsimulation model that simulates the life course of individuals and their counterfactuals under alternative policy scenarios. It enables the detailed simulation of diet policies and their impact on relevant exposures, subsequent disease epidemiology, and mortality in a competing risk framework accounting for different lag-times between exposures and outcomes. The effect of individual exposure changes on disease risk is achieved with individualised attributable fractions (Methods A in S1 Appendix under “Disease module”). Our model was calibrated to observed (2013 to 2019) and future (2020 to 2043) non-cardiovascular (CVD) mortality rates projected with a functional demographic model [38]. Epidemiological and economic outputs of the model on a population level are highly flexible and aggregated from individual life courses. We mainly report (incident) cases and (prevalent) case-years prevented/postponed (i.e., either completely prevented or delayed by 1 or more years). A detailed technical description of the modelling process is given in Methods A in S1 Appendix.

### Health-related medical, patient time, and productivity costs

We estimated medical, patient time, and productivity costs related to morbidity and mortality over the simulation period based on the life course of synthetic individuals. Economic impacts were calculated from healthcare and societal perspectives following contemporary economic evaluation guidelines using a human capital approach [60,61]. We assessed formal health sector costs by applying medical costs for the treatment of stroke, CHD, and T2DM from the newest available national evidence to each incident or prevalent case year [62,63]. In the societal perspective, we additionally assessed informal health sector (i.e., patient time costs for T2DM self-management and health services use) and productivity costs (i.e., sick leave days and early retirement associated with stroke and T2DM and premature death) [64]. These were valued according to published national estimates, except premature death costs that were calculated using average annual gross wages including fringe benefits [65–68]. Medical and patient time costs were applied until death, while productivity losses accumulated until the German retirement age of 65 years. We inflated health sector costs, informal health sector costs, and productivity losses to 2022 prices using the German medical sector price index and the German labour cost index (see Methods A in S1 Appendix under “Health economics module”) [69,70]. All costs were discounted at 3% per year [71].

### Quality-adjusted life years

Cumulative quality-adjusted life years (QALYs) over the simulation period were estimated taking the health-related quality of life of synthetic individuals, including morbidity and mortality, over their life course into account. For this, we re-estimated recently published national health utility decrements accounting for age, sex, BMI, stroke, CHD, and T2DM to improve consistency with our analysis (see Methods A in S1 Appendix under “Health economics module”) [72]. QALYs were discounted at 3% per year [71].

### Uncertainty and sensitivity analyses

IMPACT_NCD_ incorporates stochastic (first-order) and parameter (second-order) uncertainty, as well as individual heterogeneity with extensive probabilistic (Monte Carlo) uncertainty analyses (PUA) using 500 iterations [73,74]. We also assessed structural uncertainty in the predicted policy impact with regards to BMI-independent effects of SSBs by re-estimating all scenarios using only BMI-mediated effects (Fig 1) [74].

We conducted several sensitivity analyses to contextualise our results by providing further comparison scenarios and varying important policy-related assumptions. We modelled: (1) impacts of observed voluntary reformulation of SSBs by industry (i.e., 2% per 6 years) [15]; (2) the tiered tax scenario with reformulation by 10%; (3) tax rates of 10% and 30% for the “ad valorem tax” scenario; (4) the “ad valorem tax” scenario without substitution effects to fruit juice (i.e., setting the cross-price elasticity to 0); (5) the impact of price changes on SSB consumption with a meta-analytic estimate [75]; (6) a maximum impact scenario that combines reformulation and consumption reduction; and (7) varied discount rates for costs and QALYs (0%, 5%, and 10%). Further details on uncertainty and sensitivity analyses are available in Methods A and B in S1 Appendix.

### Model validation

We performed extensive analyses to validate IMPACT_NCD_ according to current guidelines [29]: (1) To ensure internal and face validity, the computational implementation, model outputs, and structure were discussed during meetings among the author group. We also compared inputs and model outputs of disease-specific prevalence and incidence and assessed the ability of the model to track past observed and projected mortality (Fig S–AD in S1 Appendix). (2) To assess external validity, we compared simulated disease-specific epidemiological data from the baseline scenario to comparable external information not used to inform model inputs (Fig AE–AM in S1 Appendix). (3) To cross-validate the predicted policy impact, we modelled all policy scenarios with an adapted version of PRIMEtime, which is a discrete-time proportional multistate life table Markov cohort model. Due to PRIMEtime’s preexisting structure, cross-validation was performed for the analysis including only BMI-mediated effects. As cross-validation targets, we used disease-specific cases prevented/postponed and QALYs. We minimised potential differences between models, by generating exposure and epidemiological inputs for PRIMEtime based on the synthetic German population; using the same data sources where possible; and aligning preexisting disease risk parameters. Uncertainty in PRIMEtime was separately assessed with 1,000 PUA iterations and only includes parameter (second-order) uncertainty. Technical descriptions of PRIMEtime have been published previously [20,76]. See Methods B and C in S1 Appendix for an overview of PRIMEtime and an extensive description of the cross-validation.

### Patient and public involvement

No patients or members of the public were involved in the design and conduct of this study or the interpretation of its results. The results of this study will be shared as policy briefs with public representatives. We acknowledge that this analysis would not have been possible without data from participants of the respective cohort and survey studies.

## 3. Results

**Fig 2:**
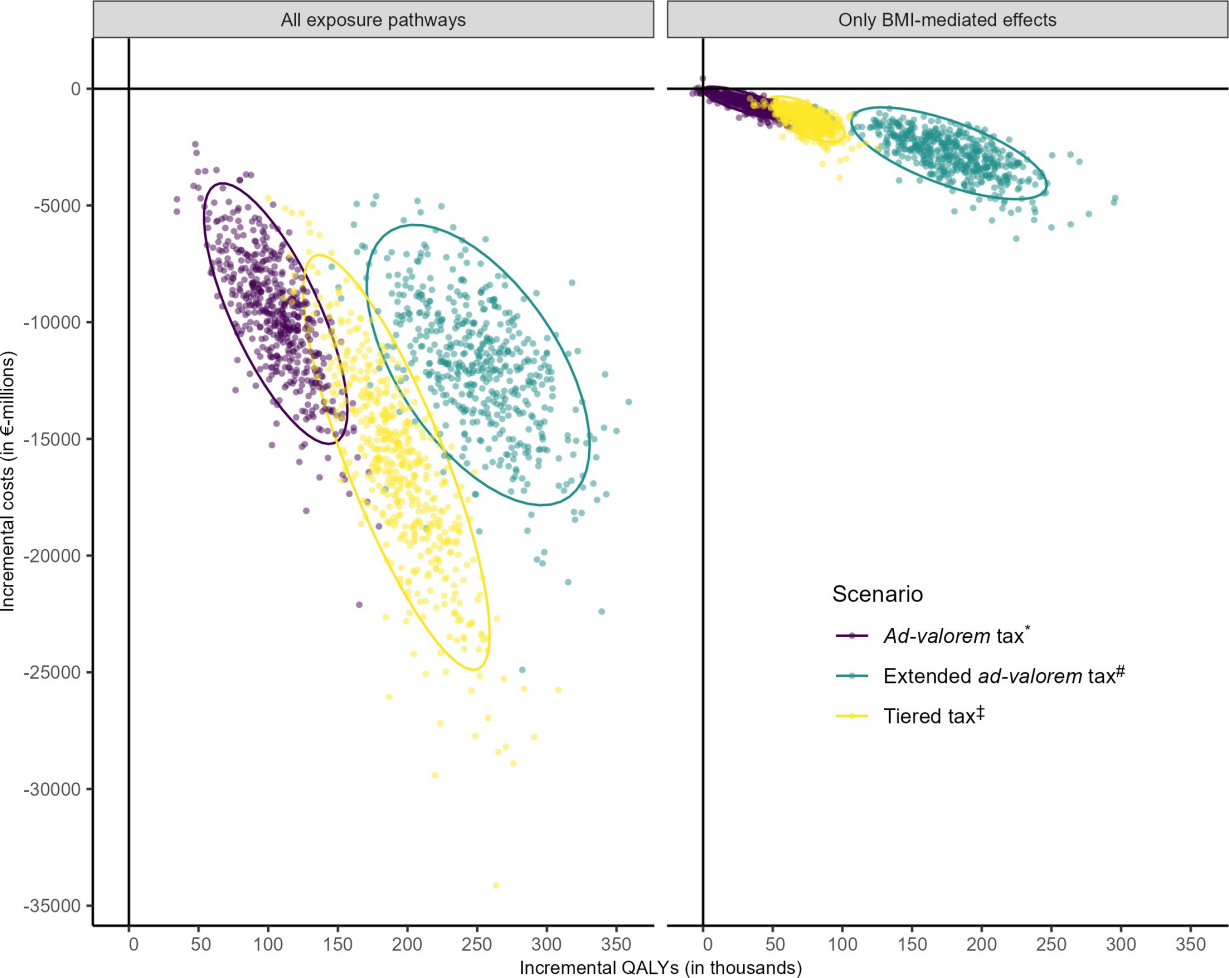
Cost-effectiveness plane for different SSB taxation scenarios in Germany 2023–2043 from a societal perspective. Cost-effectiveness plane showing incremental (i.e., compared to baseline without tax) QALYs on the x-axis and incremental costs from a societal perspective on the y-axis by scenario and included exposure pathways. Ellipses indicate 95%-uncertainty intervals of incremental QALYs and costs per scenario assuming a multivariate t-distribution. *“Ad valorem tax” refers to a 20% ad valorem tax on SSBs with a pass-through to consumers of 82% (for details, see section “Sugar-sweetened beverage taxation scenarios”). ^#^“Extended ad valorem tax” refers to a 20% ad valorem tax on SSBs and fruit juice with a pass-through to consumers of 82% (for details, see section “Sugar-sweetened beverage taxation scenarios”). ^‡^“Tiered tax” refers to a tiered tax on SSBs similar to the UK SDIL that leads to a reduction in SSB sugar content by 30% through reformulation (for details, see section “Sugar-sweetened beverage taxation scenarios”). BMI, body mass index; QALY, quality-adjusted life year; SSB, sugar-sweetened beverages; UK SDIL, United Kingdom Soft Drinks Industry Levy.

We estimated that a national tax on SSBs and/or juice would decrease consumption of sugar from these beverages in Germany by 1 g/day (95%-uncertainty interval [UI] [0.05 to 1.65]) in the “ad valorem tax” scenario; 5.91 g/day (95%-UI [5.37, 6.04]) in the “extended ad valorem tax” scenario and 2.34 g/day (95%-UI [2.32, 2.36]) in the “tiered tax” scenario (Table 1). An overview of all exposure changes by scenario is given in Table S in S1 Appendix. Over 20 years, all modelled scenarios would have a positive impact on population health in Germany, reducing obesity, saving healthcare costs, and leading to productivity gains (Table 2 and Fig 2). This finding was robust, irrespective of the simulation model used and whether direct effects of sugar in SSBs were considered, albeit estimated impacts were a lot lower when excluding them (Fig 2). We mainly focused on the results including all exposure pathways in the following sections. See Tables T–Z in S1 Appendix for stratified and additional analyses.

**Table 1:** Changes in sugar consumption from SSBs and juice under different policy scenarios in Germany by sex and age groups.

Sex and age group	Change in sugar consumption from SSBs and juice compared to baseline without tax (95%-uncertainty intervals)		
	Ad valorem tax*	Extended ad valorem tax^#^	Tiered tax^‡^
	*Absolute change from 2023 to 2043 in g/day*		
Male			
30–49 years	−2.87 (−3.82, −1.36)	−10.19 (−10.44, −9.28)	−6.11 (−6.22, −6.02)
50–69 years	−1.32 (−2.04, −0.24)	−6.81 (−6.99, −6.17)	−2.99 (−3.04, −2.94)
70–90 years	−0.57 (−1.03, 0.12)	−4.09 (−4.21, −3.69)	−1.39 (−1.42, −1.35)
Female			
30–49 years	−0.90 (−1.69, 0.26)	−6.93 (−7.12, −6.28)	−2.24 (−2.27, −2.20)
50–69 years	−0.44 (−1.06, 0.43)	−5.17 (−5.32, −4.67)	−1.25 (−1.27, −1.23)
70–90 years	−0.22 (−0.64, 0.36)	−3.36 (−3.47, −3.03)	−0.70 (−0.71, −0.68)
*Total*	−*1*.*00 (*−*1*.*65*, −*0*.*05)*	−*5*.*91 (*−*6*.*04*, −*5*.*37)*	−*2*.*34 (*−*2*.*36*, −*2*.*32)*
	*Relative change from 2023 to 2043 in %*		
Male			
30–49 years	−4.73 (−6.35, −2.24)	−16.97 (−17.44, −15.48)	−10.12 (−10.34, −9.94)
50–69 years	−3.55 (−5.45, −0.67)	−18.03 (−18.60, −16.33)	−8.02 (−8.17, −7.86)
70–90 years	−2.53 (−4.57, 0.44)	−17.91 (−18.50, −16.14)	−6.17 (−6.30, −6.02)
Female			
30–49 years	−2.23 (−4.28, 0.68)	−17.44 (−18.01, −15.84)	−5.60 (−5.71, −5.50)
50–69 years	−1.58 (−3.78, 1.54)	−18.36 (−18.98, −16.57)	−4.46 (−4.54, -4.38)
70–90 years	−1.17 (−3.46, 2.03)	−18.41 (−18.99, −16.51)	−3.71 (−3.79, -3.64)
*Total*	−*2*.*94 (*−*4*.*80*, −*0*.*18)*	−*17*.*15 (*−*17*.*54*, −*15*.*60)*	−*6*.*85 (*−*6*.*92*, *-6*.*79)*

Absolute and relative changes in sugar consumption (in grams per day) from SSBs and juice under different policy scenarios in Germany by sex and age groups.

*“Ad valorem tax” refers to a 20% ad valorem tax on SSBs with a pass-through to consumers of 82% (for details, see section “Sugar-sweetened beverage taxation scenarios”).

^#^“Extended ad valorem tax” refers to a 20% ad valorem tax on SSBs and fruit juice with a pass-through to consumers of 82% (for details, see section “Sugar-sweetened beverage taxation scenarios”).

^‡^“Tiered tax” refers to a tiered tax on SSBs similar to the UK SDIL that leads to a reduction in SSB sugar content by 30% through reformulation (for details, see section “Sugar-sweetened beverage taxation scenarios”).

g, grams; SSB, sugar-sweetened beverages; UK SDIL, United Kingdom Soft Drinks Industry Levy.

**Table 2:** Health and economic impact of different SSB taxation scenarios in Germany 2023–2043 from healthcare and societal perspectives.

*Health outcomes*	Change in outcomes compared to baseline without tax (95%-uncertainty intervals)		
	Ad valorem tax*	Extended ad valorem tax^#^	Tiered tax^‡^
Cases prevented/postponed^†^			
T2DM	132,100 (61,700, 202,900)	190,800 (112,000, 269,700)	244,100 (118,200, 365,300)
CHD	39,200 (21,100, 58,100)	45,800 (27,500, 66,200)	69,800 (38,800, 101,900)
Stroke	1,900 (0, 4,500)	4,500 (1,900, 8,500)	3,400 (800, 7,100)
Obesity	31,600 (−5,400, 72,600)	159,400 (97,100, 232,400)	72,300 (36,400, 105,500)
Case-years prevented/postponed^†^			
T2DM	1,109,300 (481,700, 1,838,200)	1,569,600 (876,500, 2,313,800)	1,940,900 (879,200, 3,106,500)
CHD	239,700 (112,300, 375,600)	274,700 (146,600, 415,100)	408,200 (206,600, 620,900)
Stroke	4,300 (−6,600, 22,200)	18,600 (2,300, 50,200)	8,900 (−6,600, 32,300)
Obesity	733,800 (99,600, 1,431,500)	3,919,200 (2,340,700, 5,490,500)	1,683,100 (1,035,800, 2,341,000)
All-cause deaths prevented/postponed	17,000 (8,600, 26,100)	21,600 (12,600, 31,800)	29,300 (15,900, 44,900)
QALYs gained	106,000 (57,200, 153,200)	252,400 (176,700, 325,800)	192,300 (130,100, 254,200)
Life years gained	95,400 (47,300, 161,000)	114,200 (61,300, 187,300)	156,700 (77,900, 255,400)
Difference in life expectancy	0.02 (−0.01, 0.05)	0.02 (−0.01, 0.06)	0.03 (0.00, 0.08)
Difference in life expectancy at age 60 years	0.01 (−0.01, 0.02)	0.01 (−0.01, 0.03)	0.01 (−0.01, 0.04)
***Health-related cost outcomes (€-millions)***			
Healthcare costs			
T2DM	−1,613 (−2,750, −684)	−2,310 (−3,492, −1,311)	−2,785 (−4,601, −1,249)
CHD	−660 (−1,003, −345)	−792 (−1,170, −461)	−1,136 (−1,631, −619)
Stroke	−89 (−225, 0)	−204 (−425, −84)	−153 (−364, −35)
Other	118 (59, 202)	144 (77, 236)	190 (97, 326)
Productivity costs			
T2DM early retirement	−24 (−60, 5)	−35 (−73, −3)	−41 (−101, 4)
T2DM sick leave	−1,170 (−2,809, −384)	−1,536 (-3,427, -541)	−2,013 (−4,707, −610)
Stroke early retirement	−1 (−33, 5)	−5 (−70, 0)	−3 (−62, 3)
Stroke sick leave	0 (−1, 0)	0 (−3, 0)	0 (−2, 0)
Premature death	−3,556 (−7,135, −1,260)	−3,904 (−7,388, −1,518)	−5,913 (−10,999, −2,265)
Time costs			
T2DM self-management	−1,146 (−2,020, −475)	−1,461 (−2,420, −730)	−1,941 (−3,368, −863)
T2DM time for health service use	−1,446 (−3,508, −502)	−1,892 (−4,424, −718)	−2,470 (−5,667, −847)
Other time for health service use	374 (153, 651)	485 (239, 781)	633 (269, 1,068)
***Cost-effectiveness***			
Total change in costs from healthcare perspective (€-millions)	−2,262 (−3,596, −1,189)	−3,141 (−4,568, −1,942)	−3,850 (−6,075, −2,070)
Total change in costs from societal perspective (€-millions)	−9,584 (−15,304, −4,714)	−11,827 (−17,887, −6,702)	−16,013 (−25,500, −8,090)
ICER^§^ (healthcare perspective)	Dominant	Dominant	Dominant
ICER^§^ (societal perspective)	Dominant	Dominant	Dominant

*“Ad valorem tax” refers to a 20% ad valorem tax on SSBs with a pass-through to consumers of 82% (for details, see section “Sugar-sweetened beverage taxation scenarios”).

^#^“Extended ad-valorem tax” refers to a 20% ad valorem tax on SSBs and fruit juice with a pass-through to consumers of 82% (for details, see section “Sugar-sweetened beverage taxation scenarios”).

^‡^“Tiered tax” refers to a tiered tax on SSBs similar to the UK SDIL that leads to a reduction in SSB sugar content by 30% through reformulation (for details, see section “Sugar-sweetened beverage taxation scenarios”).

^†^Cases and case-years prevented/postponed are defined as incident and prevalent cases completely prevented or delayed for 1 or more years, respectively.

^§^Expressed as € per QALY. Only defined for positive incremental costs and else “dominant” because no trade-off exists if health is improved while costs are saved.

CHD, coronary heart disease; ICER, incremental cost-effectiveness ratio; QALY, quality-adjusted life year; SSB, sugar-sweetened beverages; T2DM, type 2 diabetes mellitus; UK SDIL, United Kingdom Soft Drinks Industry Levy.

### Health impact of SSB taxation scenarios

A 20% ad valorem tax (scenario: “ad valorem tax”) on SSBs could prevent/postpone around 1,900 cases of stroke (95%-UI [0, 4,500]), 39,200 cases of CHD (95%-UI [21,100, 58,100]), 132,100 cases of T2DM (95%-UI [61,700, 202,900]), and 31,600 (95%-UI [−5,400, 72,600]) cases of obesity, compared to the counterfactual without the policy, and 1,109,300 (95%-UI [481,700, 1,838,200]) case-years lived with T2DM and 733,800 (95%-UI [99,600, 1,431,500]) case-years lived with obesity could be mitigated (Table 2).

Expanding the tax to fruit juice (scenario: “extended ad valorem tax”) would lead to increased overall health gains (cases prevented/postponed: 4,500 for stroke, 95%-UI [1,900, 8,500]; 45,800 for CHD, 95%-UI [27,500, 66,200]; 190,800 for T2DM, 95%-UI [112,000, 269,700]) (Table 2), and the largest impact on obesity, particularly among women due to their relatively high fruit juice consumption (Methods A in S1 Appendix under “Exposure module”). Obesity cases prevented/postponed would increase to 109,700 (95%-UI [70,400, 162,200]) for men and 50,500 (95%-UI [22,100, 80,900]) for women (Table X in S1 Appendix).

In the “tiered tax” scenario, health impacts for CHD and T2DM were substantially higher than in the “(extended) ad valorem tax” scenarios. Here, the cases prevented/postponed increased to 3,400 for stroke (95%-UI [800, 7,100]), 69,800 for CHD (95%-UI [38,800, 101,900]), 244,100 for T2DM (95%-UI [118,200, 365,300]), and 72,300 (95%-UI [36,400, 105,500]) for obesity (Table 2).

For all scenarios, the policy impact was highest among men due to their higher baseline SSB consumption, particularly in younger ages. Most cases of stroke, CHD, and T2DM would be prevented in the age groups below 70 years (Table W in S1 Appendix).

### Economic impact of SSB taxation scenarios

**Fig 3:**
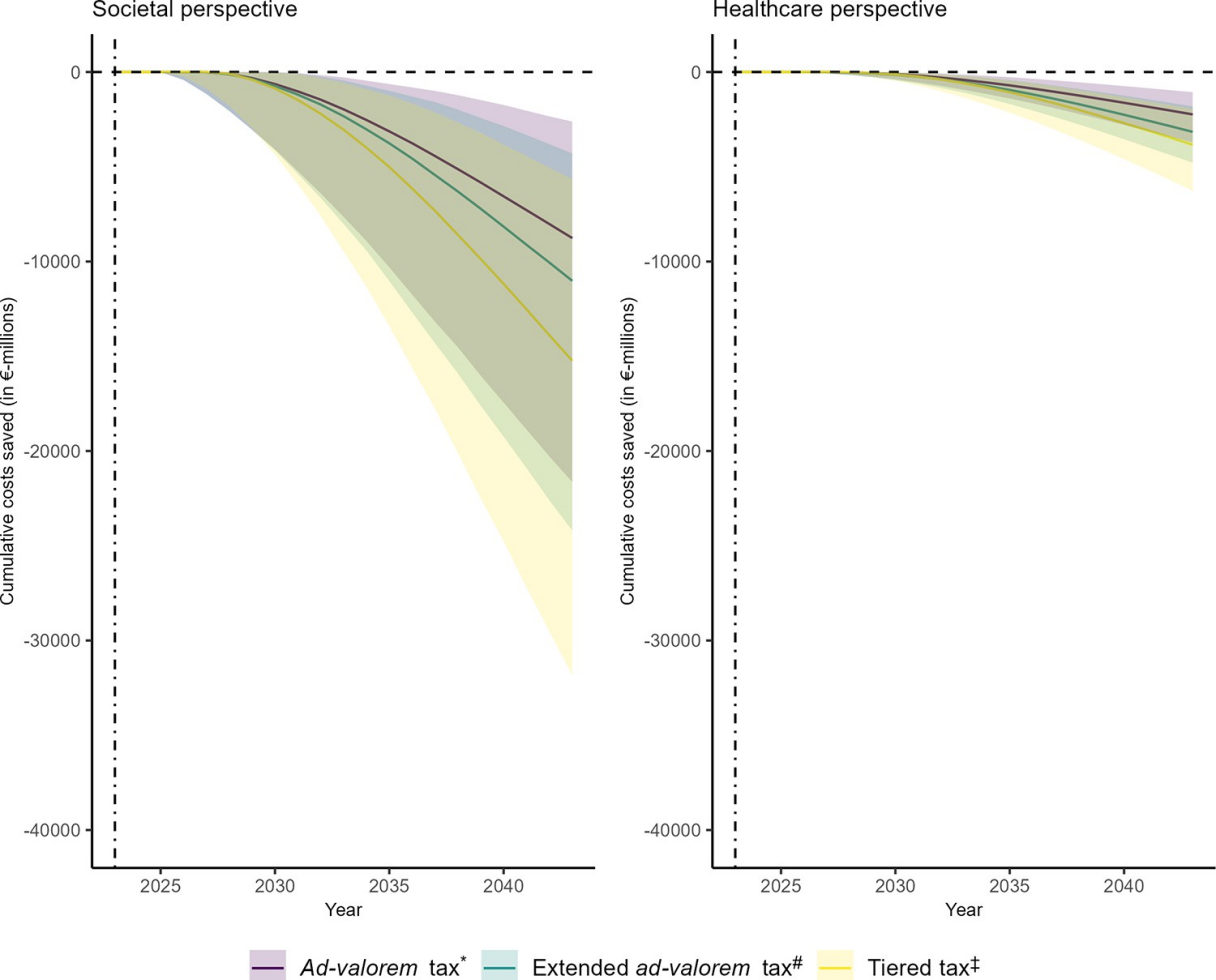
Cumulative costs saved through SSB taxation from 2023 to 2043 by scenario and health economic perspective. Line plots of median cumulative costs saved in €-millions from 2023 to 2043 as a consequence of SSB taxation scenarios stratified health economic perspective. Shaded areas indicate 95%-uncertainty intervals. *“Ad valorem tax” refers to a 20% ad valorem tax on SSBs with a pass-through to consumers of 82% (for details, see section “Sugar-sweetened beverage taxation scenarios”). ^#^“Extended ad valorem tax” refers to a 20% ad valorem tax on SSBs and fruit juice with a pass-through to consumers of 82% (for details, see section “Sugar-sweetened beverage taxation scenarios”). ^‡^“Tiered tax” refers to a tiered tax on SSBs similar to the UK SDIL that leads to a reduction in SSB sugar content by 30% through reformulation (for details, see section “Sugar-sweetened beverage taxation scenarios”). SSB, sugar-sweetened beverages; UK SDIL, United Kingdom Soft Drinks Industry Levy.

Healthcare costs saved, productivity loss averted and QALYs gained over the 20-year simulation period were substantial in all scenarios and mirror the reported health impacts in their relative magnitude between policy scenarios and distribution according to sex and age (Table 2, Tables W and X in S1 Appendix). All 3 policy scenarios were cost-saving and dominant from healthcare and societal perspectives compared to their counterfactual without SSB taxation (Fig 2). Cumulative cost savings over time per scenario and perspective are shown in Fig 3. The total QALYs gained were 106,000 (95%-UI [57,200, 107,100]) for the “ad valorem tax,” 252,400 (95%-UI [176,700, 325,800]) for the “extended ad valorem tax,” and 192,300 (95%-UI [130,100, 254,200]) for the “tiered tax.” We estimated that from a healthcare perspective €2,262 million (95%-UI [€1,189, €3,596]) costs could be saved with the “ad valorem tax”; €3,141 million (95%-UI [1,942, 4,568]) with the “extended ad valorem tax”; and €3,850 million (95%-UI [€2,070, €6,075]) with the “tiered tax.” The largest share of disease-specific costs saved in all scenarios was due to T2DM (Table 2). From a societal perspective, economic gains were many times bigger (€9,584 million, 95%-UI [€4,714, €15,304] savings for the “ad valorem tax”; €11,827 million, 95%-UI [€6,702, €17,887] for the “extended ad valorem tax”; €16,013 million, 95%-UI [€8,090, €25,500] for the “tiered tax”). Productivity gains were largely determined by the prevention of premature deaths and T2DM-related sick leave and time costs (Table 2).

### Validation results

**Fig 4:**
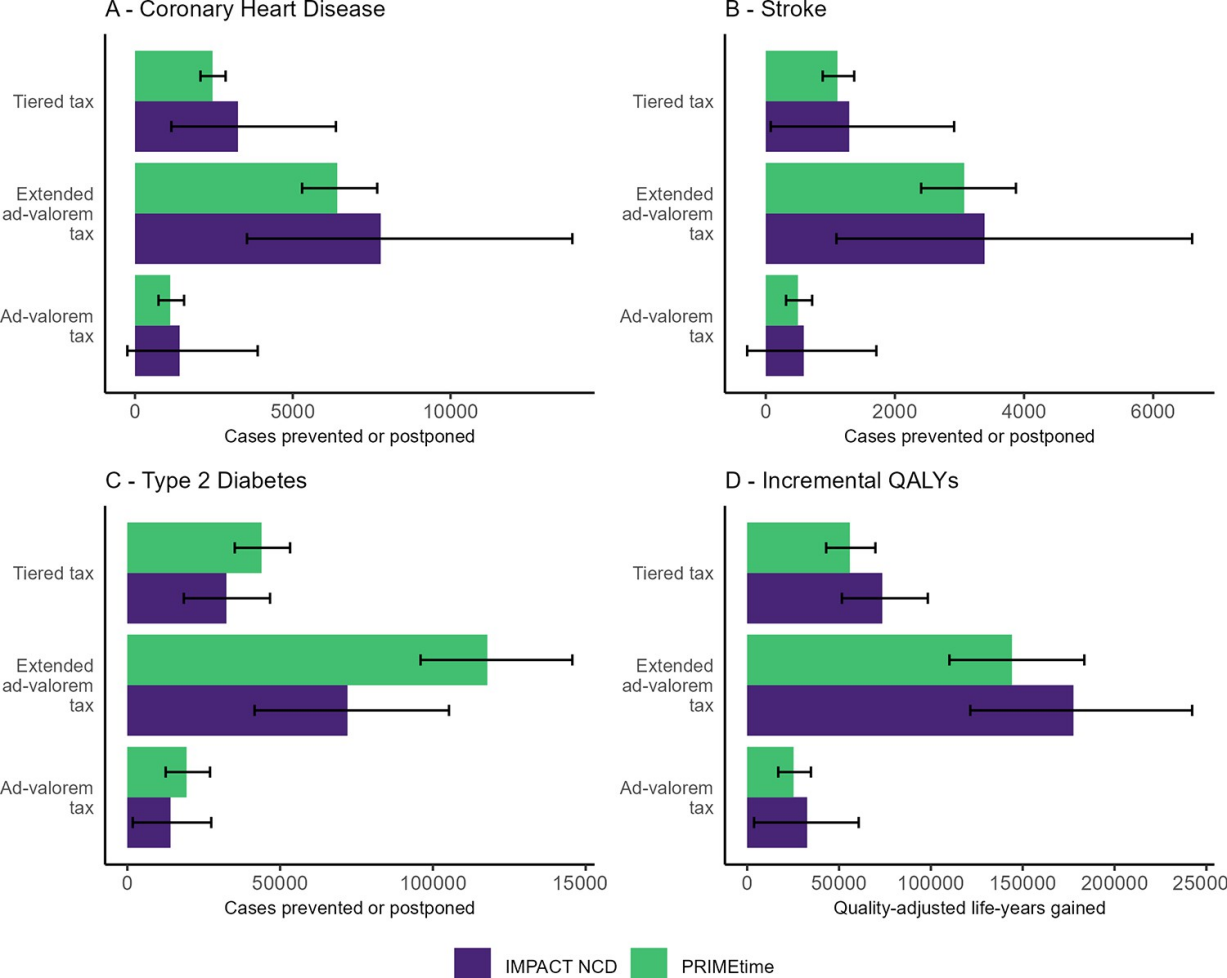
Cross-validation results of IMPACT_NCD_ Germany and PRIMEtime with key health outcomes. Horizontal bar chart comparing cross-validation outcomes for (A) CHD cases prevented/postponed, (B) stroke cases prevented/postponed, (C) type 2 diabetes cases prevented/postponed, and (D) QALYs gained between the IMPACT_NCD_ microsimulation (purple) and the PRIMEtime cohort model (green). Only BMI-mediated exposure pathways are modelled. Error bars indicate 95%-uncertainty intervals. “Ad valorem tax” refers to a 20% ad valorem tax on SSBs with a pass-through to consumers of 82% (for details, see section “Sugar-sweetened beverage taxation scenarios”). “Extended ad valorem tax” refers to a 20% ad valorem tax on SSBs and fruit juice with a pass-through to consumers of 82% (for details, see section “Sugar-sweetened beverage taxation scenarios”). “Tiered tax” refers to a tiered tax on SSBs similar to the UK SDIL that leads to a reduction in SSB sugar content by 30% through reformulation (for details, see section “Sugar-sweetened beverage taxation scenarios”). BMI, body mass index; NCD, noncommunicable disease; QALY, quality-adjusted life year; SSB, sugar-sweetened beverages; UK SDIL, United Kingdom Soft Drinks Industry Levy.

IMPACT_NCD_ performed very well in internal and external validation analyses (Fig S–AM in S1 Appendix). In the cross-validation with PRIMEtime, we found that both models estimated similar health impacts, supporting the overall robustness of our findings. Uncertainty in IMPACT_NCD_ was generally higher than in PRIMEtime but for all scenarios and outcomes, uncertainty intervals overlapped (Fig 4, Fig AN and AO in S1 Appendix).

### Structural uncertainty and sensitivity analyses

When excluding direct, BMI-independent effects of SSBs, the health and economic impact across all scenarios, particularly on T2DM and CHD, was considerably smaller (Fig 2, Table Y in S1 Appendix). Here, the largest health gains would be achieved in the “extended ad valorem tax” scenario (177,600 QALYs gained, 95%-UI [121,500, 242,300]; €2,704 million costs saved from societal perspective, 95%-UI [€1,345, €5,002]). However, considering only SSBs, the “tiered tax” would on average produce larger benefits (73,500 QALYs gained, 95%-UI [51,600, 98,400]; €1,292 million costs saved from societal perspective, 95%-UI [€620, €2,292]) than estimated in the “ad valorem tax” scenario (32,600 QALYs gained, 95%-UI [3,700, 60,700]; €589 million costs saved from societal perspective, 95%-UI [€128, €1,352]). A detailed comparison for all health and economic outcomes is available in Table Y in S1 Appendix.

Health and economic impacts changed sometimes significantly when varying the tax rate for the “ad valorem tax” scenario between 10% (change in QALYs: −43%) and 30% (+48%), excluding substitution effects from SSBs to fruit juice (+6%), or implementing the effect of taxation using a meta-analytic estimate (−25%); decreasing reformulation for the “tiered tax” to 10% (−66%); or varying the discount rate for costs and QALYs (−66% to +30% depending on the discount rate and scenario). We estimated that observed voluntary industry commitments to reduce sugar content in SSBs would only gain 16,400 QALYs and that a combined reformulation and consumption reduction of SSBs would lead with 322,200 QALYs gained to the highest impact (Tables T–V in S1 Appendix).

## 4. Discussion

We use established epidemiological evidence and national data to develop the first population health microsimulation model for Germany (IMPACT_NCD_), which we apply to estimate the health and economic impact of 3 SSB taxation scenarios. Our study suggests that the implementation of a German national ad valorem or tiered tax on SSBs following international scientific recommendations and examples such as Mexico and the UK and considering the additional taxation of fruit juice, could lead to substantial health gains (approximately 106,000 to approximately 250,000 QALYs gained) and save a substantial amount of costs from both healthcare (approximately €2,300 to approximately €3,800 million) and societal perspectives (approximately €9,600 to approximately €16,000 million) over the next 20 years. We find that a reduction of SSB sugar content, as observed following the introduction of the UK SDIL, is likely to lead to higher health gains and averted costs than the consumption reduction that could be expected from a 20% ad valorem tax without any reformulation. We show that the additional taxation of fruit juice would lead to increased health gains in the “ad valorem tax” scenario and could be justified due to high consumption levels in Germany. In sensitivity analyses, we find that health impacts that can be expected from observed voluntary industry commitments to reduce sugar in SSBs are negligible compared to the modelled SSB taxation scenarios. We also estimate that the health and economic impact of SSB taxation is considerably higher, if potential direct (BMI-independent) effects of sugar in SSBs are confirmed but that a substantial policy impact can still be expected if only established BMI-mediated effects on cardiometabolic outcomes are considered. Results from validation analyses encourage trust in the model which cross-validated well with the independently developed PRIMEtime cohort model.

Our study is consistent with international modelling studies that have shown that SSB taxation may lead to substantial long-term health and economic impacts, primarily through a reduction in BMI due to lower sugar consumption and the prevention of T2DM [28,77–79]. However, we explicitly show that the quantification of the SSB taxation impact depends to a large degree on the relevance of direct, BMI-independent health effects of SSBs from nutritional epidemiological studies which few modelling studies have considered [28,30]. By transparently reporting the resulting uncertainty, we provide a corridor for the predicted policy impact and highlight the need for robust causal epidemiological estimates. To the best of our knowledge, our study is the first diet policy evaluation to compare individual-level and cohort modelling approaches and with 2 separate, independently developed simulation models. Similar efforts have so far only been made for diabetes- and cancer-specific, clinically oriented models but are very important to improve the quality and trustworthiness of modelling studies [80,81].

We add to the few previous simulation-based studies which have analysed the potential impact of SSB taxation in Germany by providing a much more comprehensive and detailed analysis of different policy scenarios based on real-world examples [16–18]. While we come to the same overall conclusion that taxation of SSBs is very likely to have positive impacts on population health, we can provide concrete policy guidance that a tax design focusing on SSB sugar content is likely most effective. However, this depends to some degree on the relevance of BMI-independent health effects of SSBs. We also quantify the positive cardiometabolic health effects of SSB taxation in Germany and the substantial societal economic impact from productivity losses and patient time costs for T2DM self-management which is more than twice as high as the averted medical costs.

Our findings are supported by evidence on the increase of SSB prices under volumetric or ad valorem taxation, corresponding reductions in SSB purchases and observed reformulation under tiered taxation [4,32,48,82–84]. Robust evidence from randomised trials and cohort studies shows that increased sugar and SSB consumption leads to weight gain [3,85,86]. Recently, observational evidence on the effect of implemented SSB taxes on anthropometric outcomes is emerging [8].

A particular strength of our approach is the use of an established modelling framework, which enables the detailed, flexible simulation of the health of the German population under different scenarios. To our knowledge, we are the first to model the full health and economic impact of SSB taxation on cardiometabolic health in Germany based on international scientific recommendations and implemented policies. Further, our model validates very well, including on externally observed data, and we adhere to transparency guidelines by making all code available in a public repository. We comprehensively consider implications of all sources of uncertainty from parameter uncertainty to the included risk relationships and the chosen simulation method, which has not been done before in population health modelling. This makes our findings particularly robust. Finally, we provide concrete policy recommendations for the new German national strategy on food and beyond.

However, several limitations need to be considered. First, Germany lacks a regular health examination survey that includes granular, individual-level dietary intake data, and the last official national nutrition survey is from 2007 [12]. We therefore had to rely primarily on the KORA S4 cohort study with its 2 follow-ups F4 and FF4 (1999 to 2014) for anthropometric and nutrition data that was designed to be population representative of the Augsburg region in southern Germany [36]. Thus, full representation of the German population with regards to the modelled exposures might be biased and should be addressed in future iterations of the model if appropriate data sources are available. Second, we were not able to include time trends in beverage consumption which was only collected in FF4. A comparison with annual, industry-reported, aggregated beverage consumption data indicates that (1) SSB and juice intake in Germany has remained stable; (2) SSB consumption is likely underreported; and (3) juice consumption may be overreported (Methods A in S1 Appendix under “Exposure module”). Third, we do not account for health effects beyond CVD and T2DM. Fourth, we do not consider cumulative effects of sugar intake over the life course. Fifth, we do not incorporate heterogeneity in price elasticities by age, sex, or other characteristics. Additionally, our elasticities were estimated with data prior to the recent rise in inflation. However, according to economic theory, price elasticities should generally be higher during inflation as real income diminishes. Sixth, we did not include health effects in children or adolescents. Importantly, the above limitations are unlikely to meaningfully impact our results, but indeed most likely lead to underestimation of the policy impact. Seventh, we cannot perform a detailed simulation of SSB taxes depending on the sugar content of certain products, such as the SDIL, because we do not have access to product-level data. This also does not allow us to perform a standardised comparison of different tax designs or to distinguish tax-related price and reformulation effects in the tiered tax scenario. The interpretation of the relative effectiveness of the simulated tax designs should be made with these limitations in mind. Similarly, in the “ad valorem tax” scenario, we approximate the effects of volumetric taxes which were most often designed to increase prices by 10% to 20% [48]. Eighth, we have neither included costs of implementing an SSB tax, nor industry reformulation costs. Because no good guidance on SSB tax implementation cost exists, previous studies have for example assumed that these would be around 2% of the tax payments [87] or general administrative and auditing costs [88]. However, while implementation costs might be higher for tiered taxes due to their more complex design, considering the estimated population-level economic impacts these are negligible. Finally, as with all population health modelling studies, our results are subject to the validity of the underlying economic and epidemiological evidence including the employed estimates of long-term weight change. We account for the arising epistemic uncertainty by relying on high-quality risk estimates from decades of research and by incorporating as many sources of uncertainty in our simulations as possible.

Our findings have implications for German health and fiscal policy makers and the new national food strategy. We provide evidence that SSB taxation is a cost-effective tool to address the burden of NCDs in Germany. While we acknowledge that voluntary industry commitments to reduce SSB sugar content are in place, a previous analysis has shown that they fail to achieve targeted reductions [15]. We demonstrate that SSB taxation would lead to approximately 10 times larger healthcare cost savings compared to these voluntary commitments. We particularly show that focusing on reductions of SSB sugar content, for example, through a tax design incentivizing reformulation like the UK SDIL, might have larger health and economic impacts than ad valorem taxation of SSBs with moderate tax rates. This finding is consistent with a study from the US [28]. Achieving both a reduction in sugar content of SSBs and their consumption would be most effective. Considering that government efforts to reduce sugar content in SSBs through voluntary commitments were unsuccessful, the introduction of a tiered tax levied on producers, taking the UK SDIL as a blueprint, which gives producers the option to avoid the tax, seems most feasible in the German context.

Recent data from the UK government shows that the tax revenue from the SDIL accumulates to more than ₤300 million per year even after the observed product reformulation [89]. While earmarking of tax revenues to specific purposes is not possible in Germany, these resources could be acknowledged in fiscal negotiations about social and healthcare budgets for NCD preventive measures, ideally striving to alleviate existing health disparities in disadvantaged groups [2]. While we do not calculate the projected tax revenue in our study, it is important to note that different SSB tax designs have implications for this potentially relevant outcome. Since tiered taxes are designed to incentivize reformulation (i.e., to avoid the tax), their revenue might be lower than for ad valorem or volumetric taxes [90].

Based on our findings and from a public health perspective, the additional taxation of fruit juice in Germany could be justified as well, considering high consumption levels [91,92]. In fact, we predict the largest reduction in sugar consumption under the “extended ad valorem tax” (including fruit juice). Yet, this would not translate to the largest health gains if BMI-independent, direct effects of SSBs are considered. However, considering international experiences, the taxation of fruit juice is unlikely [7].

To curb the health, economic, and environmental burden of unhealthy diets over the life course, a comprehensive, population-based multicomponent policy approach transforming the food system is needed [2,93]. This is key to enable citizens to live a healthy life and protect particularly children and adolescents from negative health consequences in adulthood. Further, the German population is progressively ageing and population-based policies which prevent NCDs, such as T2DM, which increase the likelihood for early retirement, can contribute to sustain macroeconomic productivity.

Our study supports the rationale that fiscal policies (i.e., health taxes) which address specific nutritional components or food groups should be considered key preventive policies similarly to those for tobacco control [5,94]. As such they can address negative health externalities resulting from diet-related NCDs [95]. Additionally, negative effects on employment and industry have thus far not been observed [96,97]. Structural health policies such as SSB taxation are also more likely to improve health equity overall compared to alternative, often high-agency, policies like information campaigns [98]. However, one caveat of fiscal policies is that they might be regressive [95].

To further improve the accuracy of population health modelling studies and their relevance for policy makers generally and specifically in Germany many avenues for future research exist. First, the quantification of causal effects of the cross-sectoral, multidimensional, and heterogenous behavioural response to health and social policies is of particular importance [9]. This will both, improve our understanding of policy mechanisms and lead to better projections from policy simulations. Second, while simulation models are most useful for comparing potential future policy scenarios, they can also be useful for monitoring the impact of observed exposure changes within a holistic policy evaluation framework. Here, quasi-experimental methods can be used in conjunction with novel data sources to estimate causal short- and mid-term effects of policies, while simulation models use these estimates to project long-term implications which are infeasible to observe due to identification problems [9,99]. Third, future studies should also aim to find ways of comparing outcomes from real-world evaluations of NCD policies with simulation modelling studies to improve long-term predictions [9]. Fourth, more evidence on established and potential dietary risk factors, such as artificially sweetened beverages, is needed, which ideally provides causal estimates and takes food matrix effects into account [2]. A better understanding of dietary risks, obesity incidence, and health will also lead to better NCD policy modelling studies. Finally, in Germany a better surveillance of dietary and metabolic risk factors across population strata is key to enable the identification of vulnerable population subgroups and assess the equity impact of policies. Addressing these gaps will enable better, timely policy recommendations and ideally improve population health outcomes.

In conclusion, the introduction of a 20% ad valorem or tiered SSB tax in Germany, based on scientific recommendations and taxes implemented in countries like Mexico or the UK, could help to reduce the national burden of cardiometabolic NCDs and save a substantial amount of healthcare and productivity costs. A tiered tax designed to incentivize reformulation of SSBs towards less sugar might have a larger population-level health and economic impact than an ad valorem tax that incentivizes consumer behaviour change only through increased prices.

## Supporting information

S1 Appendix Supplemental methods and results. Detailed methodological description of the applied simulation models, including all preparatory analyses, model parameters and data sources. Additionally, extensive results tables and figures for all sensitivity analyses.
(DOCX)

S1 File Consolidated Health Economic Evaluation Reporting Standards (CHEERS) 2022 statement. (DOCX)

## Abbreviations

BMI: body mass index
CHD: coronary heart disease
CVD: cardiovascular disease
GAMLSS: generalised additive models for location, shape and scale
NCD: noncommunicable disease
PUA: probabilistic uncertainty analysis
QALY: quality-adjusted life year
SDIL: Soft Drinks Industry Levy
SSB: sugar-sweetened beverage
T2DM: type 2 diabetes mellitus
UI: uncertainty interval
UK: United Kingdom
US: United States
WHO: World Health Organization

## References

[1] Afshin A, Sur PJ, Fay KA, Cornaby L, Ferrara G, Salama JS, et al. Health effects of dietary risks in 195 countries, 1990–2017: a systematic analysis for the Global Burden of Disease Study 2017. Lancet. 2019;393(10184):1958–1972. doi: 10.1016/S0140-6736(19)30041-8 30954305 PMC6899507

[2] Mozaffarian D. Dietary and policy priorities to reduce the global crises of obesity and diabetes. Nat Food. 2020;1(1):38–50.

[3] Malik VS, Hu FB. The role of sugar-sweetened beverages in the global epidemics of obesity and chronic diseases. Nat Rev Endocrinol. 2022;18(4):205–218. doi: 10.1038/s41574-021-00627-6 35064240 PMC8778490

[4] Andreyeva T, Marple K, Marinello S, Moore TE, Powell LM. Outcomes Following Taxation of Sugar-Sweetened Beverages: A Systematic Review and Meta-analysis. JAMA Netw Open. 2022;5(6):e2215276. doi: 10.1001/jamanetworkopen.2022.15276 35648398 PMC9161017

[5] Mozaffarian D, Angell SY, Lang T, Rivera JA. Role of government policy in nutrition-barriers to and opportunities for healthier eating. BMJ. 2018;361:k2426. doi: 10.1136/bmj.k2426 29898890 PMC5997034

[6] World Health Organization. Fiscal policies for diet and the prevention of noncommunicable diseases. 2016 [cited 2022 Sep 8]. Available from: https://www.who.int/publications/i/item/9789241511247.

[7] Hawkes C, Jewell J, Allen K. A food policy package for healthy diets and the prevention of obesity and diet-related non-communicable diseases: the NOURISHING framework. Obes Rev. 2013;14(Suppl 2):159–168. doi: 10.1111/obr.12098 24103073

[8] Rogers NT, Cummins S, Forde H, Jones CP, Mytton O, Rutter H, et al. Associations between trajectories of obesity prevalence in English primary school children and the UK soft drinks industry levy: An interrupted time series analysis of surveillance data. PLoS Med. 2023;20(1):e1004160. doi: 10.1371/journal.pmed.1004160 36701272 PMC9879401

[9] Emmert-Fees K, Capacci S, Sassi F, Mazzocchi M, Laxy M. Estimating the impact of nutrition and physical activity policies with quasi-experimental methods and simulation modelling: an integrative review of methods, challenges and synergies. Eur J Public Health. 2022;32:iv84–iv91. doi: 10.1093/eurpub/ckac051 36444112 PMC9706116

[10] Richter A, Schienkiewitz A, Starker A, Krug S, Domanska O, Kuhnert R, et al. Health-promoting behaviour among adults in Germany—Results from GEDA 2019/2020-EHIS. J Health Monit. 2021;6(3):26–44. doi: 10.25646/8553 35146315 PMC8734172

[11] Wawro N, Kleiser C, Himmerich S, Gedrich K, Boeing H, Knueppel S, et al. Estimating Usual Intake in the 2nd Bavarian Food Consumption Survey: Comparison of the Results Derived by the National Cancer Institute Method and a Basic Individual Means Approach. Ann Nutr Metab. 2017;71(3–4):164–174. doi: 10.1159/000481148 28930718

[12] Heuer T, Krems C, Moon K, Brombach C, Hoffmann I. Food consumption of adults in Germany: results of the German National Nutrition Survey II based on diet history interviews. Br J Nutr. 2015;113(10):1603–1614. doi: 10.1017/S0007114515000744 25866161 PMC4462161

[13] Bundesministerium für Ernährung und Landwirtschaft (BMEL). Eckpunktepapier: Weg zur Ernährungsstrategie der Bundesregierung. 2022 [cited 2023 Apr 12]. Available from: https://www.bmel.de/SharedDocs/Downloads/DE/_Ernaehrung/ernaehrungsstrategie-eckpunktepapier.pdf?__blob=publicationFile&v=4.

[14] Jungvogel A, Michel M, Bechthold A, Wendt I. Die lebensmittelbezogenen Ernährungsempfehlungen der DGE. Ernährungs Umschau. 2016;63:M474–M481.

[15] von Philipsborn P, Huizinga O, Leibinger A, Rubin D, Burns J, Emmert-Fees K, et al. Interim Evaluation of Germany’s Sugar Reduction Strategy for Soft Drinks: Commitments versus Actual Trends in Sugar Content and Sugar Sales from Soft Drinks. Ann Nutr Metab. 2023. doi: 10.1159/000529592 36809753 PMC10568594

[16] Schwendicke F, Stolpe M. Taxing sugar-sweetened beverages: impact on overweight and obesity in Germany. BMC Public Health. 2017;17(1):88. doi: 10.1186/s12889-016-3938-4 28095809 PMC5240244

[17] Schwendicke F, Thomson WM, Broadbent JM, Stolpe M. Effects of Taxing Sugar-Sweetened Beverages on Caries and Treatment Costs. J Dent Res. 2016;95(12):1327–1332. doi: 10.1177/0022034516660278 27671690

[18] Tonnies T, Heidemann C, Paprott R, Seidel-Jacobs E, Scheidt-Nave C, Brinks R, et al. Estimating the impact of tax policy interventions on the projected number and prevalence of adults with type 2 diabetes in Germany between 2020 and 2040. BMJ Open Diabetes Res Care. 2021;9(1). doi: 10.1136/bmjdrc-2020-001813 33455907 PMC7813323

[19] UK Research Excellence Framework. Making the case for sugar taxes: UK, Ireland and Mexico. 2021 [cited 2023 Apr 12]. Available from: https://results2021.ref.ac.uk/impact/72ddc4a9-629c-41b4-8cc2-d92f03d2bfd4?page=1.

[20] Briggs ADM, Cobiac LJ, Wolstenholme J, Scarborough P. PRIMEtime CE: a multistate life table model for estimating the cost-effectiveness of interventions affecting diet and physical activity. BMC Health Serv Res. 2019;19(1):485. doi: 10.1186/s12913-019-4237-4 31307442 PMC6633614

[21] Imamura F, O’Connor L, Ye Z, Mursu J, Hayashino Y, Bhupathiraju SN, et al. Consumption of sugar sweetened beverages, artificially sweetened beverages, and fruit juice and incidence of type 2 diabetes: systematic review, meta-analysis, and estimation of population attributable fraction. BMJ. 2015;351:h3576. doi: 10.1136/bmj.h3576 26199070 PMC4510779

[22] Xi B, Huang Y, Reilly KH, Li S, Zheng R, Barrio-Lopez MT, et al. Sugar-sweetened beverages and risk of hypertension and CVD: a dose–response meta-analysis. Br J Nutr. 2015;113(5):709–717. doi: 10.1017/S0007114514004383 25735740

[23] Powell LM, Chaloupka FJ. Food Prices and Obesity: Evidence and Policy Implications for Taxes and Subsidies. Milbank Q. 2009;87(1):229–257. doi: 10.1111/j.1468-0009.2009.00554.x 19298422 PMC2879182

[24] Kypridemos C, Guzman-Castillo M, Hyseni L, Hickey GL, Bandosz P, Buchan I, et al. Estimated reductions in cardiovascular and gastric cancer disease burden through salt policies in England: an IMPACTNCD microsimulation study. BMJ Open. 2017;7(1):e013791. doi: 10.1136/bmjopen-2016-013791 28119387 PMC5278253

[25] Kypridemos C, Collins B, McHale P, Bromley H, Parvulescu P, Capewell S, et al. Future cost-effectiveness and equity of the NHS Health Check cardiovascular disease prevention programme: Microsimulation modelling using data from Liverpool, UK. PLoS Med. 2018;15(5):e1002573. doi: 10.1371/journal.pmed.1002573 29813056 PMC5973555

[26] Laverty AA, Kypridemos C, Seferidi P, Vamos EP, Pearson-Stuttard J, Collins B, et al. Quantifying the impact of the Public Health Responsibility Deal on salt intake, cardiovascular disease and gastric cancer burdens: interrupted time series and microsimulation study. J Epidemiol Community Health. 2019;73(9):881–887. doi: 10.1136/jech-2018-211749 31320459 PMC6820143

[27] Huang Y, Kypridemos C, Liu J, Lee Y, Pearson-Stuttard J, Collins B, et al. Cost-Effectiveness of the US Food and Drug Administration Added Sugar Labeling Policy for Improving Diet and Health. Circulation. 2019;139(23):2613–2624. doi: 10.1161/CIRCULATIONAHA.118.036751 30982338 PMC6546520

[28] Lee Y, Mozaffarian D, Sy S, Liu J, Wilde PE, Marklund M, et al. Health Impact and Cost-Effectiveness of Volume, Tiered, and Absolute Sugar Content SugarSweetened Beverage Tax Policies in the United States: A Microsimulation Study. Circulation. 2020;142(6):523–534. doi: 10.1161/CIRCULATIONAHA.119.042956 32564614 PMC7423682

[29] Eddy DM, Hollingworth W, Caro JJ, Tsevat J, McDonald KM, Wong JB, et al. Model transparency and validation: a report of the ISPOR-SMDM Modeling Good Research Practices Task Force-7. Med Decis Making. 2012;32(5):733–743. doi: 10.1177/0272989X12454579 22990088

[30] Emmert-Fees KMF, Karl FM, von Philipsborn P, Rehfuess EA, Laxy M. Simulation Modeling for the Economic Evaluation of Population-Based Dietary Policies: A Systematic Scoping Review. Adv Nutr. 2021;12(5):1957–1995. doi: 10.1093/advances/nmab028 33873201 PMC8483966

[31] Wirtschaftsvereinigung Alkoholfreie Getränke. Pro-Kopf-Konsum von Erfrischungsgetränken in Deutschland nach Getränkeart in den Jahren 2012 bis 2021. 2022 [cited 2022 Jun 20]. Available from: https://de-statista-com.eaccess.ub.tum.de/statistik/daten/studie/6200/umfrage/pro-kopf-verbrauch-von-erfrischungsgetraenken/.

[32] Pell D, Mytton O, Penney TL, Briggs A, Cummins S, Penn-Jones C, et al. Changes in soft drinks purchased by British households associated with the UK soft drinks industry levy: controlled interrupted time series analysis. BMJ. 2021;372:n254. doi: 10.1136/bmj.n254 33692200 PMC7944367

[33] Scarborough P, Adhikari V, Harrington RA, Elhussein A, Briggs A, Rayner M, et al. Impact of the announcement and implementation of the UK Soft Drinks Industry Levy on sugar content, price, product size and number of available soft drinks in the UK, 2015–19: A controlled interrupted time series analysis. PLoS Med. 2020;17(2):e1003025. 32045418 10.1371/journal.pmed.1003025 PMC7012398

[34] Krems C, Walter C, Heuer T, Hoffmann I. Nationale Verzehrsstudie II—Lebensmittelverzehr und Naehrstoffzufuhr auf Basis von 24h-Recalls. Max Rubner-Institut. 2013.

[35] Mitry P, Wawro N, Six-Merker J, Zoller D, Jourdan C, Meisinger C, et al. Usual Dietary Intake Estimation Based on a Combination of Repeated 24-H Food Lists and a Food Frequency Questionnaire in the KORA FF4 Cross-Sectional Study. Front Nutr. 2019;6:145. doi: 10.3389/fnut.2019.00145 31552261 PMC6743021

[36] Holle R, Happich M, Löwel H, Wichmann HE, MONICA/KORA Study Group. KORA—A Research Platform for Population Based Health Research. Gesundheitswesen. 2005;67(S 01):19–25. doi: 10.1055/s-2005-858235 16032513

[37] Schmidt C, Reitzle L, Dress J, Rommel A, Ziese T, Heidemann C. Prevalence and incidence of documented diabetes based on health claims data-reference analysis for diabetes surveillance in Germany. Bundesgesundheitsblatt Gesundheitsforschung Gesundheitsschutz. 2020;63(1):93–102. 31792553 10.1007/s00103-019-03068-9

[38] Hyndman RJ, Shahid UM. Robust forecasting of mortality and fertility rates: A functional data approach. Comput Stat Data Anal. 2007;51(10):4942–4956.

[39] DeStatis. Statistisches Bundesamt (DeStatis). GENESIS-Online. 2023 [cited 2022 May 19]. Available from: https://www-genesis.destatis.de/genesis/online.

[40] DeStatis. Statistisches Bundesamt (DeStatis). 14. koordinierte Bevölkerungsvorausberechnung für Deutschland. 2022 [cited 2022 May 19]. Available from: https://www.destatis.de/DE/Themen/Gesellschaft-Umwelt/Bevoelkerung/Bevoelkerungsvorausberechnung/_inhalt.html#_oz53odqfm.

[41] DeStatis, Robert Koch-Institut. Statistisches Bundesamt (DeStatis). Informationssystem der Gesundheitsberichterstattung des Bundes. 2023 [cited 2022 May 19]. Available from: https://www.gbe-bund.de/gbe/.

[42] Stasinopoulos MD, Rigby RA, Heller GZ, Voudouris V, De Bastiani F. Flexible regression and smoothing: using GAMLSS in R. CRC Press; 2017.

[43] Willett WC, Howe GR, Kushi LH. Adjustment for total energy intake in epidemiologic studies. Am J Clin Nutr. 1997;65(4):1220S–1228S. doi: 10.1093/ajcn/65.4.1220S 9094926

[44] group SOw, collaboration ESCCr. SCORE2-OP risk prediction algorithms: estimating incident cardiovascular event risk in older persons in four geographical risk regions. Eur Heart J. 2021;42(25):2455–2467. doi: 10.1093/eurheartj/ehab312 34120185 PMC8248997

[45] group Sw, collaboration ESCCr. SCORE2 risk prediction algorithms: new models to estimate 10-year risk of cardiovascular disease in Europe. Eur Heart J. 2021;42(25):2439–2454. doi: 10.1093/eurheartj/ehab309 34120177 PMC8248998

[46] Ricci C, Wood A, Muller D, Gunter MJ, Agudo A, Boeing H, et al. Alcohol intake in relation to non-fatal and fatal coronary heart disease and stroke: EPIC-CVD case-cohort study. BMJ. 2018;361:k934. doi: 10.1136/bmj.k934 29844013 PMC5972779

[47] Barendregt JJ, Van Oortmarssen GJ, Vos T, Murray CJ. A generic model for the assessment of disease epidemiology: the computational basis of DisMod II. Popul Health Metr. 2003;1(1):4. doi: 10.1186/1478-7954-1-4 12773212 PMC156029

[48] Cawley J, Thow AM, Wen K, Frisvold D. The Economics of Taxes on Sugar-Sweetened Beverages: A Review of the Effects on Prices, Sales, Cross-Border Shopping, and Consumption. Annu Rev Nutr. 2019;39:317–338. doi: 10.1146/annurev-nutr-082018-124603 31116649

[49] Nghiem N, Wilson N, Genc M, Blakely T. Understanding price elasticities to inform public health research and intervention studies: key issues. Am J Public Health. 2013;103(11):1954–1961. doi: 10.2105/AJPH.2013.301337 24028228 PMC3828704

[50] Malik VS, Hu FB. Fructose and Cardiometabolic Health: What the Evidence From Sugar-Sweetened Beverages Tells Us. J Am Coll Cardiol. 2015;66(14):1615–1624. doi: 10.1016/j.jacc.2015.08.025 26429086 PMC4592517

[51] Micha R, Shulkin ML, Peñalvo JL, Khatibzadeh S, Singh GM, Rao M, et al. Etiologic effects and optimal intakes of foods and nutrients for risk of cardiovascular diseases and diabetes: Systematic reviews and meta-analyses from the Nutrition and Chronic Diseases Expert Group (NutriCoDE). PLoS ONE. 2017;12(4):e0175149. doi: 10.1371/journal.pone.0175149 28448503 PMC5407851

[52] Huang V, Head A, Hyseni L, O’Flaherty M, Buchan I, Capewell S, et al. Identifying best modelling practices for tobacco control policy simulations: a systematic review and a novel quality assessment framework. Tob Control. 2022. doi: 10.1136/tobaccocontrol-2021-056825 35017262 PMC10447402

[53] Hall KD, Sacks G, Chandramohan D, Chow CC, Wang YC, Gortmaker SL, et al. Quantification of the effect of energy imbalance on bodyweight. Lancet. 2011;378(9793):826–837. doi: 10.1016/S0140-6736(11)60812-X 21872751 PMC3880593

[54] Lu Y, Hajifathalian K, Ezzati M, Woodward M, Rimm EB, Danaei G. Metabolic mediators of the effects of body-mass index, overweight, and obesity on coronary heart disease and stroke: a pooled analysis of 97 prospective cohorts with 1·8 million participants. Lancet. 2014;383(9921):970–983. 24269108 10.1016/S0140-6736(13)61836-X PMC3959199

[55] Singh GM, Danaei G, Farzadfar F, Stevens GA, Woodward M, Wormser D, et al. The age-specific quantitative effects of metabolic risk factors on cardiovascular diseases and diabetes: a pooled analysis. PLoS ONE. 2013;8(7):e65174. doi: 10.1371/journal.pone.0065174 23935815 PMC3728292

[56] Micha R, Peñalvo JL, Cudhea F, Imamura F, Rehm CD, Mozaffarian D. Association Between Dietary Factors and Mortality From Heart Disease, Stroke, and Type 2 Diabetes in the United States. JAMA. 2017;317(9):912–924. doi: 10.1001/jama.2017.0947 28267855 PMC5852674

[57] Stringhini S, Carmeli C, Jokela M, Avendaño M, Muennig P, Guida F, et al. Socioeconomic status and the 25 × 25 risk factors as determinants of premature mortality: a multicohort study and meta-analysis of 1·7 million men and women. Lancet. 2017;389(10075):1229–1237. 28159391 10.1016/S0140-6736(16)32380-7 PMC5368415

[58] Cho YE, Kim DK, Seo W, Gao B, Yoo SH, Song BJ. Fructose Promotes Leaky Gut, Endotoxemia, and Liver Fibrosis Through Ethanol-Inducible Cytochrome P450-2E1–Mediated Oxidative and Nitrative Stress. Hepatology. 2021;73(6):2180–2195. doi: 10.1002/hep.30652 30959577 PMC6783321

[59] Ma L, Hu Y, Alperet DJ, Liu G, Malik V, Manson JE, et al. Beverage consumption and mortality among adults with type 2 diabetes: prospective cohort study. BMJ. 2023;381:e073406. doi: 10.1136/bmj-2022-073406 37076174 PMC10114037

[60] Sanders GD, Neumann PJ, Basu A, Brock DW, Feeny D, Krahn M, et al. Recommendations for Conduct, Methodological Practices, and Reporting of Cost-effectiveness Analyses: Second Panel on Cost-Effectiveness in Health and Medicine. JAMA. 2016;316(10):1093–1103. doi: 10.1001/jama.2016.12195 27623463

[61] Husereau D, Drummond M, Augustovski F, de Bekker-Grob E, Briggs AH, Carswell C, et al. Consolidated Health Economic Evaluation Reporting Standards 2022 (CHEERS 2022) Statement: Updated Reporting Guidance for Health Economic Evaluations. Value Health. 2022;25 (1):3–9. doi: 10.1016/j.jval.2021.11.1351 35031096

[62] Kahm K, Laxy M, Schneider U, Rogowski WH, Lhachimi SK, Holle R. Health Care Costs Associated With Incident Complications in Patients With Type 2 Diabetes in Germany. Diabetes Care. 2018;41(5):971–978. doi: 10.2337/dc17-1763 29348194

[63] Kahm K, Stark R, Laxy M, Schneider U, Leidl R. Assessment of excess medical costs for persons with type 2 diabetes according to age groups: an analysis of German health insurance claims data. Diabet Med. 2020;37(10):1752–1758. doi: 10.1111/dme.14213 31834643

[64] Neumann PJ, Ganiats TG, Russell LB, Sanders GD, Siegel JE. Cost-Effectiveness in Health and Medicine. 2nd ed. New York: Oxford University Press; 2016.

[65] Ulrich S, Holle R, Wacker M, Stark R, Icks A, Thorand B, et al. Cost burden of type 2 diabetes in Germany: results from the population-based KORA studies. BMJ Open. 2016;6(11):e012527. doi: 10.1136/bmjopen-2016-012527 27872118 PMC5129071

[66] Icks A, Claessen H, Strassburger K, Waldeyer R, Chernyak N, Julich F, et al. Patient time costs attributable to healthcare use in diabetes: results from the population-based KORA survey in Germany. Diabet Med. 2013;30(10):1245–1249. doi: 10.1111/dme.12263 23796224

[67] Icks A, Haastert B, Arend W, Konein J, Thorand B, Holle R, et al. Patient time costs due to self-management in diabetes may be as high as direct medical costs: results from the population-based KORA survey FF4 in Germany. Diabet Med. 2020;37(5):895–897. doi: 10.1111/dme.14210 31829456

[68] Winter Y, Wolfram C, Schoffski O, Dodel RC, Back T. [Long-term disease-related costs 4 years after stroke or TIA in Germany]. Nervenarzt. 2008;79(8):918–20, 22–4, 26. 18528673 10.1007/s00115-008-2505-3

[69] DeStatis. Statistisches Bundesamt (DeStatis). Verdienste und Arbeitskosten. 2020 [cited 2023 Feb 6]. Available from: https://www.destatis.de/DE/Themen/Arbeit/Arbeitskosten-Lohnnebenkosten/Publikationen/Downloads-Arbeits-und-Lohnnebenkosten/arbeitskosten-bund-2163201209004.pdf?__blob=publicationFile.

[70] DeStatis. Statistisches Bundesamt (DeStatis). Verbraucherpreisindex nach Zwecken des Individualkonsums. 2023 [cited 2023 Feb 6]. Available from: https://www.destatis.de/DE/Themen/Wirtschaft/Preise/Verbraucherpreisindex/_inhalt.html#249532.

[71] Institut für Qualität und Wirtschaftlichkeit im Gesundheitswesen (IQWiG). Allgemeine Methoden: Version 6.1. 2022 [cited 2023 Apr 21]. Available from: https://www.iqwig.de/methoden/allgemeine-methoden-v6-1.pdf.

[72] Laxy M, Becker J, Kahm K, Holle R, Peters A, Thorand B, et al. Utility Decrements Associated With Diabetes and Related Complications: Estimates From a Population-Based Study in Germany. Value Health. 2021;24(2):274–280. doi: 10.1016/j.jval.2020.09.017 33518034

[73] Briggs AH, Weinstein MC, Fenwick EA, Karnon J, Sculpher MJ, Paltiel AD, et al. Model parameter estimation and uncertainty analysis: a report of the ISPOR-SMDM Modeling Good Research Practices Task Force Working Group-6. Med Decis Making. 2012;32(5):722–732. doi: 10.1177/0272989X12458348 22990087

[74] Garnett GP, Cousens S, Hallett TB, Steketee R, Walker N. Mathematical models in the evaluation of health programmes. Lancet. 2011;378(9790):515–525. doi: 10.1016/S0140-6736(10)61505-X 21481448

[75] Afshin A, Peñalvo JL, Del Gobbo L, Silva J, Michaelson M, O’Flaherty M, et al. The prospective impact of food pricing on improving dietary consumption: A systematic review and meta-analysis. PLoS ONE. 2017;12(3):e0172277. doi: 10.1371/journal.pone.0172277 28249003 PMC5332034

[76] Cobiac LJ, Law C, Scarborough P. PRIMEtime: an epidemiological model for informing diet and obesity policy. 2022.

[77] Long MW, Polacsek M, Bruno P, Giles CM, Ward ZJ, Cradock AL, et al. Cost-Effectiveness Analysis and Stakeholder Evaluation of 2 Obesity Prevention Policies in Maine. US J Nutr Educ Behav. 2019;51(10):1177–1187. 31402290 10.1016/j.jneb.2019.07.005

[78] Briggs ADM, Mytton OT, Kehlbacher A, Tiffin R, Elhussein A, Rayner M, et al. Health impact assessment of the UK soft drinks industry levy: a comparative risk assessment modelling study. Lancet Public Health. 2017;2(1):e15–e22. doi: 10.1016/S2468-2667(16)30037-8 28804786 PMC5543265

[79] Cobiac LJ, Tam K, Veerman L, Blakely T. Taxes and Subsidies for Improving Diet and Population Health in Australia: A Cost-Effectiveness Modelling Study. PLoS Med. 2017;14(2):e1002232. doi: 10.1371/journal.pmed.1002232 28196089 PMC5308803

[80] Criss SD, Cao P, Bastani M, Ten Haaf K, Chen Y, Sheehan DF, et al. Cost-Effectiveness Analysis of Lung Cancer Screening in the United States: A Comparative Modeling Study. Ann Intern Med. 2019. doi: 10.7326/M19-0322 31683314

[81] Kent S, Becker F, Feenstra T, Tran-Duy A, Schlackow I, Tew M, et al. The Challenge of Transparency and Validation in Health Economic Decision Modelling: A View from Mount Hood. Pharmacoeconomics. 2019;37:1305–1312. doi: 10.1007/s40273-019-00825-1 31347104 PMC6860461

[82] Powell LM, Leider J, Oddo VM. Evaluation of Changes in Grams of Sugar Sold After the Implementation of the Seattle Sweetened Beverage Tax. JAMA Netw Open. 2021;4(11):e2132271. doi: 10.1001/jamanetworkopen.2021.32271 34739061 PMC8571660

[83] Powell LM, Leider J. The impact of Seattle’s Sweetened Beverage Tax on beverage prices and volume sold. Econ Hum Biol. 2020;37:100856. doi: 10.1016/j.ehb.2020.100856 32070906

[84] Cawley J, Frisvold D, Jones D. The impact of sugar-sweetened beverage taxes on purchases: Evidence from four city-level taxes in the United States. Health Econ. 2020;29(10):1289–1306. doi: 10.1002/hec.4141 33463850

[85] Malik VS, Pan A, Willett WC, Hu FB. Sugar-sweetened beverages and weight gain in children and adults: a systematic review and meta-analysis. Am J Clin Nutr. 2013;98(4):1084–1102. doi: 10.3945/ajcn.113.058362 23966427 PMC3778861

[86] de Ruyter JC, Olthof MR, Seidell JC, Katan MB. A trial of sugar-free or sugar-sweetened beverages and body weight in children. N Engl J Med. 2012;367(15):1397–1406. doi: 10.1056/NEJMoa1203034 22998340

[87] Wilde P, Huang Y, Sy S, Abrahams-Gessel S, Jardim TV, Paarlberg R, et al. Cost-Effectiveness of a US National Sugar-Sweetened Beverage Tax With a Multistakeholder Approach: Who Pays and Who Benefits. Am J Public Health. 2019;109(2):276–284. doi: 10.2105/AJPH.2018.304803 30571305 PMC6336039

[88] Basto-Abreu A, Barrientos-Gutierrez T, Vidana-Perez D, Colchero MA, Hernandez FM, Hernandez-Avila M, et al. Cost-Effectiveness Of The Sugar-Sweetened Beverage Excise Tax In Mexico. Health Aff (Millwood). 2019;38(11):1824–1831. doi: 10.1377/hlthaff.2018.05469 31682510

[89] HM Revenue & Customs. Soft Drinks Industry Levy statistics commentary 2023. 2023 [cited 2023 Jul 20]. Available from: https://www.gov.uk/government/statistics/soft-drinks-industry-levy-statistics/soft-drinks-industry-levy-statistics-commentary-2021.

[90] Salgado Hernandez JC, Ng SW. Simulating international tax designs on sugar-sweetened beverages in Mexico. PLoS ONE. 2021;16(8):e0253748. doi: 10.1371/journal.pone.0253748 34411108 PMC8375996

[91] Pan A, Hu FB. Effects of carbohydrates on satiety: differences between liquid and solid food. Curr Opin Clin Nutr Metab Care. 2011;14(4). doi: 10.1097/MCO.0b013e328346df36 21519237

[92] Khan TA, Chiavaroli L, Zurbau A, Sievenpiper JL. A lack of consideration of a dose–response relationship can lead to erroneous conclusions regarding 100% fruit juice and the risk of cardiometabolic disease. Eur J Clin Nutr. 2019;73(12):1556–1560. doi: 10.1038/s41430-019-0514-x 31636410 PMC6954109

[93] von Philipsborn P, Geffert K, Klinger C, Hebestreit A, Stratil J, Rehfuess EA, et al. Nutrition policies in Germany: a systematic assessment with the Food Environment Policy Index. Public Health Nutr. 2021;1–10. 10.1017/S1368980021004742 PMC9991688 34881689

[94] Lauer JA, Sassi F, Soucat A, Vigo A, editors. Health Taxes: Policy and Practice. London, United Kingdom: WORLD SCIENTIFIC (EUROPE); 2022.

[95] Allcott H, Lockwood BB, Taubinsky D. Should We Tax Sugar-Sweetened Beverages? An Overview of Theory and Evidence. J Econ Perspect. 2019;33(3):202–227.

[96] Díaz J-J, Sánchez A, Diez-Canseco F, Jaime Miranda J, Popkin BM. Employment and wage effects of sugar-sweetened beverage taxes and front-of-package warning label regulations on the food and beverage industry: Evidence from Peru. Food Policy. 2023:115.

[97] World Bank Group. Hattersley L, Fuchs A, Gonima A, Silver L, Mandeville K. Knowledge Brief: Business, employment, and productivity impacts of SSB taxes. 2020 [cited 2023 Apr 12]. Available from: https://openknowledge.worldbank.org/server/api/core/bitstreams/2802a1fe-2b71-5e42-bf53-234cc7290dd7/content.

[98] Adams J, Mytton O, White M, Monsivais P. Why Are Some Population Interventions for Diet and Obesity More Equitable and Effective Than Others? The Role of Individual Agency. PLoS Med. 2016;13(4):e1001990. doi: 10.1371/journal.pmed.1001990 27046234 PMC4821622

[99] White JS, Basu S, Kaplan S, Madsen KA, Villas-Boas SB, Schillinger D. Evaluation of the sugar-sweetened beverage tax in Oakland, United States, 2015–2019: A quasi-experimental and cost-effectiveness study. PLoS Med. 2023;20(4):e1004212. doi: 10.1371/journal.pmed.1004212 37071600 PMC10112812

