# Supplementary material for: Projected health and economic impacts of sugar-sweetened beverage taxation in Germany: A cross-validation modelling study
Source: PLoS Med. 2023 Nov 21;20(11):e1004311. doi: 10.1371/journal.pmed.1004311 (PMC10662751; doi:10.1371/journal.pmed.1004311)
Supplement: S1 Appendix — Detailed methodological description of the applied simulation models, including all preparatory analyses, model parameters and data sources. Additionally, extensive results tables and figures for all sensitivity analyses. (DOCX) [file pmed.1004311.s001.docx]

**S1 Appendix:** **Supplemental methods and results**

**Projected health and economic impacts of sugar-sweetened beverage taxation in Germany: a cross-validation modelling study**

Karl M. F. Emmert-Fees, Ben Amies-Cull, Nina Wawro, Jakob Linseisen, Matthias Staudigel, Annette Peters, Linda J. Cobiac, Martin O’Flaherty, Peter Scarborough, Chris Kypridemos*, Michael Laxy*

* These authors contributed equally.

**Supplemental Methods**

[Methods A: Detailed technical description of IMPACT_NCD_ Germany 6](#_Toc148882230)

[1. Introduction 6](#_Toc148882231)

[2. Epidemiological engine 13](#_Toc148882232)

[High-level description 13](#_Toc148882233)

[Sociodemographic module 15](#_Toc148882234)

[Exposure module 15](#_Toc148882235)

[Data sources 15](#_Toc148882236)

[KORA 15](#_Toc148882237)

[NVS II 16](#_Toc148882238)

[Exposure estimation procedure 16](#_Toc148882239)

[Estimation of the BMI distribution 17](#_Toc148882240)

[Estimation of the SSB intake distribution 17](#_Toc148882241)

[Estimation of the proportion of diet soft drinks 18](#_Toc148882242)

[Estimation of the fruit juice intake distribution 28](#_Toc148882243)

[Estimation of sugar intake from SSBs and fruit juice 29](#_Toc148882244)

[Simulation of exposures for synthetic individuals 32](#_Toc148882245)

[Disease module 34](#_Toc148882246)

[Disease incidence 34](#_Toc148882247)

[Example procedure based on coronary heart disease (CHD) 37](#_Toc148882248)

[Estimating the observed incidence probability *I_Observed_* for type 2 diabetes 38](#_Toc148882249)

[Estimating the observed incidence probability *I_Observed_* for CHD and stroke 38](#_Toc148882250)

[Initial prevalence 40](#_Toc148882251)

[Disease duration 41](#_Toc148882252)

[Mortality 41](#_Toc148882253)

[Mortality calibration 41](#_Toc148882254)

[Policy module 42](#_Toc148882255)

[Implementation of SSB taxation scenarios 42](#_Toc148882256)

[Price elasticities of demand for beverages in Germany 43](#_Toc148882257)

[Data and methods 44](#_Toc148882258)

[Demand model 44](#_Toc148882259)

[Statistical procedures 44](#_Toc148882260)

[Sensitivity analyses 46](#_Toc148882261)

[Health economics module 47](#_Toc148882262)

[Health-related quality of life 50](#_Toc148882263)

[Systematic literature search for cost parameters in Germany 51](#_Toc148882264)

[Health sector costs 56](#_Toc148882265)

[Medical costs related to type 2 diabetes 56](#_Toc148882266)

[Medical costs related to CHD 56](#_Toc148882267)

[Medical costs related to stroke 56](#_Toc148882268)

[Unrelated (other) medical costs 56](#_Toc148882269)

[Costs outside the formal health sector 57](#_Toc148882270)

[Productivity costs related to type 2 diabetes 57](#_Toc148882271)

[Productivity costs related to stroke 57](#_Toc148882272)

[Productivity costs related to premature death 57](#_Toc148882273)

[Time costs 58](#_Toc148882274)

[3. Model outcomes 62](#_Toc148882275)

[4. Uncertainty and probabilistic sensitivity analysis 62](#_Toc148882276)

[5. Validation and calibration 63](#_Toc148882277)

[Methods B: Overview of the PRIMEtime model 85](#_Toc148882278)

[Methods C: Simulation model cross-validation 86](#_Toc148882279)

[1. Overview of cross-validation approach 86](#_Toc148882280)

[2. Estimation of input data for PRIMEtime 86](#_Toc148882281)

[Demographics and disease epidemiology 86](#_Toc148882282)

[Exposure distributions from synthetic population 87](#_Toc148882283)

[Health economics 87](#_Toc148882284)

[Implementation of policy scenarios 87](#_Toc148882285)

[3. Modifications of the PRIMEtime model structure 87](#_Toc148882286)

[Supplemental References 118](#_Toc148882287)

**Supplemental** **Figures**

[Figure A: Conceptual structure of IMPACT_NCD_ 6](#_Toc148882288)

[Figure B: Validation of BMI distribution by 10-year age group and year 19](#_Toc148882289)

[Figure C: Validation of BMI distribution by sex and year 20](#_Toc148882290)

[Figure D: Comparison of modelled and observed SSB intake from different data sources by sex and age 21](#_Toc148882291)

[Figure E: Validation of SSB intake distribution by 10-year age groups 22](#_Toc148882292)

[Figure F: Validation of SSB intake distribution by sex 23](#_Toc148882293)

[Figure G: Validation of the proportion of diet soft drinks by sex and age 24](#_Toc148882294)

[Figure H: Comparison of modelled and observed fruit juice intake from different data sources by sex and age 25](#_Toc148882295)

[Figure I: Validation of fruit juice intake distribution by 10-year age group 26](#_Toc148882296)

[Figure J: Validation of fruit juice intake distribution by sex 27](#_Toc148882297)

[Figure K: Validation of the amount of sugar per ml of SSB by age group 30](#_Toc148882298)

[Figure L: Validation of the amount of sugar per ml of SSB by sex 30](#_Toc148882299)

[Figure M: Validation of the amount of sugar per ml of fruit juice by age group 31](#_Toc148882300)

[Figure N: Validation of the amount of sugar per ml of fruit juice by sex 31](#_Toc148882301)

[Figure O: Plot of the percentile against the BMI (cumulative distribution) of female synthetic individuals for ages 30 and 50 years 33](#_Toc148882302)

[Figure P: Implemented structure of IMPACT_NCD_ Germany 37](#_Toc148882303)

[Figure Q: Median 1-year cardiovascular disease incidence probability using SCORE2 equations 40](#_Toc148882304)

[Figure R: PRISMA flow chart of the systematic cost searches 55](#_Toc148882305)

[Figure S: Observed, forecast and calibrated coronary heart disease mortality rates from 2013 to 2043 in men 64](#_Toc148882306)

[Figure T: Observed, forecast and calibrated coronary heart disease mortality rates from 2013 to 2043 in women 65](#_Toc148882307)

[Figure U: Observed, forecast and calibrated stroke mortality rates from 2013 to 2043 in men 66](#_Toc148882308)

[Figure V: Observed, forecast and calibrated stroke mortality rates from 2013 to 2043 in women 67](#_Toc148882309)

[Figure W: Observed, forecast and calibrated non-modelled mortality rates from 2013 to 2043 in men 68](#_Toc148882310)

[Figure X: Observed, forecast and calibrated non-modelled mortality rates from 2013 to 2043 in women 69](#_Toc148882311)

[Figure Y: Validation of input versus simulated output coronary heart disease incidence in 2013 70](#_Toc148882312)

[Figure Z: Validation of input versus simulated output stroke incidence in 2013 71](#_Toc148882313)

[Figure AA: Validation of input versus simulated output type 2 diabetes incidence in 2013 72](#_Toc148882314)

[Figure AB: Validation of input versus simulated output coronary heart disease prevalence in 2013 73](#_Toc148882315)

[Figure AC: Validation of input versus simulated output stroke prevalence in 2013 74](#_Toc148882316)

[Figure AD: Validation of input versus simulated output type 2 diabetes prevalence in 2013 75](#_Toc148882317)

[Figure AE: External validation of the simulated coronary heart disease prevalence in 2014 with the GEDA survey by sex and age group 76](#_Toc148882318)

[Figure AF: External validation of the simulated coronary heart disease prevalence in 2019 with the GEDA survey by sex and age group 77](#_Toc148882319)

[Figure AG: External validation of the simulated coronary heart disease prevalence from 2013 to 2018 with ambulatory diagnosis data by sex and age group 78](#_Toc148882320)

[Figure AH: External validation of the simulated stroke prevalence in 2014 with the GEDA survey by sex and age group 79](#_Toc148882321)

[Figure AI: External validation of the simulated stroke prevalence in 2019 with the GEDA survey by sex and age group 80](#_Toc148882322)

[Figure AJ: External validation of the simulated stroke prevalence in 2013 with AOK data from 2011 by sex and age group* 81](#_Toc148882323)

[Figure AK: External validation of the simulated stroke incidence in 2013 with AOK data from 2011 by sex and age group 82](#_Toc148882324)

[Figure AL: External validation of the simulated type 2 diabetes prevalence in 2014 with the GEDA survey by sex and age group 83](#_Toc148882325)

[Figure AM: External validation of the simulated type 2 diabetes prevalence in 2019 with the GEDA survey by sex and age group 84](#_Toc148882326)

[Figure AN: Cross-validation with different sets of relative risks 89](#_Toc148882327)

[Figure AO: Cross-validation with different sets of relative risks stratified by sex 91](#_Toc148882328)

[Figure AP: Cumulative cases prevented or postponed over time by scenario and sex 93](#_Toc148882329)

[Figure AQ: Cumulative case-years prevented or postponed over time by scenario and sex 95](#_Toc148882330)

**Supplemental Tables**

[Table A: Overview of complete data sources used in IMAPCT_NCD_ Germany 8](#_Toc148882331)

[Table B: Key assumptions and limitations of IMPACT_NCD_ Germany 14](#_Toc148882332)

[Table C: Overview of individual exposures and their use in the simulation model 17](#_Toc148882333)

[Table D: Comparison of aggregate SSB consumption data from different data sources 18](#_Toc148882334)

[Table E: Comparison of aggregate fruit juice consumption data from different data sources 28](#_Toc148882335)

[Table F: Overview of disease modelling 34](#_Toc148882336)

[Table G: Relative risks and etiologic effects of exposures on cardiometabolic risk 35](#_Toc148882337)

[Table H: Causal relationships included in the model and the time lag assumed between exposure and outcome 37](#_Toc148882338)

[Table I: Implemented SSB taxation policy scenarios and calculation details 43](#_Toc148882339)

[Table J: Uncompensated price elasticities of demand for beverages in Germany 46](#_Toc148882340)

[Table K: Implemented sensitivity analyses and calculation details 46](#_Toc148882341)

[Table L: Sources and assumptions of cost parameters for the economic evaluation 47](#_Toc148882342)

[Table M: Impact inventory 49](#_Toc148882343)

[Table N: Comparison of original and estimated health utility decrements 50](#_Toc148882344)

[Table O: Eligibility criteria for systematic cost searches 52](#_Toc148882345)

[Table P: MEDLINE search terms to identify studies assessing the cost of diabetes in Germany 53](#_Toc148882346)

[Table Q: MEDLINE search terms to identify studies assessing the cost of coronary heart disease in Germany 53](#_Toc148882347)

[Table R: MEDLINE search terms to identify studies assessing the cost of stroke in Germany 54](#_Toc148882348)

[Table S: Health sector cost parameter values used in the economic evaluation 59](#_Toc148882349)

[Table T: Non-health sector cost parameters used in the economic evaluation 61](#_Toc148882350)

[Table U: Change in exposures compared to baseline by scenario, age group and sex 97](#_Toc148882351)

[Table V: Health and economic impact of different sensitivity analyses I 99](#_Toc148882352)

[Table W: Health and economic impact of different sensitivity analyses II 101](#_Toc148882353)

[Table X: Health and economic impact using different discount rates for QALYs and costs 103](#_Toc148882354)

[Table Y: Health and economic impact of different SSB taxation scenarios in Germany 2023-2043 by age group 107](#_Toc148882355)

[Table Z: Health and economic impact of SSB taxation scenarios in Germany 2023-2043 by sex 111](#_Toc148882356)

[Table AA: Health and economic impact of SSB taxation scenarios in Germany 2023-2043 by included exposures 114](#_Toc148882357)

[Table AB: Health impact of different SSB taxation scenarios in Germany 2023-2043 by simulation model 117](#_Toc148882358)

# Methods A: Detailed technical description of IMPACT_NCD_ Germany

## Introduction

IMPACT_NCD_ is an open-source microsimulation modelling framework for public health policy planning and decision-making in non-communicable disease (NCD) prevention. The epidemiological engine of the modelling framework translates changes in the trends of disease risk factors into changes in disease incidence and case fatality, and subsequent disease prevalence. The policy layer of the framework complements the epidemiological engine and translates policy changes (hypothetical or real) into changes in the trends of disease risk factors.

Models from the IMPACT_NCD_ family have been used extensively to model primary prevention policies nationally in England, Brazil, and the United States (US), and locally in Liverpool [1-8]. A simplified diagram of the model structure is presented in **Figure A**. For this study we have developed a version of IMPACT_NCD_ for Germany: IMPACT_NCD_ Germany.

Figure A: Conceptual structure of IMPACT_NCD_


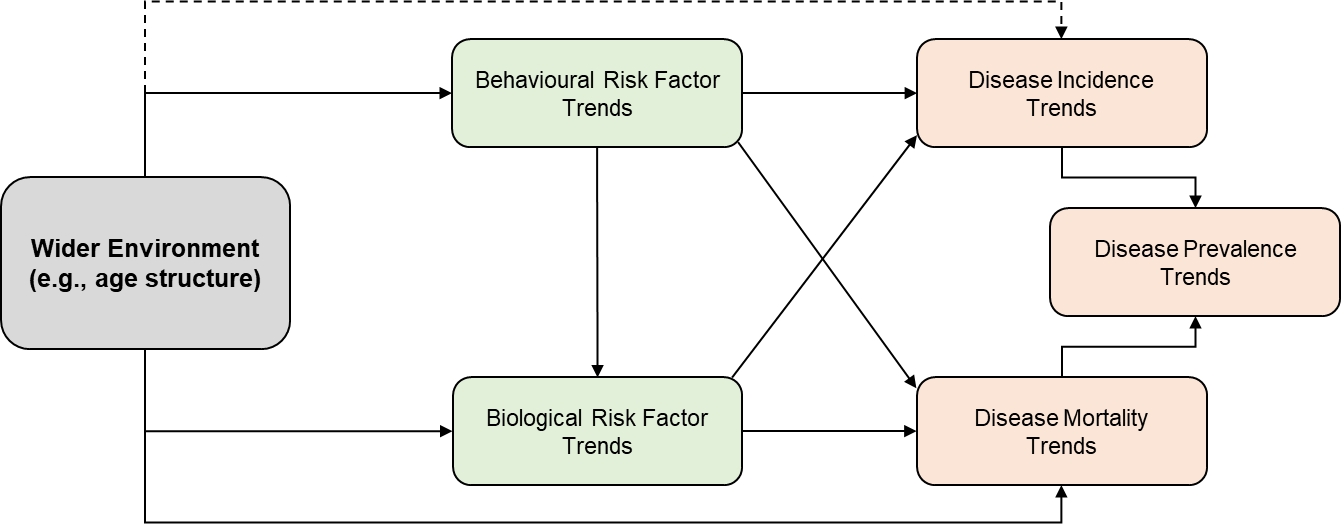


Conceptual structure of the IMPACT_NCD_ modeling framework with its different layers.

At the core of the model is an epidemiological engine which, in the case of IMPACT_NCD_ Germany, includes age, sex, body mass index, and sugar-sweetened beverage (SSB) consumption, as risk factors. The diseases modelled from risk factor trends include coronary heart disease (CHD), stroke, and type 2 diabetes mellitus (T2DM). Notably, diseases can be risk factors for other diseases (i.e., diabetes mellitus is a risk factor for coronary heart disease). The policy layer is dedicated to modelling either a specific public health policy formulation or a hypothetical scenario and consists of a mathematical/statistical model of the policy. In this version of the model, the policy layer implements different scenarios for the taxation of SSBs and fruit juice in Germany.

This version of IMPACT_NCD_ Germany uses data from the *Kooperative Gesundheitsforschung in der Region Augsburg* (KORA) study (S4 and follow-ups F4 and FF4; 1999-2014), a population-based cohort in southern Germany, and the nationally representative dietary survey *Nationale Verzehrsstudie* (NVS) II (2006) to inform risk factors [9, 10]. National disease surveillance data, including mortality data from the Federal Health Monitoring System (*Gesundheitsberichterstattung des Bundes*, [www.gbe-bund.de](http://www.gbe-bund.de)) is used to inform trends in disease incidence, prevalence, and disease-specific mortality [11, 12]. Population estimates and projections from the German Federal Statistical Office (GENESIS, www.destatis.de) are used to inform population size and structure, and for calibrating mortality. An overview of all data sources used in the current version of IMPACT_NCD_ Germany and this study is available in **Table A**.

The IMPACT_NCD_ framework can be easily extended by new diseases and exposures. It allows third parties to develop policy layers independently and hook them on the epidemiological engine. The open-source licence (GPLv3) ensures transparency and accountability while it promotes collaborative work throughout development. The full code for IMPACT_NCD_ Germany is available under: <https://github.com/kalleEF/IMPACT-NCD-Germany>.

Table A: Overview of complete data sources used in IMAPCT_NCD_ Germany

| Input data/parameter | Source | Data year | Details | Comments & Limitations | Subjective quality |
| --- | --- | --- | --- | --- | --- |
| *Sociodemographic module* |  |  |  |  |  |
| Population size and composition | Federal statistical office [13] | 2013-2019 | Official population data | - | Very high |
| Population projections | Federal statistical office [14] | 2020-2050 | Official population projections | Underlying assumptions about future mortality, fertility, and migration | High |
| *Exposure module* |  |  |  |  |  |
| BMI | KORA S4, F4, FF4  [9] | 1999, 2007, 2014 | KORA is a population-based research platform in southern Germany with multiple cohorts; NVS II is the last official national German nutrition survey | Representativeness unclear; no recent data | Moderate |
| SSB consumption | NVS II, KORA FF4  [9, 10] | 2006, 2014 |  | Representativeness of KORA FF4 unclear; no recent data | Moderate |
| Fruit juice consumption | NVS II, KORA FF4  [9, 10] | 2006, 2014 |  | Representativeness of KORA FF4 unclear; no recent data | Moderate |
| Sugar intake from SSBs and fruit juice | KORA FF4 [9] | 2014 |  | Representativeness unclear; no recent data | Moderate |
| Proportion of diet soft drinks | KORA FF4 [9] | 2014 |  | Representativeness unclear; no recent data | Moderate |
| Correlation between exposures | KORA FF4 [9] | 2014 |  | Representativeness unclear; no recent data | Moderate |
| *Disease module* |  |  |  |  |  |
| Incidence of type 2 diabetes | Schmidt et al., 2020 [11] | 2011 | Based on the German national diabetes surveillance incorporating data from all individuals insured in the SHI (≈ 85% of the population) | No recent data; based on SHI | Very high |
| Prevalence of type 2 diabetes | Schmidt et al., 2020 [11] | 2011 |  | No recent data; based on SHI | Very high |
| Incidence of CVD | KORA S4, F4, FF4 (SCORE-2, SCORE-2 OP) [15, 16] | 1999, 2007, 2014 | SCORE-2 (OP) are validated European cardiovascular risk equations | Indirect evidence but no measured incidence; processed with DISMOD II (CHD & stroke separately) | Low |
| Proportion of CHD and stroke of total CVD incidence | Ricci et al., 2018 [17] | 1991-2010 | Based on the *European Prospective Investigation into Cancer and Nutrition* study | Representativeness not guaranteed | Moderate |
| Prevalence of CHD | HSE [18] | 2013 | The HSE is the official English health survey | No national German prevalence; self-reported; processed with DISMOD II | High |
| Prevalence of stroke | HSE [18] | 2013 |  | No national German prevalence; self-reported; processed with DISMOD II | High |
| Case fatality of CHD | DISMOD II [19] | 2014 |  | Subject to DISMOD assumptions | Moderate |
| Case fatality of stroke | DISMOD II [19] | 2014 |  | Subject to DISMOD assumptions | Moderate |
| Non-CVD (i.e., non-modelled) mortality | Federal health reporting system [12] | 1991-2019 | Official mortality data | Smoothed and disaggregated with R package *demography* | Very high |
|  | Forecast with functional demographic model [20] | 2020-2050 |  | Mortality in future years cannot be observed; established forecasting method | Very high |
| Effect of sugar intake on BMI | Micha et al., 2017 [21]; Huang et al., [8] | n/a | Meta-analysis of three cohort studies | Source study measured exposure in SSB servings; effect estimate assumes 20g of sugar per 8 fluid ounces (≈ 227ml) of SSB; the same effect is assumed for sugar from fruit juice; age stratification from Huang et al., [8] | Very high |
| Effect of SSBs on CHD | Xi et al., 2015 [22]; Huang et al., [8] |  | Meta analysis of four cohort studies | We assumed 20g of sugar per 227ml; adjusted for BMI; age stratification from Huang et al., [8]; the assessment of the direct effect of single dietary components on disease outcomes is challenging and potentially subject to residual confounding despite sophisticated statistical analysis [23] | Very high |
| Effect of SSBs on type 2 diabetes | Imamura et al., 2015 [24]; Huang et al., [8] |  | Meta-analysis of 17 cohort studies |  | Very high |
| Effect of BMI on CHD | Lu et al., 2014 [25]; Huang et al., [8] | n/a | Meta-analysis of 97 cohort studies | Estimates are adjusted for glucose as a proxy for diabetes (authors report no significant differences between glucose and diabetes adjusted models); age stratification from Huang et al., [8] | Very high |
| Effect of BMI on stroke | Lu et al., 2014 [25]; Huang et al., [8] |  |  |  | Very high |
| Effect of BMI on type 2 diabetes | Singh et al., 2013 [26]; Huang et al., [8] |  | Pooled analysis of international cohort pooling projects (on average 123 cohorts with data on 1.4 million individuals) | Estimate is adjusted for CHD and stroke; values for youngest age group from Huang et al., [8] | Very high |
| Effect of type 2 diabetes on CHD | Sarwar et al., 2010 [27] |  | Meta-analysis of 102 prospective studies | Estimates are adjusted for sex, smoking status, BMI, and systolic blood pressure; estimate for ischemic stroke was used | Very high |
| Effect of type 2 diabetes on stroke | Sarwar et al., 2010 [27] |  |  |  | Very high |
| Effect of type 2 diabetes on non-CVD mortality | Stringhini et al., 2017 [28] |  | Analysis based on individual data from 48 independent prospective cohort studies | Estimate is adjusted for age, sex and ethnicity | Very high |
| *Policy module* |  |  |  |  |  |
| Own- and cross-price elasticities of SSBs and fruit juice | Own calculations | 2013 & 2018 | Based on official German household consumption survey (EVS) | Subject to assumptions of Almost Ideal Demand System | High |
| Pass-through of SSB tax | Andreyeva et al., 2022 [29] | n/a | Meta-analysis of 41 studies | Recent meta-analysis but subject to limitations of included primary studies | High |
| Size of SSB tax | International studies & WHO [30, 31] | n/a |  | Based on recommendations and implemented taxes | Very high |
| Sugar reduction through SSB reformulation | von Philipsborn et al., 2023 [32] | 2011-2021 | Evaluation of the sales-weighted reduction in sugar content in UK soft drinks | Based on Euromonitor data | Moderate |
| Time until BMI change | Assumption | n/a | Assumed to be three years | Metabolic processes change over time and not instantaneously | Moderate |
| *Health economics module* |  |  |  |  |  |
| Medical costs of type 2 diabetes | Kähm et al., 2018 [33] | 2013-2015 | Based on large samples of individuals insured in the German SHI system | See **Table J** | Very high |
| Medical costs of CHD | Kähm et al., 2018 [33] | 2015 |  |  | High |
| Medical costs of stroke | Kähm et al., 2018 [33] | 2015 |  |  | High |
| Medical costs of otherwise healthy individuals | Kähm et al., 2020 [34] | 2015 |  |  | Very high |
| Costs associated with early retirement (type 2 diabetes) | Ulrich et al., 2016 [35] | 2004-2011 | Based on the KORA research platform (see above) |  | Moderate |
| Costs associated with sick leave days (type 2 diabetes) | Ulrich et al., 2016 [35] | 2004-2011 |  |  | Moderate |
| Costs associated with early retirement (stroke) | Winter et al., 2008 [36] | 1999 |  |  | Very low |
| Costs associated with sick leave days (stroke) | Winter et al., 2008 [36] | 1999 |  |  | Very low |
| Costs associated with time for self-management (type 2 diabetes) | Icks et al., 2020 [37] | 2014 | Based on the KORA research platform (see above) |  | Moderate |
| Costs associated with use of health services | Icks et al., 2013 [38] | 1999 |  |  | Moderate |
| Mean annual gross wage | Official national salary assessment [39] | 2018 |  |  | Very high |
| Fringe benefit rate | Official national assessment of employers’ social security contributions [40] | 2020 |  |  | Very high |
| Consumer price index – Health | Official German COICOP [41] | 2003-2022 |  |  | Very high |
| Price index for labour costs | DeStatis GENESIS [42] | 2003-2022 |  |  | Very high |
| Health utility decrements | Re-estimated based on Laxy et al., 2021 [43] | 2016 |  | See section Health-related quality of life | High |

Abbreviations: BMI, body mass index; CHD, coronary heart disease; COICOP, Classification of Individual Consumption by Purpose; CVD, cardiovascular disease; HSE, Health Survey of England; KORA, Kooperative Gesundheitsforschung in der Region Augsburg; NVS, Nationale Verzehrsstudie; SHI, statuatory health insurance; SSBs, sugar-sweetened beverages; WHO, World Health Organisation.

## Epidemiological engine

### High-level description

The epidemiological engine of IMPACT_NCD_ Germany is a discrete-time, dynamic, stochastic microsimulation, consisting of three modules: the sociodemographic module, the exposure module, and the disease module. The policy and health economics modules are two additional modules that can be used to interact with the epidemiological engine or process its outputs. Within the IMPACT_NCD_ Germany epidemiological engine, each unit is a synthetic individual (simulant) represented by a record containing a unique identifier and a set of associated attributes. The microsimulation then projects the life-course of each synthetic individual. The attributes of each synthetic individual include sociodemographic characteristics, exposures to risk factors, acquired diseases, and cause of death if relevant.

Specific attributes include:

1. Age and sex as sociodemographic exposures.

2. Consumption of SSBs as the main behavioural risk exposure variable. We additionally include consumption of fruit juice and an individual amount of sugar in grams per millilitre of SSB and fruit juice to calculate sugar intake from these beverages (the latter are quasi-exposures and only used to calculate the individual net reduction in sugar intake from beverages in each scenario; described in the policy module section).

3. Body mass index (BMI) as the main biological risk exposure.

4. The risk for the three diseases is modelled explicitly from relevant exposures to sociodemographic, behavioural, and biological risk factor attributes; CHD, stroke, T2DM. The Disease module section summarises the modelling approach and gives more detail on the modelling and data sources for each condition. Based on their risk to develop any of these conditions, simulants will develop some of these during their life course.

5. Mortality from the diseases listed in 4 or any other cause is recorded if it occurs.

All these attributes are updated in discrete annual steps according to a set of stochastic rules. We structured these rules, based on well-established epidemiological principles. Specifically, behavioural risk exposures are conditional on sociodemographic exposures; biological risk exposures are conditional on behavioural and sociodemographic exposures, and diseases are conditional on biological, behavioural, and sociodemographic exposures. Finally, mortality is conditional on sociodemographic, behavioural, and disease exposures.

The life course of synthetic individuals is simulated as many times as the number of scenarios to be modelled, using the same random numbers for all policy scenarios to reduce stochastic noise. One of the scenarios is always the ‘baseline’ scenario with which all remaining policy scenarios are compared. In this study, the baseline is a scenario without SSB taxation in Germany. The comparison of the disease outcomes from the life courses under the baseline scenario versus the policy scenarios generates the health impact of the policy scenarios. The output of the epidemiological engine is a dataset that contains the adult life course of the simulated synthetic individuals with all the attributes mentioned above recorded on an annual basis for every scenario. From this dataset of life course trajectories, summary measures such as mortality, disease incidence and disease prevalence can be calculated annually.

As we mentioned above, the epidemiological engine of IMPACT_NCD_ Germany consists of three modules: the sociodemographic module, the exposure module, and the disease module. In the following paragraphs, we will describe these three modules. **Table B** summarises the key assumptions and limitations of the IMPACT_NCD_ microsimulation model.

Table B: Key assumptions and limitations of IMPACT_NCD_ Germany

| Model component | **Key assumptions** |
| --- | --- |
| Sociodemographic module | Migration is not modelled explicitly in the model. However, the model outputs are calibrated to official population projections, which take into account migration. Nevertheless, we assume that migrants have similar characteristics to the indigenous population. |
|  | Social mobility is not considered. |
|  | We do not consider socioeconomic strata such as income or education due to data limitations. While this does not allow us to model the equity impact of policy scenarios, the overall population results are not affected. |
| Exposure module | We assume that the surveys used are truly representative of the population. |
|  | On average, simulants remain in the same exposure quantile throughout their life course. |
|  | The linear correlations in exposure quantiles remain constant over time (i.e., the clustering of exposures in some subpopulations). |
|  | We assume that the age-sex-specific distribution of exposures for which we are unable to include time trends remains the same over the simulation period. |
| Disease module | We assume multiplicative risk effects. |
|  | We assume log-linear dose-response for the continuous risk factors. |
|  | We assume that the effects of the risk factors on incidence and mortality are equal. |
|  | We assume a mean lag time between exposure and outcome that is about 4-5 years for most exposure/outcome pairs. |
|  | We assume 100% risk reversibility for all exposures. |
|  | We assume that trends in disease incidence are attributable mainly to trends of the relevant modelled risk factors or other diseases modelled. Since we model only few risk factors, we assume a further reduction in the non-modelled disease incidence of 3% per year. |
|  | We assume that the disease epidemiological data we use is representative of the adult population of Germany. |
| Baseline scenario  (No SSB tax) | We assume that, over the simulation period, no other policies affect consumption of SSBs or their sugar content (i.e., *ceteris paribus*). |
| Scenario 1 – 20% ad-valorem tax on SSBs (“*ad-valorem* tax”) | We assume that own- and cross-price elasticities of beverage demand are homogenous across subpopulations and can be validly estimated with the economic method we used.  We assume that the policy immediately affects consumption. |
| Scenario 2 – 20% ad-valorem tax on SSBs and fruit juice (“extended *ad-valorem* tax”) | We assume that the reduction in demand of SSBs and fruit juice would be additive if they were both taxed at the same time.  We assume that the policy immediately affects consumption. |
| Scenario 3 – Tiered tax leading to reformulation of SSBs by 30% (“tiered tax”) | We assume that the introduction of a tiered tax (i.e., higher tax rates for SSBs with higher sugar content), such as introduced in the UK, would incentivize SSB producers to reformulate the sugar content of SSBs in Germany to avoid the tax [44].  We assume that this tax would lead to reformulation that reduces sugar content by 30% over three years, as observed in a recent analysis [32]. |

Abbreviations: SSB, sugar-sweetened beverages; UK, United Kingdom.

### Sociodemographic module

The first year of every simulation in IMPACT_NCD_ Germany is 2013, this allows us to use the overlapping period 2013-2019 to calibrate and validate the model and ensures alignment with available data.

For each simulation iteration, the algorithm in the module draws 200,000 synthetic individuals, aged 30 to 90 years, from the joint age- and sex-distribution of the German population for 2013 which is informed by official population estimates. Since we do not use further stratification variables, such as geographic information or ethnicity in this version of IMPACT_NCD_ Germany due to data limitations, the sociodemographic module is comparably simple.

So far, the algorithm has created a synthetic population that is a snapshot of the German population in 2013. The following steps of the algorithm create backward and forward projections of the synthetic population that are essential to model exposure time trends and time lags between exposures and diseases.

The backward projection of the synthetic population goes back to 2003; therefore, the maximum time lag we allow in the model is ten years. As everyone alive and older than 30 years old in 2013 was alive in 2003, the algorithm simply creates the back projections by appropriately reducing the age of the synthetic individuals, while keeping constant all other sociodemographic variables (i.e., sex).

Similarly, for the forward projections, we project until the year 2043, and the algorithm increases the age of the synthetic individuals while keeping all other sociodemographic variables constant. For forward projections, mortality needs to be considered. We describe mortality with the disease module as disease-specific mortality which is closely related to disease prevalence. IMPACT_NCD_ follows an open cohort approach. Every simulated year from 2013 onwards, a new cohort of 30 years old synthetic individuals enters the model. The same sources inform the size of the cohort and the joint age- and sex-distribution we described above. For example, in 2014, the new 30-year-old cohort will be informed by the population size and the joint age- sex- ethnicity-distribution of those who were 29 years old in 2013.

The final model outputs are scaled up to the national German population by using appropriate population weights. These weights are informed by official population counts and population projections retrieved from the German Federal Statistical Office (GENESIS, [www.destatis.de](http://www.destatis.de)) [13, 14].

### Exposure module

#### Data sources

This module simulates the adult life course exposures of the synthetic individuals based on the KORA S4 (1999) study with its follow-ups F4 and FF4 (2007 and 2014) and the NVS II (2006). Details on these studies are given elsewhere [9, 10]. Besides their limitations, KORA and the NVS II are the two best national exposure input data sources for our application.

##### KORA

KORA is a population-based research platform with multiple follow-up studies in epidemiology, health economics and health services research [9]. The KORA S4 study was designed to be representative of the German population. However, representativeness cannot be guaranteed anymore due to attrition of the closed cohort over time. At all assessment time points of the KORA S4 study, information on a large amount of genetic, clinical, psychometric, behavioural, sociodemographic, and economic variables of participants was collected in standardized interviews conducted by professional study staff at the study centre. The follow-up FF4 of the KORA S4 study conducted in 2014 also included a nutrition module making it the most recent available detailed dietary data for the adult German population [45].

Estimation of usual dietary intake in KORA FF4 was based on a combination of 24-h food lists and a food frequency questionnaire. Granular information on the intake of a variety of food groups, micro- and macronutrients is available. Importantly, the intake of nutrients from specific food groups can also be estimated. This is key for our application as it allows the calculation of sugar intake from SSBs and fruit juice besides total sugar intake. It is further possible to distinguish between diet soft drinks and SSBs.

Since it is ideal to consider the correlation between exposures (as described below), the KORA S4, F4 and FF4 studies enable us to have comparably recent nutritional, as well as anthropometric data in one single dataset. However, a key limitation of the KORA FF4 nutritional data is that it contains only limited information on the usual diet of younger age groups due to the maturity of the cohort. Particularly for SSBs, intake is highest in children and adolescents, progressively decreasing with age [46]. While we do not model children and adolescents in this study, ignoring the skew in the SSB intake distribution across age in the statistical model which we use, might lead to an underestimation of the overall SSB intake in the population that we model. We therefore considered the NVS II as a supplementary dataset to allow for a more accurate estimation of beverage intake distributions (see detailed explanation below).

##### NVS II

The NVS II study is representative of the German population and unfortunately the last German national nutrition survey despite being conducted over a decade ago in 2006 [10]. In the NVS II information on anthropometrics, as well as information on the intake of several food groups, micro- and macronutrients is available. Estimation of usual dietary intake was based on the average of two 24h dietary recalls which were telephone administered on two non-consecutive days and one to six weeks apart. Energy and nutrient intake were calculated based on the German federal food database (*Bundeslebensmittelschlüssel*, <https://blsdb.de/>). Population-representative dietary intakes can be estimated using the available survey weights. How the NVS II was integrated in the estimation of the usual intake of SSBs and fruit juice is described and justified in detail below.

#### Exposure estimation procedure

For all simulated exposures (**Table C**), we follow the same general principles. First, we fit an appropriate statistical model to the KORA/NVS II data with the exposure of interest as the dependent variable, and some functions of age, sex and, if possible, year as independent variables. Then, we use the statistical model to predict the exposure level of every synthetic individual in the simulation, based on their sociodemographic characteristics that were estimated from the sociodemographic module. In the context of this study, we estimate multiple quasi-exposures, which constitute characteristics of the synthetic individuals but are not directly linked to the causal epidemiological mechanisms explicitly modelled (see Disease Module). These quasi-exposures are however important for the estimation of the individual net reduction in sugar intake from beverages in each policy scenario.

The inclusion of year as an independent variable in some of our exposure models allows us to extract time trends and project them into the future. Furthermore, it allows us to make backward projections of exposures that we use when we simulate time lags. For example, for a synthetic female individual aged 30 years in 2013, we can estimate her BMI in 2003 when she was 20 and in 2033 when she will be 50 years old. For exposures for which we were not able to include time trends due to data limitations we assume that their age-sex-specific distribution remains constant over the simulation period.

We used a *Generalised Additive Models for Location, Scale, and Shape* (GAMLSS) approach to estimate the distribution of exposures [47, 48]. GAMLSS models are flexible statistical models that can make all parameters of an assumed distribution for the dependent variable, conditional to some function of the independent variables. For example, GAMLSS can model both the mean and the standard deviation of a dependent normally distributed variable, while a linear regression only models the mean. A full overview of the capabilities of GAMLSS models is given in the books by Stasinopoulos et al. (2017) and Rigby et al. (2019) [47, 48]. In all cases, the selection of the best fitting distribution and model structure (e.g., inclusion of interaction effects between independent variables, splines, or distribution mixtures) is based on the generalised Akaike information criterion (GAIC). In the next sections we describe details of how the distribution for each exposure was estimated.

Table C: Overview of individual exposures and their use in the simulation model

| Modelled exposure | Unit | Type | Used for… |
| --- | --- | --- | --- |
| BMI | kg/m² | Exposure | Disease modelling |
| SSB intake | ml/day | Exposure | Disease modelling |
| Proportion of diet soft drinks | % | Quasi-exposure | Adjustment of SSB intake for diet soft drinks |
| Fruit juice intake | ml/day | Quasi-exposure | Estimation of intake of sugar from beverages; consideration of substitution to untaxed beverages |
| Sugar intake per SSB | g/ml | Quasi-exposure | Estimation of sugar from SSBs |
| Sugar intake per fruit juice | g/ml | Quasi-exposure | Estimation of sugar from fruit juice |

Abbreviations: BMI, body mass index; g, gram; kg, kilogram; m², square metre; ml, millilitre; SSB, sugar-sweetened beverages.

#### Estimation of the BMI distribution

To estimate the distribution of BMI in the German population, we used data from 9,564 observations across the KORA S4 (1999), F4 (2007) and FF4 (2014) studies. In the KORA study BMI was measured by study staff. Information from all three assessment timepoints allows us to estimate age-sex-specific trends in BMI. We therefore modelled the distribution of BMI conditional on age, sex, and year as independent variables using a GAMLSS model with a Box-Cox Power Exponential distribution. **Figure B** and **Figure C** display the observed and modelled cumulative distribution of BMI by age, sex, and year. Since not all ages were fully observed in all years, the model extrapolates for these combinations. This approach also allows us to predict the age-sex-specific distribution in future and past unobserved years.

#### Estimation of the SSB intake distribution

To estimate the distribution of SSB intake in the German population, we used data from 1,601 observations from the KORA FF4 (2014) study and 14,429 observations from the NVS II (2006). As described above, usual daily SSB intake in ml/day was calculated based on a combination of 24-h food lists and a food frequency questionnaire in KORA FF4 and based on the average of two 24h dietary recalls in NVS II [10, 45]. The rationale for the combination of the two data sources was that we are interested in estimating a least biased age-sex-specific distribution of SSB intake in Germany. However, the youngest participant in KORA FF4 nutrition module is age 38 years. Although our method allows the extrapolation of usual SSB intakes beyond the age range supported by the data, this procedure would lead to an underestimation of the SSB intake distribution (**Figure D**). We therefore opted to combine both datasets despite that fact that this would be unreasonable in other scientific circumstances. Weighing the advantages and disadvantages in the context of the goal of this step to construct a realistic synthetic German population, we concluded that this procedure is reasonable and justified. In both studies, we adjusted SSB intake for misreporting with the residual method by regressing it on energy intake and sex and predicting the corrected SSB intake with the sex-specific mean energy intake [49].

Comparing the estimated overall mean per-capita SSB intake in litre per year between single and combined data sources with published industry-reported aggregate consumption data further supports our approach which most likely still underestimates SSB consumption in Germany (**Table D**) [50, 51]. The same aggregated consumption figures further indicate that SSB consumption in Germany has only slightly declined over the past decade. **Figure D** shows both observed SSB intake in KORA FF4 and NVS II by age and sex, as well as the results from the estimated distributions based on the KORA FF4-only and combined datasets.

Table D: Comparison of aggregate SSB consumption data from different data sources

| Source | Year | Mean per-capita SSB intake (l/year) |
| --- | --- | --- |
| KORA FF4 | 2014 | 13.47 |
| NVS II | 2006 | 62.51 |
| Combined | n/a | 58.38 |
| Aggregate industry-reported consumption data* | 2020 | 78.20 |
|  | 2014 | 83.50 |
|  | 2006 | 92.00 |

*Source: Entwicklung des Pro-Kopf-Verbrauchs von Alkoholfreien Getränken nach Getränkearten 2012 – 2021 [50, 51]; this number includes sugar-sweetened beverages (SSBs), fruit drinks and other sugary non-diet drinks. Abbreviations: KORA, Kooperative Gesundheitsforschung in der Region Augsburg; l, litre; n/a, not applicable; NVS, Nationale Verzehrsstudie.

We therefore modelled the distribution of SSB intake conditional on age and sex as independent variables using a GAMLSS model on the combined KORA FF4 and NVS II dataset (n = 16,030). To account for the long tail in the SSB intake distribution (i.e., many people rarely consume SSBs, and few people consume large amounts) we use a mixture of two distributions. The selection of the best fitting mixtures is done using a grid search approach over potentially viable distributions available in the GAMLSS package [47]. **Figure E** and **Figure F** display the observed and modelled cumulative distribution of SSB intake by age and sex. Since SSB intake is only available at one time point we are not able to model time trends assume that consumption remains stable. This is supported by observed aggregate consumption data (see above).

#### Estimation of the proportion of diet soft drinks

To estimate the proportion of diet soft drinks in the overall soft drink consumption in the German population, we use data from 1,601 observations from the KORA FF4 (2014) study. To be able to calculate an individual-specific proportion of diet soft drinks, we estimate a GAMLSS model with a Generalised Beta 1 distribution and age and sex as independent variables. **Figure G** displays the observed and modelled proportion of diet soft drinks by age and sex. Since not all relevant age groups are observed in KORA FF4 we use the model to extrapolate to younger ages. The results of this procedure are consistent with findings that the share of consumed diet soft drinks is higher in older age groups [52].

Figure B: Validation of BMI distribution by 10-year age group and year


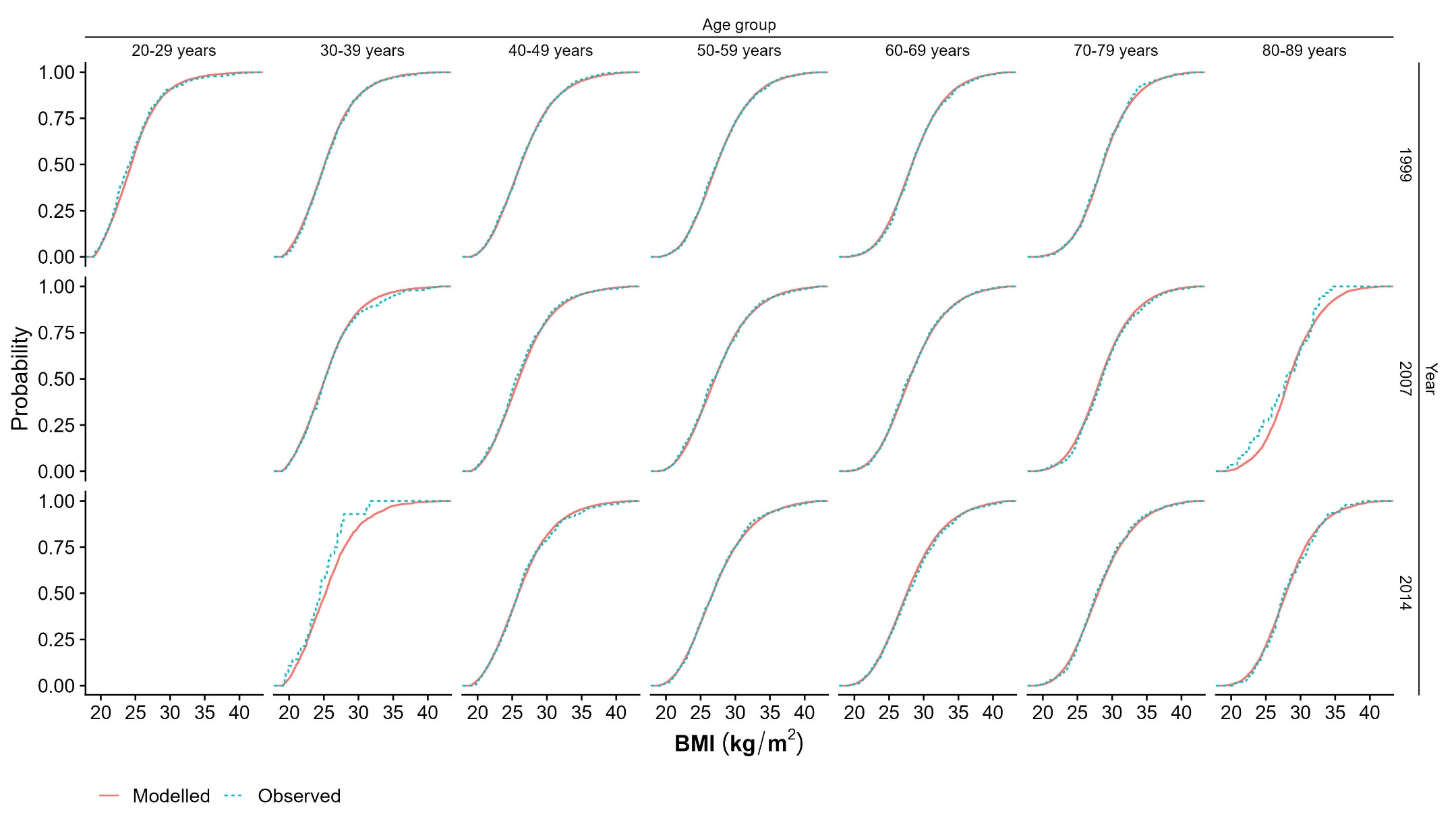
Cumulative distribution plot of the observed (blue dashed lines) and modelled (red lines) BMI distribution by 10-year age groups and year based on the Generlized Additive Models of Location, Shape and Scale (GAMLSS) model. Years indicate the survey year of the Kooperative Gesundheitsforschung in der Region Augsburg (KORA) S4, F4, and FF4 study which were used for the modelling of the BMI distribution. Abbreviations: BMI, body mass index; kg, kilograms; m², square metre.

Figure C: Validation of BMI distribution by sex and year


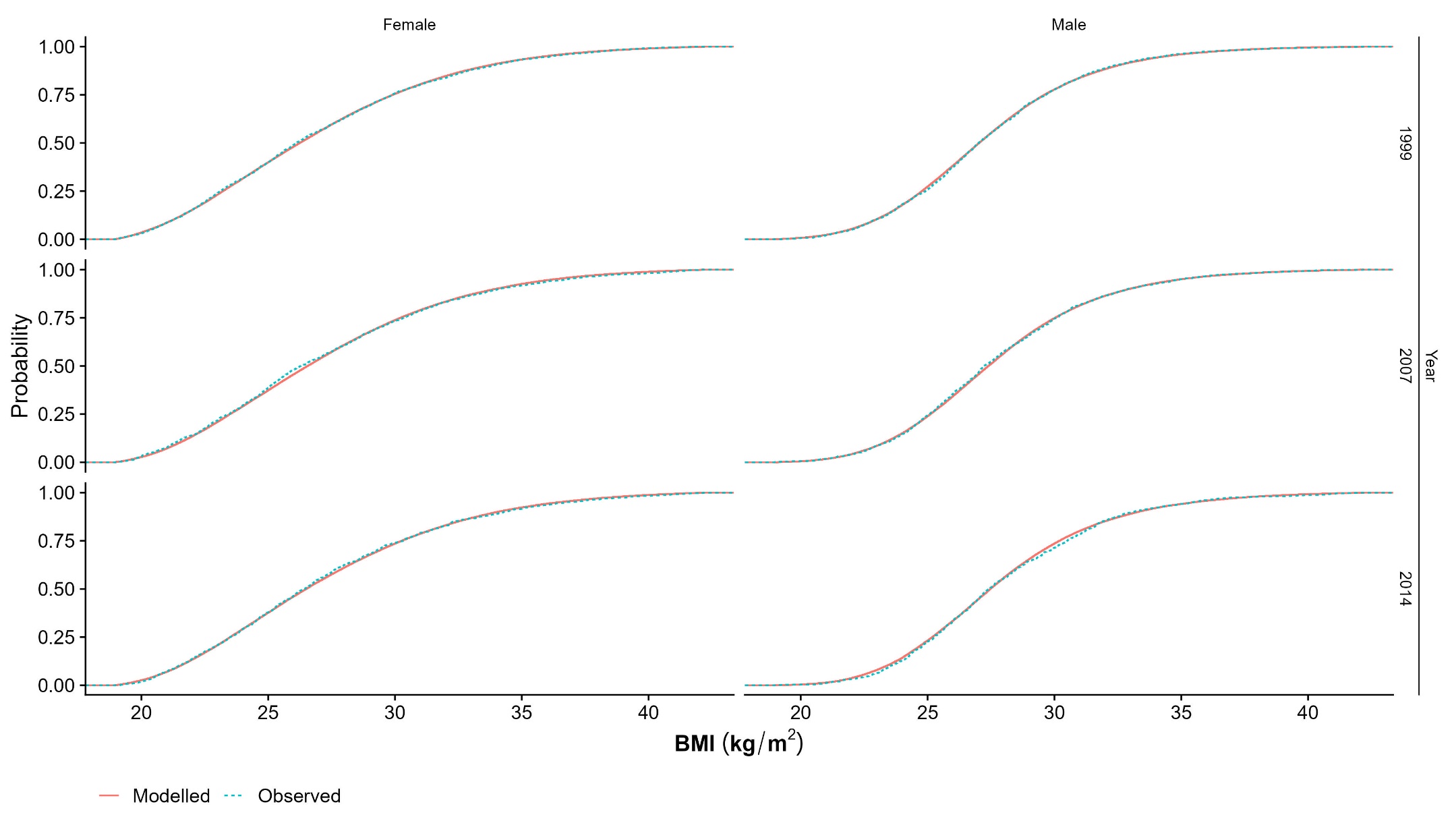
Cumulative distribution plot of the observed (blue dashed lines) and modelled (red lines) BMI distribution by sex and year based on the Generlized Additive Models of Location, Shape and Scale (GAMLSS) model. Years indicate the survey year of the Kooperative Gesundheitsforschung in der Region Augsburg (KORA) S4, F4, and FF4 study which were used for the modelling of the BMI distribution. Abbreviations: BMI, mody mass index; kg, kilograms; m², square metre.

Figure D: Comparison of modelled and observed SSB intake from different data sources by sex and age


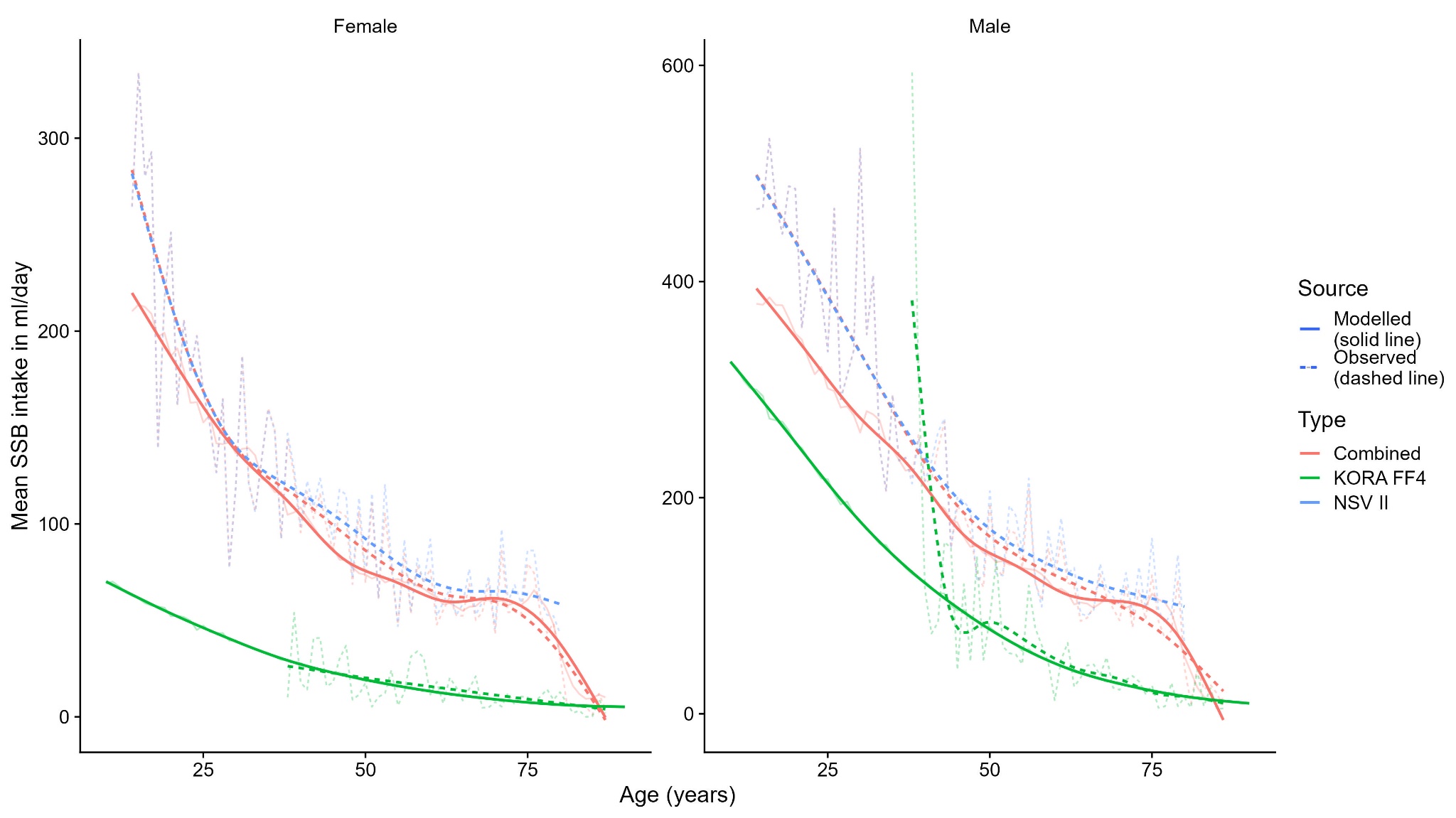


Line plot of the observed (dashed lines) and Generlized Additive Models of Location, Shape and Scale (GAMLSS) modelled (solid lines) mean intake of sugar-sweetened beverages (SSB) by age and sex. Combined data (red lines) includes both Kooperative Gesundheitsforschung in der Region Augsburg (KORA) and Nationale Verzehrsstudie (NVS) II observations to better approximate consumption in younger age groups. Green and blue lines represent only KORA and NVS II data, respectively. Light-colored and full-colored lines are raw and smoothed data, respectively. Abbreviations: ml, millilitre.

Figure E: Validation of SSB intake distribution by 10-year age groups


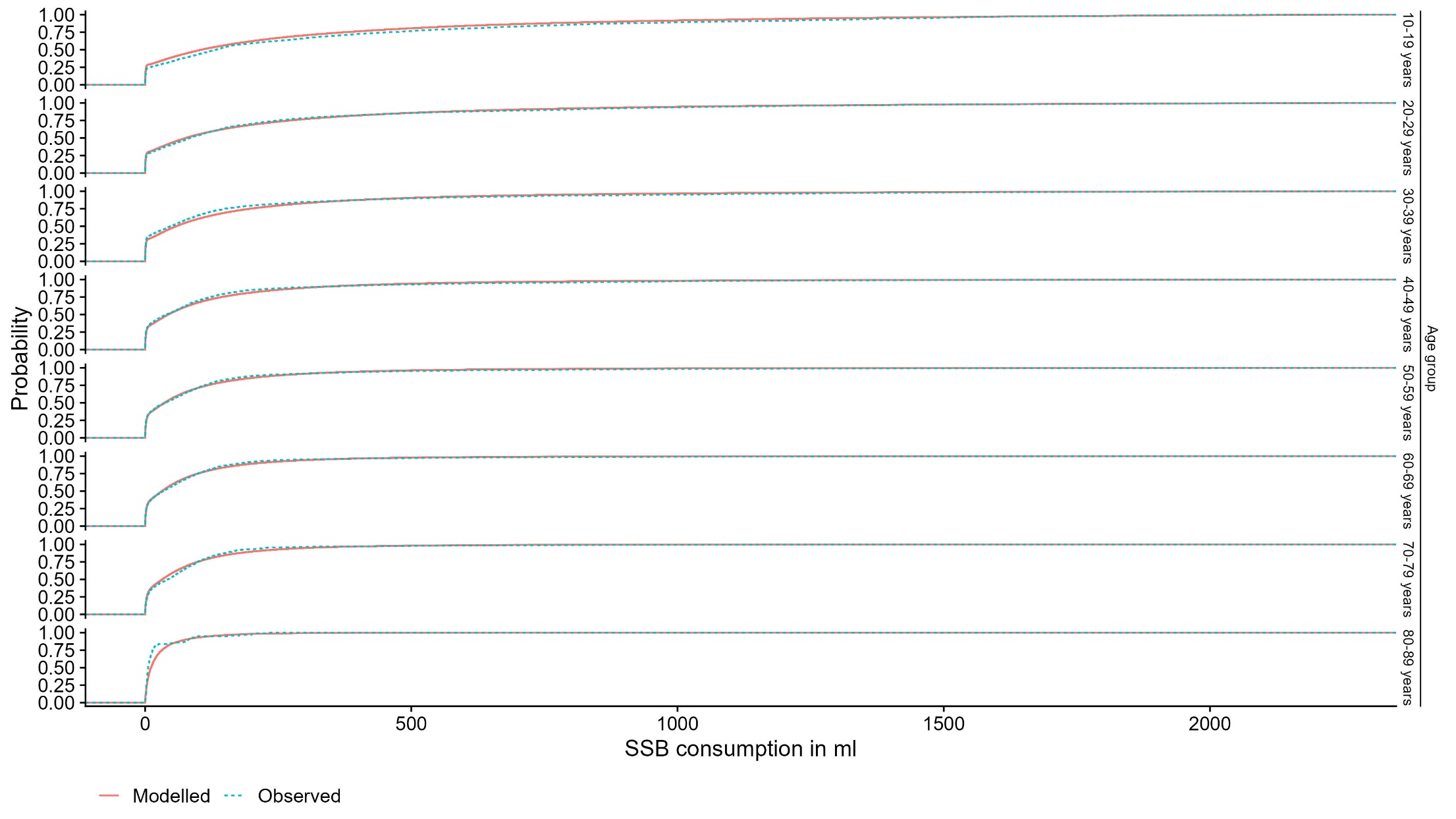
Cumulative distribution plot of the observed (blue dashed lines) and modelled (red lines) SSB intake distribution stratified by 10-year age groups based on the Generlized Additive Models of Location, Shape and Scale (GAMLSS) model. Abbreviations: ml, millilitre; SSB, sugar-sweetened beverages.

Figure F: Validation of SSB intake distribution by sex


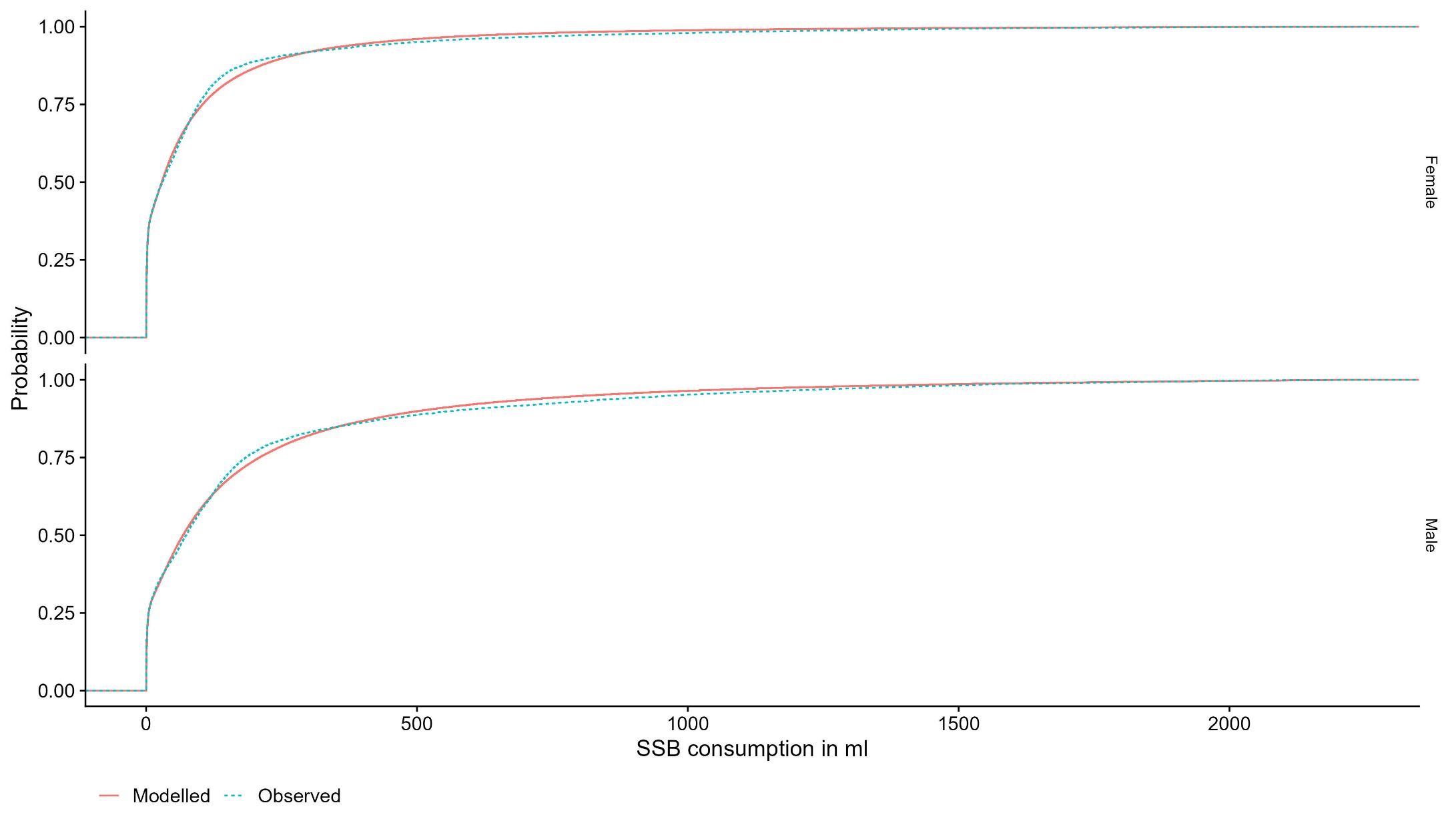
 Cumulative distribution plot of the observed (blue dashed lines) and modelled (red lines) SSB intake distribution by sex based on the Generlized Additive Models of Location, Shape and Scale (GAMLSS) model. Abbreviations: ml, millilitre; SSB, sugar-sweetened beverages.

Figure G: Validation of the proportion of diet soft drinks by sex and age
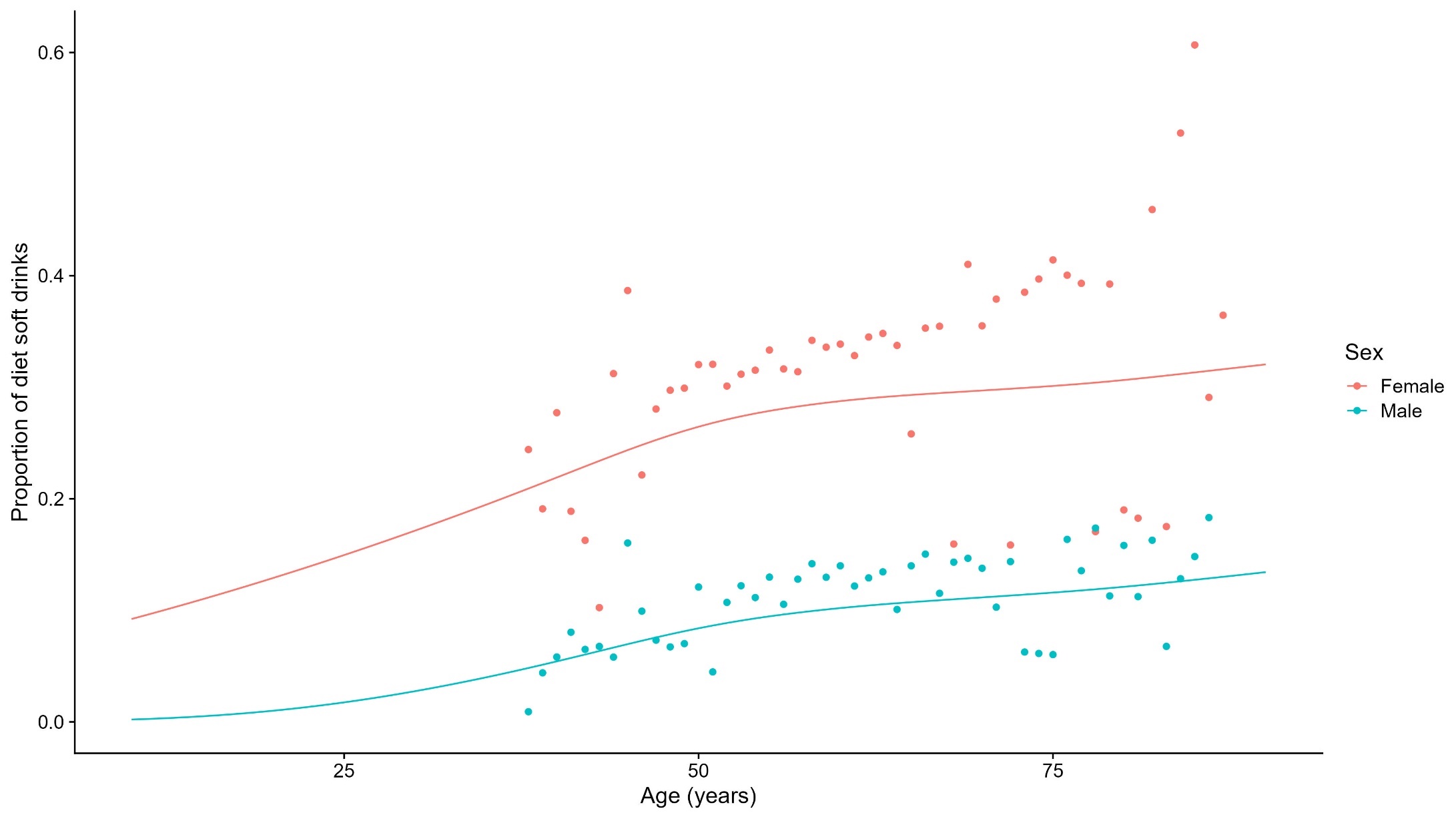


Line plot of the observed (points) and Generlized Additive Models of Location, Shape and Scale (GAMLSS) modelled (lines) mean proportion of diet soft drinks of all soft drinks by age and sex based on the Kooperative Gesundheitsforschung in der Region Augsburg (KORA) FF4 study.

Figure H: Comparison of modelled and observed fruit juice intake from different data sources by sex and age
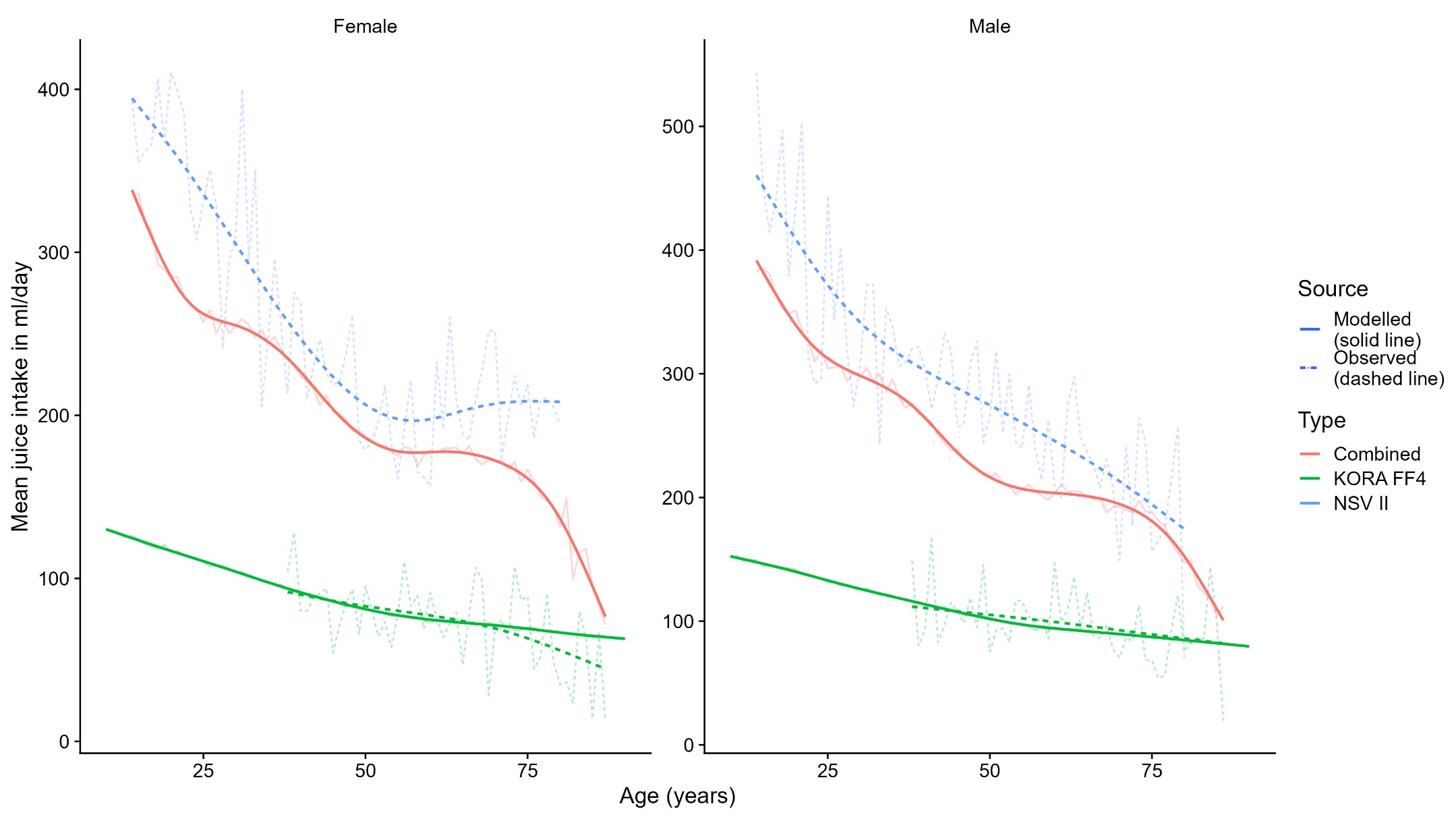


Line plot of the observed (dashed lines) and Generlized Additive Models of Location, Shape and Scale (GAMLSS) modelled (solid lines) mean intake of fruit juice by age and sex. Combined data (red lines) includes both Kooperative Gesundheitsforschung in der Region Augsburg (KORA) and Nationale Verzehrsstudie (NVS) II observations to better approximate consumption in younger age groups. Green and blue lines represent only KORA and NVS II data, respectively. Light-colored and full-colored lines are raw and smoothed data, respectively. Abbreviations: ml, millilitre.

Figure I: Validation of fruit juice intake distribution by 10-year age group
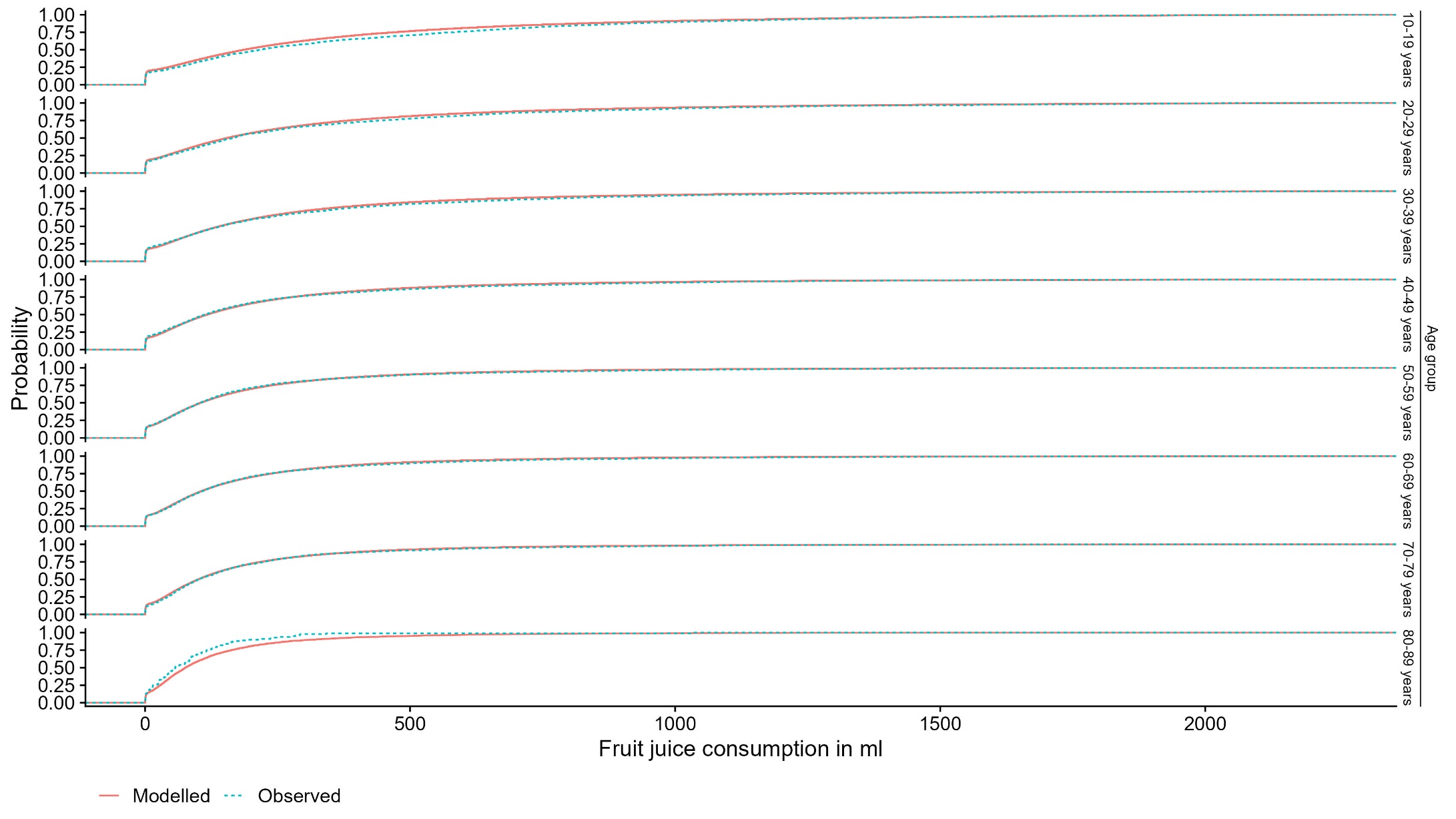


Cumulative distribution plot of the observed (blue dashed lines) and modelled (red lines) fruit juice intake distribution stratified by 10-year age groups based on the Generlized Additive Models of Location, Shape and Scale (GAMLSS) model. Abbreviations: ml, millilitre.

Figure J: Validation of fruit juice intake distribution by sex
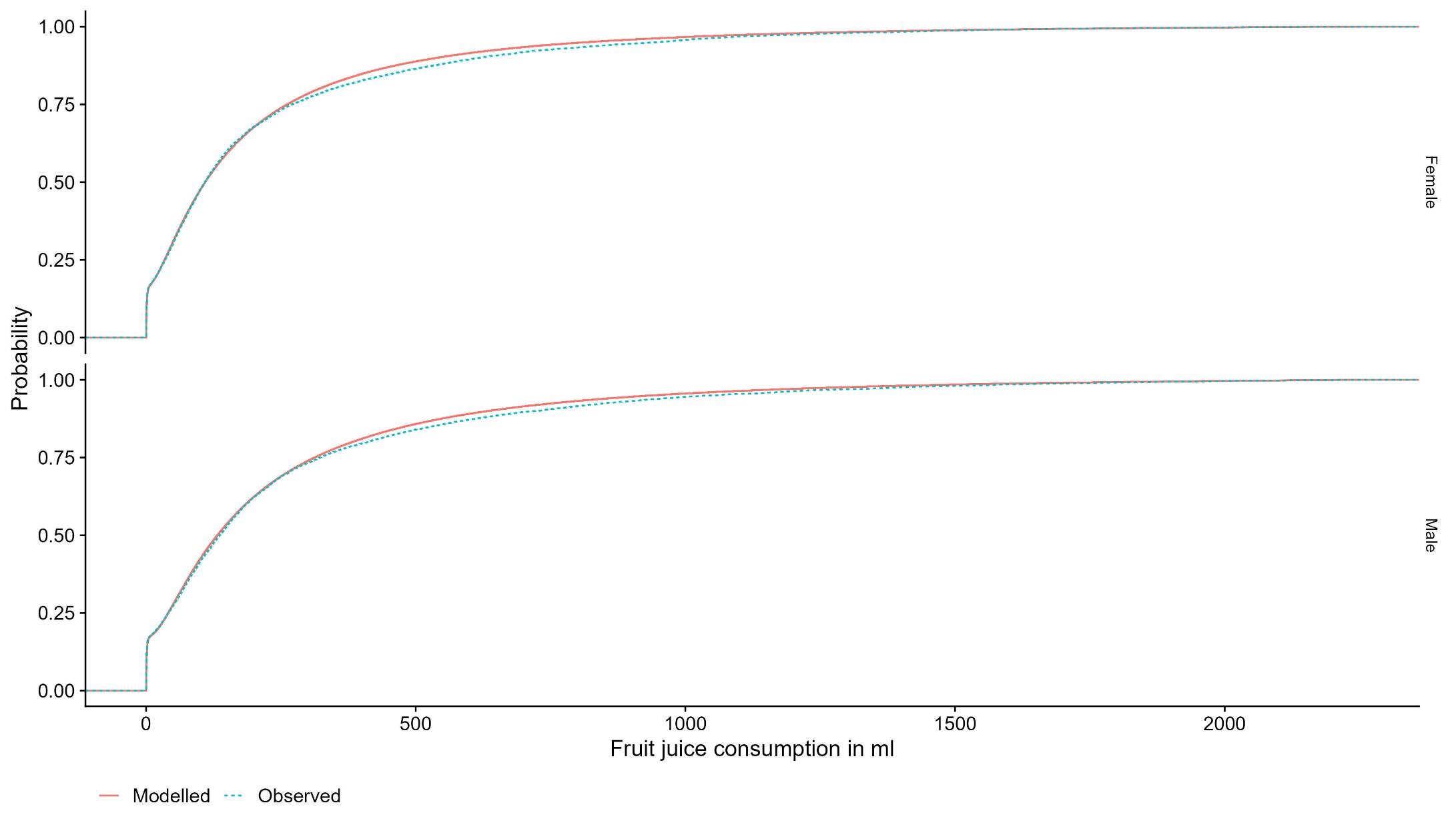


Cumulative distribution plot of the observed (blue dashed lines) and modelled (red lines) fruit juice intake distribution by sex based on the Generlized Additive Models of Location, Shape and Scale (GAMLSS) model. Abbreviations: ml, millilitre.

#### Estimation of the fruit juice intake distribution

To estimate the distribution of fruit juice intake in the German population, we used data from 1,601 observations from the KORA FF4 (2014) study and 14,429 observations from the NVS II (2006). As described above, usual daily fruit juice intake in ml/day was calculated based on a combination of 24-h food lists and a food frequency questionnaire in KORA FF4 and based on the average of two 24h dietary recalls in NVS II [10, 45]. The rationale for the combination of the two data sources is consistency with the approach we take to estimate SSB intake (see above). We therefore opted to combine both datasets for fruit juice as well, despite that fact that this would be unreasonable in other scientific circumstances. Weighing the advantages and disadvantages in the context of the goal of this step to construct a realistic synthetic German population, we concluded that this procedure is reasonable and justified. In both studies, we adjusted fruit juice intake for misreporting with the residual method by regressing it on energy intake and sex and predicting the corrected fruit juice intake with the sex-specific mean energy intake [49].

However, comparing the estimated overall mean per-capita fruit juice intake in litre per year between single and combined data sources with published industry-reported aggregate consumption, we see large differences in estimated consumption (**Table E**) [50, 51]. The same aggregated consumption figures further indicate that fruit juice consumption in Germany has only slightly declined over the past decade. **Figure H** shows both observed fruit juice intake in KORA FF4 and NVS II by age and sex, as well as the results from the estimated distributions based on the KORA FF4-only and combined datasets.

The implications of a potential under- or overestimation of the intake distribution of fruit juice for the results of our study are complex. Overestimation makes scenarios with substitution (i.e., *ad-valorem* and tiered tax scenarios in the main text) more conservative because the mechanism of substitution which we implement via cross-price elasticities is based on a relative change (i.e., if the price of SSBs increases by 1%, the demand for fruit juice increases by X%). However, the policy impact in the extended *ad-valorem* tax scenario, where fruit juice is taxed as well, may be overestimated. Underestimation of fruit juice intake on the other hand may lead to overly optimistic results in scenarios with substitution and underestimate the effects of a tax on fruit juice. Considering these differential implications and to retain consistency with the estimation of SSB intake, we decided to use the combined KORA FF4 and NVS II dataset for the estimation of the fruit juice intake distribution as well. This is the more conservative approach for most scenarios.

**Table E: Comparison of aggregate fruit juice consumption data from different data** sources

| Source | Year | Mean per-capita fruit juice intake (l/year) |
| --- | --- | --- |
| KORA FF4 | 2014 | 32.12 |
| NVS II | 2006 | 99.39 |
| Combined | n/a | 92.73 |
| Aggregate industry-reported consumption data* | 2020 | 30.00 |
|  | 2014 | 32.00 |
|  | 2006 | 39.83 |

*Source: Entwicklung des Pro-Kopf-Verbrauchs von Alkoholfreien Getränken nach Getränkearten 2012 – 2021 [50, 51]. Abbreviations: KORA, Kooperative Gesundheitsforschung in der Region Augsburg; l, litre; n/a, not applicable; NVS, Nationale Verzehrsstudie.

We therefore modelled the distribution of SSB intake conditional on age and sex as independent variables using a GAMLSS model on the combined KORA FF4 and NVS II dataset (n = 16,030). To account for the long tail in the SSB intake distribution (i.e., many people rarely consume SSBs, and few people consume large amounts) we use a mixture of two distributions. The selection of the best fitting mixture (Log-normal 2 and Pareto 2) was done using a grid search approach over potentially viable distributions available in the GAMLSS package [48]. **Figure I** and **Figure J** display the observed and modelled cumulative distribution of SSB intake by age and sex. Since SSB intake is only available at one time point we are not able to model time trends assume that consumption only depends on age and sex. This is supported by observed aggregate consumption data (see above).

#### Estimation of sugar intake from SSBs and fruit juice

To calculate sugar intake from each of the two modelled beverages, SSBs and fruit juice, and account for potentially heterogenous preferences over the life course, we estimated an individual age- and sex-specific amount of sugar per ml for each beverage. For this, we used data from 1,601 observations from the KORA FF4 (2014) study. As described above, usual dietary intake was calculated based on a combination of 24-h food lists and a food frequency questionnaire in KORA FF4. The total and food group-specific intake of micro- and macronutrients was estimated as well. In both studies, we adjusted beverage-specific sugar intake for misreporting with the residual method by regressing it on energy intake and sex and predicting the corrected sugar intake with the sex-specific mean energy intake [49].

We modelled the distribution of the amount of sugar consumed per ml of beverage conditional on age and sex as independent variables using a GAMLSS models with a Box-Cox Power Exponential distribution for both SSBs and fruit juice. **Figure K**, **Figure L**, **Figure M**, and **Figure N** display the observed and modelled cumulative distribution of sugar from SSBs and fruit juice by age and sex, respectively.

**Figure K: Validation of the amount of sugar per ml of SSB by age group**


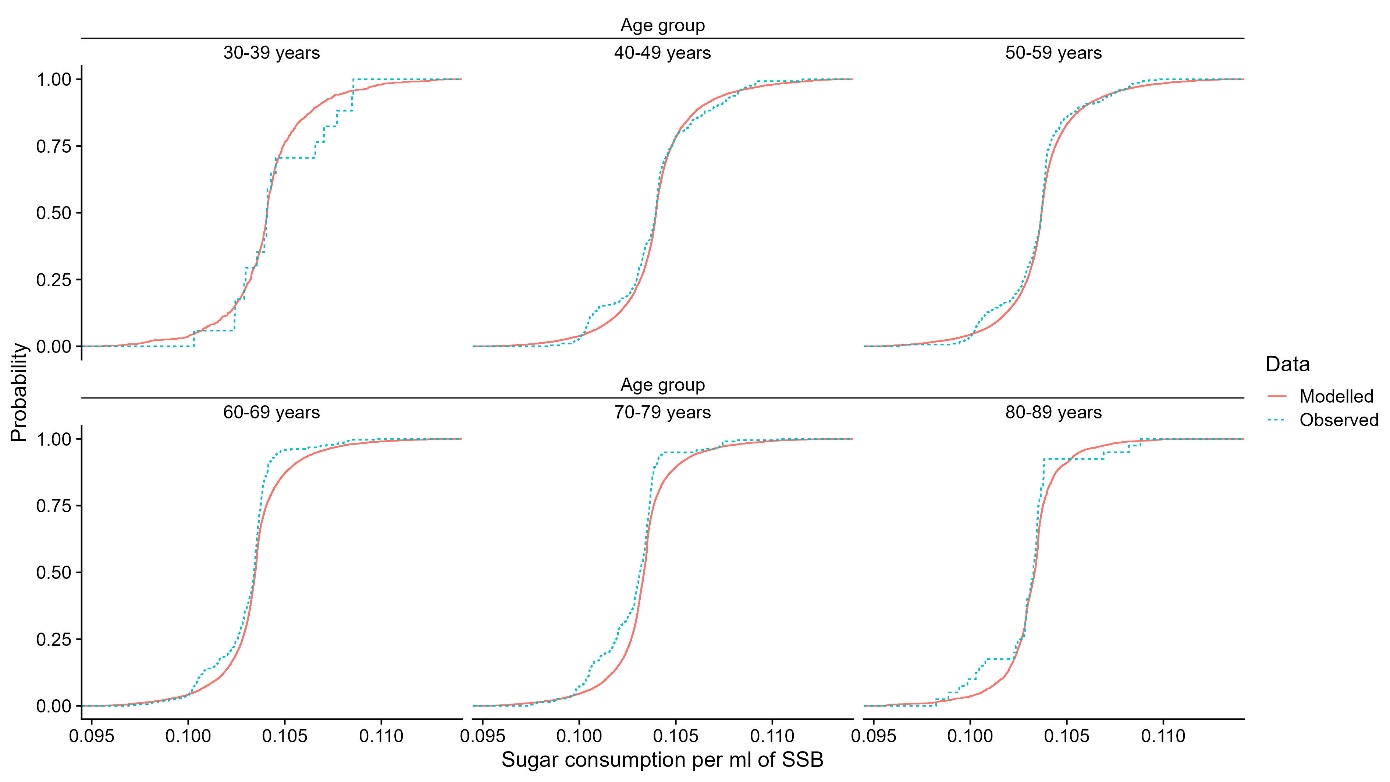


Cumulative distribution plot of the observed (blue dashed lines) and modelled (red lines) distribution of sugar consumption per ml of sugar-sweetened beverage (SSB) by age based on the Generlized Additive Models of Location, Shape and Scale (GAMLSS) model. Abbreviations: ml, millilitre.

Figure L: Validation of the amount of sugar per ml of SSB by sex

**
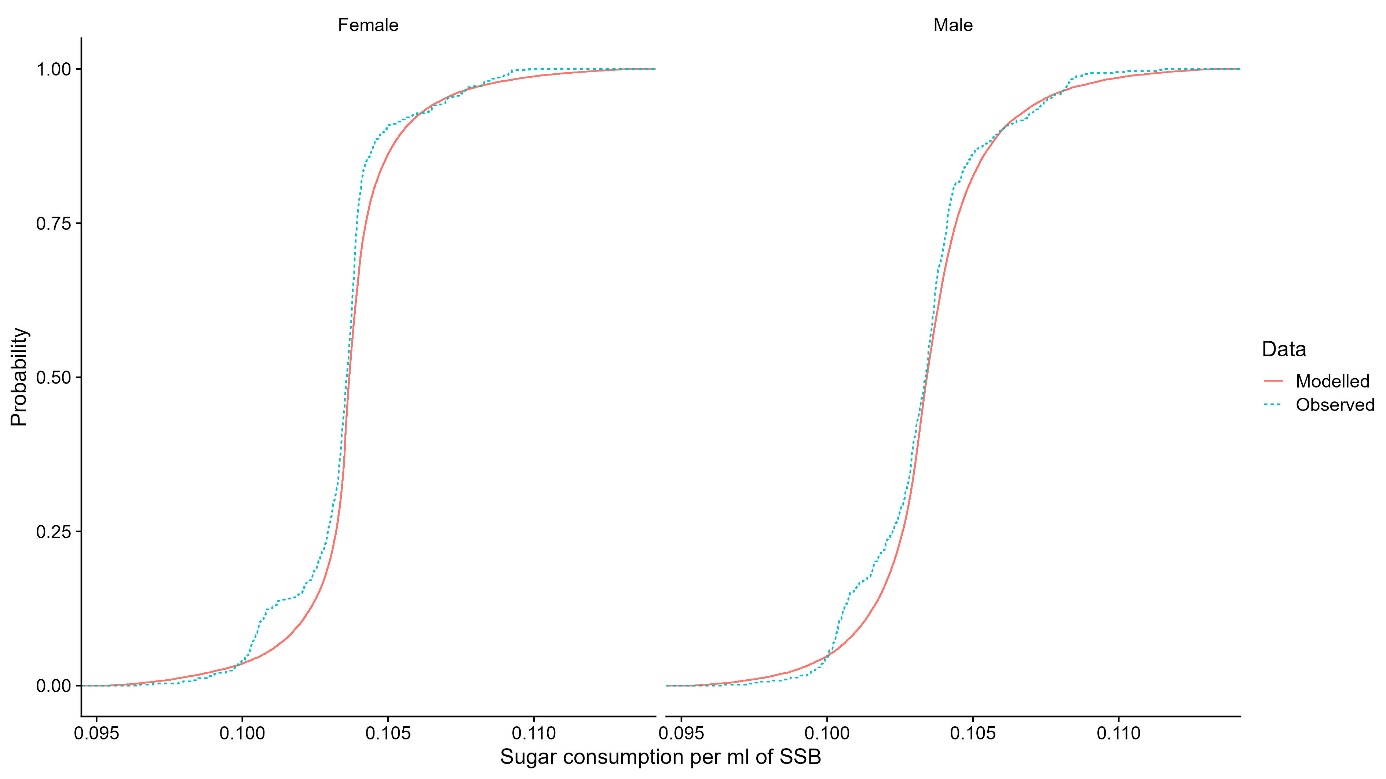
**

Cumulative distribution plot of the observed (blue dashed lines) and modelled (red lines) distribution of sugar consumption per ml of sugar-sweetened beverage (SSB) by sex based on the Generlized Additive Models of Location, Shape and Scale (GAMLSS) model. Abbreviations: ml, millilitre.

Figure M: Validation of the amount of sugar per ml of fruit juice by age group

**
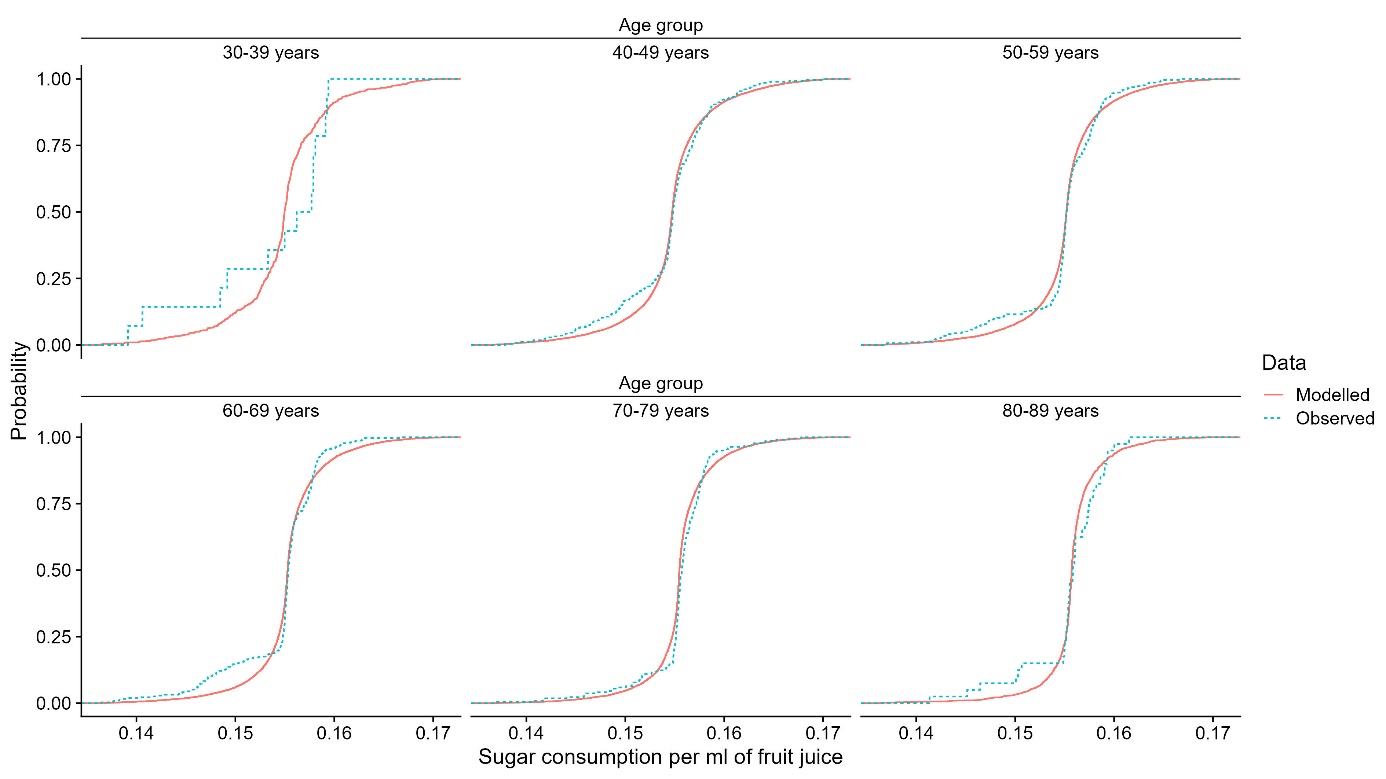
**

Cumulative distribution plot of the observed (blue dashed lines) and modelled (red lines) distribution of sugar consumption per ml of fruit juice by age based on the Generlized Additive Models of Location, Shape and Scale (GAMLSS) model. Abbreviations: ml, millilitre.

Figure N: Validation of the amount of sugar per ml of fruit juice by sex


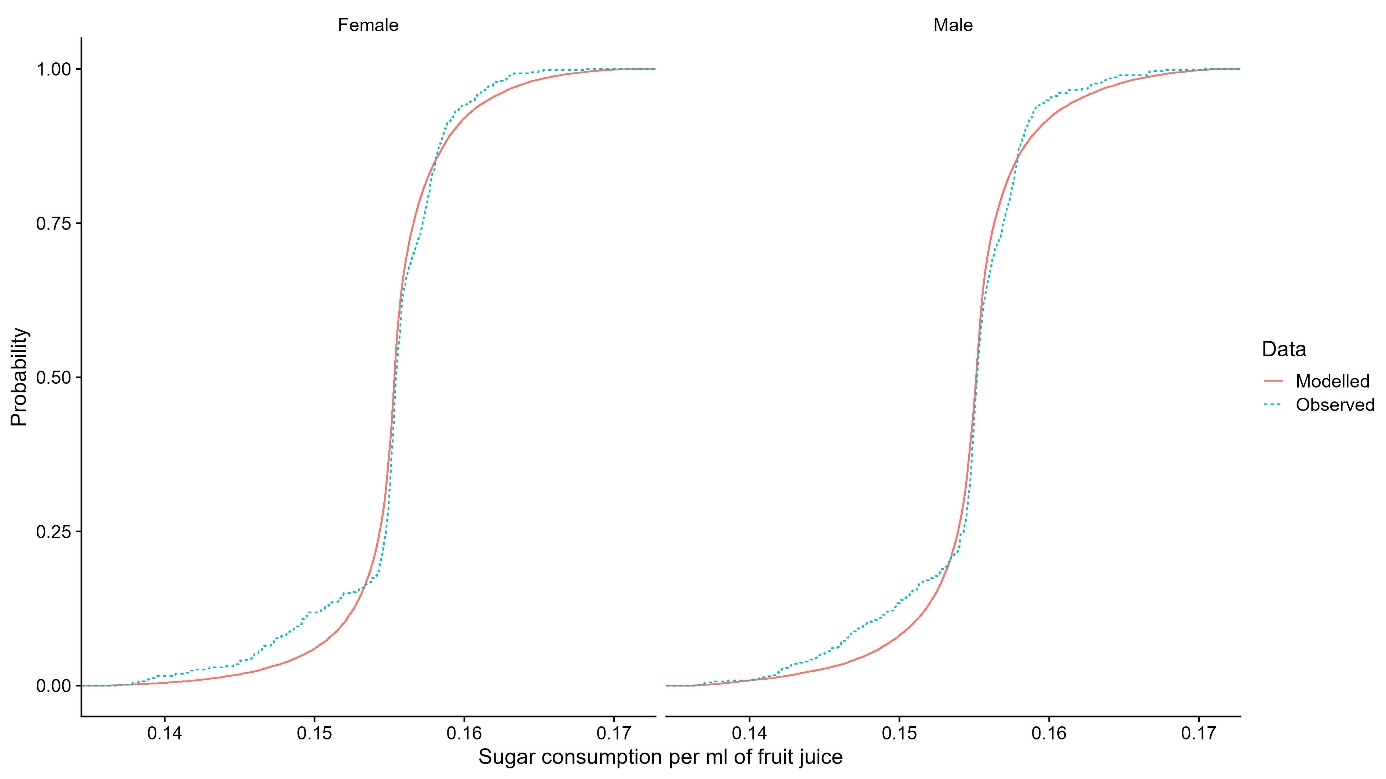


Cumulative distribution plot of the observed (blue dashed lines) and modelled (red lines) distribution of sugar consumption per ml of fruit juice by sex based on the Generlized Additive Models of Location, Shape and Scale (GAMLSS) model. Abbreviations: ml, millilitre.

#### Simulation of exposures for synthetic individuals

The approach described above provides us with equations to estimate the distribution of exposure to a risk factor for a given time and the sociodemographic characteristics of a synthetic individual. When the synthetic individual enters the simulation, a vector of random numbers between 0 and 1 and of size equal to the number of the modelled exposures is allocated to her. Each one of them represents the percentile of the relevant exposure distribution. The principle is that synthetic individuals retain their percentiles throughout their life course (this is known as the rank stability assumption) [53]. For example, in 2014, a 30-year-old female synthetic individual with a BMI of 21.9 kg/m² has a BMI percentile of 0.5. Twenty years later, the same synthetic individual has retained her percentile score for BMI. However, her BMI is now estimated to 24.5 kg/m² because the BMI distribution has changed to reflect the BMI of 50-year-old women in 2034 (**Figure O**). In IMPACT_NCD_, we allow the percentiles of the synthetic individuals to fluctuate every year using random walks, to relax the rank stability assumption.

Finally, exposures in individuals are correlated. For example, people with a high BMI may also have a high consumption of SSBs and/or fruit juice. To accommodate this, we model the linear correlation structure in KORA FF4 using the following approach:

1. We used the quantile function of the distribution estimated by the exposure models, to convert exposures in KORA FF4 to percentiles. Because the distributions were conditional on the independent variables used in each model, the percentiles are adjusted for these variables (i.e., age and sex).

2. We estimated the linear correlation matrix of the percentiles of the exposures of interest in KORA FF4 using Pearson’s correlation.

3. We used the linear correlation matrix from #2 to generate streams of uniforms between 0 and 1 that had a correlation structure similar to the one observed in KORA FF4 [54, 55].

4. We used the correlated streams of random numbers from #3 as the exposure percentiles for the synthetic individuals.

For simplicity, we assumed that the correlation structure of the exposure percentiles remains constant over time.

Figure O: Plot of the percentile against the BMI (cumulative distribution) of female synthetic individuals for ages 30 and 50 years


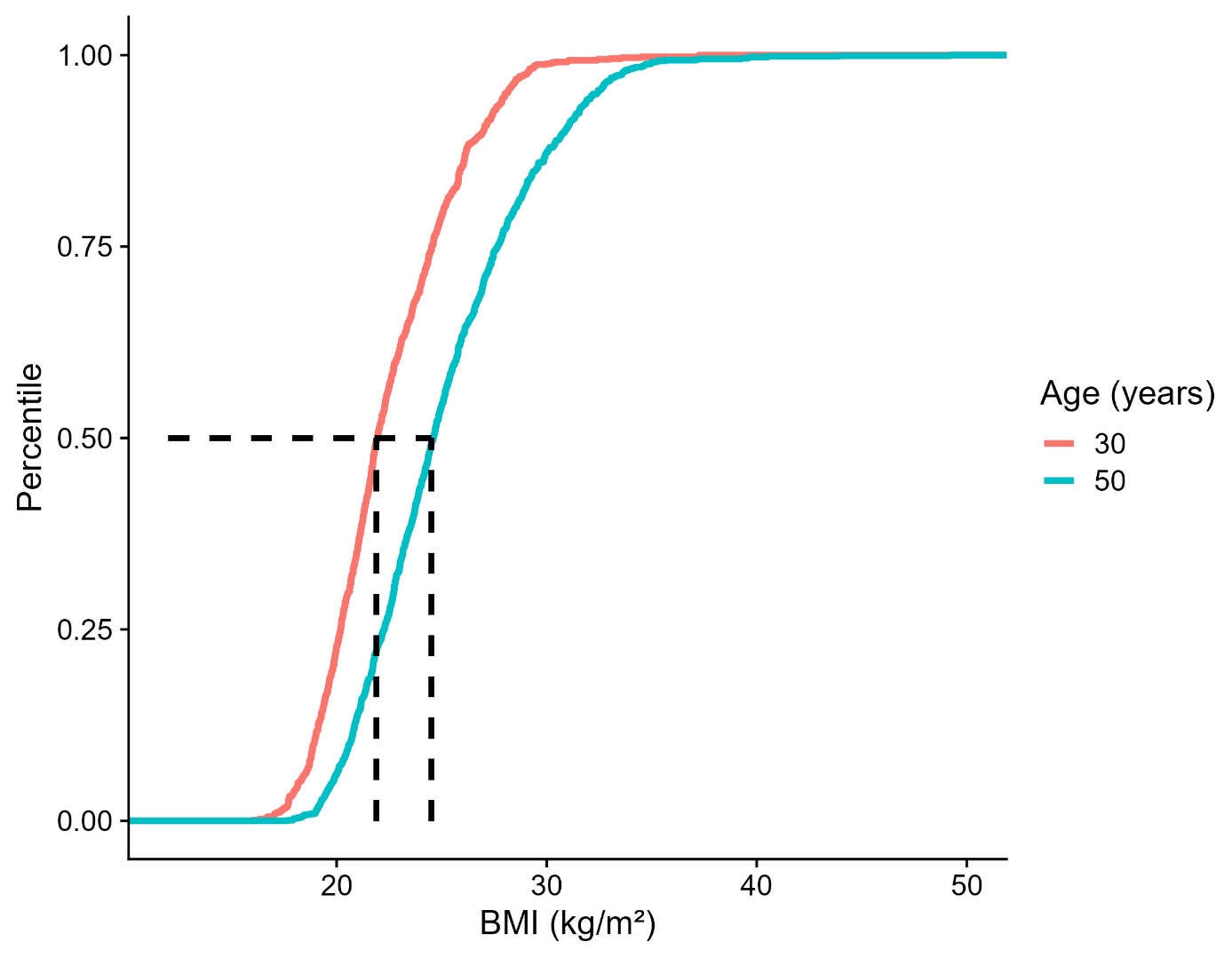


Black dashed line indicates 50^th^ percentile (median) on y-axis and corresponding value of the cumulative BMI distribution on the x-axis. Abbreviations: BMI, body mass index, kg, kilograms; m², square metre.

### Disease module

The previous two modules for demographics and exposure generate a dynamic close-to-reality synthetic population that is composed of the adult life course exposures of each of the synthetic individuals. The disease module then translates these exposures to disease incidence, using a population-attributable risk approach [56]. We will first describe how the disease incidence is simulated in the model, and then how the model simulates mortality.

We have modelled diseases based on the currently accepted causal associations with their risk factors. The included risk factors and the modelled relationships between risk factors and disease incidence are those where sufficient, good quality data on relative risks were available, and where there is sufficient evidence of a causal relationship between a risk factor and incidence of disease. Relative risks were obtained from published systematic reviews, meta-analyses, randomized controlled trials or cohort pooling projects [8, 21, 22, 24-28]. For some parameters, we have additionally relied on previous publications which used microsimulation to answer similar questions in different contexts such as a recently published evaluation of SSB taxation in the US [8, 57].

#### Disease incidence

To estimate the individualised annual probability of a synthetic individual developing a specific disease conditional on their cumulative risk exposures, we follow a 3-step approach:

*Step 1:*

The proportion of incidence attributable to each modelled risk factor by age and sex is estimated, assuming a specific time lag between exposure and disease. The relationships between exposures and disease incidence included in the model are outlined in **Table F** and **Figure P**. An overview of the implemented relative risks is given in **Table G**. The time lags in the model vary stochastically between 1 and 10 years following a shifted binomial distribution. We set the mean lag time for each pair of risk exposure and disease combination according to the best possible empirical data based on the observation period of cohort studies and time to risk reversal in randomised clinical trials (**Table H**).

*Step 2:*

The portion of the disease incidence attributable to all the modelled risk factors is estimated and subtracted from the total incidence for 2013, assuming multiplicative risks.

*Step 3:*

The probability of developing the disease is estimated for each individual in the synthetic population and is used in an independent Bernoulli trial to select those who finally develop the disease.

The implementation of the above method is described in more detail using CHD as an example.

Table F: Overview of disease modelling

| Modelled condition | Recovery & recurrence | Causal relationship with risk factors |
| --- | --- | --- |
| Stroke | No recovery | Yes |
| Type 2 Diabetes | No recovery | Yes |
| Coronary heart disease | No recovery | Yes |

Table G: Relative risks and etiologic effects of exposures on cardiometabolic risk

| Risk factor/exposure | Outcome | Source | Unit | Risk estimate by age group (95%-confidence intervals)* | | | | | |
| --- | --- | --- | --- | --- | --- | --- | --- | --- | --- |
|  |  |  |  | 25-34 years | 35-44 years | 45-54 years | 55-64 years | 65-74 years | 75+ years |
| Sugar from SSBs or fruit juice | BMI (baseline BMI <25 kg/m²) | Micha et al., 2017 [21];  Huang et al., 2019 [8] | kg/m² per gram of sugar^#‡^ | 0.005  (0.0025; 0.0075) | | | | | |
|  | BMI (baseline BMI >25 kg/m²) |  |  | 0.0115  (0.007; 0.016) | | | | | |
| SSBs  (adjusted for BMI) | CHD | Xi et al., 2015 [22];  Huang et al., 2019 [8] | Per 227ml/day^#†^ | 1.33  (1.19; 1.47) | 1.31  (1.18; 1.45) | 1.26  (1.15; 1.37) | 1.21  (1.13; 1.30) | 1.17  (1.10; 1.24) | 1.09  (1.06; 1.13) |
| SSBs  (adjusted for BMI) | Type 2 diabetes | Imamura et al., 2015 [24];  Huang et al., 2019 [8] | Per 227ml/day^#†^ | 1.35  (1.14; 1.59) | 1.33  (1.13; 1.56) | 1.27  (1.11; 1.46) | 1.22  (1.09; 1.36) | 1.18  1.07; 1.29) | 1.10  (1.05; 1.15) |
| BMI  (adjusted for diabetes) | CHD | Lu et al., 2014 [25];  Huang et al., 2019 [8] | Per 5 kg/m² BMI increase | 1.45  (1.36; 1.53) | 1.42  (1.35; 1.51) | 1.35  (1.29; 1.41) | 1.28  (1.23; 1.33) | 1.23  (1.19; 1.27) | 1.13  (1.11; 1.14) |
| BMI  (adjusted for diabetes) | Stroke | Lu et al., 2014 [25]; Huang et al., 2019 [8] | Per 5 kg/m² BMI increase | 1.24  (1.16; 1.33) | 1.23  (1.15; 1.32) | 1.19  (1.13; 1.26) | 1.16  (1.10; 1.21) | 1.13  (1.09; 1.17) | 1.07  (1.05; 1.09) |
| BMI | Type 2 diabetes | Singh et al., 2013 [26]; Huang et al., 2019 [8] | Per 5 kg/m² BMI increase | 3.55  (2.41; 5.23) | 3.07  (2.28; 4.15) | 2.66  (2.15; 3.30) | 2.32  (2.04; 2.63) | 2.03  (1.95; 2.11) | 1.52  (1.40; 1.65) |
|  |  |  |  | **25-59 years** | | **60-69 years** | | **70+ years** | |
| Type 2 diabetes | CHD | Sarwar et al., 2010 [27] | n/a | 2.51  (2.25; 2.80) | | 2.01  (1.80; 2.26) | | 1.78  (1.54; 2.05) | |
| Type 2 diabetes | Stroke | Sarwar et al., 2010 [27] | n/a | 3.74  (3.06; 4.58) | | 2.06  (1.64; 2.58) | | 1.80  (1.42; 2.27) | |
|  |  |  |  | **30+ years** | | | | | |
| Type 2 diabetes | Non-CVD mortality | Stringhini et al., 2017 [28] | n/a | 1.87  (1.72; 2.03) | | | | | |

*Age patterns were incorporated based on the study by Huang et al., [30], which applied a method to account for the proportional decline of effects with age [26]. ^#^The used effect estimate assumes 20g sugar per 8 fluid ounces of sugar-sweetened beverages (≈ 227ml). ^‡^These effect estimates are more conservative than traditional weight change equations such as those developed by [58], [59] or [60]. Assuming a reduction of 20g of sugar, which equates to about 77 kilocalories (≈322 kilojoule), for a male individual of age 35 years, 175cm height, 85kg weight (= body mass index of 27.76 kg/m²), and average phyiscal activity level of 1.5 (see [58] for details) our method predicts a long-term BMI reduction of 20*0.0115 = 0.23kg/m² (= 0.70kg). Additionally assuming 2500kcal of daily energy intake, the equations from [58] predict a long-term BMI reduction of 1.25kg/m². Assuming the same values, the formula from [59] predicts a long-term reduction BMI of 0.62 kg/m². Using the same values, the dynamic model from [60] predicts a long-term BMI reduction of 0.70kg/m² in the first year alone (https://www.niddk.nih.gov/bwp). ^†^We excluded these direct BMI-independent effects of SSBs in robustness analyses. Abbreviations: BMI, body mass index; CHD, coronary heart disease; CVD, cardiovascular disease; m², square metre; n/a, not applicable; SSBs, sugar-sweetened beverages.

Figure P: Implemented structure of IMPACT_NCD_ Germany


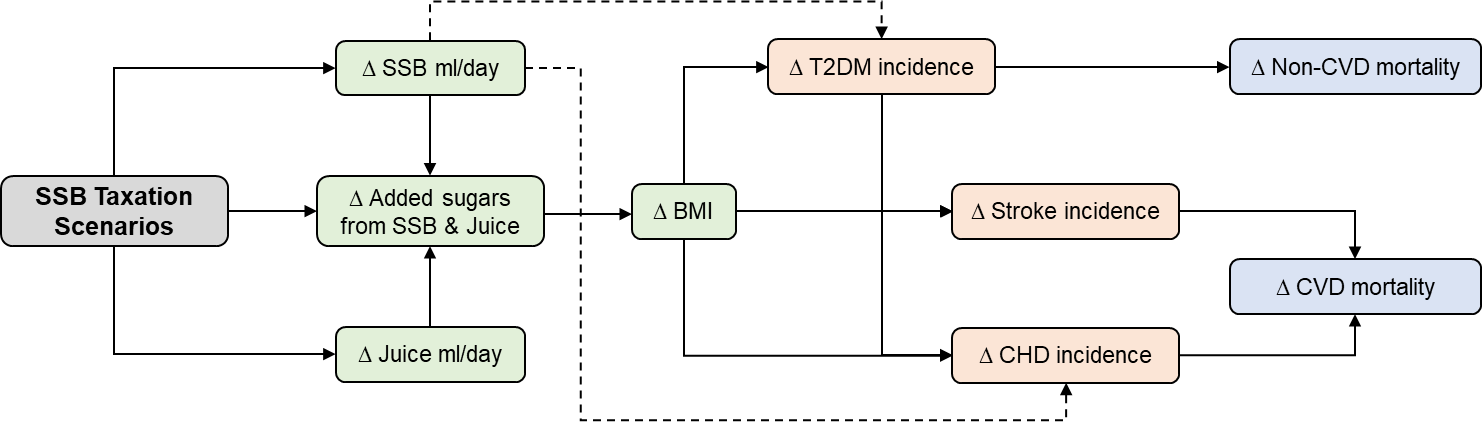


Overview of the causal model structure implemented in this study. Green boxes indicate exposures/risk factors, red boxes indicate disease outcomes, and blue boxes indicate mortality outcomes. Dashed lines represent BMI-independent health effects of SSBs which are excluded in robustness analyses. Abbreviations: Δ, “change in”; BMI, body mass index; CHD, coronary heart disease; CVD, cardiovascular disease; SSBs, sugar-sweetened beverages; T2DM, type 2 diabetes.

Table H: Causal relationships included in the model and the time lag assumed between exposure and outcome

|  | Coronary Heart Disease | Stroke | Type 2 Diabetes | Mortality from non-modelled diseases |
| --- | --- | --- | --- | --- |
| BMI | 4 years | 4 years | 5 years | - |
| SSB consumption | 4 years | - | 4 years | - |
| Type 2 Diabetes | 4 years | 4 years | - | 5 years |

The proportion of incidence attributable to each modelled risk factor by age and sex is estimated, assuming a specific time lag (in years) between exposure and disease. Abbreviations: BMI, body mass index; SSBs, sugar-sweetened beverages.

##### Example procedure based on coronary heart disease (CHD)

*Step 1:*

The Population Attributable Fraction (PAF) is an epidemiological measure that estimates the proportion of the disease attributable to an associated risk factor. It depends on the relative risk associated with the risk factor and the prevalence of the risk factor in the population. In a microsimulation context where exposures to risk factors are known at the individual level and assuming multiplicative risk factors, the PAF (i.e., the individualized attributable fraction) can be estimated using the formula:

$$PAF= 1-\frac{n}{\sum_{i=1}^{n} (RR_{i1}* RR_{i2}*\ldots* RR_{ik})}$$

where $n$ is the number of synthetic individuals in the population, and ${RR}_{i1\ldots ik}$ are the relative risks (RR) of the risk factors associated with CHD, for each individual $i$. We calculated the PAF based on the above formula stratified by age and sex. Consistent with findings from the respective meta-analyses that were used for IMPACT_NCD_ a BMI below 22 kg/m^2^ and SSB consumption of 0 ml/day were considered to have a relative risk of 1 **Table H**.

*Step 2:*

The incidence of CHD not attributable to the modelled risk factors can be estimated by the formula:

$$I_{Non-attributable}= I_{Observed}*\left( 1-PAF \right)$$

Where $I_{Observed}$ is the CHD incidence and $PAF$ is from Step 1. $I_{Non-attributable}$ represents CHD incidence if all the modelled risk factors were at optimal levels but not its theoretical minimum because we do not model all CHD risk factors in this study.

The non-attributable and observed incidences are calculated only in the initial year of the simulation (see below). To account for time trends in CHD incidence not attributable to the modelled risk factors, the model updates $I_{Observed}$ every simulated year. For this we assume that half of the observed or forecasted annual change in CHD mortality (see section Mortality) is attributed to changes in CHD incidence and the other half to changes in CHD case fatality. This assumption is based on evidence from England, and modelling studies in England and the US [61-64]. To further account for potential improvements in non-modelled risk factors, the non-attributable incidence is assumed to decrease by 3% per year in the years after the simulation is initiated to account for improvements in non-modelled risk factors such as systolic blood pressure in the case of CHD.

*Step 3:*

Assuming that $I_{non-attributable}$ is the annual baseline probability of a synthetic individual to develop CHD for a given age and sex due to risk factors not included in the model (i.e. genetics, air pollution, systolic blood pressure other dietary exposures etc.), the individualised annual probability of developing CHD, $\mathbb{P}\left( \text{CHD | age, sex,}\text{ }\text{exposures} \right)$, given his/her risk factors were estimated by the formula:

$$\mathbb{P}\left( CHD | age,sex, exposures \right)= I_{non-attributable}*RR_{i1}*RR_{i2}*RR_{i3}*\ldots*RR_{ik}$$

Where $RR_{i1 \ldots ik}$ are the relative risks that are related to the specific risk exposures of the synthetic individual, same as in step 1.

##### Estimating the observed incidence probability *I_Observed_* for type 2 diabetes

To estimate the observed incidence probability $I_{Observed}$ for T2DM, we use recently published data from the German national diabetes surveillance program which provides information on diabetes incidence by age and sex based on all 70 million persons with statutory health insurance in Germany for 2011 [11]. More recent data is unfortunately not available. These incidence estimates include type 1 diabetes however, the authors state that only 0.28%-points of the total diabetes prevalence of 9.68% are due to type 1 diabetes. We therefore deem these estimates appropriate for our analysis as the expected error due to type 1 diabetes is negligible.

To calculate the incidence probability by single years of age, we disaggregate and smooth the original incidence data by estimating a GAMLSS model with a Generalized Beta 1 distribution for men and women separately. Here the incidence probability is the independent variable and age the only dependent variable. The age effect is modelled with a penalized beta spline. To improve fitting, we inject small amounts of normally distributed random noise around the point estimate of the original data.

Finally, to account for biases in the data, we injected uncertainty of ± 2% (relative) to the estimated disease incidence probability during the simulation. We deliberately chose a small amount of additional uncertainty here since the T2DM incidence data we use is based on almost the entire German population and we believe it to be of very high quality.

##### Estimating the observed incidence probability *I_Observed_* for CHD and stroke

To estimate the observed incidence probability $I_{Observed}$ for CHD and stroke, we applied the recently published SCORE2 (age 40 to 69 years) and SCORE2-OP (age 70+ years) cardiovascular disease (CVD) risk equations to 9,620 observations from KORA S4, F4 and FF4, as no other national data sources were available [9, 15, 16]. The SCORE2 equations enable the sex-specific prediction of the risk for cardiovascular disease based on sociodemographic and clinical characteristics (i.e., age, smoking status, systolic blood pressure, total cholesterol, and high-density lipoprotein cholesterol). Following the guidance in the supplementary material of the original papers, we used the SCORE2 risk equations to predict the 10-year risk for CVD for all participants in the KORA S4, F4 and FF4 studies which we then transformed into a 1-year probability using standard rate-probability transformations [55]. Finally, we used sex-specific GAMLSS models to calculate the median 1-year probability to develop CVD by age and sex. **Figure Q** shows the results of this process. We used the estimated median by age and sex from FF4 as the observed incidence probability $I_{Observed}$ for CVD in the baseline year of the simulation.

Since we needed the observed incidence probability $I_{Observed}$ separately for CHD and stroke, we adjusted the estimated incidence probability for CVD with the share of fatal and non-fatal CHD events among all CVD events (i.e., CHD and stroke) from the *European Prospective Investigation into Cancer and Nutrition* (EPIC) study [17]. EPIC reported 17,594 cardiovascular events (CHD, *International Statistical Classification of Diseases and Related Health Problems* [ICD] I21-I25; Stroke, ICD I60-69). Among men 6,688 (66.65%) events (fatal and non-fatal) were CHD and 3,346 stroke. Among women 4,318 (57.12%) events (fatal and non-fatal) were CHD and 3,242 stroke. We therefore multiplied the estimated incidence probability for CVD with the share of CHD events among CVD events that EPIC observed in Germany (62.55% for men; 42.45% for women) to calculate the observed incidence probability $I_{Observed}$ for CHD. We used the remaining proportion of CVD events (37.45% for men; 57.55% for women) to calculate the observed incidence probability $I_{Observed}$ for stroke. We therefore assumed that CHD and stroke incidence follow the same functional form over age.

As a last step, we used DISMOD II to improve consistency of the estimated age- and sex-specific incidence probability for CHD and stroke with information on disease-specific prevalence and mortality [19]. DISMOD II is a multi-state life table model that enables the estimation of the incidence, prevalence, mortality, case fatality and remission of a disease. Since these disease epidemiological variables depend on each other, DISMOD II can derive values for the missing variables under the condition that information about at least three of these variables is available. A similar approach was used by the Global Burden of Disease study and others [65, 66]. We here only consider the first ever episode of CHD. For the DISMOD II calculations, we assumed that incidence and case-fatality rates had been declining by 3% (relative), over the last 10 years.

To account for uncertainty in the estimation procedure and biases in the KORA data, we injected uncertainty of ± 10% (relative) to the estimated CHD and stroke incidence probability during the simulation.

Figure Q: Median 1-year cardiovascular disease incidence probability using SCORE2 equations
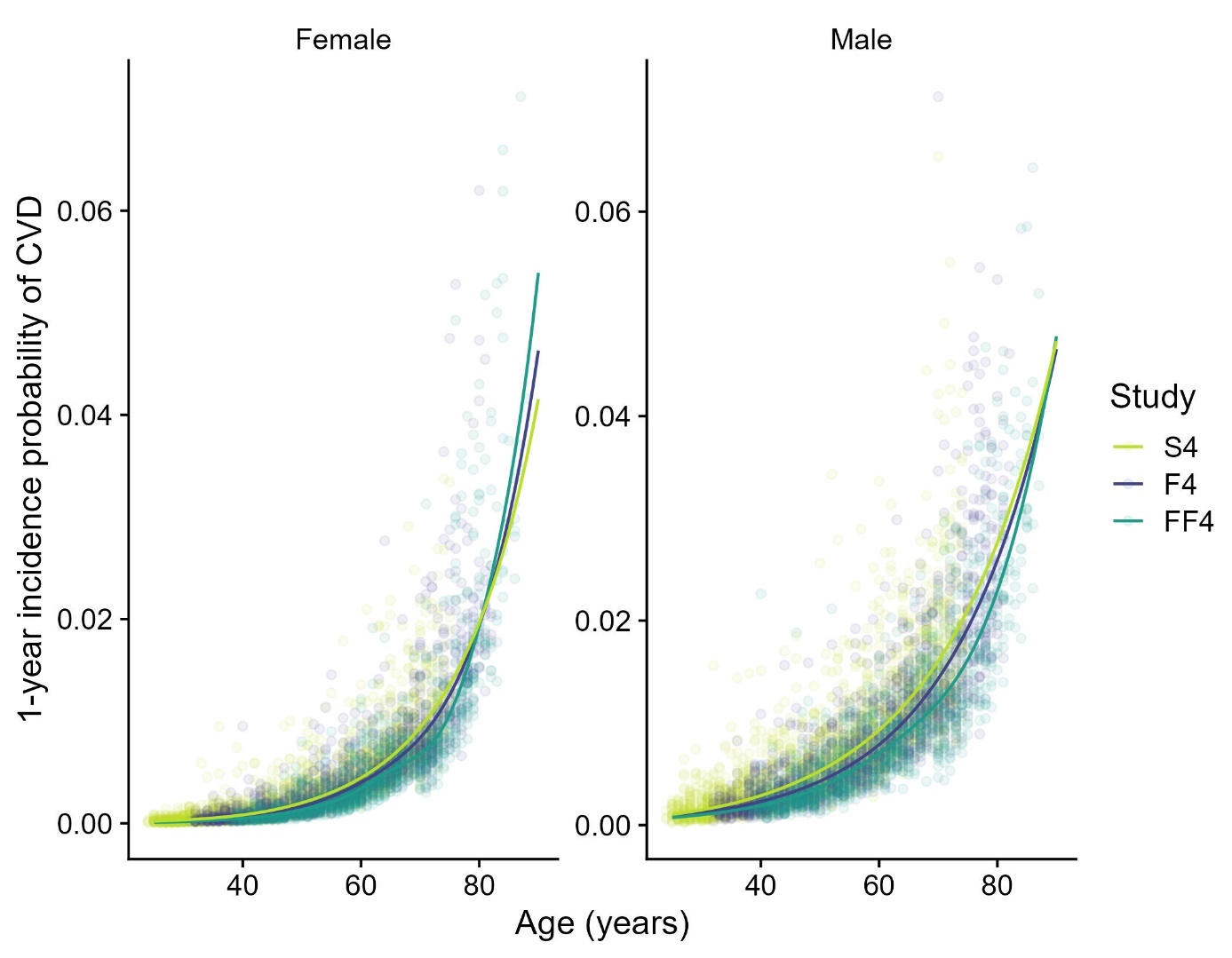


Points indicate estimated 1-year incidence probability of cardiovascular disease (CVD) for each individual in the respective Kooperative Gesundheitsforschung in der Region Augsburg (KORA) survey and follow-ups (S4 [year 1999], F4 [year 2007], FF4 [year 2014]). Lines are the median estimated yearly incidence probability to develop CVD in Germany by sex over age according to the different surveys and follow-ups.

##### Initial prevalence

For the initial simulation year, some synthetic individuals need to be allocated as prevalent cases for each of the modelled diseases. To allocate the initial year disease prevalence in the simulation, we sampled prevalent cases in each age-sex-stratum using a weighted sampling algorithm that takes the disease-specific risk factor profile of synthetic individuals into account. This ensures that individuals with a less favourable risk factor profile are more likely to be a prevalent case in the initial simulation year.

The initial prevalence of T2DM was based on the same recently published study as the incidence data (see above) [11]. We used a similar sex-specific GAMLSS estimation procedure to disaggregate the published prevalence values into single years of age. Again, we injected uncertainty of ± 2% (relative) to the estimated prevalence during the simulation.

No data on the age- and sex-specific prevalence of CHD and stroke in Germany was available. We therefore used age- and sex-specific data on the prevalence of these diseases from England which was supplied by collaborators as a starting point to estimate the initial prevalence with DISMOD II (see above for details about DISMOD II) [18]. To account for heterogeneity in data quality, DISMOD II allows the user to specify weights for each of the three needed disease parameters in the procedure. We therefore applied the minimal possible weight to the CHD and stroke prevalence to let the algorithm estimate plausible prevalence values based on the available CHD incidence and mortality data. The external validation of the modelled CHD and stroke prevalence with available national survey data shows that this procedure was successful (see section Validation and calibration). Still, to account for the high uncertainty in the estimated prevalence of CHD and stroke, we injected uncertainty of ± 10% (relative) to the estimated prevalence during the simulation.

**Dependencies between conditions**

We modelled dependencies between conditions with strong correlations based on our epidemiological understanding. In the context of this study, we modelled T2DM as a risk factor for CHD and stroke (**Table G**, **Figure** **P**).

#### Disease duration

For existing prevalent conditions among simulants at the start of the simulation (2013), the number of years lived with each condition can be assigned based on sociodemographic characteristics. However, since we do not apply different health or productivity cost values based on prevalent disease durations, we assumed a Poisson distribution with λ = 5 for all prevalent cases for all diseases. This is necessary for the model to run as intended but has no impact on the results. In future iterations of IMPACT_NCD_ Germany disease duration can be based on more granular information.

#### Mortality

All synthetic individuals are exposed to the risk of dying from any of their acquired modelled conditions (i.e., CHD and stroke) or any other non-modelled (i.e., non-CVD) cause. We treat the latter as a condition that everyone is a prevalent case of. This allowed us to treat non-CVD mortality like any other condition in the model.

Data on the national mortality from CHD and stroke were retrieved from the German Information System of the Federal Health Monitoring (*Gesundheitsberichterstattung des Bundes*, www.gbe-bund.de) from 1991 to 2019 by sex and five-year age groups [12]. We used data as reported by the European Shortlist for Causes of Death (ESCD) which harmonizes death counts before 1997 (ICD-9, nineth revision) and after 1998 (ICD-10, tenth revision). CHD was defined with the ESCD category for ‘ischemic heart diseases’, corresponding to the ICD-10 categories I20-I25 and ICD-9 categories 410-414, thus also including acute myocardial infarctions. Stroke was defined using the ESCD ‘cerebrovascular diseases’ category, corresponding to the ICD-10 categories I60-I69 and ICD-9 categories 430-438. Corresponding yearly mortality rates were calculated by dividing the age- and sex-specific number of deaths per disease by the respective population size retrieved from the German Federal Statistical Office (GENESIS, [www.destatis.de](http://www.destatis.de)) [13]. Mortality rates were smoothed and disaggregated to single years of age with the *R* package “*demography*” [20].

For CHD and stroke, we used DISMOD II to estimate the disease-specific case fatality (the probability of a prevalent case of a condition dying of this condition) in 2013, conditional on age and sex. This ensures that the used prevalence, incidence, and mortality data are consistent. However, we were not able to decompose case fatality to the first-year post-diagnosis case fatality and the second year onwards post-diagnosis case fatality. For all potentially fatal conditions (i.e., CHD, stroke, and non-CVD), we applied the same PAF approach, described above for incidence, to the case fatality. Therefore, we allow exposures to risk factors to influence the mortality probability of the simulants.

##### Mortality calibration

To calibrate the disease-specific mortality projected by IMPACT_NCD_ Germany, we fitted functional demographic models by sex to the official mortality rate estimates by single year of age from 1991 to 2019 and projected CHD, stroke, and non-modelled mortality rates beyond the simulation horizon (2050) using the *R* package “*demography”* [20]. Functional demographic models are generalisations of the Lee-Carter demographic model, influenced by ideas from functional data analysis and non-parametric smoothing [20]. We then adjusted the case fatality rates of CHD and stroke diseases in the simulation by an age- and sex-specific calibration factor, to track the observed (2013-2019) and projected (2020-2050) mortality rates of the functional demographic model until the simulation horizon (2043). For further details see section Validation and calibration.

### Policy module

The policy module of IMPACT_NCD_ Germany allows the user to specify detailed policy scenarios in a highly flexible way. Scenarios can depend on all modelled sociodemographic characteristics, exposures and the respective simulation year. To calculate the effectiveness of policies, outcomes for each scenario can be calculated (see section Model outcomes) and compared with a counterfactual baseline scenario without any policy or intervention.

#### Implementation of SSB taxation scenarios

In this study we implemented three main policy scenarios, each representing the potential effect of the introduction of differently designed taxes on sugar-sweetened beverages in Germany on a national level. All modelled scenarios were based on real-world implemented tax designs such as the volumetric tax on SSBs in Mexico, the ad-valorem tax in Chile or the tiered tax in the United Kingdom (UK) [67].

Due to data limitations, we are not able to model each tax design directly in detail. For example, to model a tiered tax with specific sugar thresholds and respective tax rates which incentivizes reformulation through producers, researchers need access to product-level purchasing data including the exact sugar content of the beverages bought per individual or household. Unfortunately, we did not have access to such data during this project. The situation is similar for volumetric taxes, which also depend on the characteristics of the specific products (i.e., their size).

However, our model was built to approximate the chosen scenarios and their overall effect on SSB and sugar consumption. To implement reformulation effects, we leverage individual information on the sugar consumed from SSBs (as described above under Exposure module). To approximate volumetric taxes, we use the fact that most of the implemented taxes (volumetric or *ad-valorem*) were designed to increase SSB prices on average by 10-20% [68]. An *ad-valorem* tax of 20% (excluding pass-through) thus approximates similarly designed volumetric taxes. An overview of the modelled scenarios and details regarding their implementation is given in **Table I**.

In all scenarios in which beverage consumption is changed, we assume that the policy immediately affects consumption. We assume that the consequent impact on BMI through reduced intake of sugar is coming into effect over the course of the next three years in the simulation. We include this parameter in the probabilistic sensitivity analysis (see below) and vary it between 1 and 5 years, following a uniform distribution. The proportion of the tax that is passed on to consumers (tax pass-through) in scenarios 1, 2 and 4 was assumed to be 82% (95%-confidence interval: 66%; 98%) based on a recent meta-analysis [29].

Table I: Implemented SSB taxation policy scenarios and calculation details

| Scenario | Affected exposures | Policy lag | Calculation details |
| --- | --- | --- | --- |
| 1) 20% *ad-valorem* tax on SSBs (“*ad-valorem* tax”) | SSB intake ↓;  fruit juice intake ↑ (substitution) | 0 years | 1. Change in consumption of SSBs after tax based on *own-price* elasticity. 2. Change in consumption of fruit juice after tax based on *cross-price* elasticity. 3. Change in sugar consumed from SSBs and fruit juice (multiplication of new consumption in ml/day from 1. and 2. with individual amount of sugar per beverage in g/ml). 4. Change in BMI due to change in sugar consumption from beverages (stratified by BMI > 25 m²/kg and BMI < 25 m²/kg). |
| 2) 20% *ad-valorem* tax on SSBs & fruit juice (“extended *ad-valorem* tax”) | SSB intake ↓;  fruit juice intake ↓ | 0 years | 1. Change in consumption of SSBs and fruit juice after tax based on *own-price* elasticities. 2. Change in sugar consumed from SSBs and fruit juice (multiplication of new consumption in ml/day from 1. and 2. with individual amount of sugar per beverage in g/ml). 3. Change in BMI due to change in sugar consumption from beverages (stratified by BMI > 25 m²/kg and BMI < 25 m²/kg). |
| 3) 30% reformulation and stable consumption (“tiered tax”) | Sugar from SSBs ↓ | 3 years | 1. Change in individual amount of sugar per ml of SSB due to reformulation (30% over three years). 2. Change in sugar consumed from SSBs (multiplication of new individual amount of sugar per beverage in g/ml with (unchanged) consumption in ml/day). 3. Estimation of the direct (BMI-independent) effect of SSBs in ml/day by re-translating the reduction in sugar (g/day) into an equivalent effect in ml/day (division of reduction in sugar in g/day by the original individual amount of sugar per beverage in g/ml; g/day / g/ml = ml/day). This is mathematically equivalent to implementing the (BMI-independent) effects of SSBs directly via sugar in the model. 4. Change in BMI due to change in sugar consumption from beverages (stratified by BMI > 25 m²/kg and BMI < 25 m²/kg). |

Abbreviations: BMI, body mass index; g, grams; ml, mililitre; SSBs, sugar-sweetened beverages.

#### Price elasticities of demand for beverages in Germany

To be able to estimate the change in beverage consumption under the *ad-valorem* tax scenarios, we implemented price elasticities for different beverage categories in Germany. Because no national price elasticities were available, these were newly estimated by an experienced economist as part of the author team for this project. The general methodological approach was based on a previous study on price elasticities of meat in Germany [69]. All data and methods used to estimate the beverage price elasticities used in this study are described in detail in the following paragraphs.

##### Data and methods

To estimate beverage price elasticities, we used the commonly applied Almost Ideal Demand System (AIDS) and data from two consecutive waves (2013 & 2018) of the German national household expenditure survey (EVS) which is an official survey of living conditions of households in Germany (*Einkommens- und Verbraucherstichprobe*; <https://www.forschungsdatenzentrum.de/de/haushalte/evs>).

The EVS is population representative and takes place every five years collecting data from 60,000 German households. Data on the consumption of food and beverages is collected in a subsample by the Federal Office of Statistics using detailed expenditure lists over one month by prices and quantity. Hereby the month of data collection is rotated over households to account for seasonality effects. In 2013 and 2018 a subsample of 11,648 and 10,562 households provided records on expenditures for foods and beverages. After removing outliers and households without beverage consumption, our analysis sample consisted of 21,636 households. For these households, we selected the six non-alcoholic beverage categories plain milk, flavoured milk, “coffee & tea”, waters, SSBs, and fruit juices. Household expenditures were adjusted using the Organization for Economic Co-Operation and Development equivalence scale (1 for the first adult, 0.5 for a child age ≤ 15 years and 0.3 for any other child and additional adult). To adjust for seasonality, we obtained temperature data from the German Weather Service (<https://opendata.dwd.de/climate_environment/CDC/regional_averages_DE/>), which was aggregated to Nielsen areas (see below).

##### Demand model

We estimated a linearly approximated AIDS system according to Deaton and Muellbauer (1980) adjusted for censoring in the dependent variable [70]. We follow the procedures as in Roosen et al. (2022) [69]. Demand for the six beverage categories is modelled as expenditure shares to introduce constraints on adding up, homogeneity, and symmetry.

The expenditure share of household *h* on category *i*, $w_{ih}$, is denoted as

$w_{ih}=\alpha_{i}+\sum_{j} \gamma_{ij}lnp_{jh}+\beta_{i}ln\left( {M_{h}}/{P_{h}} \right)$ for $i=1, \ldots, 6$ (1)

In equation (1), $p_{ih}$ denotes the per-unit price of product category *i* and $M_{h}$ is the equivalent household net income of household *j*. As for the linear AIDS we used a linear approximation of the price index $M_{h}$ following Moschini (1995) [71].

We impose the theoretical properties of demand by:

Adding up: $\sum_{i} \alpha_{i}=1$, $\sum_{i} \beta_{i}=0$, $\sum_{i} \gamma_{ij}=0$ (2a)

Homogeneity: $\sum_{j} \gamma_{ij}=0$ (2b)

Symmetry: $\gamma_{ij}=\gamma_{ji}$ (2c)

##### Statistical procedures

The resulting system of equations was estimated using the seemingly unrelated regression approach. We included binary indicators for season and region. For regions, we used Nielsen regions that correspond to the German federal states with some exceptions:

1. Hamburg, Bremen, Lower Saxony, and Schleswig-Holstein were summarized into one region.
2. Hesse, Rhineland Palatinate, and Saarland were summarized into one region.
3. Berlin, Brandenburg, Mecklenburg-Western Pomerania and Saxony-Anhalt were summarized into one region.
4. Saxony and Thuringia were summarized into one region.

Prices were quality-adjusted using methods developed by Cox and Wohlgenant (1986), by regressing unit values on household size, monthly household net income, social status of the household head, the age of the main earner, the number of income earners in the household, whether the main earner is male, the number of children in the age categories below 1 year, between 1 and 3 years, between 3 and six years, between 6 and 12 years and between 12 and 18 years. To assure adjusted unit values the adjustment procedures was based on log prices [72].

To allow for censoring in each individual product category, we used the two-step approach developed by Shonkwiler and Yen (1999) [73]. We estimated probit models of positive expenditure in a product category. Conditional on non-zero expenditures, equation (1) is corrected by the predicted probability density function $\phi_{ih}$ and cumulative distribution function $\Phi_{ih}$ to yield:

$w_{ih}= \Phi_{ih}\times\left[ \alpha_{i}+\sum_{j} \gamma_{ij}\ln p_{jh}+\beta_{i}\ln\left( M_{h}/P_{h} \right) \right]+\theta_{i}\phi_{ih}+\varepsilon_{ih}$ (3)

The expenditure share equations are estimated for five product categories. The product category “coffee & tea” was left out to avoid singularity. The corresponding parameters can be obtained via the constraints (2a) – (2c).

The probit model was based on the same socio-demographic and economic variables mentioned above and additionally adjusted for region and month. Based on the above, we estimate the uncompensated own- and cross-price elasticities for all six beverage categories using the formulas from Green and Alston (1990) [74].

Uncompensated price-elasticities: $\varepsilon_{ii}=\bar{\Phi}_{i}\cdot\left( \frac{\hat{\gamma}_{ii}}{\bar{w}_{i}}-\hat{\beta}_{i} \right)-1$ (4b)

$\varepsilon_{ij}=\bar{\Phi}_{i}\cdot\left( \frac{\hat{\gamma}_{ij}-\hat{\beta}_{i}\bar{w}_{j}}{\bar{w}_{i}} \right)$, with $k\neq j$ (4c)

The resulting uncompensated own- and cross-price elasticities of demand for the six modelled beverage categories are presented in **Table J**. In our model we only apply the own-price elasticities of SSBs and fruit juice and the cross-price elasticity of a change in SSBs on the demand for fruit juice. Generally, our newly estimated values are in line with other published beverage price elasticities [29].

Table J: Uncompensated price elasticities of demand for beverages in Germany

| Beverage category | Price elasticities (standard error) | | | | | |
| --- | --- | --- | --- | --- | --- | --- |
|  | *Change in price by 1%* | | | | | |
|  | Flavoured milk | Pure milk | Water | SSBs | Fruit juice | Coffee & tea |
| *Change in demand* |  |  |  |  |  |  |
| Flavoured milk | **-1.055** (0.151) | -0.036 (0.14) | -0.077 (0.15) | 0.087 (0.164) | 0.203 (0.164) | -0.073 (0.087) |
| Pure milk | -0.018 (0.06) | **-0.788** (0.169) | -0.082 (0.093) | 0.038 (0.097) | -0.063 (0.116) | 0.006 (0.079) |
| Water | -0.029 (0.051) | -0.067 (0.074) | **-0.801** (0.099) | -0.036 (0.078) | 0.080 (0.081) | -0.072 (0.053) |
| SSBs | 0.026 (0.055) | 0.012 (0.076) | -0.055 (0.077) | **-0.956** (0.111) | 0.047 (0.082) | -0.104 (0.047) |
| Fruit juice | 0.077 (0.065) | -0.081 (0.107) | 0.068 (0.093) | 0.052 (0.097) | **-1.106** (0.149) | -0.063 (0.055) |
| Coffee & tea | -0.024 (0.027) | -0.024 (0.05) | -0.088 (0.044) | -0.089 (0.041) | -0.044 (0.040) | **-0.801** (0.064) |

Uncompensated own- and cross-price elasticities of the six modelled beverage categories estimated by the linearly approximated Almost Ideal Demand System. Own-price elasticities are on the diagonal in bold format. Cross-price elasticities indicate the change in demand of category *i* (row) based on a change in price of category *j* (column) by 1%. For example, the change in demand of water if the price of juice changes by 1% is in column five (juice) and row three (water), equal to 0.080*1% = 0.080%. Abbreviations: SSBs, sugar-sweetened beverages.

#### Sensitivity analyses

We have implemented multiple sensitivity analyses to check the robustness of our results and provide more context to our main SSB taxation scenarios.

As specified in the main text, we modelled: 1) impacts of observed voluntary reformulation of SSBs by industry (i.e., 2% per 6 years) derived from a recent analysis in Germany [32]; 2) a tiered tax with reformulation by 10%; 3) ad-valorem tax rates of 10% and 30%; 4) Scenario 1 without substitution effects to fruit juice (i.e., setting the cross-price elasticity to 0); 5) the impact of price changes on SSB consumption with a meta-analytic estimate; 6) a maximum impact scenario which combines reformulation and consumption reduction; and 7) varied discount rates for costs and quality adjusted life-years (QALYs) (0%, 5% and 10%). An overview of all sensitivity analyses and calculation details is given in **Table K**.

Table K: Implemented sensitivity analyses and calculation details

| Sensitivity analysis | Based on main scenario… | Calculation details |
| --- | --- | --- |
| 1) Observed voluntary industry reformulation | Tiered tax | Same calculation as in the tiered tax scenario but with 2% reformulation every 6 years. This is equivalent to an annual reduction of SSB sugar content by 0.33%. |
| 2) Less reformulation | Tiered tax | Same calculation as in the tiered tax scenario but with 10% reformulation. |
| 3) 10% / 30% *ad-valorem* tax on SSBs | *Ad-valorem* tax | Same calculation as in the *ad-valorem* tax scenario but with 10% / 30% tax rate. |
| 4) 20% *ad-valorem* tax on SSBs without substitution | *Ad-valorem* tax | Same calculation as in the *ad-valorem* tax scenario but without substitution to fruit juice (step 2 is skipped). |
| 5) Meta-analytic estimate of price changes and SSB consumption | *Ad-valorem* tax | Same calculation as in the *ad-valorem* tax scenario but with an estimate of the own-price elasticity of SSBs (‑0.674; standard error: 0.187) which was extracted from a recent meta-analysis. This estimate was used by other evaluations of SSB taxation scenarios [57, 75]. |
| 6) Maximum impact scenario | *Ad-valorem* tax and tiered tax | Same calculations as in the *ad-valorem* tax scenario but with additional reformulation as in the tiered tax scenario. |
| 6) Discount rates of 0% / 5% / 10% | All scenarios | Same calculation as in main scenarios (discount rate 3%) but with discount rates of 0% / 5% / 10%. |

Abbreviations: SSBs, sugar-sweetened beverages.

### Health economics module

The health economics module of IMPACT_NCD_ Germany uses the output of the previously described modules to estimate health economic outcomes such as quality-adjusted life years and various costs. In contrast to the other modules of IMPACT_NCD_ Germany, the health economics module consists of multiple post-processing procedures which add health utility and cost information into the already simulated life course of individuals.

In this study, we use these techniques to evaluate the economic impact of the analysed SSB taxation scenarios compared to the baseline scenario without any policy from healthcare and societal perspectives following recent guidelines and recommendations [76, 77]. The healthcare perspective hereby is equivalent to the perspective of the statutory health insurance (SHI) in Germany under which around 85% of the German population are insured. To identify the most recent and relevant cost parameters, we conducted a systematic literature search which we supplemented with hand and forward/backward citation searching. We additionally re-estimated health utilities based on a previous analysis conducted by our team to improve alignment with the scope of our analysis [43]. An overview of the included cost-parameters, their source and respective assumptions is given in **Table L**. An impact inventory according to Neumann et al. (2016) is shown in **Table M** [76].

Table L: Sources and assumptions of cost parameters for the economic evaluation

| Cost parameter | Source | Strata | Comments & assumptions | Likely direction of bias |
| --- | --- | --- | --- | --- |
| Mean annual healthcare costs of otherwise healthy individuals (i.e., no diabetes, CHD, or stroke) | Kähm et al., 2020 [34] | Age | Estimates not adjusted for CHD and stroke; We assume this bias is negligible | ↑ |
| Mean annual healthcare costs of individuals with uncomplicated diabetes | Kähm et al., 2018 [33] | Age, sex | Standard error is set to 2% of mean estimate (sampe size is very large); Does not account for average microvascular complication rate and related costs | ↓ |
| Mean annual healthcare costs of individuals with CHD (without diabetes) | Kähm et al., 2018 [33] | Age, sex | No unique estimate identified; Computed from healthcare costs of people with diabetes and CHD *minus* healthcare costs of uncomplicated diabetes; Different values for incident, prevalent and fatal cases | ↓ |
| Mean annual healthcare costs of individuals with stroke (without diabetes) | Kähm et al., 2018 [33] | Age, sex | No unique estimate identified; Computed from healthcare costs of people with diabetes and stroke *minus* healthcare costs of uncomplicated diabetes; Different values for incident, prevalent and fatal cases; Mean costs across included stroke subtypes | ↓ |
| Mean annual healthcare costs of individuals with CHD and diabetes | Kähm et al., 2018 [33] | Age, sex | Different values for incident, prevalent and fatal cases | - |
| Mean annual healthcare costs of individuals with stroke and diabetes | Kähm et al., 2018 [33] | Age, sex | Different values for incident, prevalent and fatal cases | - |
| Mean annual costs due to early retirement in individuals with/without diabetes | Ulrich et al., 2016 [35] | - | In the source paper, indirect costs consist of sick leave and early retirement costs but only total indirect costs and sick leave costs are reported; Computed from total direct costs minus sick leave costs; Standard error is set to 15% of mean estimate; Estimates not adjusted for CHD and stroke | ↑ |
| Mean annual costs due to early retirement in individuals with stroke | Winter et al., 2008 [36] | - | Based on very low sample size; Uncertainty based on sampling from Gamma distribution with parameters informed by mean estimate and standard error from source (method of moments) [55]; | ↓ |
| Mean annual costs due to sick leave in individuals with/without diabetes | Ulrich et al., 2016 [35] | - | Standard error is set to 15% of mean estimate; Estimates not adjusted for CHD and stroke | ↑ |
| Mean annual costs due to sick leave in individuals with stroke | Winter et al., 2008 [36] | - | Based on very low sample size; Uncertainty based on sampling from Gamma distribution with parameters informed by mean estimate and standard error from source (method of moments) [55]; | ↓ |
| Mean annual time costs for diabetes self-management | Icks et al., 2020 [37] | - | Estimate does not account for time costs of people without type 2 diabetes | ↑ |
| Mean annual time costs for health services use in people with/without diabetes | Icks et al., 2013 [38] | - | Comparably old estimates and small sample size | - |
| Mean annual gross wage | National salary assessment (*Verdienststrukturerhebung*) (2018) [39] | Age, sex | Self-employed not included | ↓ |
| Fringe benefit rate | National assessment of employers’ social security contributions (*Sozialbeiträge der Arbeitgeber* in *Arbeits- und Lohnnebenkosten*) (2020) [40] | - | Self-employed not included | - |
| Consumer price index – Health | DeStatis Genesis COICOP [41] | - | Used for inflation adjustment of health care cost categories | - |
| Price index for labour costs | DeStatis Genesis [42] | - | Used for inflation adjustment of productivity and time costs | - |

Abbreviations: CHD, coronary heart disease; COICOP, Classification of Individual Consumption by Purpose.

Table M: Impact inventory

| Sector | Type of impact | Included in cost perspective? | | Evidence/Notes | Included in evaluation? |
| --- | --- | --- | --- | --- | --- |
|  |  | *Healthcare* | *Societal* |  |  |
| **Formal healthcare sector** | | | | | |
| **Health** | *Health effects* |  |  |  |  |
|  | Longevity effects | x | x |  | Yes |
|  | Health-related quality of life effects | x | x | Measured by EQ-5D-5L* | Yes |
|  | Effects on disease incidence | x | x |  | Yes |
|  | *Medical costs* |  |  |  |  |
|  | Paid for by patients  (out-of-pocket) |  |  | Not relevant in Germany (statutory health insurance) | No |
|  | Paid for by private third-parties |  |  | Healthcare perspective is that of statutory health insurance | No |
|  | Paid for by public third-parties | x | x |  | Yes |
|  | Future related medical costs | x | x |  | Yes |
|  | Future unrelated medical costs | x | x | No data | No |
| **Informal healthcare sector** | | | | | |
| **Health** | Patient time costs for disease management |  | x | Only for individuals with diabetes | Yes |
|  | Patient time costs for health services use |  | x | Only for healthy individuals and those with diabetes | Yes |
| **Non-healthcare sector** | | | | | |
| **Economic** | Lost productivity due to premature death |  | x |  | Yes |
|  | Lost productivity due to early retirement |  | x | Only for individuals with diabetes and stroke | Yes |
|  | Lost productivity due to sick leave |  | x | Only for individuals with diabetes and stroke | Yes |
|  | Cost of unpaid lost productivity |  | x | No data | No |
|  | Cost of lost household production |  | x | No data | No |

*The EQ-5D-5L is a multi-attribute descriptive system which combines the fives dimensions mobility, self-care, usual activities, pain or discomfort, and anxiety or depression [43].

#### Health-related quality of life

To estimate health utility values for synthetic individuals we re-estimated regression equations of a recently published analysis from our team [43]. Re-estimation was required to align the estimated health utilities decrements and values with the scope and stratification of our model. Briefly, the original study assessed health utility in 8,755 participants of the KORA study (S1, S2, S3, S4; 1984-1999) with the EQ-5D-5L multi-attribute descriptive system which combines the fives dimensions mobility, self-care, usual activities, pain or discomfort, and anxiety or depression. The applied value set is based on a German population-based sample [43]. To estimate health utility decrements, calculated health utilities were regressed on T2DM and a range of diabetes-related diseases (hypertension, myocardial infarction, stroke, CHD, cardiac arrhythmia, and heart failure), diabetes-unrelated diseases (chronic obstructive pulmonary disease (COPD), cancer, asthma, and chronic bronchitis), sociodemographic (age, sex, and education) and behavioural covariates (smoking status and BMI). Additionally, microvascular complications of T2DM were considered (neuropathy, peripheral vascular disease, diabetic foot, nephropathy, and blindness). In the linear regression model, interactions between diabetes-related diseases and microvascular T2DM complications were included [43].

In the re-estimation of the original analysis, we excluded smoking and education since our model is not stratified by education and we do not consider smoking as a risk factor. The new estimates thus give us the marginal health utility intercept and decrements irrespective of these characteristics. We also excluded microvascular T2DM complications because we do not explicitly model them in IMPACT_NCD_ Germany. Using the published health utility decrement for T2DM from the original study would thus in the estimation of health utilities assume that prevalent T2DM cases in our model do not develop complications. The new estimate, however, includes the utility decrements of T2DM complications in the T2DM main effect estimate. Finally, we collated the diabetes-related diseases CHD, myocardial infarction, and heart failure into one category to improve consistency with our CHD incidence and mortality data. **Table N** compares the original and the re-estimated health utility decrements.

Table N: Comparison of original and estimated health utility decrements

|  | Original health utility decrement from Laxy et al. [43] | Re-estimated health utility decrement |
| --- | --- | --- |
| Intercept | 1.187 | 1.216 |
| Age (in years) | -0.003 | -0.003 |
| Sex (reference: male) | -0.029 | -0.035 |
| BMI | -0.003 | -0.003 |
| COPD | -0.085 | -0.093 |
| Cancer | -0.025 | -0.025 |
| Asthma | -0.032 | -0.031 |
| Bronchitis | -0.047 | -0.051 |
| Type 2 diabetes | -0.028 | -0.040 |
| Hypertension | 0.005 | 0.006 |
| Type 2 diabetes*hypertension | 0.024 | 0.019 |
| Stroke | -0.070 | -0.073 |
| Type 2 diabetes*stroke | -0.052 | -0.068 |
| Arrhythmia | -0.031 | -0.034 |
| Type 2 diabetes*arrhythmia | 0.001 | -0.021 |
| CHD | -0.028 | -0.066 |
| Type 2 diabetes*CHD | 0.031 | -0.005 |

Abbreviations: BMI, body mass index; CHD, coronary heart disease; COPD, chronic obstructive pulmonary disease.

In IMPACT_NCD_ Germany, the calculation of the health utility values for synthetic individuals is directly performed using the re-estimated regression coefficients. Individual standard errors for the estimated health utilities needed for uncertainty sampling are directly calculated by computing the square-root of the matrix multiplication of the linear predictor with the variance-covariance matrix from the re-estimated regression [55].

#### Systematic literature search for cost parameters in Germany

As described above we conducted a systematic literature search to identify relevant cost parameters for the diseases modelled in this study. We conducted independent searches of the MEDLINE database starting in the year 2010 until June 2022 to identify studies assessing the costs of T2DM, CHD, and stroke in Germany. Studies were included if they were 1) observational studies, modelling studies or review articles; 2) covering the German general population or relevant sub-populations of all ages; 3) assessing a disease of interest (i.e., T2DM, CHD, or stroke) including its potential subtypes as the exposure; 4) having any type of economic costs of a disease of interest as the outcome; 5) without any particular intervention, except large-scale population-based disease management programs; and 6) without comparator or comparing any type of economic costs in people with the disease of interest and those without the condition. **Table O** summarises the eligibility criteria and gives further justification. **Tables P-R** show the applied search terms and number of retrieved records for each of the three disease-specific searches. Studies were first deduplicated and then screened by title and abstract and full text separately. **Figure R** shows the *Preferred Reporting Items for Systematic Reviews and Meta-Analyses* (PRISMA) flow chart and the results of the screening process. We additionally supplemented the results from these systematic searches with additional hand searches and (selective) forward and backwards citation searching. We analysed the 40 included studies that were identified in the systematic search with regards to their usability to parameterise the model with economic consequences resulting from the modelled diseases (**Table L**). Details on the used cost parameters are given in the following sections.

Table O: Eligibility criteria for systematic cost searches

| **Dimension** | **Specification** | **Comment** | **Exclusion** |
| --- | --- | --- | --- |
| Study Design | Observational studies, review articles, modeling studies. | Specifically cost-of-illness studies might have a modeling/extrapolation component. | Study protocols, Randomized Controlled Trials, Guidelines, Treatment Evaluation Studies etc. |
| Population | German general population or sub-groups (including specific other conditions) of all ages. | This is formulated very broad on purpose. | Populations with a specific disease (e.g., people with dementia) and very specific sub-groups that may not be representative of the general population |
| Exposure | Condition/disease of interest, including subtypes | The relevant exposure is always the condition (e.g., stroke, coronary heart disease or diabetes). | Anything else |
| Intervention | No intervention, population-based disease management program | No interventional studies, except population-based disease management programs from which cost estimates in the control group can be used | Treatments (e.g., insulin and other medication), new treatment regimens, lifestyle interventions etc. |
| Comparator | 1. Population without condition/disease of interest (excess costs studies) 2. No comparator (costing studies) 3. No comparator (cost-of-illness studies) | There are three broad groups of studies:  Excess cost studies,  costing studies without comparison or extrapolation, and cost-of-illness studies. | Anything else |
| Outcome | 1. (Average) additive or multiplicative cost of condition/disease per person/year 2. (Average) costs of condition/disease per person/year 3. Overall past or future costs of condition/disease | See above. | Health outcomes, costs of single treatments or treatment regimens etc. |

Table P: MEDLINE search terms to identify studies assessing the cost of diabetes in Germany

|  | **Search terms** | **Hits** |
| --- | --- | --- |
| 1 | ("costs and cost analysis"[Mesh] OR "cost of illness*"[Mesh] OR "Health Expenditures*"[Mesh] OR "health resources"[Mesh] OR "Health Care Costs*"[Mesh] OR "economic*"[Mesh] OR cost*[TIAB] OR "excess cost*"[TIAB] OR "indirect cost"[TIAB] OR "absenteeism"[TIAB] OR "presenteeism"[TIAB] OR "sick leave"[TIAB] OR "loss of productivity"[TIAB] OR "productivity loss"[TIAB] OR "lost productivity"[TIAB] OR "unit cost"[TIAB] OR "payment"[TIAB] OR "budget"[TIAB] OR "spending"[TIAB] OR "economic model"[TIAB] OR "productivity costs"[TIAB] OR "human capital"[TIAB] OR "informal caregiving"[TIAB] OR "friction cost"[TIAB] OR "healthcare costs"[TIAB] OR "health care costs" OR "health costs"[TIAB] OR "cost of illness"[TIAB] OR "disease cost"[TIAB] OR "cost analysis"[TIAB] OR "health expenditure"[TIAB] OR "economic*"[TIAB]) | 1,472,075 |
| 2 | ("Diabetes Mellitus, Type 2"[Mesh] OR "hyperglycemia"[Mesh] OR "type 2 diabetes"[TIAB] OR "diabetes type 2"[TIAB] OR "T2DM"[TIAB] OR "T2D"[TIAB] OR "non-insulin dependent diabetes"[TIAB] OR "diabetes"[TIAB]) | 651,082 |
| 3 | ("German*" [TIAB] OR "Deutsch*" [TIAB]) |  |
| 4 | 1 AND 2 AND 3 | 578 |
| 5 | ("2010/01/01": today[PDAT]) |  |
| 6 | 4 AND 5 | 392 |
| 7 | (humans[Filter]) AND (english[Filter] OR german[Filter])) |  |
| 8 | 6 AND 7 | **340** |

Table Q: MEDLINE search terms to identify studies assessing the cost of coronary heart disease in Germany

|  | **Search terms** | **Hits** |
| --- | --- | --- |
| 1 | ("costs and cost analysis"[Mesh] OR "cost of illness*"[Mesh] OR "Health Expenditures*"[Mesh] OR "health resources"[Mesh] OR "Health Care Costs*"[Mesh] OR "economic*"[Mesh] OR cost*[TIAB] OR "excess cost*"[TIAB] OR "indirect cost"[TIAB] OR "absenteeism"[TIAB] OR "presenteeism"[TIAB] OR "sick leave"[TIAB] OR "loss of productivity"[TIAB] OR "productivity loss"[TIAB] OR "lost productivity"[TIAB] OR "unit cost"[TIAB] OR "payment"[TIAB] OR "budget"[TIAB] OR "spending"[TIAB] OR "economic model"[TIAB] OR "productivity costs"[TIAB] OR "human capital"[TIAB] OR "informal caregiving"[TIAB] OR "friction cost"[TIAB] OR "healthcare costs"[TIAB] OR "health care costs" OR "health costs"[TIAB] OR "cost of illness"[TIAB] OR "disease cost"[TIAB] OR "cost analysis"[TIAB] OR "health expenditure"[TIAB] OR "economic*"[TIAB]) | 1,472,075 |
| 2 | "Myocardial Ischemia" [Mesh] OR "Acute Coronary Syndrome" [Mesh] OR "Angina Pectoris" [Mesh] OR "Coronary Disease*" [Mesh] OR "Coronary Artery Disease" [Mesh] OR "Heart Arrest" [Mesh] OR "Myocardial Infarction*" [Mesh] OR "Heart Failure" [Mesh] OR "coronary heart disease*"[TIAB] OR "ischemic heart disease*"[TIAB] OR "ischaemic heart disease"[TIAB] OR "myocardial infarct*"[TIAB] OR "myocardial failur*"[TIAB] OR "cardiac failur*"[TIAB] OR "heart failure"[TIAB] OR "heart attack*"[TIAB] OR "stenocardia*"[TIAB] OR "coronary syndrome*"[TIAB] OR "coronary disease*"[TIAB] OR "coronary disorder*"[TIAB] OR "coronary event*"[TIAB] OR "coronary failur*"[TIAB] OR "heart arrest*"[TIAB] OR "heart death"[TIAB] OR "cardiac arrest*"[TIAB] OR "heart disorder*"[TIAB] OR "angina"[TIAB] OR "angina pectoris"[TIAB] | 824,188 |
| 3 | ("German*" [TIAB] OR "Deutsch*" [TIAB]) | 162,201 |
| 4 | 1 AND 2 AND 3 | 546 |
| 5 | ("2010/01/01": today[PDAT]) |  |
| 6 | 4 AND 5 | 315 |
| 7 | (humans[Filter]) AND (english[Filter] OR german[Filter])) |  |
| 8 | 6 AND 7 | **284** |

Table R: MEDLINE search terms to identify studies assessing the cost of stroke in Germany

|  | **Search terms** | **Hits** |
| --- | --- | --- |
| 1 | ("costs and cost analysis"[Mesh] OR "cost of illness*"[Mesh] OR "Health Expenditures*"[Mesh] OR "health resources"[Mesh] OR "Health Care Costs*"[Mesh] OR "economic*"[Mesh] OR cost*[TIAB] OR "excess cost*"[TIAB] OR "indirect cost"[TIAB] OR "absenteeism"[TIAB] OR "presenteeism"[TIAB] OR "sick leave"[TIAB] OR "loss of productivity"[TIAB] OR "productivity loss"[TIAB] OR "lost productivity"[TIAB] OR "unit cost"[TIAB] OR "payment"[TIAB] OR "budget"[TIAB] OR "spending"[TIAB] OR "economic model"[TIAB] OR "productivity costs"[TIAB] OR "human capital"[TIAB] OR "informal caregiving"[TIAB] OR "friction cost"[TIAB] OR "healthcare costs"[TIAB] OR "health care costs"OR "health costs"[TIAB] OR "cost of illness"[TIAB] OR "disease cost"[TIAB] OR "cost analysis"[TIAB] OR "health expenditure"[TIAB] OR "economic*"[TIAB]) | 1,472,075 |
| 2 | ("Stroke"[Mesh] OR "stroke*"[TIAB] OR "apoplexy"[TIAB] OR "cerebrovascular accident*"[TIAB] OR "cerebrovascular incident*"[TIAB] OR "brain vascular accident*"[TIAB] OR "cerebrovascular stroke*"[TIAB] OR "cerebral stroke*"[TIAB] OR "acute stroke*"[TIAB] OR "acute cerebrovascular accident*"[TIAB] OR "acute cerebrovascular incident*"[TIAB] OR "cerebrovascular insult*"[TIAB] OR "cerebrovascular disease*"[TIAB] OR "ischemic stroke"[TIAB] OR "ischaemic stroke"[TIAB] OR "hemorrhagic stroke"[TIAB]) | 355,427 |
| 3 | ("German*" [TIAB] OR "Deutsch*" [TIAB]) |  |
| 4 | 1 AND 2 AND 3 | 298 |
| 5 | ("2010/01/01": today[PDAT]) |  |
| 6 | 4 AND 5 | 208 |
| 7 | (humans[Filter]) AND (english[Filter] OR german[Filter])) |  |
| 8 | 6 AND 7 | **177** |

Figure R: PRISMA flow chart of the systematic cost searches


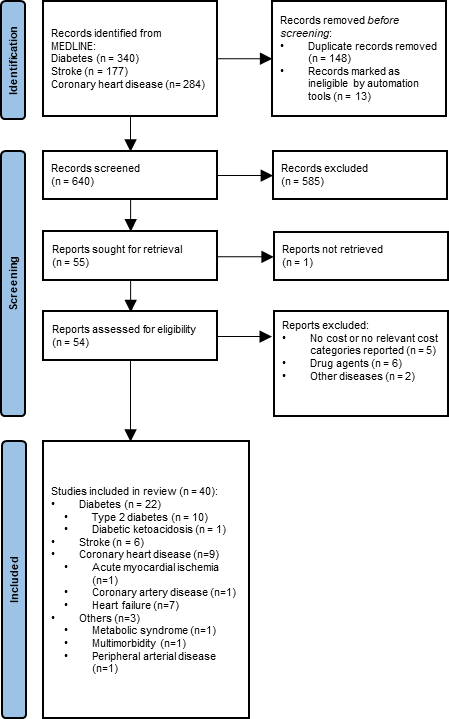


Preferred Reporting Items for Systematic Reviews and Meta-Analyses (PRISMA) flow chart for the systematic search for studies estimating the costs of diabetes, stroke or coronary heart disease.

#### Health sector costs

##### Medical costs related to type 2 diabetes

Medical costs related to T2DM were calculated based on prevalent case-years of T2DM. Based on our systematic search, we identified a high-quality study using nationwide SHI data from 316,220 patients with T2DM from 2013 to 2015 to estimate annual medical costs related to T2DM [33]. In this study, medical costs included costs for inpatient care, outpatient care, medication, rehabilitation, and non-medical aids. The study assessed costs for uncomplicated T2DM and various micro- and macrovascular complications, including their interactions adjusted for age and sex. The extensive supplementary material enabled the calculation of detailed costs for T2DM and the cardiovascular complications that are relevant for this study (i.e., CHD and stroke). However, standard errors/confidence intervals for the needed combinations of age, sex and disease outcomes are not provided. Due to the high sample size in the original analysis and based on reported standard errors for overall estimates, we assumed a standard error of 2% for all estimates extracted from this source study. Exact parameter values are given in **Table S**. In the model, cost estimates were inflated using the health category of the official German consumer price index based on the cost reporting year of the respective source study [41].

##### Medical costs related to CHD

Medical costs related to CHD were calculated based on prevalent case-years of CHD. Based on our systematic search, no studies were identified that enabled the direct parameterisation of annual medical costs related to CHD in the model. We therefore relied on the high-quality indirect evidence from the nationwide SHI study used for medical costs related to T2DM (see above). We assumed that the difference between medical costs in patients with T2DM and CHD and medical costs in patients with uncomplicated T2DM was a good approximation of the medical costs related to CHD. Based on this study we were further able to distinguish between costs for incidence, prevalent and fatal cases of CHD. We here also assumed a standard error of 2% for all estimates. Exact parameter values are given in **Table S**. In the model, cost estimates were inflated using the health category of the official German consumer price index based on the cost reporting year of the respective source study [41].

##### Medical costs related to stroke

Medical costs related to stroke were calculated based on prevalent case-years of stroke. Based on our systematic search, no studies were identified that enabled the direct parameterisation of annual medical costs related to stroke in the model. We therefore relied on the high-quality indirect evidence from the nationwide SHI study used for medical costs related to T2DM (see above). We assumed that the difference between medical costs in patients with T2DM and stroke and medical costs in patients with uncomplicated T2DM was a good approximation of the medical costs related to stroke. Based on this study we were further able to distinguish between costs for incidence, prevalent and fatal cases of stroke. The extracted estimates are assumed to be the average medical costs across stroke subtypes. We here also assumed a standard error of 2% for all estimates. Exact parameter values are given in **Table S**. In the model, cost estimates were inflated using the health category of the official German consumer price index based on the cost reporting year of the respective source study [41].

##### Unrelated (other) medical costs

Unrelated medical costs were calculated based on all case-years in which simulated individuals were not a prevalent case of one of the modelled diseases. Based on our systematic search, no studies were identified that enabled the direct parameterisation of annual baseline/other medical costs not related to any of the modelled diseases. We here relied on high-quality indirect evidence from an excess T2DM cost study using nationwide SHI data [34]. This study analysed 291,709 patients with T2DM and compared their medical costs with an age- and sex-matched control group of the same size. We used age-specific costs from this control group reported in the study as baseline costs unrelated to any of the modelled diseases. This enables us to include costs in additional healthy years lived in the economic evaluation. While these estimates are slightly too high since they include costs for CHD and stroke, we here assume that the arising bias is negligible. Standard errors were based on the confidence intervals reported in the study. Exact parameter values are given in **Table S**. In the model, cost estimates were inflated using the health category of the official German consumer price index based on the cost reporting year of the respective source study [41].

#### Costs outside the formal health sector

##### Productivity costs related to type 2 diabetes

Productivity costs related to T2DM were calculated based on prevalent case-years of T2DM. Based on our systematic search we identified a study that estimated annual costs related to early retirement and sick leave due to T2DM using a human capital approach based on 9,160 observations from 6,803 participants of the KORA study [35]. In the survey administered in the KORA study, costs due to sick leave days were assessed with a question about the number of sick leave days in the previous 12 months. Costs due to early retirement were assessed based on participants indication of receiving incapacity benefits. The study directly reported the total estimated costs due to sick leave or early retirement and the estimated costs due to sick leave separately for individuals with and without T2DM. We could therefore directly parameterise the model with the latter but had to disaggregate the total estimate to be able to parameterise the model with an estimate of costs due to early retirement alone. We assumed that the difference between the reported total estimated costs due to sick leave or early retirement and the estimated costs due to sick leave was a good approximation of the costs due to early retirement. The standard error for the estimated costs due to sick leave, was based on the reported confidence interval. The standard error for the estimated costs due to early retirement was assumed to be 15% based on the standard error of the total estimate. Exact parameter values are given in **Table T**. In the model, cost estimates were inflated using the German price index for labour costs based on the cost reporting year of the respective source study [42].

##### Productivity costs related to stroke

Productivity costs related to stroke were calculated based on prevalent case-years of stroke. Based on our systematic search we did not identify a study that estimated annual costs related to early retirement and sick leave due to stroke. However, we identified a study via additional hand searches that used a bottom-up costing approach to estimate the stroke-associated costs of sick leave and early retirement via a human capital approach [36]. Unfortunately, the data used in this study are comparably old and sample consists of only 151 patients from one university hospital in Germany. However, we included estimates from this study to parameterise stroke-related productivity costs since specifically costs due to stroke-related early retirement might be relevant for the economic evaluation of SSB taxes. However, due to the small sample size we did not directly implement the reported cost estimates. We instead used the reported costs to separately estimate the parameters of a Gamma distribution with the method of moments. The actual values used in the model are sampled from this Gamma distribution. Exact parameter values are given in **Table T**. In the model, cost estimates were inflated using the German price index for labour costs based on the cost reporting year of the respective source study [42].

##### Productivity costs related to premature death

Premature death was defined as death before the official German retirement age of 65 years. Productivity costs due to premature death were calculated using a human capital approach and based on mean annual gross wages reported by the last official national salary assessment (NSA) in 2018 (*Verdienststrukturerhebung 2018*) and the average fringe benefit rate calculated from the last official national assessment of employers’ social security contributions in 2020 (*Sozialbeiträge der Arbeitgeber in Arbeits- und Lohnkosten 2020*). To account for the fact that simulated individuals may die randomly during each simulated year, we subtract half of the estimated productivity loss in the year of death. The NSA is based on a stratified (i.e., state, economic sector, company size) random sample of around 60,000 German employers and one million employment relationships. Employers need to provide information about salaries by law. The income of self-employed individuals is not included in the resulting values. The German federal statistical office provides information that the margin of error on the higher aggregation levels (in our case the average across all economic sectors, states, and company sizes) is very small and around 0%. We therefore did not consider uncertainty in the productivity costs related to premature death. In the model, cost estimates were inflated using the German price index for labour costs [42].

##### Time costs

Based on our systematic search we identified two studies that assessed the annual time costs associated with T2DM related to self-management of the disease and health services use [37, 38]. Both studies were again based on the KORA study, albeit on different samples. The study assessing T2DM self-management included 227 participants with T2DM. Self-management activities were assessed using a validated questionnaire. The study assessing time costs of health services use included 250 participants of which 221 had normal glucose values and 29 diagnosed diabetes. While estimates from both studies are limited due to the small sample sizes, the estimated time costs due to self-management are potentially biased upwards because the study did not compare time costs to a control group without T2DM. For both studies we extracted the reported estimates to parameterise time costs in the model. Standard errors were calculated based on the respective confidence intervals. Exact parameter values are given in **Table T**. In the model, cost estimates were inflated using the German price index for labour costs based on the cost reporting year of the respective source study [42].

Table S: Health sector cost parameter values used in the economic evaluation

| Disease | Complication | Sex | Age group (in years) | Mean value* | Standard error | Uncertainty distribution | Source |
| --- | --- | --- | --- | --- | --- | --- | --- |
| None | - | Female | <50 | €1,019 | 8.67 | Normal | [34] |
|  |  |  | 50-59 | €1,483 | 9.18 |  |  |
|  |  |  | 60-69 | €2,140 | 10.20 |  |  |
|  |  |  | 70-79 | €3,156 | 14.80 |  |  |
|  |  |  | 80+ | €3,975 | 38.78 |  |  |
|  |  | Male | <50 | €1,019 | 8.67 |  |  |
|  |  |  | 50-59 | €1,483 | 9.18 |  |  |
|  |  |  | 60-69 | €2,140 | 10.20 |  |  |
|  |  |  | 70-79 | €3,156 | 14.80 |  |  |
|  |  |  | 80+ | €3,975 | 38.78 |  |  |
| Type 2 diabetes | - | Female | <50 | €3,001 | 2% of mean value | Normal | [33] |
|  |  |  | 50-59 | €2,889 |  |  |  |
|  |  |  | 60-69 | €2,864 |  |  |  |
|  |  |  | 70-79 | €3,052 |  |  |  |
|  |  |  | 80+ | €2,388 |  |  |  |
|  |  | Male | <50 | €2,102 |  |  |  |
|  |  |  | 50-59 | €2,296 |  |  |  |
|  |  |  | 60-69 | €2,574 |  |  |  |
|  |  |  | 70-79 | €2,911 |  |  |  |
|  |  |  | 80+ | €2,558 |  |  |  |
| Type 2 diabetes | CHD (incident) | Female | <50 | €8,894 | 2% of mean value | Normal | [33] |
|  |  |  | 50-59 | €11,110 |  |  |  |
|  |  |  | 60-69 | €8,563 |  |  |  |
|  |  |  | 70-79 | €8,806 |  |  |  |
|  |  |  | 80+ | €8,195 |  |  |  |
|  |  | Male | <50 | €7,287 |  |  |  |
|  |  |  | 50-59 | €9,401 |  |  |  |
|  |  |  | 60-69 | €9,296 |  |  |  |
|  |  |  | 70-79 | €9,123 |  |  |  |
|  |  |  | 80+ | €8,225 |  |  |  |
| Type 2 diabetes | CHD (prevalent) | Female | <50 | €7,092 | 2% of mean value | Normal | [33] |
|  |  |  | 50-59 | €4,469 |  |  |  |
|  |  |  | 60-69 | €3,614 |  |  |  |
|  |  |  | 70-79 | €4,957 |  |  |  |
|  |  |  | 80+ | €4,832 |  |  |  |
|  |  | Male | <50 | €2,080 |  |  |  |
|  |  |  | 50-59 | €4,649 |  |  |  |
|  |  |  | 60-69 | €4,362 |  |  |  |
|  |  |  | 70-79 | €3,703 |  |  |  |
|  |  |  | 80+ | €4,186 |  |  |  |
| Type 2 diabetes | CHD (fatal) | Female | <50 | €10,124 | 2% of mean value | Normal | [33] |
|  |  |  | 50-59 | €10,054 |  |  |  |
|  |  |  | 60-69 | €10,038 |  |  |  |
|  |  |  | 70-79 | €10,156 |  |  |  |
|  |  |  | 80+ | €9,741 |  |  |  |
|  |  | Male | <50 | €24,209 |  |  |  |
|  |  |  | 50-59 | €24,319 |  |  |  |
|  |  |  | 60-69 | €24,475 |  |  |  |
|  |  |  | 70-79 | €24,665 |  |  |  |
|  |  |  | 80+ | €24,466 |  |  |  |
| Type 2 diabetes | Stroke (incident) | Female | <50 | €24,804 | 2% of mean value | Normal | [33] |
|  |  |  | 50-59 | €15,575 |  |  |  |
|  |  |  | 60-69 | €15,018 |  |  |  |
|  |  |  | 70-79 | €14,781 |  |  |  |
|  |  |  | 80+ | €11,592 |  |  |  |
|  |  | Male | <50 | €13,584 |  |  |  |
|  |  |  | 50-59 | €13,459 |  |  |  |
|  |  |  | 60-69 | €14,684 |  |  |  |
|  |  |  | 70-79 | €14,391 |  |  |  |
|  |  |  | 80+ | €12,465 |  |  |  |
| Type 2 diabetes | Stroke (prevalent) | Female | <50 | €34,123 | 2% of mean value | Normal | [33] |
|  |  |  | 50-59 | €14,036 |  |  |  |
|  |  |  | 60-69 | €10,534 |  |  |  |
|  |  |  | 70-79 | €9,804 |  |  |  |
|  |  |  | 80+ | €5,979 |  |  |  |
|  |  | Male | <50 | €7,958 |  |  |  |
|  |  |  | 50-59 | €7,478 |  |  |  |
|  |  |  | 60-69 | €10,070 |  |  |  |
|  |  |  | 70-79 | €9,361 |  |  |  |
|  |  |  | 80+ | €6,014 |  |  |  |
| Type 2 diabetes | Stroke (fatal) | Female | <50 | €11,263 | 2% of mean value | Normal | [33] |
|  |  |  | 50-59 | €11,193 |  |  |  |
|  |  |  | 60-69 | €11,177 |  |  |  |
|  |  |  | 70-79 | €11,295 |  |  |  |
|  |  |  | 80+ | €10,880 |  |  |  |
|  |  | Male | <50 | €12,765 |  |  |  |
|  |  |  | 50-59 | €12,874 |  |  |  |
|  |  |  | 60-69 | €13,031 |  |  |  |
|  |  |  | 70-79 | €13,220 |  |  |  |
|  |  |  | 80+ | €13,021 |  |  |  |
| CHD (incident) | - | Female | <50 | €5,893 | 2% of mean value | Normal | [33] |
|  |  |  | 50-59 | €8,221 |  |  |  |
|  |  |  | 60-69 | €5,699 |  |  |  |
|  |  |  | 70-79 | €5,754 |  |  |  |
|  |  |  | 80+ | €5,807 |  |  |  |
|  |  | Male | <50 | €5,185 |  |  |  |
|  |  |  | 50-59 | €7,105 |  |  |  |
|  |  |  | 60-69 | €6,722 |  |  |  |
|  |  |  | 70-79 | €6,212 |  |  |  |
|  |  |  | 80+ | €5,667 |  |  |  |
| CHD (prevalent) | - | Female | <50 | €4,091 | 2% of mean value | Normal | [33] |
|  |  |  | 50-59 | €1,580 |  |  |  |
|  |  |  | 60-69 | €750 |  |  |  |
|  |  |  | 70-79 | €1,905 |  |  |  |
|  |  |  | 80+ | €2,444 |  |  |  |
|  |  | Male | <50 | €0 |  |  |  |
|  |  |  | 50-59 | €2,353 |  |  |  |
|  |  |  | 60-69 | €1,788 |  |  |  |
|  |  |  | 70-79 | €792 |  |  |  |
|  |  |  | 80+ | €1,628 |  |  |  |
| CHD (fatal) | - | Female | <50 | €7,123 | 2% of mean value | Normal | [33] |
|  |  |  | 50-59 | €7,165 |  |  |  |
|  |  |  | 60-69 | €7,174 |  |  |  |
|  |  |  | 70-79 | €7,104 |  |  |  |
|  |  |  | 80+ | €7,353 |  |  |  |
|  |  | Male | <50 | €22,107 |  |  |  |
|  |  |  | 50-59 | €22,023 |  |  |  |
|  |  |  | 60-69 | €21,901 |  |  |  |
|  |  |  | 70-79 | €21,754 |  |  |  |
|  |  |  | 80+ | €21,908 |  |  |  |
| Stroke (incident) | - | Female | <50 | €21,803 | 2% of mean value | Normal | [33] |
|  |  |  | 50-59 | €12,686 |  |  |  |
|  |  |  | 60-69 | €12,154 |  |  |  |
|  |  |  | 70-79 | €11,729 |  |  |  |
|  |  |  | 80+ | €9,204 |  |  |  |
|  |  | Male | <50 | €11,482 |  |  |  |
|  |  |  | 50-59 | €11,163 |  |  |  |
|  |  |  | 60-69 | €12,110 |  |  |  |
|  |  |  | 70-79 | €11,480 |  |  |  |
|  |  |  | 80+ | €9,907 |  |  |  |
| Stroke (prevalent) | - | Female | <50 | €31,122 | 2% of mean value | Normal | [33] |
|  |  |  | 50-59 | €11,147 |  |  |  |
|  |  |  | 60-69 | €7,670 |  |  |  |
|  |  |  | 70-79 | €6,752 |  |  |  |
|  |  |  | 80+ | €3,591 |  |  |  |
|  |  | Male | <50 | €5,856 |  |  |  |
|  |  |  | 50-59 | €5,182 |  |  |  |
|  |  |  | 60-69 | €7,496 |  |  |  |
|  |  |  | 70-79 | €6,450 |  |  |  |
|  |  |  | 80+ | €3,456 |  |  |  |
| Stroke (fatal) | - | Female | <50 | €8,262 | 2% of mean value | Normal | [33] |
|  |  |  | 50-59 | €8,304 |  |  |  |
|  |  |  | 60-69 | €8,313 |  |  |  |
|  |  |  | 70-79 | €8,243 |  |  |  |
|  |  |  | 80+ | €8,492 |  |  |  |
|  |  | Male | <50 | €10,663 |  |  |  |
|  |  |  | 50-59 | €10,578 |  |  |  |
|  |  |  | 60-69 | €10,457 |  |  |  |
|  |  |  | 70-79 | €10,309 |  |  |  |
|  |  |  | 80+ | €10,463 |  |  |  |
| *The values in this table are not inflation adjusted. Abbreviations: CHD, coronary heart disease. | | | | | | | |

Table T: Non-health sector cost parameters used in the economic evaluation

| Disease | Outcome | Sex | Age group (in years) | | Mean value* | Standard error | Uncertainty distribution | Source |
| --- | --- | --- | --- | --- | --- | --- | --- | --- |
| None | Costs due to sick leave | All | | | €1,299 | 58 | Normal | [35] |
| Type 2 diabetes |  |  |  |  | €3,344 | 688 |  |  |
| Stroke |  |  |  |  | €130 | 870 | Gamma (0.022; 5822) | [36] |
| None | Costs due to early retirement | All | | | €682 | 15% of mean value | Normal | [35] |
| Type 2 diabetes |  |  |  |  | €759 |  |  |  |
| Stroke |  |  |  |  | €1,130 | 1,170 | Gamma (0.932; 1211) | [36] |
| Type 2 diabetes | Time costs for self-management | All | | | €2,068 | 209 | Normal | [37] |
| None | Time costs for health services use | All | | | €2,447 | 838 | Normal | [38] |
| Type 2 diabetes |  |  |  |  | €589 | 78 |  |  |
| None | Human capital costs due to premature death (mean gross annual wages) | Female | | <25 | €13,067 | - | None | [39] |
|  |  |  |  | 25-29 | €28,293 |  |  |  |
|  |  |  |  | 30-34 | €29,700 |  |  |  |
|  |  |  |  | 35-39 | €26,133 |  |  |  |
|  |  |  |  | 40-44 | €25,107 |  |  |  |
|  |  |  |  | 45-49 | €25,431 |  |  |  |
|  |  |  |  | 50-54 | €25,939 |  |  |  |
|  |  |  |  | 55-59 | €25,367 |  |  |  |
|  |  |  |  | 60-64 | €24,168 |  |  |  |
|  |  |  |  | 65+ | €5304 |  |  |  |
|  |  | Male | | <25 | €14,094 |  |  |  |
|  |  |  |  | 25-29 | €32,431 |  |  |  |
|  |  |  |  | 30-34 | €38,100 |  |  |  |
|  |  |  |  | 35-39 | €41,147 |  |  |  |
|  |  |  |  | 40-44 | €42,241 |  |  |  |
|  |  |  |  | 45-49 | €43,841 |  |  |  |
|  |  |  |  | 50-54 | €45,026 |  |  |  |
|  |  |  |  | 55-59 | €43,790 |  |  |  |
|  |  |  |  | 60-64 | €40,812 |  |  |  |
|  |  |  |  | 65+ | €5,400 |  |  |  |
| *The values in this table are not inflation adjusted. | | | | | | | | |

## Model outcomes

The population in the model for this study was the national German population. The model has a time horizon of 30 years, from 2013 to 2043, although our policy scenarios are modelled over 20 years and start from 2023 onwards. The reason the simulation begins in 2013 is to allow for validation and calibration with the observed (2013-2019) and forecast (2020-2043) mortality data used to inform the model. We additionally aimed to align our model as well as possible with our main epidemiological data sources which are mainly from 2011 to 2014. The model outputs produce life-course trajectories for each simulant. From these, annual summary measures such as incidence, prevalence and mortality rates are calculated. These measures can then be compared across time and between scenarios to estimate the effects and equity of different scenarios. Stratification is possible by year, sex, and age group, allowing examination of how effects differ across time and sub-populations.

Crucially, the model is a dynamic, open-cohort microsimulation model. This means the model is trying to estimate the actual impact of the scenarios within a dynamic population where people are born, people age, people’s risk factors change, and people die. The detailed modelling of the population dynamics in our model is thus different from many other population-health and economic models which are often closed-cohort, meaning they follow the same population (and not individuals) over time, and often have a lifetime horizon.

Key outcome measures calculated in this study are the cases and case-years prevented/postponed by disease, all-cause deaths prevented/postponed, life years and quality-adjusted life years gained, differences in life expectancy, overall and at age 60 years, healthcare costs per disease saved, productivity costs (i.e., due to early retirement, sick leave, and premature death) saved, and time costs (i.e., T2DM self-management and health services use) saved. A disease case or case-year is assumed to be prevented/postponed if disease onset (i.e., incidence) in the same individual does not occur or is delayed at minimum by 1 year under one of the modelled SSB taxation scenarios. In contrast to cases prevented/postponed, case-years prevented/postponed additionally measure the additional time a simulated individual may be free of disease under one of the modelled SSB taxation scenarios. For example, let us consider the case where an individual develops T2DM in 2025 in the baseline scenario without SSB tax. Under one of the (counterfactual) SSB taxation scenarios the same individual develops T2DM 5 years later in 2030. Consequently, this would count as one case, but five case-years of T2DM prevented/postponed.

## Uncertainty and probabilistic sensitivity analysis

IMPACT_NCD_ Germany implements a 2^nd^ order Monte Carlo approach to estimate uncertainty intervals (UI) for each scenario [78, 79]. For each iteration, a different set of input parameters is used, by sampling from the respective distributions of input parameters. We assumed log-normal distributions for relative risks and hazard ratios, normal distributions for coefficients of linear regression equations, and uniform distributions for estimates of incidence, prevalence, and case fatality rates. Specifically, for relative risks and hazard ratios, the distributions were bounded above 1 when the mean was above 1 and vice versa.

IMPACT_NCD_ Germany allows stochastic uncertainty, parameter uncertainty, individual heterogeneity, and to some extent structural uncertainty to be propagated in the reported UI. In the model, we minimise stochastic uncertainty by using the same random numbers for all scenarios, when appropriate. The following example illustrates the different types of uncertainty that were considered in the model. Let us assume that the annual risk for CHD is 5%. If we apply this risk to all individuals and randomly draw from a Bernoulli distribution with p = 5% to select those who will manifest CHD, we only consider stochastic uncertainty by using the same random numbers for all scenarios, where appropriate. If we allow the annual risk for CHD to be conditional on individual characteristics (i.e., age, sex, exposure to risk factors), then individual heterogeneity is considered. Finally, when the uncertainty of the relative risks due to sampling errors is considered in the estimation of the annual risk for CHD, the parameter uncertainty is considered. From these three types of uncertainty, only the parameter uncertainty can be reduced by better studies in the future.

The structure of the model is grounded in fundamental epidemiological ideas and well-established causal pathways on which exposures are causally related to the specific NCDs which are explicitly modelled. Hence, structural sensitivity analysis is only sensible within risk factor-disease pairs that are medically established. For example, it would not be necessary to explore the possibility of hypertension being a risk factor for lung cancer (which we do not include in the current model anyway). Although we generally considered this type of uncertainty relatively small for most risk factor-disease relationships, we explicitly account for structural uncertainty in two cases:

First, the discrete-time bias that arises from the fact that time in IMPACT_NCD_ Germany is not continuous. A synthetic individual within the model may die of multiple causes within one year; however, the discrete-time nature of the simulation does not allow the identification of the cause that ‘killed’ the simulant first. Every time this happens to a simulant, we randomly select a cause of death from the list of all the terminal events that occurred for the simulant that year. Hence, we propagate discrete-time uncertainty to the model output.

Second, in this study we explicitly assess the relevance of BMI-independent health effects of SSBs on the risk to develop T2DM and CHD. The implemented estimates are based on recent, high-quality meta-analyses and we believe that an additional sugar-related health risk of SSBs beyond weight gain exists [24]. However, these estimates are more likely to be subject to residual confounding compared to, for example, the very established effect of BMI on the risk to develop T2DM [26]. We therefore model all scenarios both with and without the inclusion of BMI-independent health effects of SSBs on the risk to develop T2DM and CHD.

## Validation and calibration

We validated IMPACT_NCD_ Germany’s epidemiological engine using internal and to some extent external validation. Further, mortality in the model was calibrated to observed mortality rates (2013-2019) and mortality rate projections (2020-2050) as described above (see section Mortality) (**Figures S-X**). We present the relevant validation plots for modelled exposures above in the section Exposure module. Internal validation plots which compare the input with the output incidence and prevalence in the initial simulation year are shown below (**Figures Y-AD**). External validation plots which compare the modelled incidence and prevalence of the modelled diseases to external published data sources are also shown below (**Figures AE-AM**). For the latter we collected disease epidemiological information from past published official German health surveys and ambulatory claims data [80-84]. Overall, the validation results suggest that IMPACT_NCD_ Germany simulates the epidemiology of the modelled diseases in the German population reasonably well for the purpose of this study. Particularly the comparison with external disease epidemiological data is encouraging regarding the validity of IMPACT_NCD_ Germany. When interpreting the results of the external validation, one must account for the fact that the incidence output of IMPACT_NCD_ Germany is the cumulative 1-year incidence, while the prevalence output of IMPACT_NCD_ Germany is the lifetime prevalence rate since we only consider the first event of disease. Different assumptions, datasets and measurement errors in the comparison sources therefore prohibit a direct comparison with our simulated disease epidemiology to some degree and must be factored in.

Figure S: Observed, forecast and calibrated coronary heart disease mortality rates from 2013 to 2043 in men


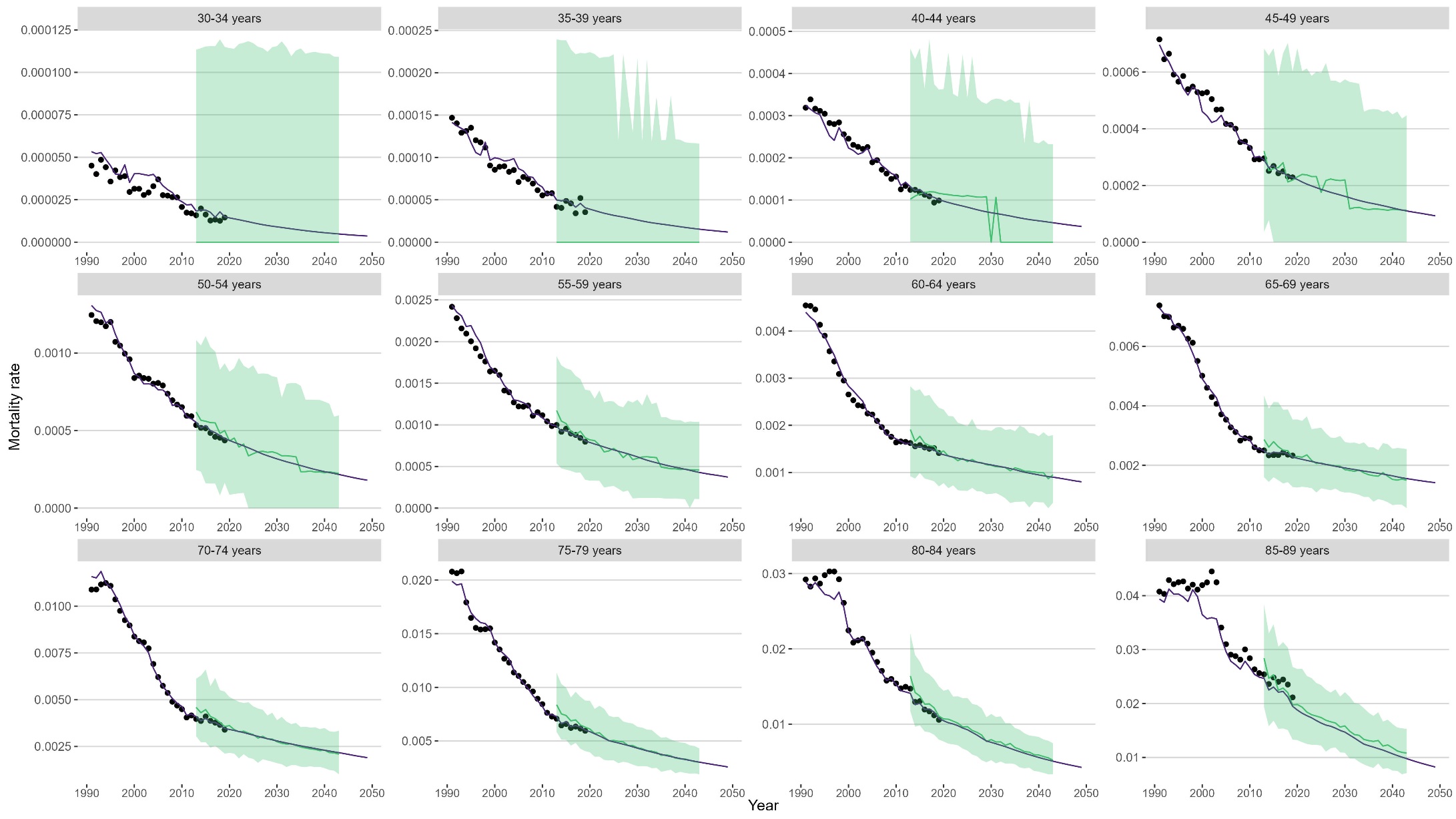
Line plots of observed (1999-2019, black points), by functional demographic model and forecast (1999-2050, purple line), and through IMPACT_NCD_ Germany simulated (2013-2043, green line; shaded area indicate 95%-uncertainty interval) coronary heart diease (CHD) mortality rates in Germany in men.

Figure T: Observed, forecast and calibrated coronary heart disease mortality rates from 2013 to 2043 in women


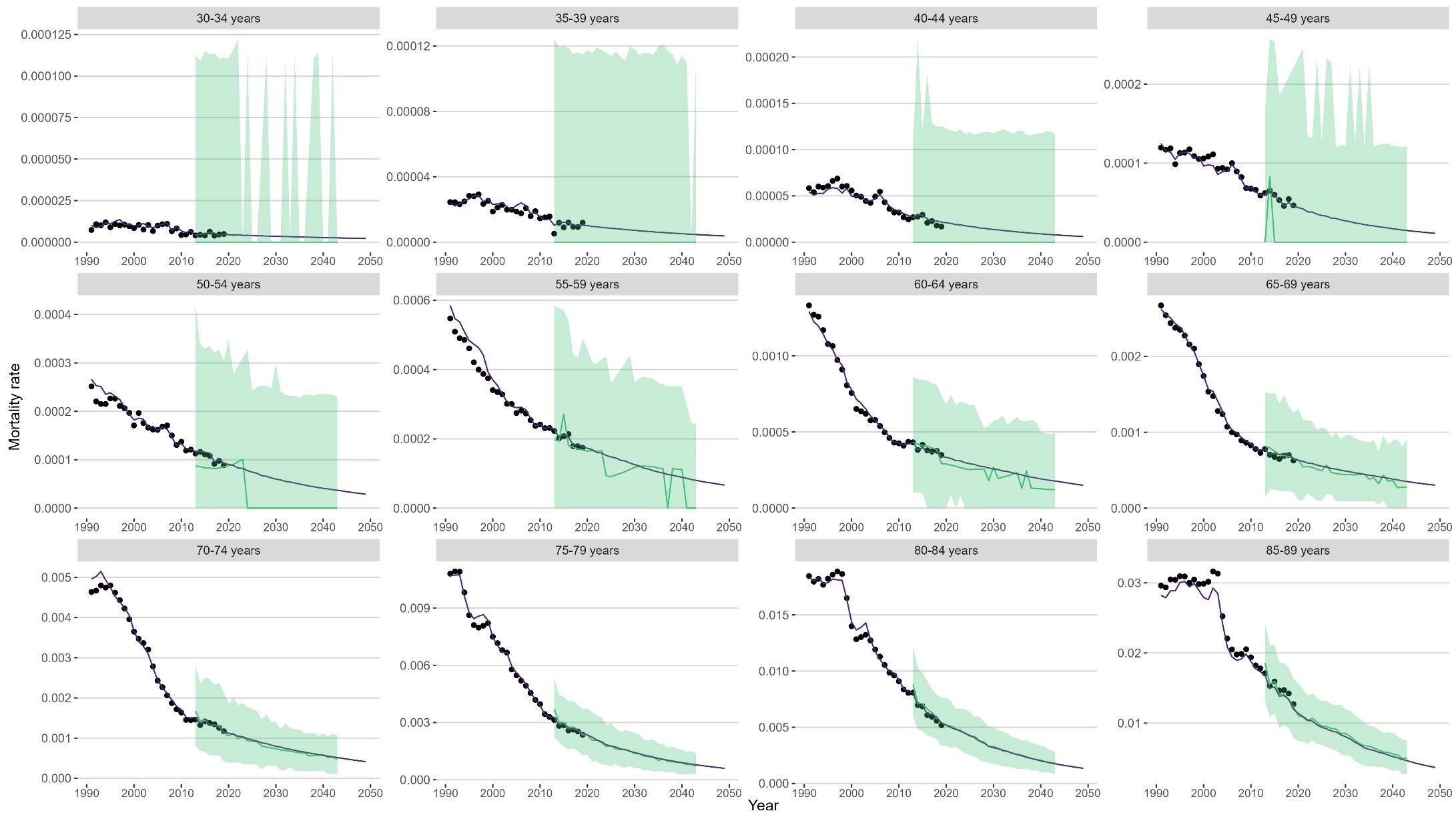
 Line plots of observed (1999-2019, black points), by functional demographic model and forecast (1999-2050, purple line), and through IMPACT_NCD_ Germany simulated (2013-2043, green line; shaded area indicate 95%-uncertainty interval) coronary heart diease mortality (CHD) rates in Germany in women.

Figure U: Observed, forecast and calibrated stroke mortality rates from 2013 to 2043 in men


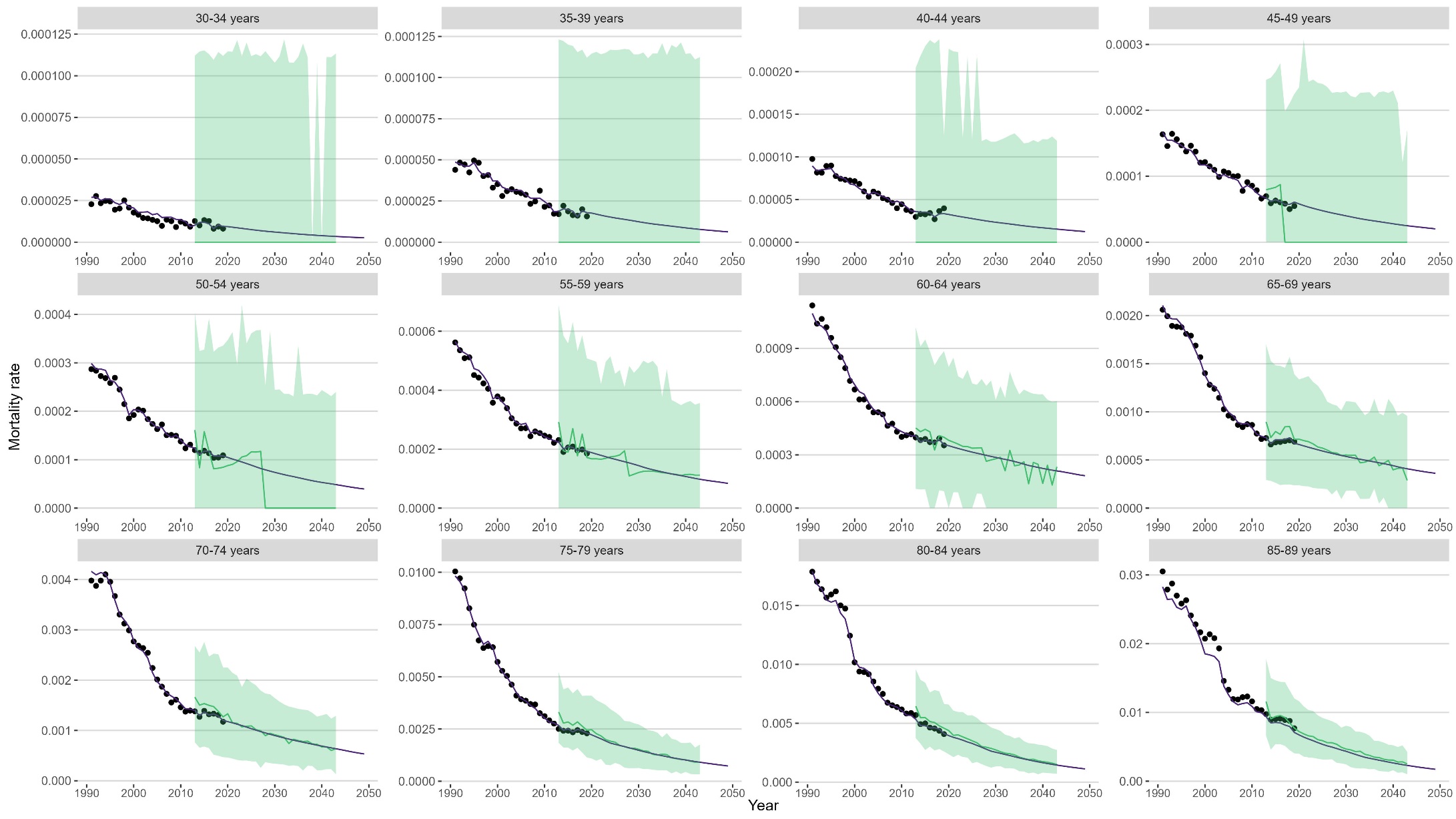
 Line plots of observed (1999-2019, black points), by functional demographic model and forecast (1999-2050, purple line), and through IMPACT_NCD_ Germany simulated (2013-2043, green line; shaded area indicate 95%-uncertainty interval) stroke mortality rates in Germany in men.

Figure V: Observed, forecast and calibrated stroke mortality rates from 2013 to 2043 in women


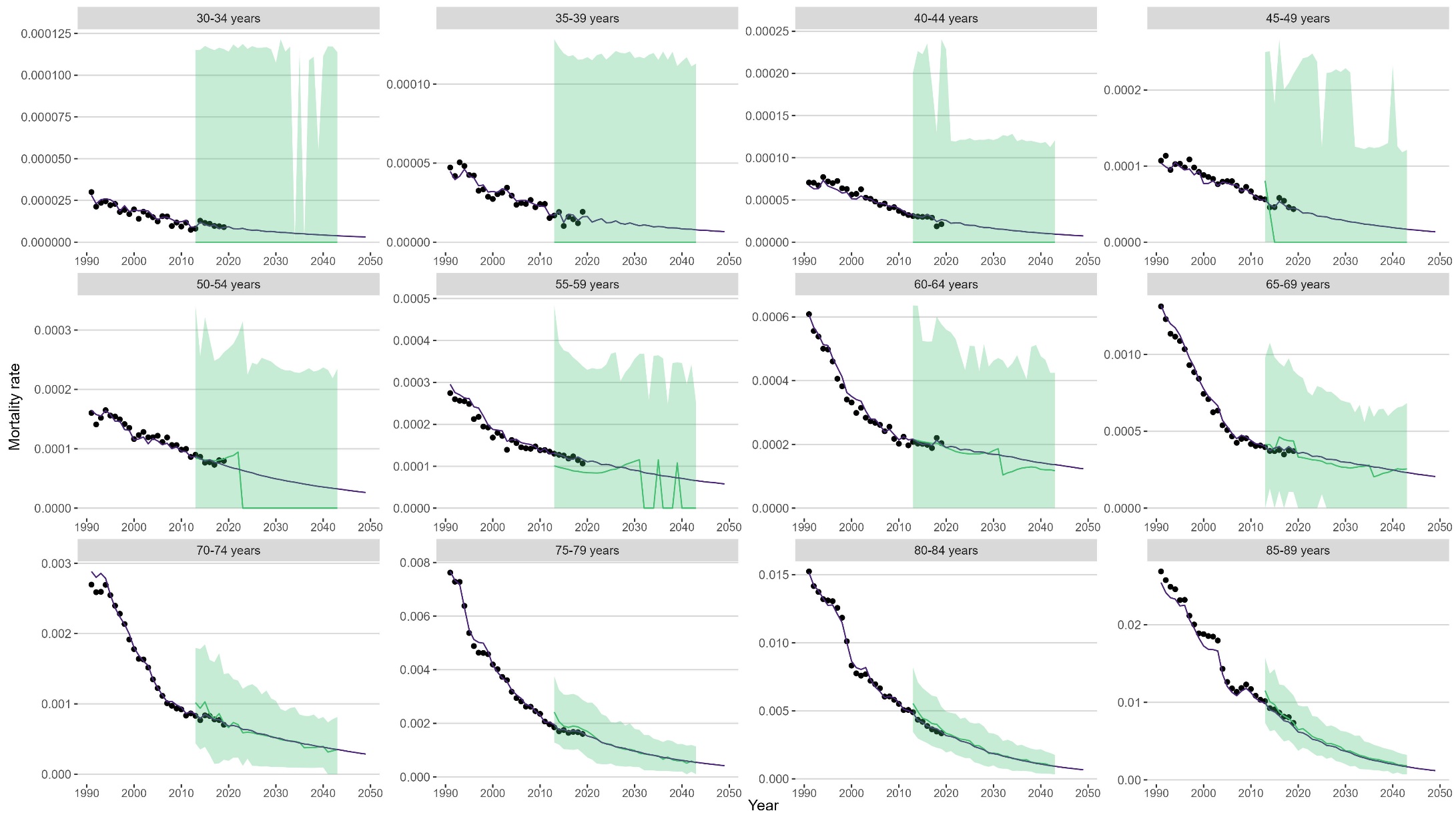
 Line plots of observed (1999-2019, black points), by functional demographic model and forecast (1999-2050, purple line), and through IMPACT_NCD_ Germany simulated (2013-2043, green line; shaded area indicate 95%-uncertainty interval) stroke mortality rates in Germany in women.

Figure W: Observed, forecast and calibrated non-modelled mortality rates from 2013 to 2043 in men


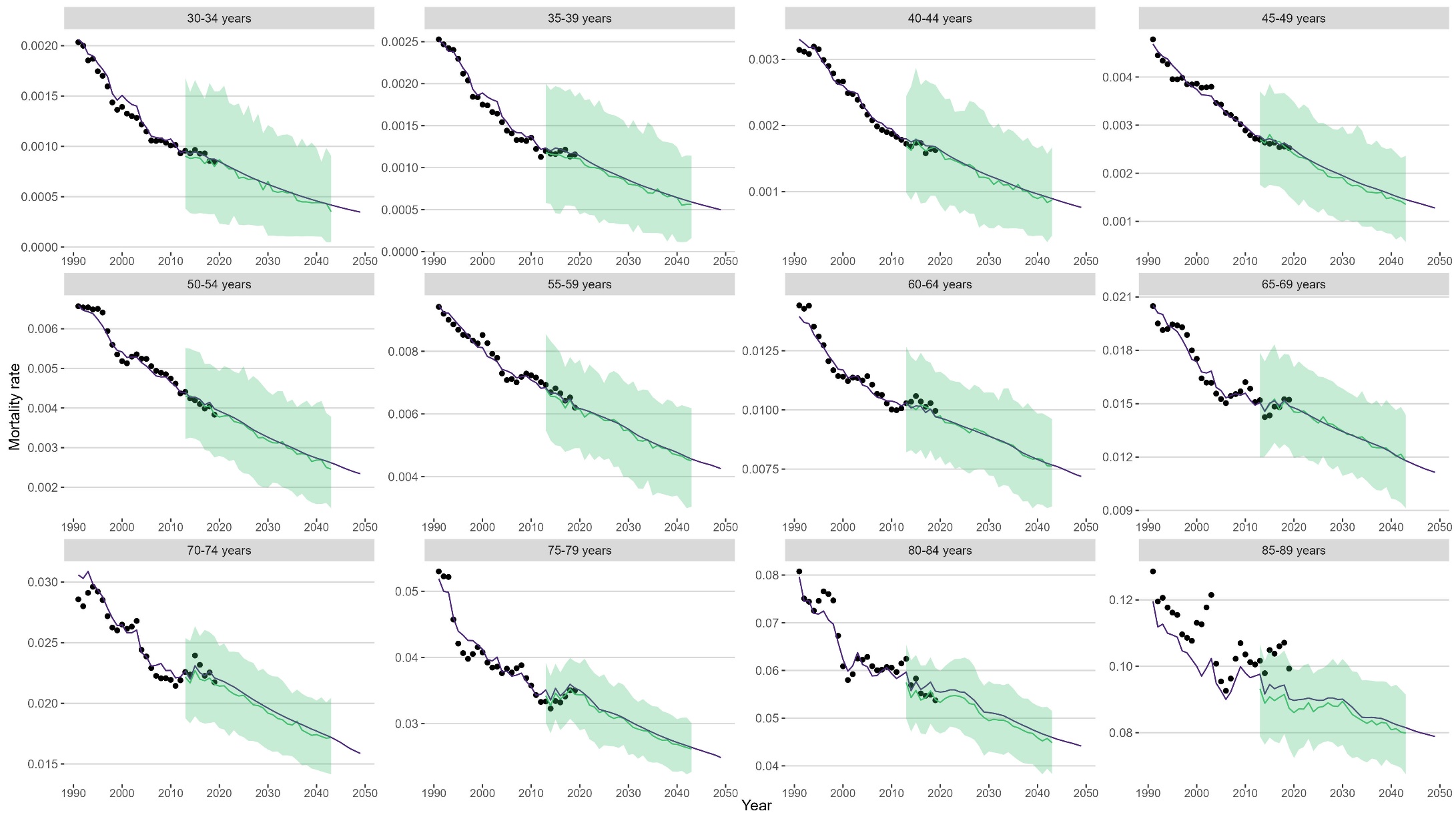
 Line plots of observed (1999-2019, black points), by functional demographic model and forecast (1999-2050, purple line), and through IMPACT_NCD_ Germany simulated (2013-2043, green line; shaded area indicate 95%-uncertainty interval) non-modelled (i.e., non-cardiovascular) mortality rates in Germany in men.

Figure X: Observed, forecast and calibrated non-modelled mortality rates from 2013 to 2043 in women


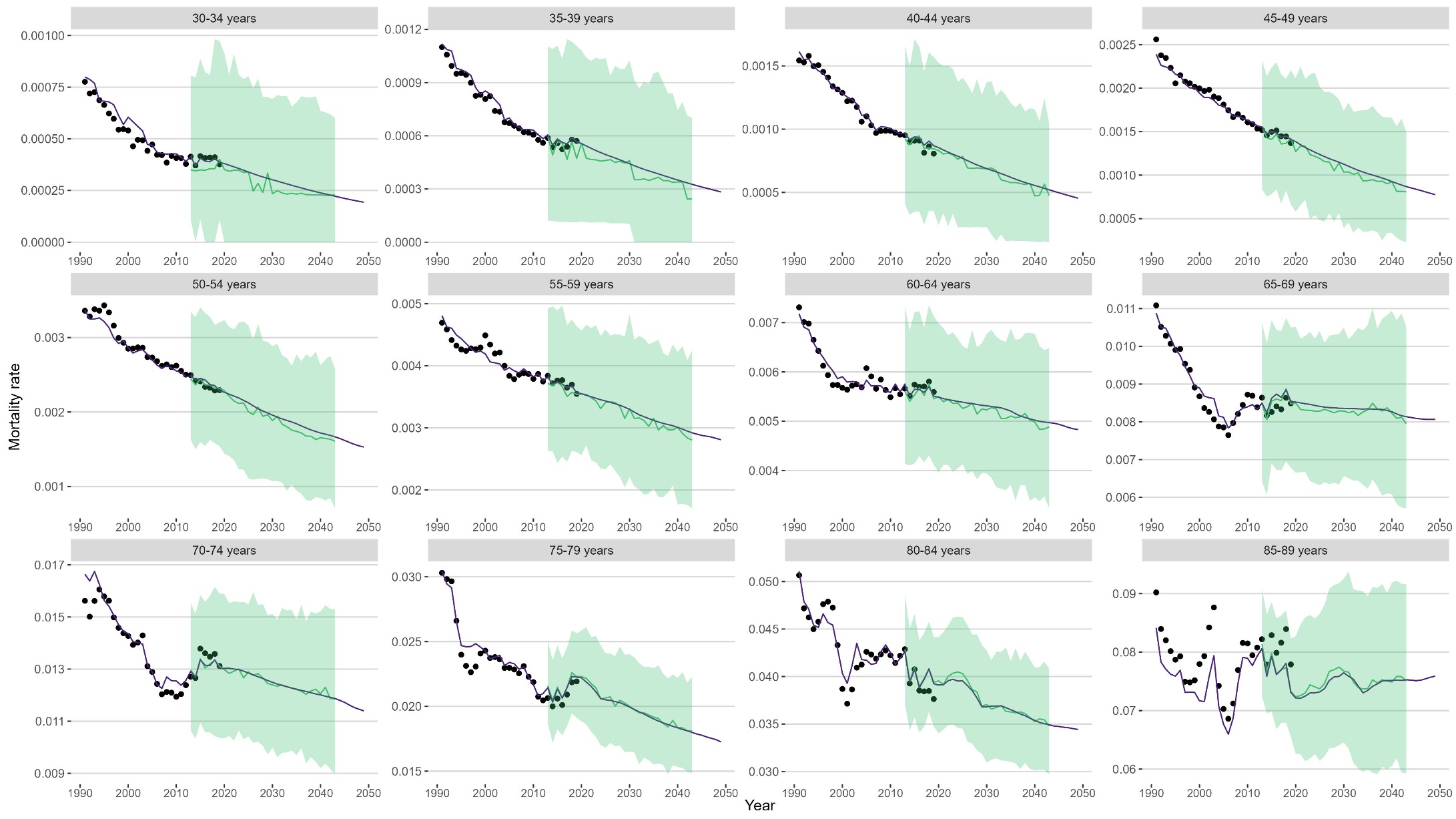
 Line plots of observed (1999-2019, black points), by functional demographic model and forecast (1999-2050, purple line), and through IMPACT_NCD_ Germany simulated (2013-2043, green line; shaded area indicate 95%-uncertainty interval) non-modelled (i.e., non-cardiovascular) mortality rates in Germany in women.

Figure Y: Validation of input versus simulated output coronary heart disease incidence in 2013


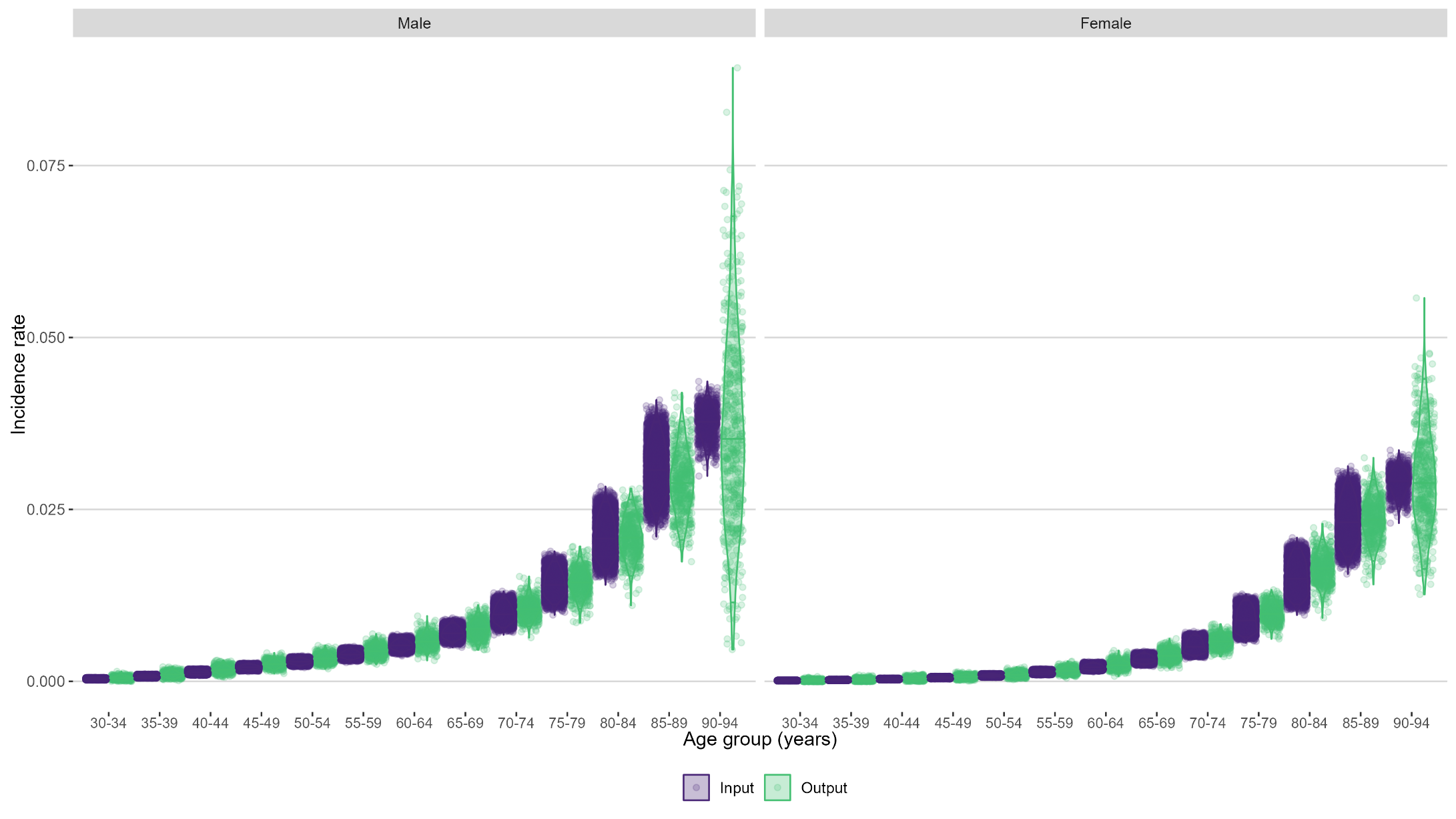
 Point clouds and violin plots indicating input (purple) and simulated output (green) coronary heart disease (CHD) incidence values based on input and output uncertainty (Monte Carlo sampling) by sex and age group in year 2013.

Figure Z: Validation of input versus simulated output stroke incidence in 2013


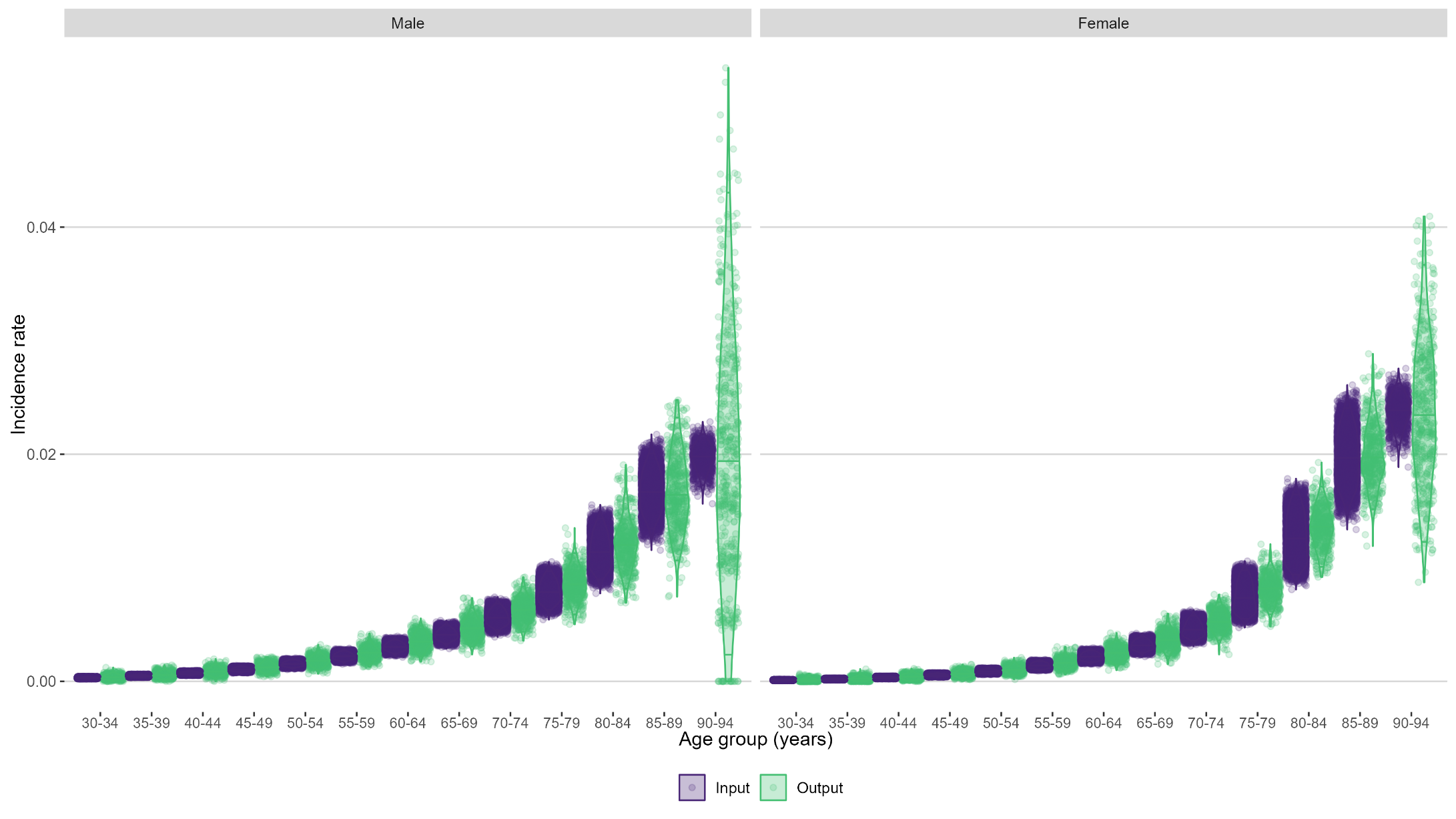
 Point clouds and violin plots indicating input (purple) and simulated output (green) stroke incidence values based on input and output uncertainty (Monte Carlo sampling) by sex and age group in year 2013.

Figure AA: Validation of input versus simulated output type 2 diabetes incidence in 2013


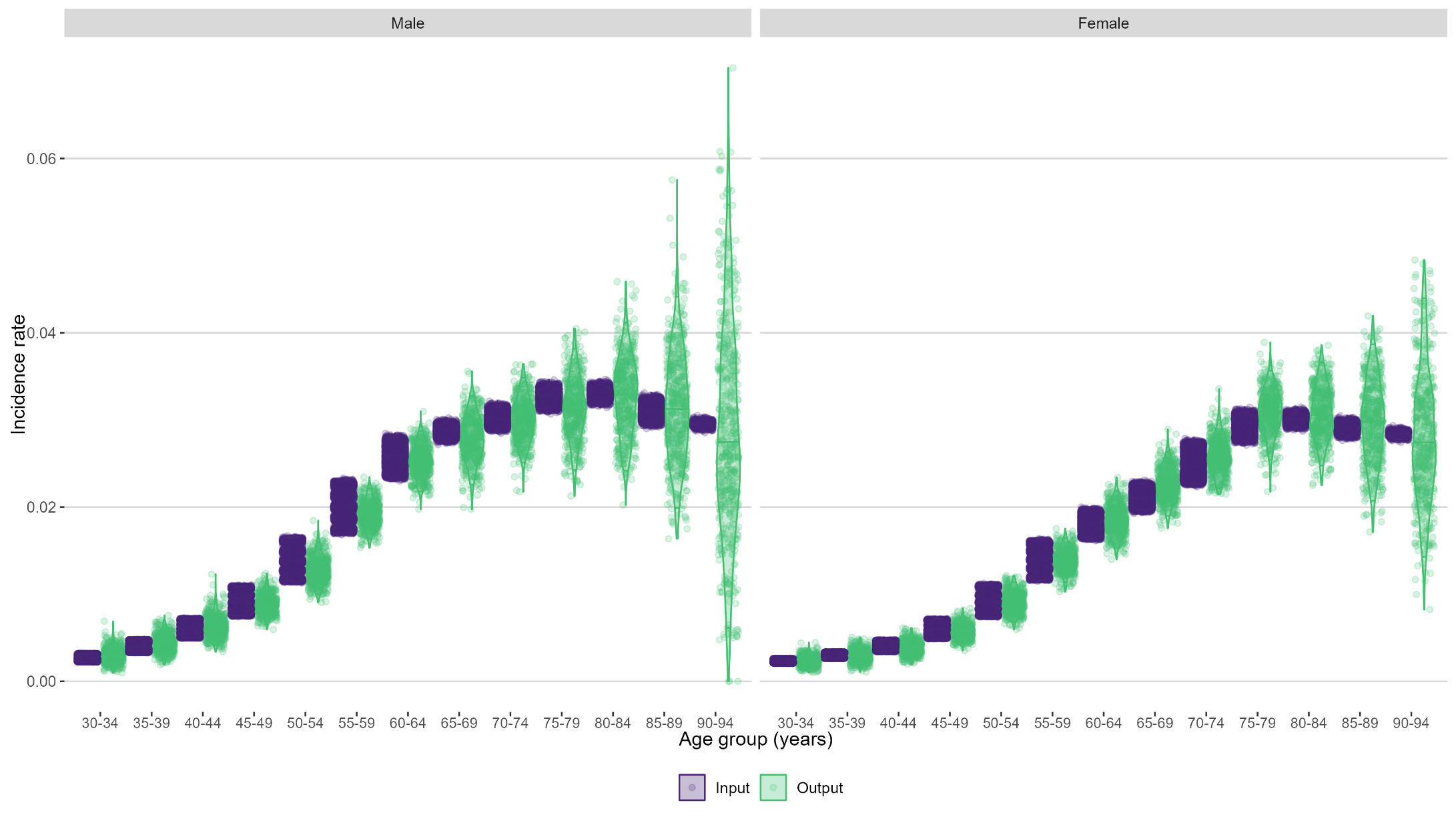
 Point clouds and violin plots indicating input (purple) and simulated output (green) type 2 diabetes incidence values based on input and output uncertainty (Monte Carlo sampling) by sex and age group in year 2013.

Figure AB: Validation of input versus simulated output coronary heart disease prevalence in 2013


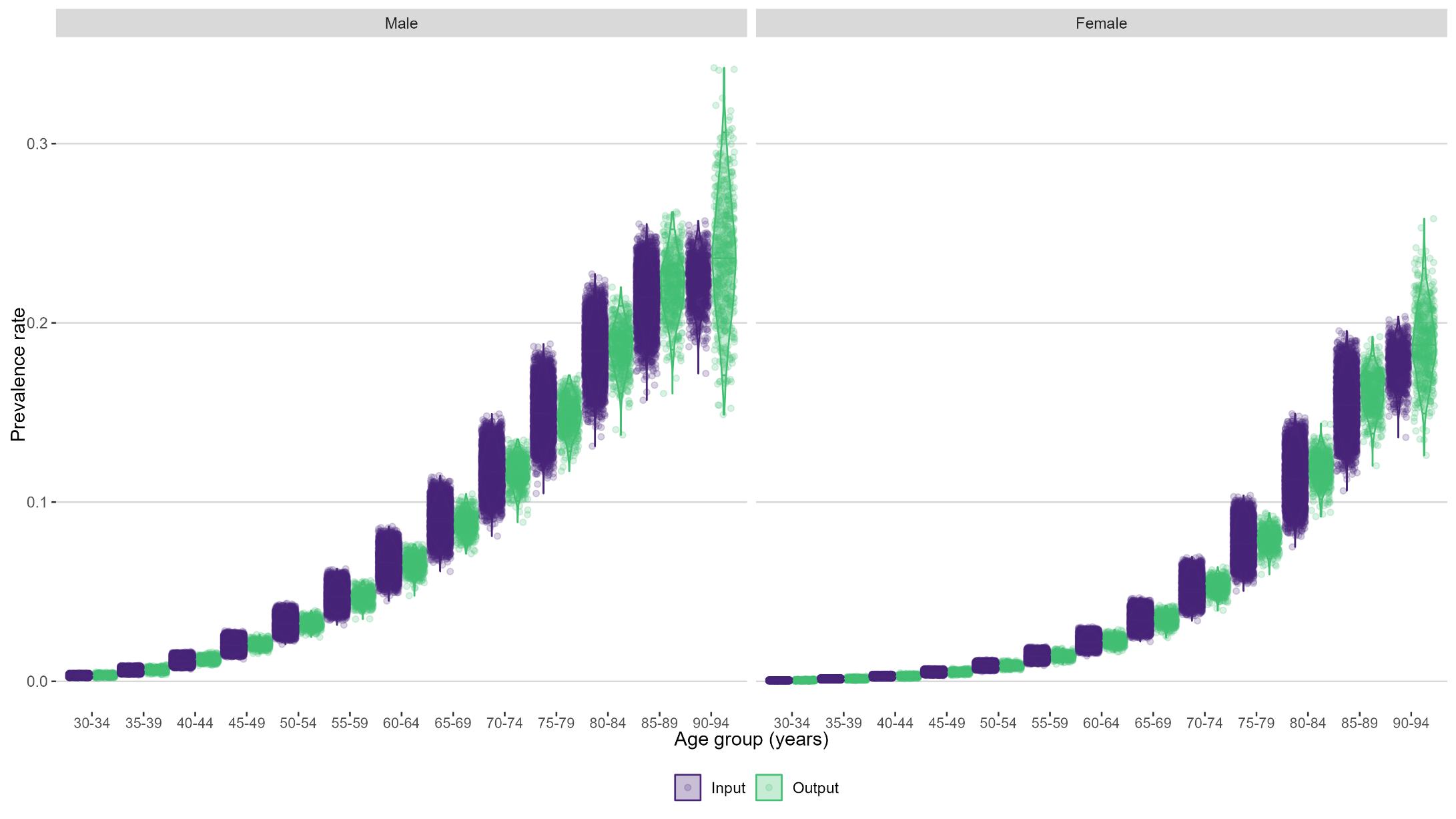
 Point clouds and violin plots indicating input (purple) and simulated output (green) coronary heart disease (CHD) prevalence values based on input and output uncertainty (Monte Carlo sampling) by sex and age group in year 2013.

Figure AC: Validation of input versus simulated output stroke prevalence in 2013


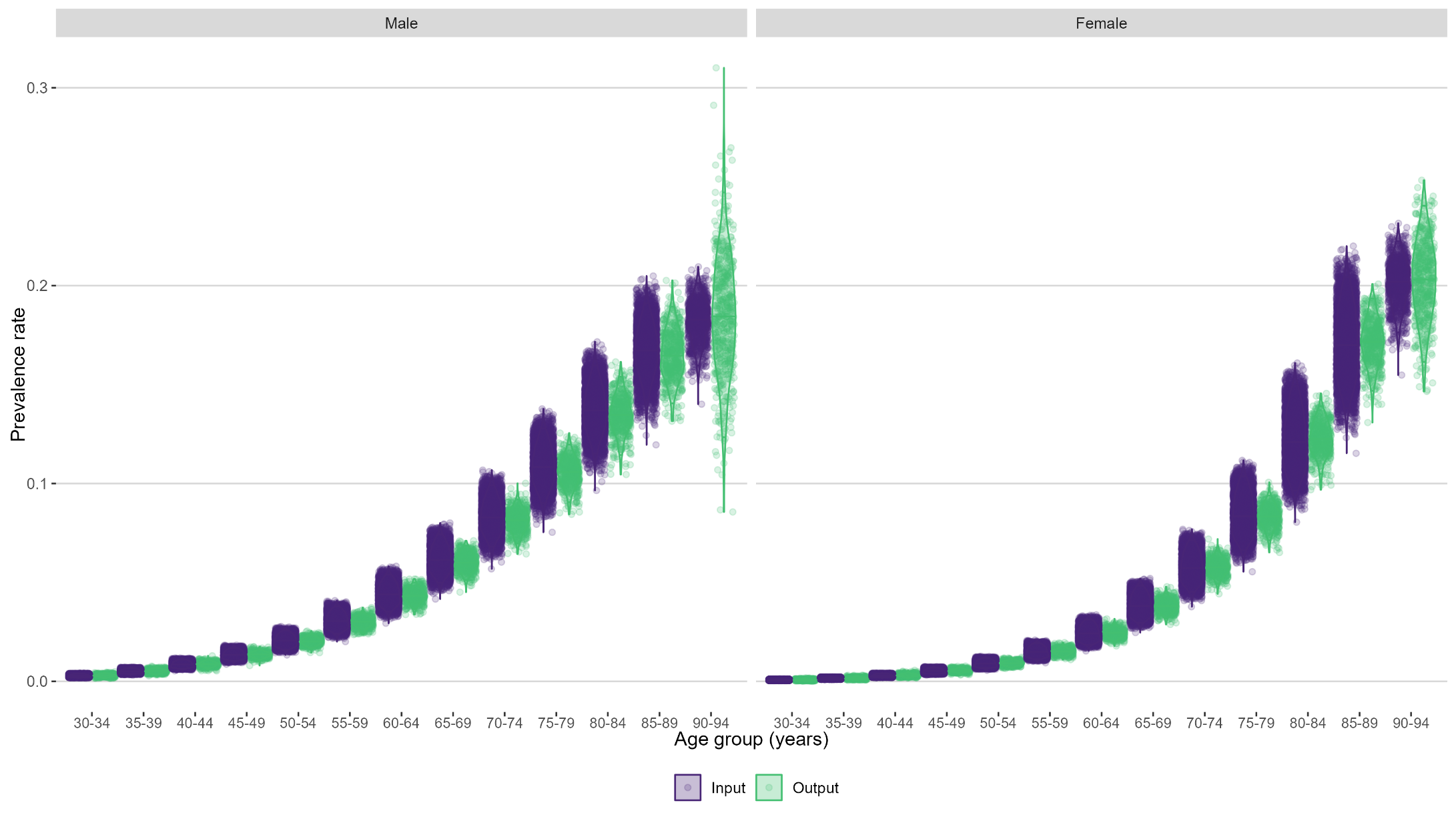
 Point clouds and violin plots indicating input (purple) and simulated output (green) stroke prevalence values based on input and output uncertainty (Monte Carlo sampling) by sex and age group in year 2013.

Figure AD: Validation of input versus simulated output type 2 diabetes prevalence in 2013


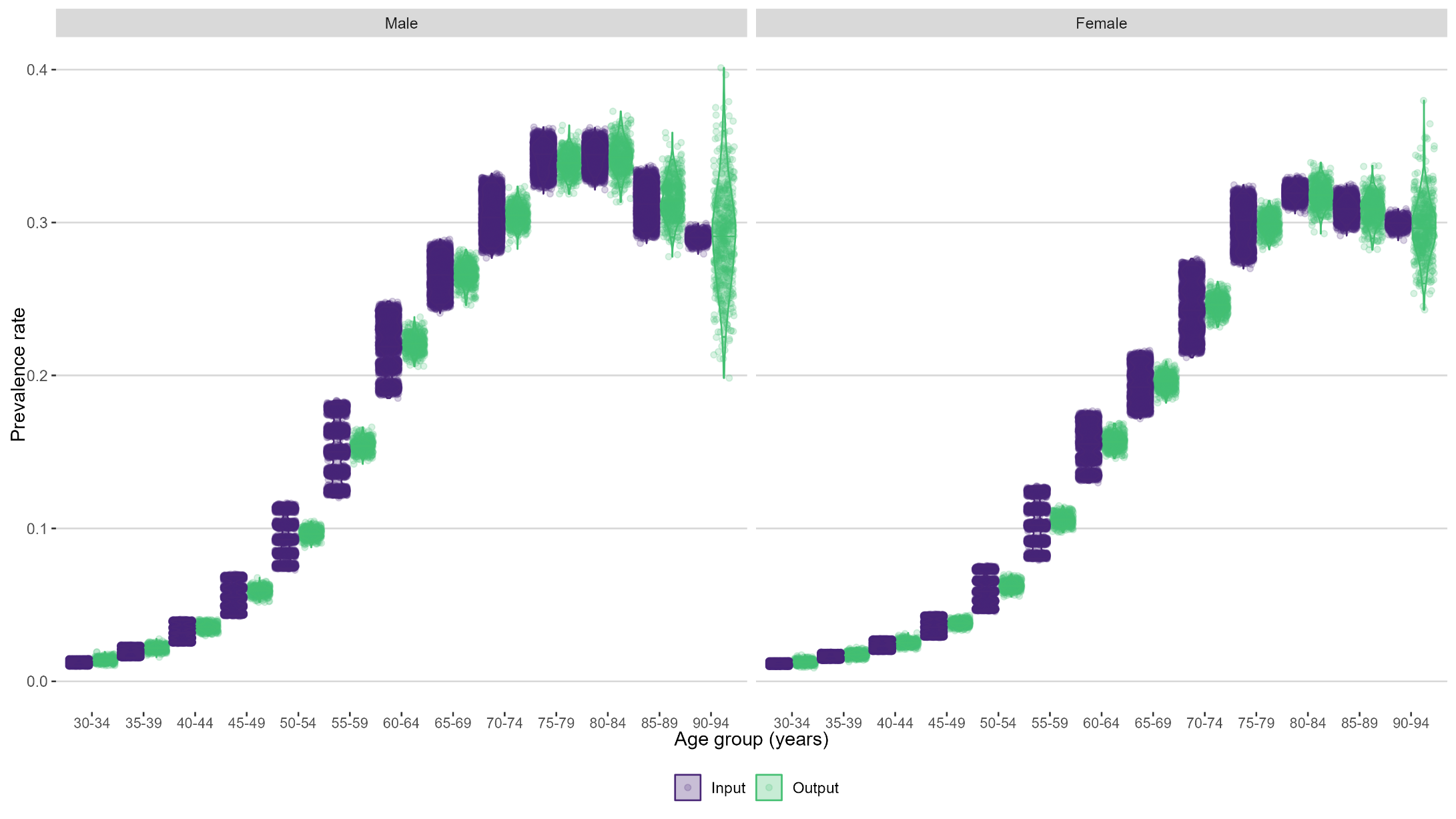
 Point clouds and violin plots indicating input (purple) and simulated output (green) type 2 diabetes prevalence values based on input and output uncertainty (Monte Carlo sampling) by sex and age group in year 2013.

Figure AE: External validation of the simulated coronary heart disease prevalence in 2014 with the GEDA survey by sex and age group


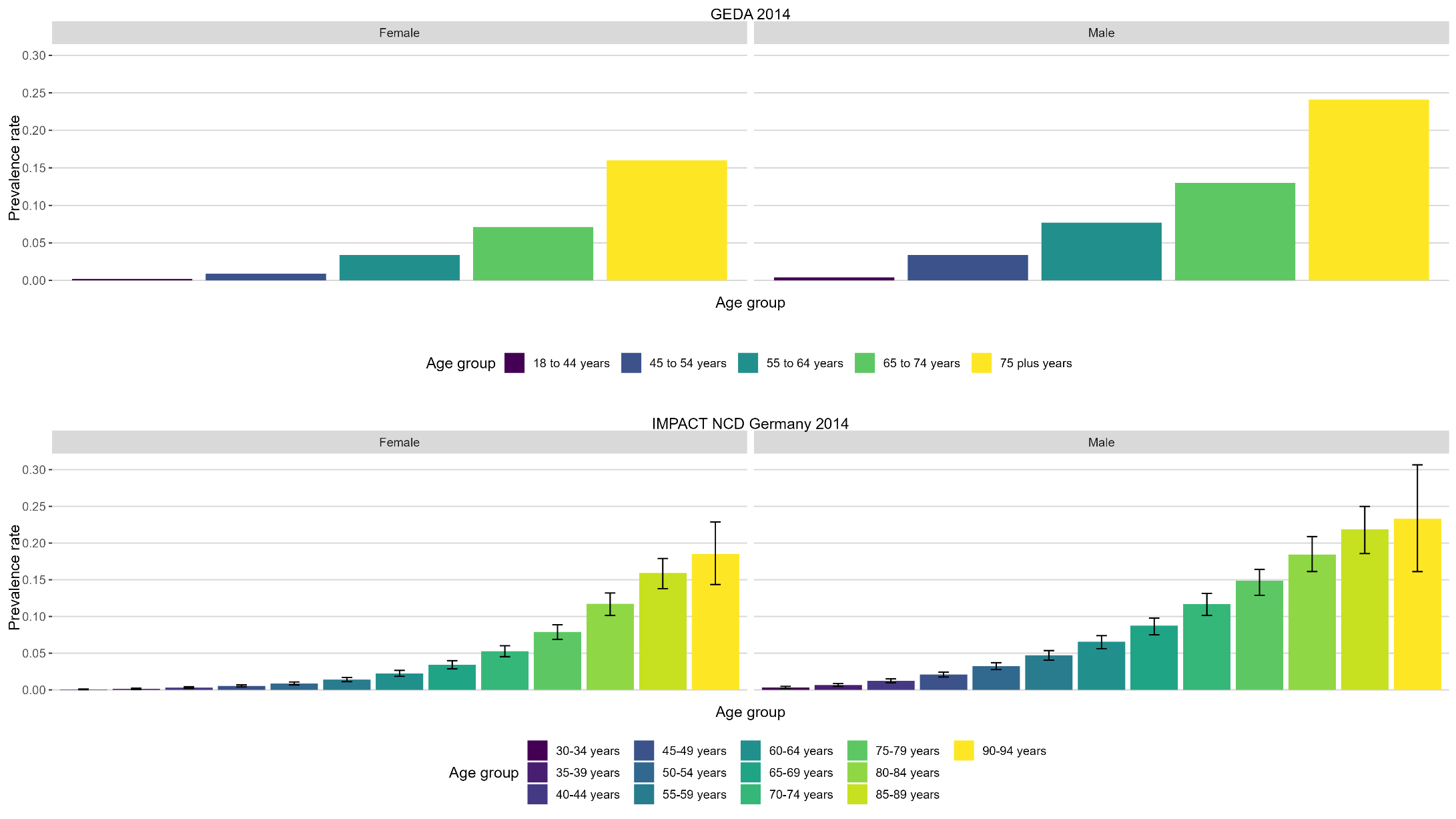
External validation comparison of the prevalence of coronary heart disease (CHD) from the German *Gesundheit in Deutschland Aktuell* (GEDA) telephone survey 2014 and IMPACT_NCD_ Germany by sex and age group [80]. Vertical error bars indicate 95% uncertainty intervals.

Figure AF: External validation of the simulated coronary heart disease prevalence in 2019 with the GEDA survey by sex and age group


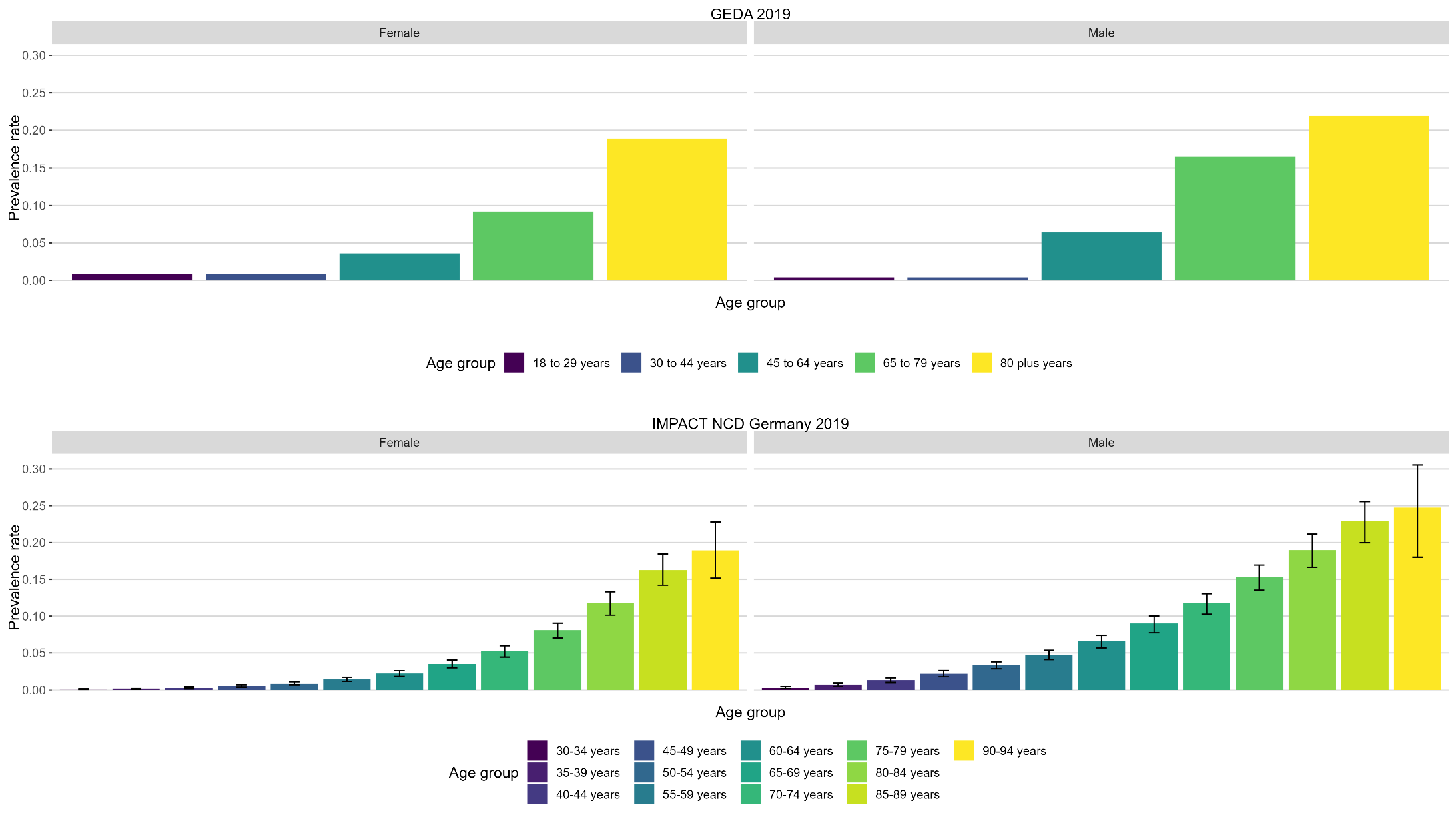
External validation comparison of the prevalence of coronary heart disease (CHD) from the German *Gesundheit in Deutschland Aktuell* (GEDA) telephone survey 2019 and IMPACT_NCD_ Germany by sex and age group [83]. Vertical error bars indicate 95% uncertainty intervals.

Figure AG: External validation of the simulated coronary heart disease prevalence from 2013 to 2018 with ambulatory diagnosis data by sex and age group


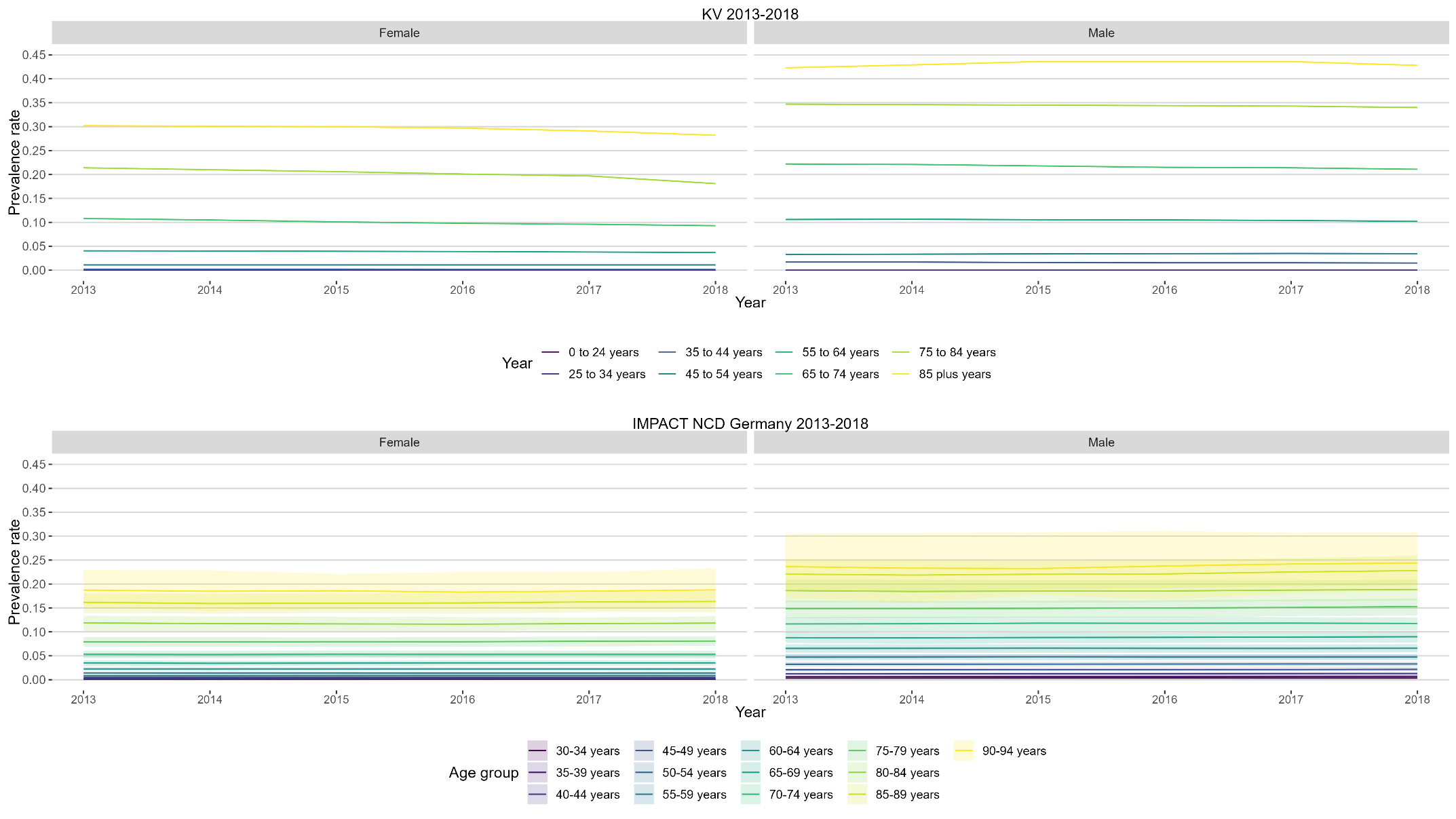
External validation comparison of the prevalence of coronary heart disease (CHD) from the German *Kassenärztliche Vereinigung* (KV) ambulatory claims data across the statuatory health insurance (i.e., diagnosis prevalence) 2013 to 2018 and IMPACT_NCD_ Germany by sex and age group [84]. Shaded areas indicate 95% uncertainty intervals.

Figure AH: External validation of the simulated stroke prevalence in 2014 with the GEDA survey by sex and age group


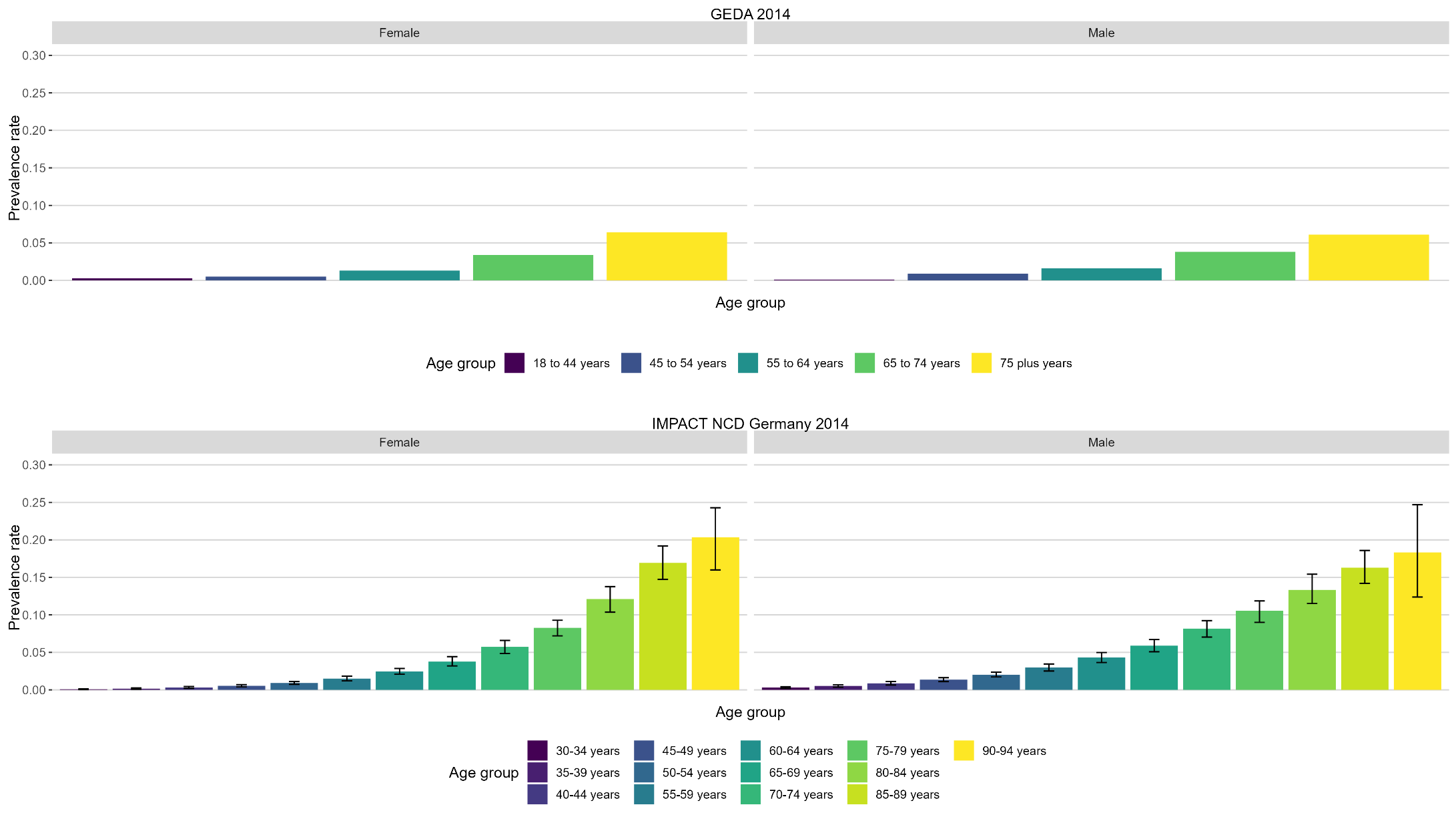
External validation comparison of the prevalence of stroke from the German *Gesundheit in Deutschland Aktuell* (GEDA) telephone survey 2014 and IMPACT_NCD_ Germany by sex and age group [81]. Vertical error bars indicate 95% uncertainty intervals.

Figure AI: External validation of the simulated stroke prevalence in 2019 with the GEDA survey by sex and age group


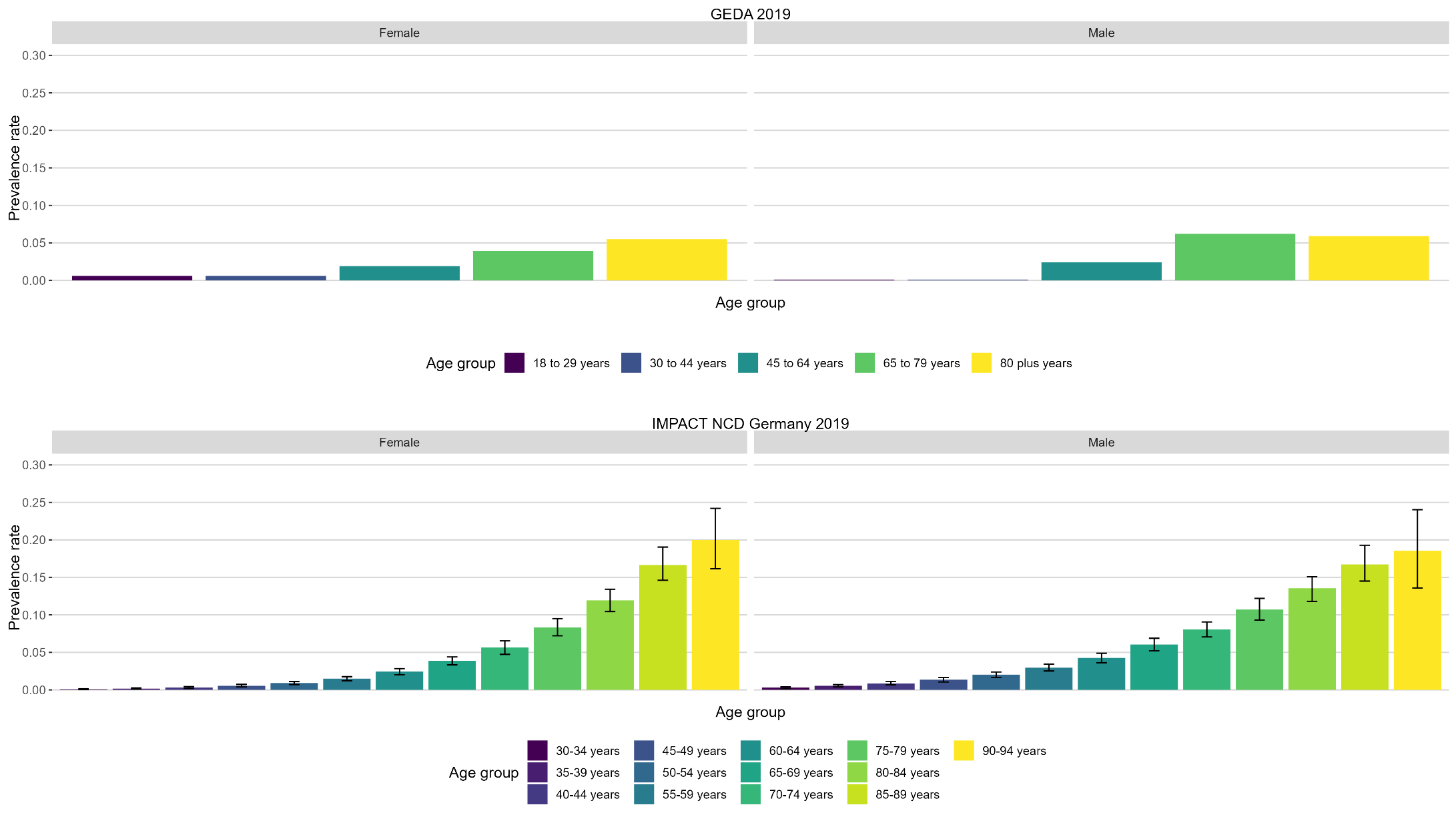
External validation comparison of the prevalence of stroke from the German *Gesundheit in Deutschland Aktuell* (GEDA) telephone survey 2019 and IMPACT_NCD_ Germany by sex and age group [83]. Vertical error bars indicate 95% uncertainty intervals.

Figure AJ: External validation of the simulated stroke prevalence in 2013 with AOK data from 2011 by sex and age group*


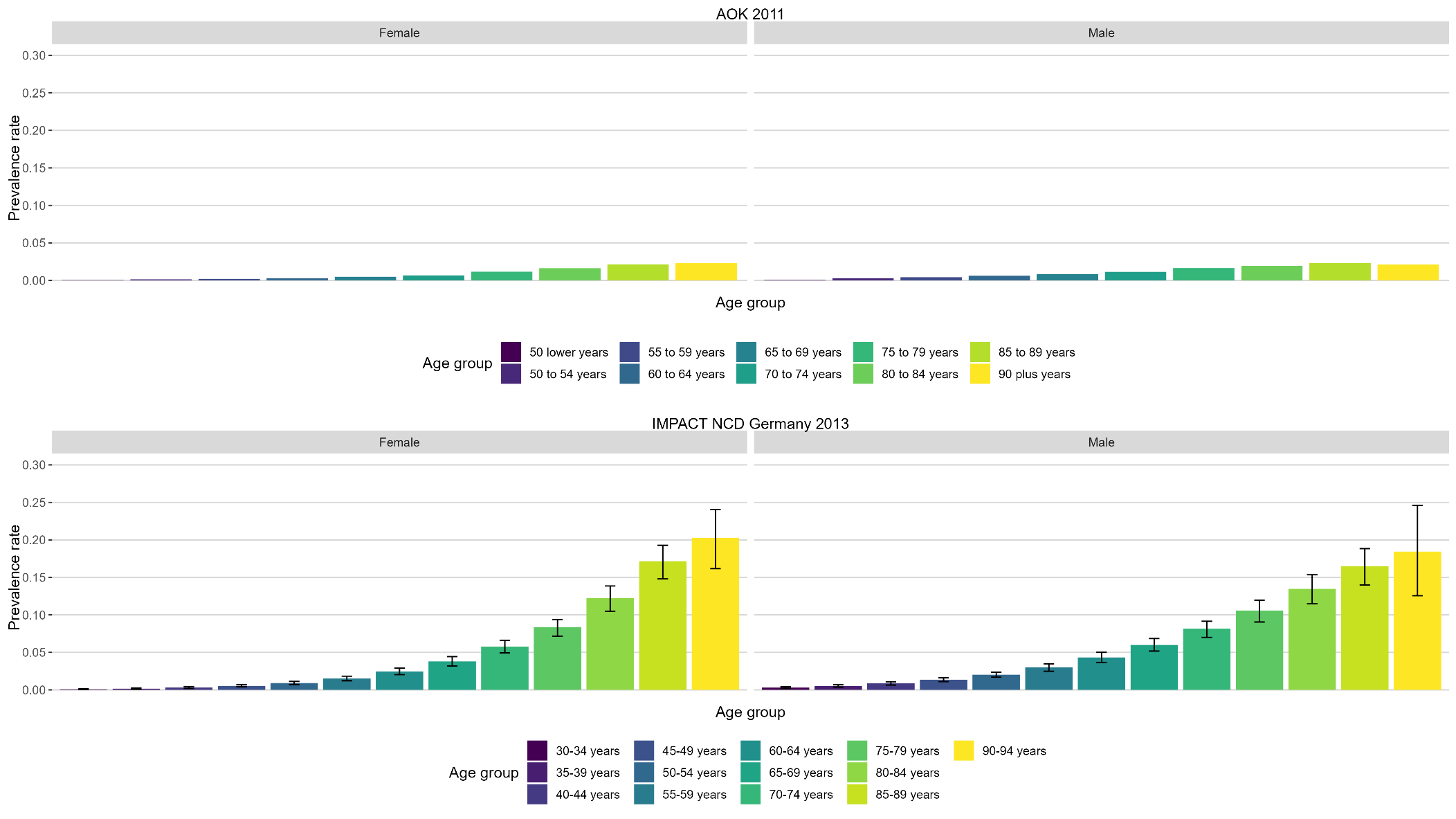
External validation comparison of the prevalence of stroke from a study using data of one statuatory health insurance Allgemeine Ortskrankenkasse (AOK) and IMPACT_NCD_ Germany by sex and age group [85]. Vertical error bars indicate 95% uncertainty intervals. *The prevalence data in the study that is used for comparison is based on stroke diagnoses based on AOK health claims data in 2011. This leads to stroke prevalence and incidence being practically identical in this data. Other external sources, such as the *Gesundheit in Deutschland Aktuell* (GEDA) survey, account for chronic disability due to stroke in their indicator and are therefore much closer to our simulation.

Figure AK: External validation of the simulated stroke incidence in 2013 with AOK data from 2011 by sex and age group


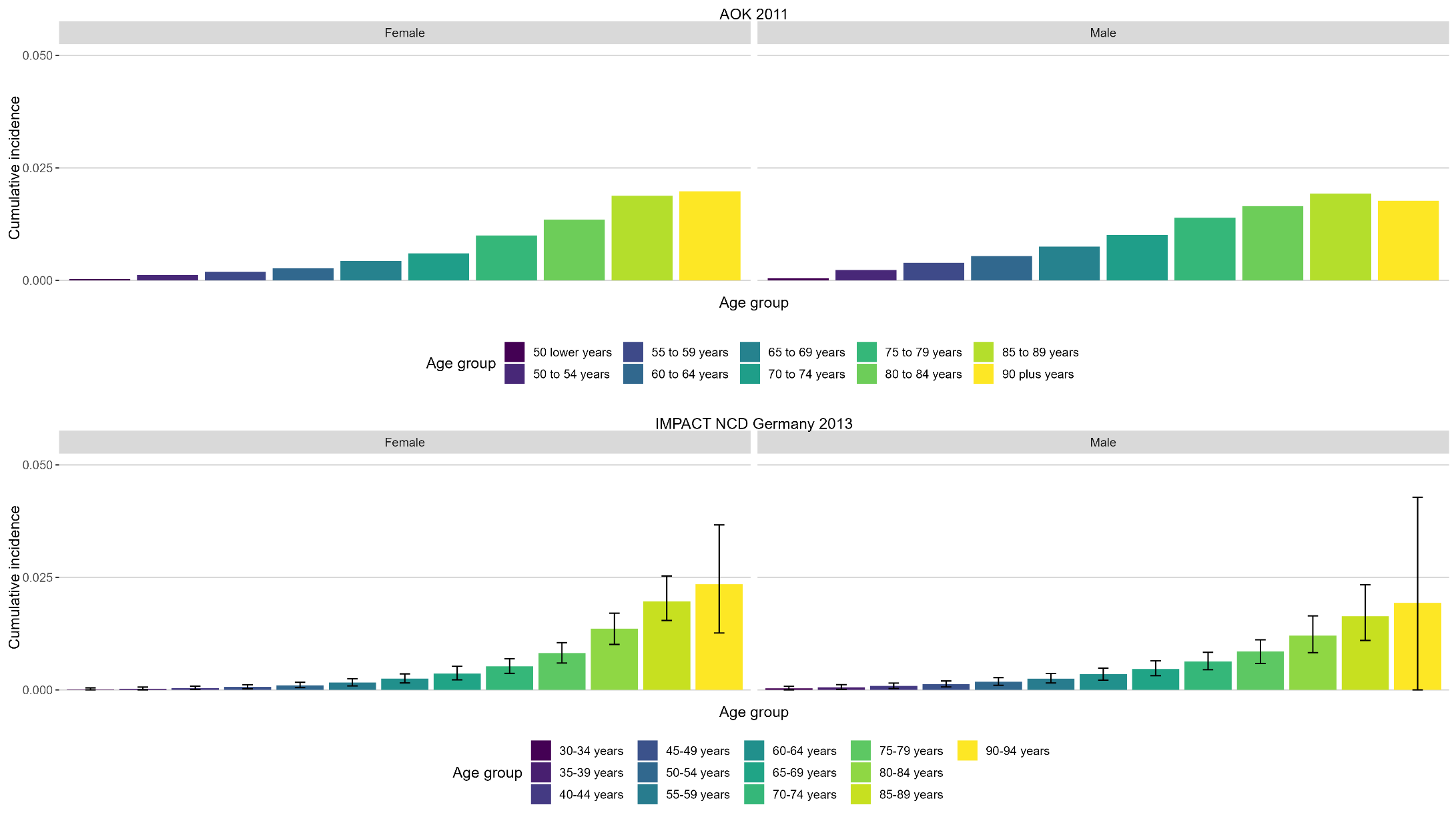
External validation comparison of the cumulative incidence of stroke from a study using data of one statuatory health insurance Allgemeine Ortskrankenkasse (AOK) and IMPACT_NCD_ Germany by sex and age group [85]. Vertical error bars indicate 95% uncertainty intervals.

Figure AL: External validation of the simulated type 2 diabetes prevalence in 2014 with the GEDA survey by sex and age group


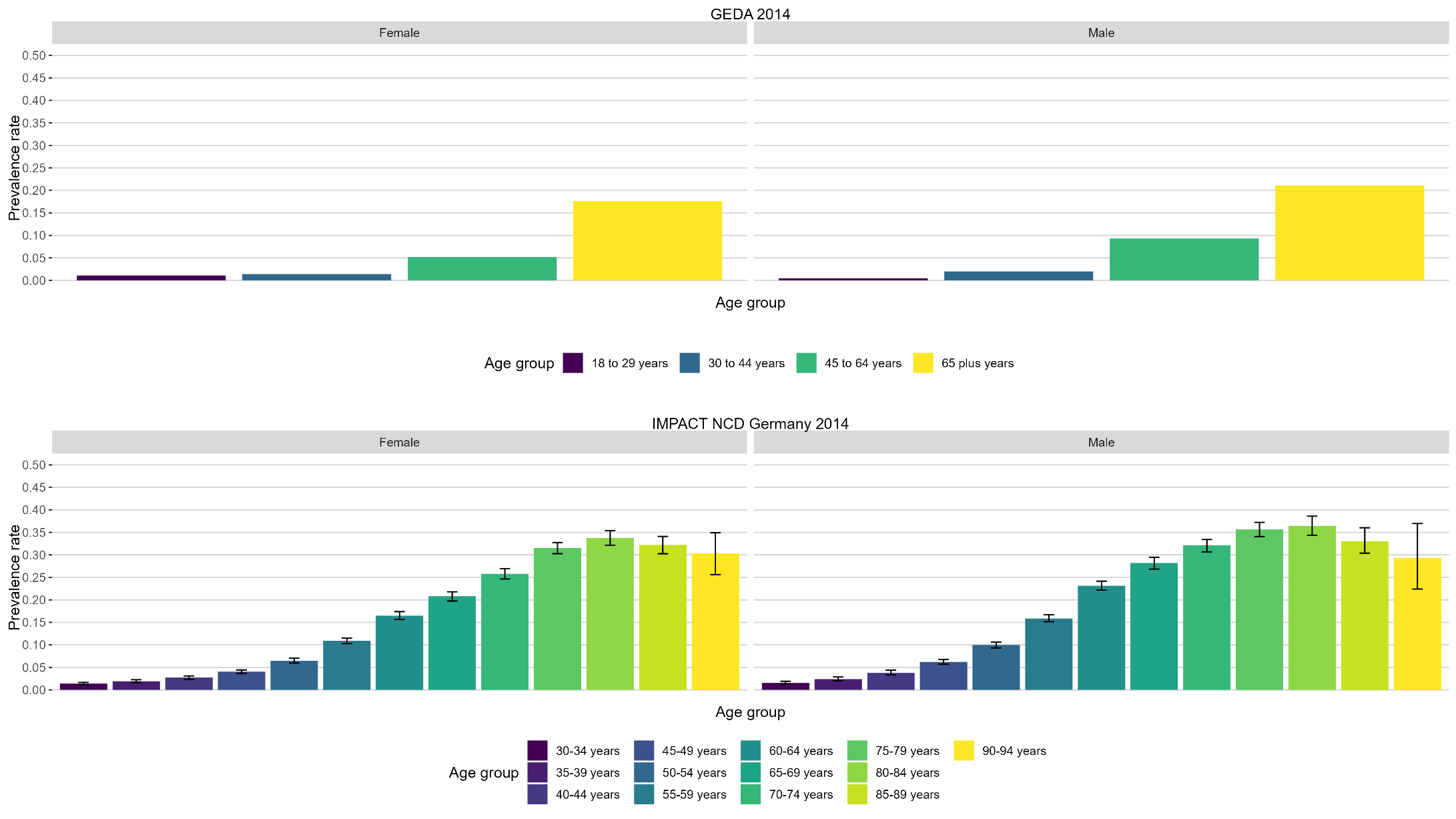
 External validation comparison of the prevalence of type 2 diabetes from the German *Gesundheit in Deutschland Aktuell* (GEDA) telephone survey 2014 and IMPACT_NCD_ Germany by sex and age group [82]. Vertical error bars indicate 95% uncertainty intervals.

Figure AM: External validation of the simulated type 2 diabetes prevalence in 2019 with the GEDA survey by sex and age group


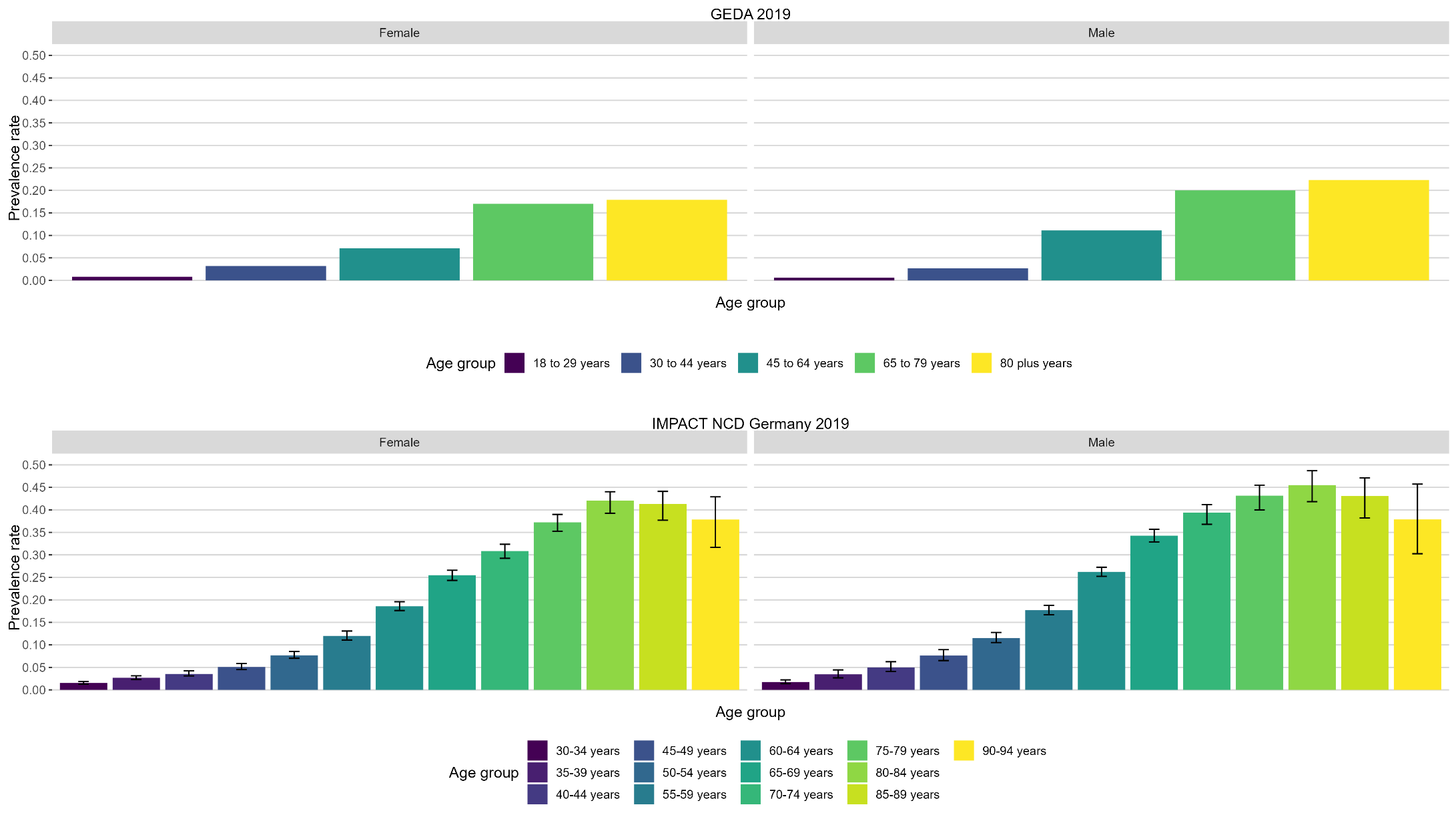
 External validation comparison of the prevalence of type 2 diabetes from the German *Gesundheit in Deutschland Aktuell* (GEDA) telephone survey 2019 and IMPACT_NCD_ Germany by sex and age group [83]. Vertical error bars indicate 95% uncertainty intervals.

# Methods B: Overview of the PRIMEtime model

PRIMEtime is a proportional multi-state life table Markov cohort model, which has, together which its predecessors, been applied in multiple evaluations of preventive non-communicable disease (NCD) policies and population health modeling studies around the globe [31, 86-90]. The modeling methods, centered around the integration of multi-morbidity in life tables, estimating reductions in disease incidence through population impact fractions, are implemented in Microsoft Excel and have been described extensively in previous methodological publications and will thus not be described in detail here [19, 91-95]. In the following paragraph, we give a brief overview of the model.

As mentioned, PRIMEtime is a cohort model which in our case is stratified by 5-year age groups and sex as demographic characteristics. The impact of a change in the modelled risk factors on disease incidence is estimated using population impact fractions [95]. In our study, body mass index (BMI) is the only modelled risk factor, and the modelled diseases are coronary heart disease (CHD), stroke, and type 2 diabetes (T2DM). The impact on disease incidence is then integrated in independent multi-state Markov models for each of the modelled diseases, which consist of the three exclusive states “healthy”, “diseased”, and “dead” [19]. For each disease, the proportion of the population in each state is tracked over time governed by epidemiological parameters such as incidence, prevalence, and case fatality. Finally, the disease-specific results are integrated in an overall lifetable, which enables the estimation of the total disease burden with regards to quality-adjusted life years (QALYs) or disability-adjusted life years (DALYs). While diseases are generally assumed to be independent of each other, the model allows the incidence of CHD and stroke to be adjusted based on the T2DM prevalence rate in the respective cohort. In the model, time lags between risk factors and diseases and risk factor trends can be considered as well. The modelled policy scenarios, which each have an impact on the modelled risk factors (i.e., only BMI in our case) can thus be compared to a counterfactual baseline scenario without any policy intervention where disease incidence is not impacted via these risk factors. Scenario analyses can be both done using an open cohort approach over a specific time horizon or following the starting cohorts over their lifetime [96]. Parametric uncertainty can be assessed by Monte Carlo analysis incorporating disease-risk factor relative risks, health utility values, health care costs and, if available, intervention effect.

# Methods C: Simulation model cross-validation

## Overview of cross-validation approach

In the following sections we will describe the approach we used to cross-validate IMPACT_NCD_ Germany with PRIMEtime. We first introduce the concept of cross-validation and then provide details on our rationale.

Generally, cross-validation is of interest to ensure the internal, to some extent external, and specifically the operational validity of any model. With all statistical models, including classic regression or machine learning models, questions about the validity of produced estimates arise [97]. One particular question is whether estimates and their resulting predictions are influenced by the specific dataset (or input data) used in their estimation and if they generalize well to an independent dataset that was not used in the model fitting. If so, the corresponding model seems to capture the data generating process well. Cross-validation procedures try to achieve this generalizability by producing estimates based on different subsets of the main dataset (the training data) and predicting outcomes in the remainder of the main same dataset (the test data). Model performance can be assessed by comparing both predicted outcomes based on the trained estimates and true outcomes in the test data [97].

In the typical case of population health simulation modelling, this setup is not possible because no information on true outcomes is available, we do not have typical effect estimates to compare, and no single dataset is used. However, we can conceptually try something similar by varying the simulation process instead of the input data and comparing the resulting outputs. In our case outputs are health impacts. Therefore cross-validation in the case of simulation modelling means to what extent the simulated model outcomes are the same when analysing the same problem (i.e., sugar-sweetened beverage [SSB] taxation) using the same input data with two (or more) independently developed simulation models with the same key ingredients but a different structure and modelling approach [98, 99].

As mentioned above, in this study we cross-validated the health impacts of sugar-sweetened beverage (SSB) taxation in Germany simulated by IMPACT_NCD_ Germany with the health impacts of SSB taxation in Germany simulated by PRIMEtime. The outcomes that we used for validation (validation targets) are coronary heart disease (CHD), stroke and type 2 diabetes (T2DM) cases prevented/postponed and quality-adjusted life years (QALYs) gained. While IMPACT_NCD_ Germany and PRIMEtime are both population health models, their implementation and related modelling decisions vary. As part of the cross-validation procedure, our goal was to make both models as similar as possible without changing the original model structure. This particularly relates to the used input data, the implementation of policy scenarios and how outcomes are aggregated. In the following sections we describe all steps taken for the cross-validation in detail.

## Estimation of input data for PRIMEtime

### Demographics and disease epidemiology

We populated the PRIMEtime model with the same population demographic characteristics as IMPACT_NCD_ Germany. This includes population size estimates stratified by age and sex and population projections needed for the open cohort approach from the input data tables used for IMPACT_NCD_ Germany. Similarly, we directly implemented the processed prevalence, incidence, case fatality and mortality values for CHD, stroke and T2DM used in IMPACT_NCD_ Germany. Disease trends (i.e., annual decline disease-specific incidence) in PRIMEtime were also directly informed by trends implemented in IMPACT_NCD_ Germany.

### Exposure distributions from synthetic population

Since PRIMEtime is a cohort model, the population in each age-sex cohort is assumed to have the same risk factor profile. To ensure consistency between both models, we computed the mean and standard deviation for each risk factor per age-sex cohort and, if possible, year using individual information from the generated synthetic population. Thus, risk factor trends in PRIMEtime were based on the actual trends in IMPACT_NCD_ Germany. To ensure population representativeness, we applied the respective population weights.

As described above, body mass index (BMI) is the only risk factor that is modelled in the version of PRIMEtime we used. However, information on cohort-specific consumption of SSBs, fruit juice and sugar from beverage is needed to implement the SSB taxation policy scenarios in the same way as in IMPACT_NCD_ Germany. We therefore implemented the cohort-specific BMI values and time trends from the synthetic German population in PRIMEtime and further extended the input data by cohort-specific values for beverage and sugar consumption.

### Health economics

Due to the detailed modelling of individual economic impacts in IMPACT_NCD_ Germany, we decided that a comparable implementation in PRIMEtime was beyond the scope of this cross-validation procedure. However, we implemented the re-estimated German health utility weights (see section Health Economics module in **Methods A**) in PRIMEtime as well. This enabled us the calculation and comparison of QALYs for both models. In accordance with the main analyses, we set the discount rate for QALYs to 3%.

### Implementation of policy scenarios

Policy scenarios in PRIMEtime are implemented in the same way as in IMPACT_NCD_ Germany. However, the estimated reduction in sugar from beverages is based on the average values in the respective age-sex cohort. Otherwise, scenarios were programmed with the same assumptions, including lag times between reduced sugar consumption and impacts on BMI (see section Policy module in **Methods A**).

## Modifications of the PRIMEtime model structure

To ensure that model outcomes in the cross-validation procedure were comparable and due to how some specific aspects were implemented in both IMPACT_NCD_ Germany and PRIMEtime, we slightly adjusted the structure of PRIMEtime in several aspects:

1. We adjusted how the health utility decrement associated with different BMI and age values is implemented. Originally this was based on BMI and age categories in PRIMEtime but continuous in IMPACT_NCD_ Germany.
2. We included the estimate of the long-term effect of sugar consumption on BMI in the probabilistic uncertainty framework of PRIMEtime.
3. We included the own- and cross-price elasticities of SSBs and fruit juice and the estimated tax pass-through in the probabilistic uncertainty framework of PRIMEtime.
4. We adjusted how disease outcomes, such as cases prevented/postponed are counted within the open cohort framework of PRIMEtime to be consistent with IMPACT_NCD_ Germany.
5. We included the relative risk estimates used to model the relationship between risk factors and diseases in IMPACT_NCD_ Germany in PRIMEtime. A comparison of the estimated health impact and respective difference between PRIMEtime’s original risk parameterisation and the one used in IMPACT_NCD_ Germany is shown in **Figures AN & AO**.

Albeit we aimed to make both models as comparable as possible, some differences remain. For example, PRIMEtime does not include future trends in all-cause mortality and disease-specific mortality is calibrated manually in IMPACT_NCD_ Germany, while general trends are applied in PRIMEtime. Additionally, IMPACT_NCD_ Germany accounts for uncertainty in all epidemiological parameters, which PRIMEtime does not. Remaining outcome differences between both models might thus be explained by small remaining differences in certain assumptions or modelling decisions. Resolving these was beyond the scope of this study and can be seen as part of the structural uncertainty arising from different simulation models and their underlying model structures.

Figure AN: Cross-validation with different sets of relative risks


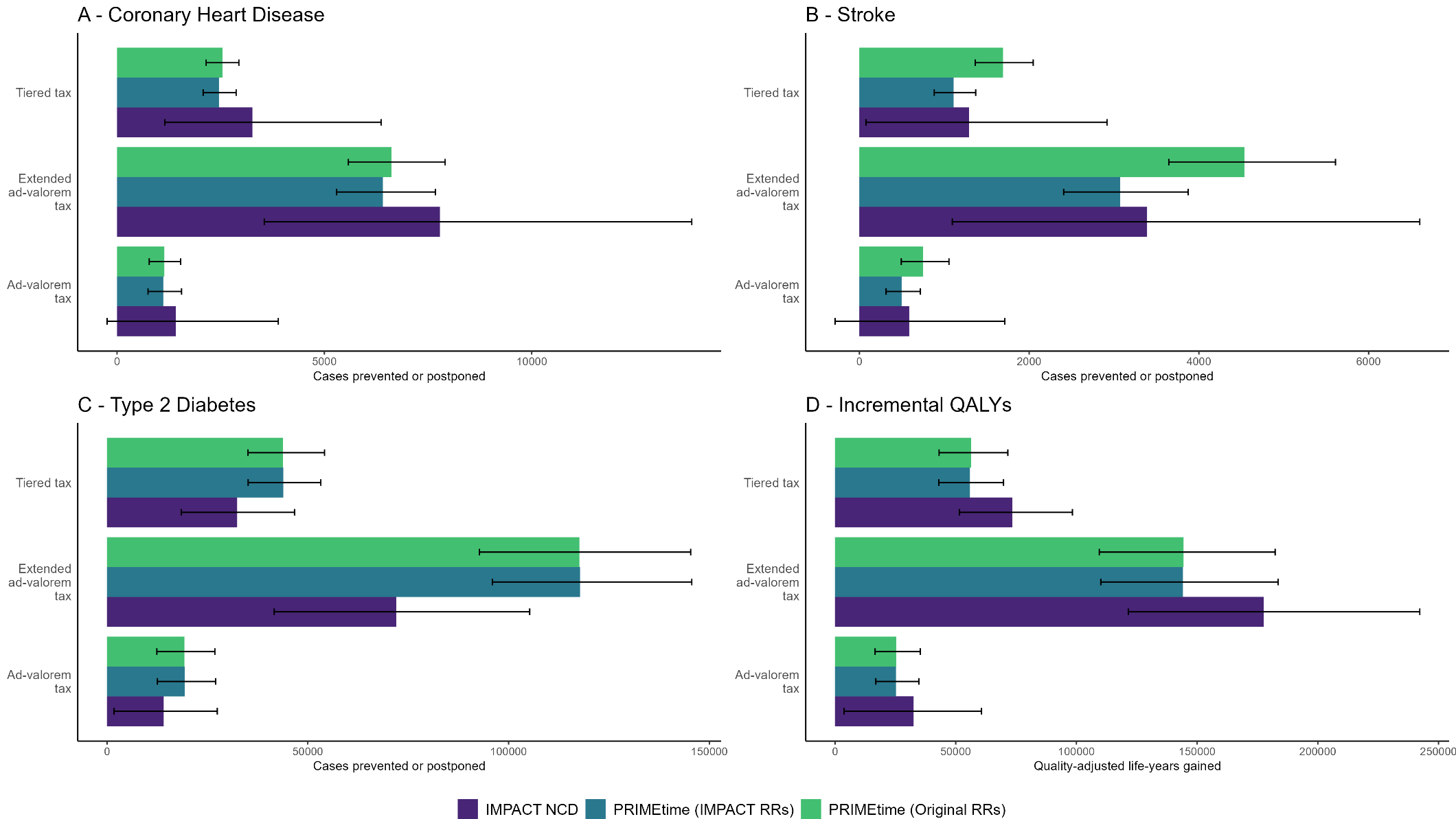


Horizontal bar chart comparing cross-validation outcomes for A) coronary heart disease cases prevented/postponed, B) stroke cases prevented/postponed, C) type 2 diabetes cases prevented/postponed, and D) QALYs gained between the IMPACT_NCD_ microsimulation (purple) and the PRIMEtime cohort model with its original relative risk (RR) parameterisation (green) and updated RR parameterisation to improve consistency with IMPACT_NCD_ (turquoise). Only BMI-mediated exposure pathways are modelled. Error bars indicate 95%-uncertainty intervals. “Ad-valorem tax” refers to a 20% ad-valorem tax on SSBs with a pass-through to consumers of 82% (for details see section “Sugar-sweetened beverage taxation scenarios” in the main text). “Extended ad-valorem tax” refers to a 20% ad-valorem tax on SSBs and fruit juice with a pass-through to consumers of 82% (for details see section “Sugar-sweetened beverage taxation scenarios” in the main text). “Tiered tax” refers to a tiered tax on SSBs similar to the United Kingdom Soft Drinks Industry Levy that leads to a reduction in SSB sugar content by 30% through reformulation (for details see section “Sugar-sweetened beverage taxation scenarios” in the main text). Error bars indicate 95%-confidence intervals. Abbreviations: BMI, body mass index; NCD, non-communicable disease; QALY, quality-adjusted life year; SSB, sugar-sweetened beverages.

Figure AO: Cross-validation with different sets of relative risks stratified by sex


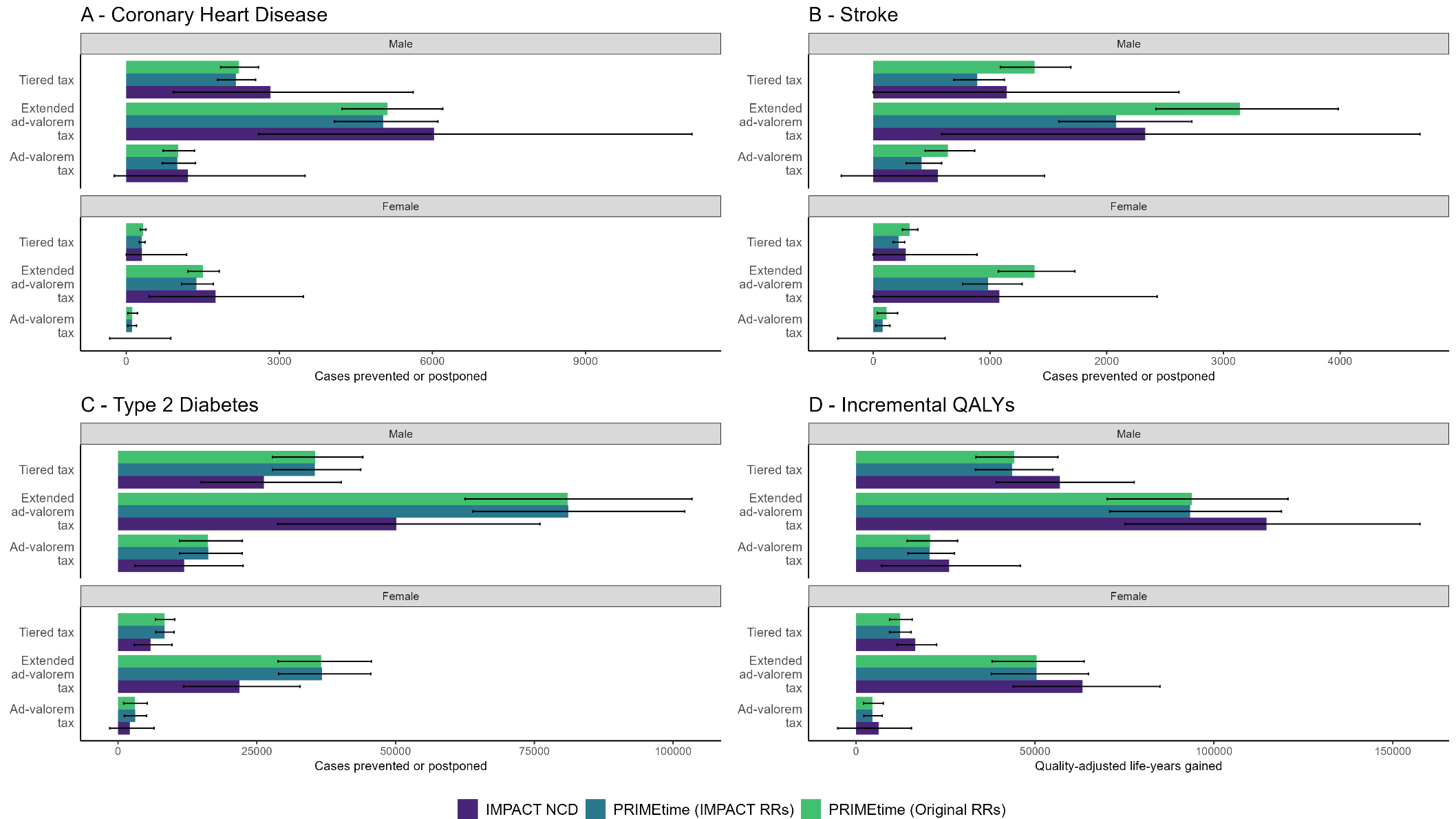


Horizontal bar chart comparing cross-validation outcomes for A) coronary heart disease cases prevented/postponed, B) stroke cases prevented/postponed, C) type 2 diabetes cases prevented/postponed, and D) QALYs gained between the IMPACT_NCD_ microsimulation (purple) and the PRIMEtime cohort model with its original relative risk (RR) parameterisation (green) and updated RR parameterisation to improve consistency with IMPACT_NCD_ (turquoise) by sex. Only BMI-mediated exposure pathways are modelled. Error bars indicate 95%-uncertainty intervals. “Ad-valorem tax” refers to a 20% ad-valorem tax on SSBs with a pass-through to consumers of 82% (for details see section “Sugar-sweetened beverage taxation scenarios” in the main text). “Extended ad-valorem tax” refers to a 20% ad-valorem tax on SSBs and fruit juice with a pass-through to consumers of 82% (for details see section “Sugar-sweetened beverage taxation scenarios” in the main text). “Tiered tax” refers to a tiered tax on SSBs similar to the United Kingdom Soft Drinks Industry Levy that leads to a reduction in SSB sugar content by 30% through reformulation (for details see section “Sugar-sweetened beverage taxation scenarios” in the main text). Error bars indicate 95%-confidence intervals. Abbreviations: BMI, body mass index; NCD, non-communicable disease; QALY, quality-adjusted life year; SSB, sugar-sweetened beverages.

Figure AP: Cumulative cases prevented or postponed over time by scenario and sex


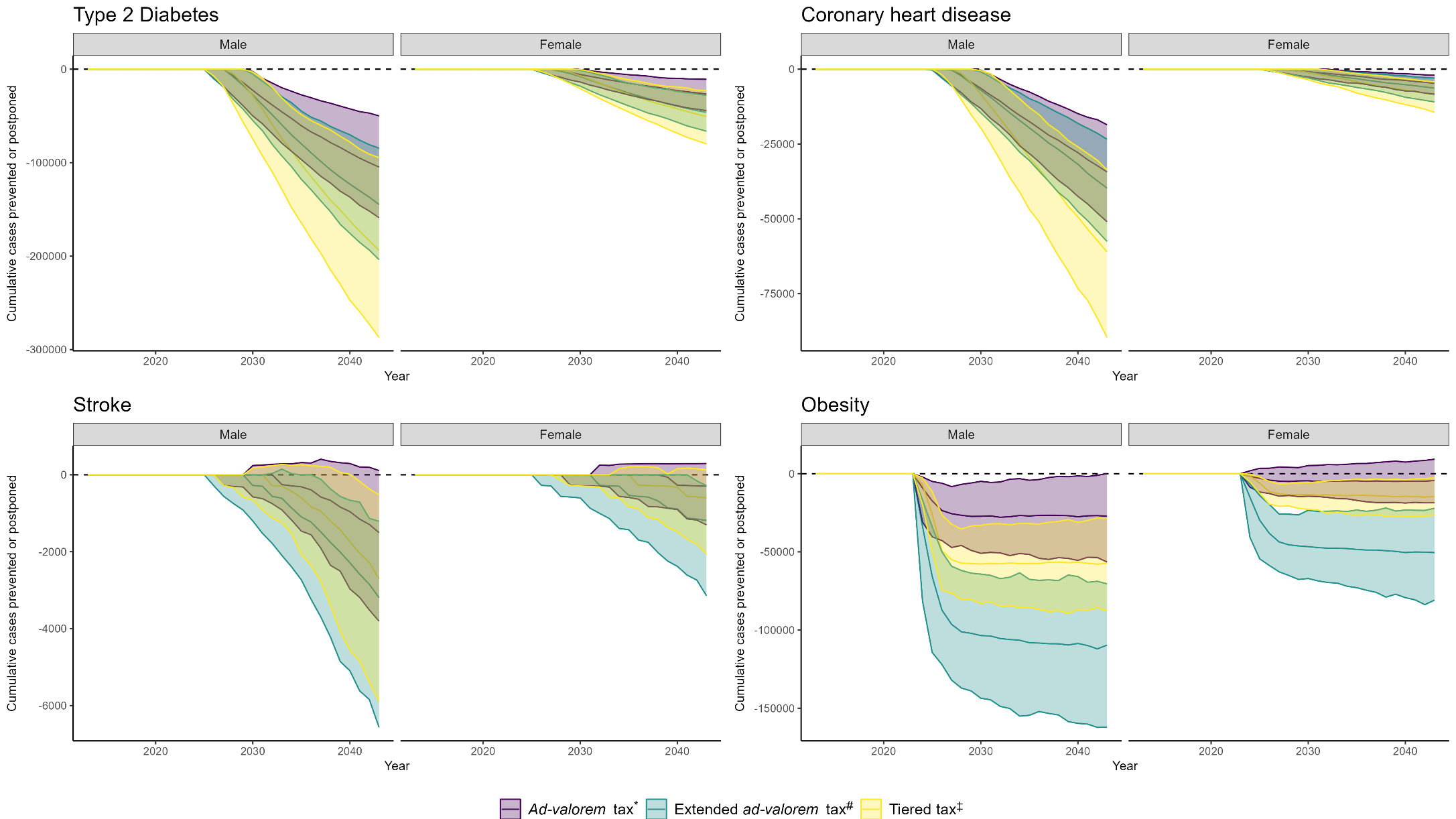


Line plots of disease-specific median cumulative cases prevented or postponed from 2013 to 2043 as a consequence of sugar-sweetened beverage (SSB) taxation scenarios stratified by sex. Shaded areas indicate 95%-confidence intervals. *“Ad-valorem tax” refers to a 20% ad-valorem tax on SSBs with a pass-through to consumers of 82% (for details see section “Sugar-sweetened beverage taxation scenarios” in the main text). ^#^“Extended ad-valorem tax” refers to a 20% ad-valorem tax on SSBs and fruit juice with a pass-through to consumers of 82% (for details see section “Sugar-sweetened beverage taxation scenarios” in the main text). ^‡^“Tiered tax” refers to a tiered tax on SSBs similar to the United Kingdom Soft Drinks Industry Levy that leads to a reduction in SSB sugar content by 30% through reformulation (for details see section “Sugar-sweetened beverage taxation scenarios” in the main text).

Figure AQ: Cumulative case-years prevented or postponed over time by scenario and sex


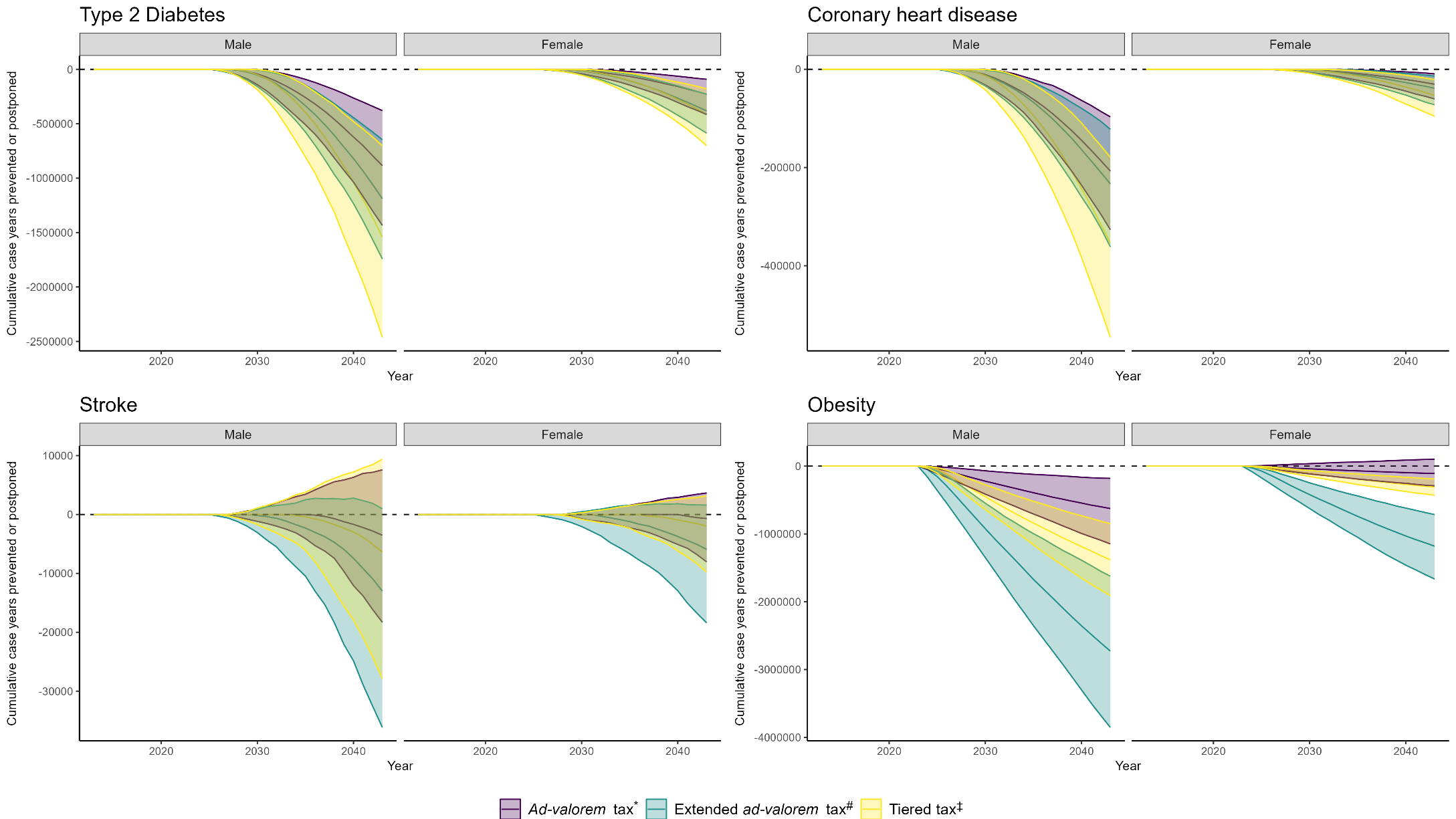


Line plots of disease-specific median cumulative case-years prevented or postponed from 2013 to 2043 as a consequence of sugar-sweetened beverage (SSB) taxation scenarios stratified by sex. Shaded areas indicate 95%-confidence intervals. *“Ad-valorem tax” refers to a 20% ad-valorem tax on SSBs with a pass-through to consumers of 82% (for details see section “Sugar-sweetened beverage taxation scenarios” in the main text). ^#^“Extended ad-valorem tax” refers to a 20% ad-valorem tax on SSBs and fruit juice with a pass-through to consumers of 82% (for details see section “Sugar-sweetened beverage taxation scenarios” in the main text). ^‡^“Tiered tax” refers to a tiered tax on SSBs similar to the United Kingdom Soft Drinks Industry Levy that leads to a reduction in SSB sugar content by 30% through reformulation (for details see section “Sugar-sweetened beverage taxation scenarios” in the main text).

Table U: Change in exposures compared to baseline by scenario, age group and sex

| **Exposure** | **Difference to baseline in 2043 (95%-uncertainty intervals)** | | |
| --- | --- | --- | --- |
|  | *Ad-valorem* tax* | Extended *ad-valorem* tax^#^ | Tiered tax^‡^ |
| **Mean BMI (kg/m²)** |  |  |  |
| Male |  |  |  |
| Age 30-49 years | -0.03 (-0.04, -0.01) | -0.10 (-0.12, -0.06) | -0.06 (-0.08, -0.04) |
| Age 50-69 years | -0.01 (-0.02, 0.00) | -0.06 (-0.08, -0.04) | -0.03 (-0.04, -0.01) |
| Age 70-90 years | 0.00 (-0.01, 0.00) | -0.04 (-0.05, -0.02) | -0.01 (-0.02, -0.01) |
| Female |  |  |  |
| Age 30-49 years | -0.01 (-0.01, 0.00) | -0.04 (-0.06, -0.03) | -0.01 (-0.02, -0.01) |
| Age 50-69 years | 0.00 (-0.01, 0.00) | -0.04 (-0.05, -0.03) | -0.01 (-0.01, -0.01) |
| Age 70-90 years | 0.00 (-0.01, 0.00) | -0.03 (-0.04, -0.02) | -0.01 (-0.01, 0.00) |
| *Total* | -0.01 (-0.02, 0.00) | -0.05 (-0.06, -0.03) | -0.02 (-0.03, -0.01) |
| **Mean SSB (ml/day)** |  |  |  |
| Male |  |  |  |
| Age 30-49 years | -30.34 (-31.05, -28.63) | -30.34 (-31.05, -28.63) | 0.00 (0.00, 0.00)^†^ |
| Age 50-69 years | -14.87 (-15.29, -13.91) | -14.87 (-15.29, -13.91) | 0.00 (0.00, 0.00)^†^ |
| Age 70-90 years | -6.99 (-7.19, -6.56) | -6.98 (-7.19, -6.56) | 0.00 (0.00, 0.00)^†^ |
| Female |  |  |  |
| Age 30-49 years | -11.13 (-11.38, -10.44) | -11.13 (-11.38, -10.44) | 0.00 (0.00, 0.00^†^ |
| Age 50-69 years | -6.27 (-6.40, -5.88) | -6.27 (-6.40, -5.88) | 0.00 (0.00, 0.00^†^ |
| Age 70-90 years | -3.50 (-3.60, -3.27) | -3.50 (-3.60, -3.27) | 0.00 (0.00, 0.00)^†^ |
| *Total* | -11.66 (-11.85, -10.95) | -11.66 (-11.85, -10.95) | 0.00 (0.00, 0.00)^†^ |
| **Mean fruit juice (ml/day)** |  |  |  |
| Male |  |  |  |
| Age 30-49 years | 2.09 (-4.14, 10.48) | -45.09 (-46.50, -40.41) | 0.00 (-0.02, 0.06) |
| Age 50-69 years | 1.58 (-3.12, 7.90) | -33.86 (-34.95, -30.38) | 0.02 (-0.04, 0.13) |
| Age 70-90 years | 1.01 (-1.94, 5.05) | -21.63 (-22.32, -19.31) | 0.01 (-0.03, 0.06) |
| Female |  |  |  |
| Age 30-49 years | 1.73 (-3.42, 8.71) | -37.20 (-38.39, -33.46) | 0.00 (0.00, 0.01) |
| Age 50-69 years | 1.36 (-2.68, 6.76) | -29.10 (-30.01, -26.08) | 0.00 (-0.01, 0.03) |
| Age 70-90 years | 0.89 (-1.76, 4.51) | -19.23 (-19.91, -17.27) | 0.00 (-0.01, 0.02) |
| *Total* | 1.41 (-2.76, 7.04) | -30.22 (-30.97, -27.21) | 0.01 (-0.01, 0.03) |

*“Ad-valorem tax” refers to a 20% ad-valorem tax on SSBs with a pass-through to consumers of 82% (for details see section “Sugar-sweetened beverage taxation scenarios” in the main text). ^#^“Extended ad-valorem tax” refers to a 20% ad-valorem tax on SSBs and fruit juice with a pass-through to consumers of 82% (for details see section “Sugar-sweetened beverage taxation scenarios” in the main text). ^‡^“Tiered tax” refers to a tiered tax on SSBs similar to the United Kingdom Soft Drinks Industry Levy that leads to a reduction in SSB sugar content by 30% through reformulation (for details see section “Sugar-sweetened beverage taxation scenarios” in the main text). ^†^Technically consumption does change in the model for the tiered tax scenario to implement the direct effects of sugar-sweetened beverages (SSBs) in this scenario. See Policy module for details. Briefly, because the direct effects of SSBs are implemented per ml/day of SSB, the reduction in sugar content (g/day) in the tiered taxation scenario is translated into an equivalent consumption reduction effect based on an individual amount of sugar per SSB (g/ml). This is justified because it is assumed that the direct effects of SSBs are due to their sugar content [100]. Abbreviations: BMI, body mass index; g, grams; m², square metre; ml, millilitre.

Table V: Health and economic impact of different sensitivity analyses I

| ***Health outcomes*** | **Change in outcomes compared to baseline without tax (95%-uncertainty intervals)** | | |
| --- | --- | --- | --- |
|  | *Sensitivity analysis 1: Ad-valorem tax with 10% tax rate* | *Sensitivity analysis 2: Ad-valorem tax with 30% tax rate* | *Sensitivity analysis 3: Ad-valorem tax without substitution to fruit juice* |
| Cases prevented/postponed* |  |  |  |
| T2DM | 66,800 (30,400, 102,700) | 196,400 (90,100, 304,200) | 134,700 (65,400, 204,400) |
| CHD | 20,200 (10,400, 30,000) | 58,600 (31,100, 86,800) | 39,800 (21,900, 58,800) |
| Stroke | 900 (-300, 2,600) | 2,700 (200, 6,900) | 2,000 (200, 4,600) |
| Obesity | 16,900 (-2,600, 38,500) | 43,800 (-11,300, 97,000) | 40,900 (20,900, 67,200) |
| Case-years prevented/postponed* |  |  |  |
| T2DM | 564,700 (237,200, 922,800) | 1,654,200 (701,200, 2,709,000) | 1,138,900 (517,800, 1,852,300) |
| CHD | 119,800 (52,100, 190,500) | 351,400 (173,000, 550,400) | 241,900 (118,600, 376,000) |
| Stroke | 2,200 (-6,900, 13,500) | 6,700 (-9,100, 30,900) | 5,200 (-6,100, 22,800) |
| Obesity | 371,400 (45,300, 720,900) | 1,099,200 (140,500, 2,142,400) | 904,100 (539,800, 1,286,800) |
| All-cause deaths prevented/postponed | 8,700 (4,000, 13,900) | 25,100 (13,300, 39,100) | 17,400 (8,800, 26,500) |
| QALYs gained | 53,800 (28,600, 78,000) | 157,200 (87,000, 225,200) | 112,500 (75,400, 153,000) |
| Life years gained | 49,000 (22,500, 84,000) | 139,900 (74,000, 234,100) | 96,100 (48,600, 162,000) |
| Difference in life expectancy | 0.01 (-0.01, 0.04) | 0.02 (-0.01, 0.07) | 0.02 (-0.01, 0.06) |
| Difference in life expectancy at age 60 years | 0.00 (-0.01, 0.02) | 0.01 (-0.01, 0.03) | 0.01 (-0.01, 0.02) |
| ***Health-related cost outcomes (€-millions)*** |  |  |  |
| **Healthcare costs** |  |  |  |
| T2DM | -820 (-1,373 to -341) | -2401 (-4,057 to -996) | -1,664 (-2,777 to -722) |
| CHD | -334 (-511 to -167) | -984 (-1,450 to -512) | -666 (-998 to -355) |
| Stroke | -43 (-123 to 18) | -132 (-333 to -9) | -93 (-230 to -14) |
| Other | 61 (27 to 107) | 174 (89 to 302) | 119 (60 to 206) |
| **Productivity costs** |  |  |  |
| T2DM early retirement | -12 (-32 to 4) | -35 (-88 to 7) | -24 (-60 to 4) |
| T2DM sick leave | -591 (-,1380 to -201) | -1,718 (-4,186 to -556) | -1,196 (-2,781 to -387) |
| Stroke early retirement | -1 (-17 to 8) | -2 (-56 to 5) | -1 (-34 to 5) |
| Stroke sick leave | 0 (-1 to 0) | 0 (-2 to 0) | 0 (-1 to 0) |
| Premature death | -1,820 (-3,930 to -525) | -5,194 (-9,937 to -2,012) | -3,586 (-7,135 to -1,325) |
| **Time costs** |  |  |  |
| T2DM self-management | -581 (-1,051 to -235) | -1,692 (-2,994 to -705) | -1,159 (-2,024 to -506) |
| T2DM time for health service use | -733 (-1,814 to -249) | -2,139 (-5,178 to -756) | -1,482 (-3,506 to -524) |
| Other time for health service use | 187 (82 to 329) | 548 (232 to 960) | 379 (159 to 660) |
| ***Cost-effectiveness*** |  |  |  |
| Total change in costs from healthcare perspective (€-millions) | -1,124 (-1,800 to -585) | -3,331 (-5,296 to -1,732) | -2,306 (-3,616 to -1,237) |
| Total change in costs from societal perspective (€-millions) | -4,849 (-7,930 to -2,355) | -13,981 (-22,492 to -6,722) | -9,689 (-15,387 to -4,909) |
| ICER^#^ (healthcare perspective) | Dominant | Dominant | Dominant |
| ICER^#^ (societal perspective) | Dominant | Dominant | Dominant |

*Cases and case-years prevented/postponed are defined as incident and prevalent cases completely prevented or delayed for one or more years, respectively. ^#^Expressed as € per QALY. Only defined for positive incremental costs and else “dominant” because no trade-off exists if health is improved while costs are saved. Abbreviations: CHD, coronary heart disease; CVD, cardiovascular disease; ICER, incremental cost-effectiveness ratio; QALY, quality-adjusted life year; SSB, sugar-sweetened beverages; T2DM, type 2 diabetes mellitus.

Table W: Health and economic impact of different sensitivity analyses II

| ***Health outcomes*** | **Change in outcomes compared to baseline without tax (95%-uncertainty intervals)** | | | |
| --- | --- | --- | --- | --- |
|  | *Sensitivity analysis 4: Tiered tax with 10% reformulation* | *Sensitivity analysis 5: Ad-valorem tax with meta-analytic price elasticities* | *Sensitivity analysis 6: Reformulation based on voluntary industry commitments (2% per 6 years)* | *Sensitivity analysis 7: Maximum impact scenario with 30% reformulation and consumption reduction as under ad-valorem tax* |
| Cases prevented/postponed* |  |  |  |  |
| T2DM | 83,700 (40,200, 126,000) | 92,600 (40,900, 156,000) | 25,700 (11,700, 41,200) | 433,800 (204,000, 651,500) |
| CHD | 24,200 (12,500, 36,100) | 27,500 (12,600, 43,800) | 7,000 (3,300, 11,600) | 128,000 (71,600, 186,500) |
| Stroke | 1,200 (-100, 3,100) | 1,400 (0, 3,500) | 300 (-300, 1,200) | 5,700 (1,400, 12,600) |
| Obesity | 27,400 (11,800, 46,900) | 28,500 (10,900, 53,500) | 15,800 (5,100, 28,400) | 83,400 (40,000, 135,600) |
| Case-years prevented/postponed* |  |  |  |  |
| T2DM | 671,500 (293,800, 1,078,500) | 783,700 (299,000, 1,421,200) | 153,500 (64,900, 272,500) | 3,560,500 (1,574,100, 5,827,200) |
| CHD | 138,400 (61,500, 222,800) | 162,900 (64,100, 275,800) | 30,500 (10,300, 61,300) | 771,300 (396,600, 1,159,000) |
| Stroke | 2,700 (-5,800, 15,500) | 3,500 (-6,300, 18,100) | 0 (-3,200, 5,800) | 13,500 (-11,400, 53,500) |
| Obesity | 569,200 (342,200, 803,500) | 621,200 (305,200, 1,019,300) | 210,400 (126,300, 290,700) | 2,066,400 (1,125,000, 3,276,100) |
| All-cause deaths prevented/postponed | 10,200 (5,100, 16,200) | 12,000 (5,200, 20,200) | 2,500 (700, 5,100) | 52,800 (29,700, 79,600) |
| QALYs gained | 65,300 (43,400, 89,500) | 79,700 (40,200, 117,900) | 16,400 (10,800, 23,000) | 322,200 (210,100, 441,500) |
| Life years gained | 55,200 (25,800, 96,400) | 68,300 (27,900, 122,600) | 11,100 (2,700, 26,000) | 288,700 (156,200, 474,900) |
| Difference in life expectancy | 0.01 (-0.01, 0.04) | 0.01 (-0.01, 0.05) | 0.00 (-0.01, 0.02) | 0.05 (0.01, 0.13) |
| Difference in life expectancy at age 60 years | 0.00 (-0.01, 0.02) | 0.00 (-0.01, 0.02) | 0.00 (0.00, 0.01) | 0.02 (0.00, 0.06) |
| ***Health-related cost outcomes (€-millions)*** |  |  |  |  |
| **Healthcare costs** |  |  |  |  |
| T2DM | -962 (-1,586 to -419) | -1,131 (-2,111 to -434) | -201 (-376 to -79) | -5,156 (-8,678, -2,261) |
| CHD | -386 (-593 to -193) | -470 (-745 to -206) | -88 (-155 to -34) | -2,131 (-3,055, -1,241) |
| Stroke | -50 (-143 to 5) | -66 (-181 to 2) | -9 (-44 to 12) | -271 (-637, -50) |
| Other | 67 (31 to 122) | 84 (36 to 153) | 12 (2 to 31) | 364 (197, 625) |
| **Productivity costs** |  |  |  |  |
| T2DM early retirement | -14 (-35 to 3) | -17 (-45 to 5) | -3 (-9 to 2) | -79 (-187, 6) |
| T2DM sick leave | -702 (-1,623 to -226) | -803 (-2,176 to -265) | -150 (-371 to -37) | -3,654 (-8,539, -1,160) |
| Stroke early retirement | -1 (-18 to 4) | -1 (-26 to 4) | 0 (-6 to 2) | -6 (-99, 2) |
| Stroke sick leave | 0 (-1 to 0) | 0 (-1 to 0) | 0 (0 to 0) | 0 (-3, 0) |
| Premature death | -2,137 (-4,500 to -670) | -2,538 (-5,324 to -810) | -524 (-1,461 to -6) | -9,979 (-18,032, -4,274) |
| **Time costs** |  |  |  |  |
| T2DM self-management | -675 (-1,198 to -269) | -797 (-1,530 to -302) | -144 (-283 to -51) | -3,531 (-6,279, -1,494) |
| T2DM time for health service use | -850 (-2,029 to -281) | -1038 (-2,568 to -311) | -180 (-467 to -53) | -4,523 (-10,600, -1,528) |
| Other time for health service use | 219 (90 to 384) | 260 (102 to 509) | 47 (18 to 92) | 1,152 (475, 1,996) |
| ***Cost-effectiveness*** |  |  |  |  |
| Total change in costs from healthcare perspective (€-millions) | -1,329 (-2,056 to -717) | -1,587 (-2,714 to -726) | -290 (-494 to -123) | -7,133 (-11,494, -3,889) |
| Total change in costs from societal perspective (€-millions) | -5,638 (-9,151 to -2,799) | -6,793 (-11,824 to -2,945) | -1,258 (-2,450 to -534) | -28,629 (-45,623, -14,619) |
| ICER^#^ (healthcare perspective) | Dominant | Dominant | Dominant | Dominant |
| ICER^#^ (societal perspective) | Dominant | Dominant | Dominant | Dominant |

*Cases and case-years prevented/postponed are defined as incident and prevalent cases completely prevented or delayed for one or more years, respectively. ^#^Expressed as € per QALY. Only defined for positive incremental costs and else “dominant” because no trade-off exists if health is improved while costs are saved. Abbreviations: CHD, coronary heart disease; CVD, cardiovascular disease; ICER, incremental cost-effectiveness ratio; QALY, quality-adjusted life year; SSB, sugar-sweetened beverages; T2DM, type 2 diabetes mellitus.

Table X: Health and economic impact using different discount rates for QALYs and costs

| ***Health and cost outcomes*** | **Change in outcomes compared to baseline without tax (95%-uncertainty intervals)** | | |
| --- | --- | --- | --- |
|  | *Ad-valorem* tax* | Extended *ad-valorem* tax^#^ | Tiered tax^‡^ |
| ***Discount rate 1%*** | | | |
| QALYs gained | 138,500 (75,700 to 199,400) | 318,600 (222,900 to 414,200) | 250,200 (168,600 to 332,300) |
| **Healthcare costs (€-millions)** |  |  |  |
| T2DM | -2,158 (-3,652 to -915) | -3101 (-4,637 to -1,772) | -3,765 (-6,081 to -1,686) |
| CHD | -869 (-1,314 to -461) | -1,047 (-1,541 to -612) | -1,504 (-2,141 to -834) |
| Stroke | -120 (-301 to -2) | -275 (-564 to -116) | -209 (-491 to -47) |
| Other | 159 (81 to 270) | 197 (106 to 319) | 260 (133 to 437) |
| **Productivity costs (€-millions)** |  |  |  |
| T2DM early retirement | -32 (-78 to 7) | -47 (-97 to -5) | -55 (-134 to 6) |
| T2DM sick leave | -1,566 (-3,694 to -523) | -2,059 (-4,510 to -737) | -2,700 (-6,251 to -825) |
| Stroke early retirement | -2 (-46 to 7) | -6 (-92 to 0) | -4 (-84 to 3) |
| Stroke sick leave | 0 (-1 to 0) | 0 (-4 to 0) | 0 (-2 to 0) |
| Premature death | -4,595 (-8,986 to -1,585) | -4,939 (-9,344 to -2,000) | -7,649 (-14,107 to -3,011) |
| **Time costs (€-millions)** |  |  |  |
| T2DM self-management | -1,524 (-2,647 to -641) | -1958 (-3218 to -979) | -2,606 (-4,459 to -1,173) |
| T2DM time for health service use | -1,920 (-4,616 to -672) | -2,524 (-5,832 to -957) | -3,317 (-7,523 to -1,156) |
| Other time for health service use | 501 (206 to 866) | 647 (322 to 1,040) | 851 (363 to 1,425) |
| ***Cost-effectiveness*** |  |  |  |
| Total change in costs from healthcare perspective (€-millions) | -3,004 (-4,744 to -1,584) | -4,186 (-6,047 to -2,604) | -5,161 (-8,078 to -2,772) |
| Total change in costs from societal perspective (€-millions) | -12,648 (-20,258 to -6,218) | -15,590 (-23,314 to -8,880) | -21,188 (-33,268 to -10,729) |
| ICER^†^ (healthcare perspective) | Dominant | Dominant | Dominant |
| ICER^†^ (societal perspective) | Dominant | Dominant | Dominant |
| ***Discount rate 5%*** | | | |
| QALYs gained | 82,000 (43,800 to 119,900) | 202,200 (142,200 to 262,100) | 149,500 (102,100 to 197,500) |
| **Healthcare costs (€-millions)** |  |  |  |
| T2DM | -1,230 (-2,118 to -516) | -1,739 (-2,644 to -972) | -2,081 (-3,542 to -936) |
| CHD | -507 (-780 to -261) | -607 (-898 to -352) | -864 (-1,255 to -465) |
| Stroke | -66 (-170 to 1) | -154 (-325 to -62) | -114 (-274 to -25) |
| Other | 88 (43 to 153) | 108 (57 to 177) | 140 (71 to 246) |
| **Productivity costs (€-millions)** |  |  |  |
| T2DM early retirement | -18 (-46 to 4) | -26 (-56 to -2) | -31 (-77 to 4) |
| T2DM sick leave | -885 (-2,162 to -286) | -1,160 (-2,636 to -406) | -1,513 (-3,555 to -456) |
| Stroke early retirement | -1 (-25 to 4) | -4 (-53 to 0) | -2 (-45 to 3) |
| Stroke sick leave | 0 (-1 to 0) | 0 (-2 to 0) | 0 (-1 to 0) |
| Premature death | -2,829 (-5,747 to -1,022) | -3089 (-5,938 to -1,179) | -4,646 (-8,743 to -1,723) |
| **Time costs (€-millions)** |  |  |  |
| T2DM self-management | -867 (-1,563 to -355) | -1,110 (-1,840 to -554) | -1,460 (-2,583 to -640) |
| T2DM time for health service use | -1,103 (-2,717 to -375) | -1,431 (-3,358 to -545) | -1,858 (-4,361 to -627) |
| Other time for health service use | 284 (115 to 500) | 368 (180 to 600) | 473 (201 to 818) |
| ***Cost-effectiveness*** |  |  |  |
| Total change in costs from healthcare perspective (€-millions) | -1,717 (-2,743 to -896) | -2,386 (-3,491 to -1,468) | -2,894 (-4,633 to -1,561) |
| Total change in costs from societal perspective (€-millions) | -7,343 (-11,702 to -3,613) | -9,067 (-13,822 to -5,105) | -12,195 (-19,698 to -6,172) |
| ICER^†^ (healthcare perspective) | Dominant | Dominant | Dominant |
| ICER^†^ (societal perspective) | Dominant | Dominant | Dominant |
| ***Discount rate 10%*** | | | |
| QALYs gained | 46,200 (23,500 to 68,900) | 123,500 (85,200 to 164,100) | 84,100 (58,100 to 110,700) |
| **Healthcare costs (€-millions)** |  |  |  |
| T2DM | -638 (-1,159 to -261) | -897 (-1,431 to -496) | -1,051 (-1,877 to -466) |
| CHD | -277 (-435 to -136) | -328 (-491 to -186) | -459 (-692 to -233) |
| Stroke | -33 (-87 to 0) | -78 (-173 to -30) | -57 (-140 to -11) |
| Other | 44 (21 to 80) | 54 (28 to 91) | 70 (34 to 127) |
| **Productivity costs (€-millions)** |  |  |  |
| T2DM early retirement | -10 (-25 to 3) | -14 (-30 to -1) | -16 (-41 to 2) |
| T2DM sick leave | -461 (-1,183 to -144) | -599 (-1,427 to -205) | -769 (-1,880 to -229) |
| Stroke early retirement | 0 (-13 to 3) | -2 (-28 to 0) | -1 (-22 to 2) |
| Stroke sick leave | 0 (0 to 0) | 0 (-1 to 0) | 0 (-1 to 0) |
| Premature death | -1,640 (-3,478 to -597) | -1,768 (-3,648 to -651) | -2,618 (-5,177 to -1,011) |
| **Time costs (€-millions)** |  |  |  |
| T2DM self-management | -457 (-859 to -184) | -578 (-980 to -284) | -747 (-1,381 to -315) |
| T2DM time for health service use | -581 (-1,444 to -188) | -738 (-1,776 to -277) | -957 (-2,330 to -307) |
| Other time for health service use | 149 (59 to 267) | 192 (91 to 325) | 243 (99 to 431) |
| ***Cost-effectiveness*** |  |  |  |
| Total change in costs from healthcare perspective (€-millions) | -897 (-1,483 to -476) | -1,253 (-1,874 to -754) | -1,504 (-2,456 to -799) |
| Total change in costs from societal perspective (€-millions) | -4,056 (-6,741 to -1,925) | -4,937 (-7,813 to -2,651) | -6,550 (-10,757 to -3,224) |
| ICER^†^ (healthcare perspective) | Dominant | Dominant | Dominant |
| ICER^†^ (societal perspective) | Dominant | Dominant | Dominant |

*“Ad-valorem tax” refers to a 20% ad-valorem tax on SSBs with a pass-through to consumers of 82% (for details see section “Sugar-sweetened beverage taxation scenarios” in the main text). ^#^“Extended ad-valorem tax” refers to a 20% ad-valorem tax on SSBs and fruit juice with a pass-through to consumers of 82% (for details see section “Sugar-sweetened beverage taxation scenarios” in the main text). ^‡^“Tiered tax” refers to a tiered tax on SSBs similar to the United Kingdom Soft Drinks Industry Levy that leads to a reduction in SSB sugar content by 30% through reformulation (for details see section “Sugar-sweetened beverage taxation scenarios” in the main text). ^†^Expressed as € per QALY. Only defined for positive incremental costs and else “dominant” because no trade-off exists if health is improved while costs are saved. Abbreviations: CHD, coronary heart disease; CVD, cardiovascular disease; ICER, incremental cost-effectiveness ratio; QALY, quality-adjusted life year; SSB, sugar-sweetened beverages; T2DM, type 2 diabetes mellitus.

Table Y: Health and economic impact of different SSB taxation scenarios in Germany 2023-2043 by age group

| ***Outcome category*** | ***Age group (in years)*** | **Change in outcomes compared to baseline without tax (95%-uncertainty intervals)** | | |
| --- | --- | --- | --- | --- |
|  |  | *Ad-valorem* tax* | Extended *ad-valorem* tax^#^ | Tiered tax^‡^ |
| ***Health outcomes*** | | | | |
| **Cases prevented/postponed**^†^ |  |  |  |  |
| T2DM | 30-49 | 69,100 (30,800, 113,600) | 85,200 (44,000, 132,500) | 122,000 (54,200, 199,000) |
|  | 50-69 | 50,800 (24,100, 76,900) | 81,600 (51,600, 110,500) | 96,900 (49,400, 142,500) |
|  | 70-90 | 11,600 (4,200, 19,800) | 24,000 (14,200, 34,500) | 23,900 (11,800, 36,600) |
| CHD | 30-49 | 13,000 (6,300, 22,200) | 14,000 (6,900, 23,600) | 22,700 (11,100, 35,500) |
|  | 50-69 | 16,500 (8,800, 25,300) | 19,400 (11,300, 28,600) | 29,800 (16,300, 43,600) |
|  | 70-90 | 9,300 (4,600, 15,000) | 12,300 (7,100, 18,900) | 17,100 (9,900, 26,200) |
| Stroke | 30-49 | 300 (0, 1,600) | 700 (0, 2,200) | 700 (0, 2,300) |
|  | 50-69 | 900 (-200, 2,800) | 2,200 (300, 4,700) | 1,800 (0, 4,200) |
|  | 70-90 | 400 (-500, 1,500) | 1,600 (300, 3,500) | 800 (-400, 2,400) |
| Obesity | 30-49 | 29,300 (5,400, 57,400) | 122,300 (75,600, 169,500) | 62,700 (35,200, 92,200) |
|  | 50-69 | 3,800 (-13,300, 21,200) | 46,500 (15,200, 81,900) | 12,100 (-6,900, 29,800) |
|  | 70-90 | -1,600 (-11,100, 8,300) | -4,900 (-28,300, 16,300) | -3,100 (-15,600, 9,000) |
| **Case-years prevented/postponed**^†^ |  |  |  |  |
| T2DM | 30-49 | 446,900 (190,200, 762,800) | 531,900 (247,400, 893,000) | 761,000 (329,500, 1,278,900) |
|  | 50-69 | 477,200 (207,800, 795,400) | 702,100 (425,100, 1,031,300) | 847,400 (400,700, 1,353,700) |
|  | 70-90 | 180,100 (62,000, 313,000) | 326,800 (198,600, 483,000) | 326,500 (142,700, 549,300) |
| CHD | 30-49 | 68,600 (29,300, 121,500) | 72,900 (31,800, 126,300) | 114,200 (54,500, 194,900) |
|  | 50-69 | 100,800 (42,800, 164,400) | 116,100 (59,100, 185,200) | 174,400 (83,700, 273,000) |
|  | 70-90 | 65,600 (27,800, 109,000) | 82,600 (41,900, 126,700) | 115,700 (51,300, 181,800) |
| Stroke | 30-49 | 1,400 (-1,500, 7,300) | 3,000 (-700, 11,700) | 2,700 (-1,100, 10,700) |
|  | 50-69 | 3,100 (-5,400, 15,200) | 9,500 (-2,000, 24,400) | 5,700 (-5,600, 21,400) |
|  | 70-90 | -400 (-6,900, 7,800) | 6,700 (-3,700, 21,500) | -200 (-8,100, 10,100) |
| Obesity | 30-49 | 376,200 (147,700, 634,400) | 1,356,600 (825,100, 1,869,100) | 790,400 (497,200, 1,070,200) |
|  | 50-69 | 261,300 (-9,000, 555,200) | 1,672,900 (987,800, 2,357,800) | 638,500 (375,800, 898,100) |
|  | 70-90 | 91,800 (-64,500, 246,200) | 888,800 (529,800, 1,268,100) | 254,600 (147,800, 369,700) |
| All-cause deaths prevented/postponed | 30-49 | 2,400 (600, 5,600) | 2,600 (600, 5,700) | 3,900 (1,200, 8,200) |
|  | 50-69 | 7,600 (3,300, 12,700) | 8,900 (4,300, 14,300) | 12,900 (6,000, 20,800) |
|  | 70-90 | 6,800 (3,300, 11,100) | 9,800 (6,100, 14,900) | 11,900 (6,700, 18,500) |
| QALYs gained | 30-49 | 36,000 (19,500, 53,000) | 91,700 (65,200, 120,300) | 68,000 (47,300, 91,000) |
|  | 50-69 | 45,000 (23,500, 66,000) | 100,900 (69,200, 132,400) | 80,100 (51,500, 108,900) |
|  | 70-90 | 24,300 (11,400, 36,300) | 58,900 (40,700, 76,200) | 43,600 (28,600, 61,500) |
| Life-years gained | 30-49 | 10,400 (1,700, 25,500) | 11,100 (1,900, 26,800) | 16,800 (4,000, 37,000) |
|  | 50-69 | 48,100 (20,200, 84,100) | 53,800 (23,200, 91,600) | 76,800 (35,500, 135,400) |
|  | 70-90 | 36,500 (17,700, 61,300) | 49,400 (29,200, 78,800) | 62,000 (32,600, 101,200) |
| ***Health-related cost outcomes (€-millions)*** | | | | |
| **Healthcare costs** |  |  |  |  |
| T2DM | 30-49 | -565 (-996 to -239) | -678 (-1,144 to -307) | -944 (-1,661 to -406) |
|  | 50-69 | -719 (-1233 to -303) | -1,049 (-1,610 to -618) | -1,247 (-2,076 to -574) |
|  | 70-90 | -318 (-568 to -103) | -564 (-842 to -333) | -563 (-966 to -229) |
| CHD | 30-49 | -107 (-188 to -48) | -116 (-200 to -53) | -181 (-308 to -85) |
|  | 50-69 | -328 (-518 to -158) | -384 (-590 to -212) | -564 (-841 to -296) |
|  | 70-90 | -216 (-335 to -104) | -293 (-415 to -164) | -378 (-562 to -202) |
| Stroke | 30-49 | -13 (-63 to 4) | -23 (-95 to 4) | -25 (-95 to 0) |
|  | 50-69 | -41 (-118 to 10) | -92 (-190 to -18) | -73 (-181 to -4) |
|  | 70-90 | -28 (-89 to 20) | -86 (-186 to -25) | -52 (-130 to 1) |
| Other | 30-49 | 5 (1 to 14) | 6 (1 to 15) | 9 (2 to 21) |
|  | 50-69 | 47 (20 to 85) | 53 (24 to 93) | 74 (33 to 134) |
|  | 70-90 | 65 (30 to 116) | 86 (47 to 140) | 108 (57 to 188) |
| **Productivity costs** |  |  |  |  |
| T2DM early retirement | 30-49 | -19 (-40 to -4) | -23 (-46 to -8) | -31 (-66 to -10) |
|  | 50-69 | -5 (-23 to 14) | -12 (-31 to 6) | -11 (-39 to 17) |
|  | 70-90* | 0 (0 to 0) | 0 (0 to 0) | 0 (0 to 0) |
| T2DM sick leave | 30-49 | -628 (-1,479 to -210) | -762 (-1,700 to -249) | -1,069 (-2,433 to -339) |
|  | 50-69 | -527 (-1,337 to -178) | -764 (-1,716 to -277) | -924 (-2,289 to -308) |
|  | 70-90^§^ | 0 (0 to 0) | 0 (0 to 0) | 0 (0 to 0) |
| Stroke early retirement | 30-49 | 0 (-19 to 2) | -1 (-29 to 1) | -1 (-30 to 1) |
|  | 50-69 | -1 (-20 to 7) | -3 (-50 to 1) | -2 (-36 to 6) |
|  | 70-90^§^ | 0 (0 to 0) | 0 (0 to 0) | 0 (0 to 0) |
| Stroke sick leave | 30-49 | 0 (0 to 0) | 0 (-1 to 0) | 0 (0 to 0) |
|  | 50-69 | 0 (-1 to 0) | 0 (-2 to 0) | 0 (-1 to 0) |
|  | 70-90^§^ | 0 (0 to 0) | 0 (0 to 0) | 0 (0 to 0) |
| Premature death | 30-49 | -2,002 (-4,809 to -477) | -2,128 (-4,964 to -601) | -3,273 (-6,986 to -945) |
|  | 50-69 | -1,535 (-2,933 to -432) | -1,680 (-3,146 to -526) | -2,529 (-4,515 to -1,031) |
|  | 70-90^§^ | 0 (0 to 0) | 0 (0 to 0) | 0 (0 to 0) |
| **Time costs** |  |  |  |  |
| T2DM self-management | 30-49 | -604 (-1,116 to -244) | -725 (-1,268 to -306) | -1,026 (-1,808 to -413) |
|  | 50-69 | -539 (-957 to -219) | -746 (-1,186 to -415) | -924 (-1,601 to -401) |
|  | 70-90^§^ | 0 (0 to 0) | 0 (0 to 0) | 0 (0 to 0) |
| T2DM time for health service use | 30-49 | -752 (-1,844 to -252) | -918 (-2,236 to -331) | -1,269 (-3,024 to -431) |
|  | 50-69 | -681 (-1,574 to -231) | -960 (-2,153 to -380) | -1,178 (-2,695 to -416) |
|  | 70-90^§^ | 0 (0 to 0) | 0 (0 to 0) | 0 (0 to 0) |
| Other time for health service use | 30-49 | 193 (77 to 356) | 227 (102 to 406) | 322 (127 to 578) |
|  | 50-69 | 178 (76 to 317) | 249 (137 to 400) | 305 (140 to 529) |
|  | 70-90^§^ | 0 (0 to 0) | 0 (0 to 0) | 0 (0 to 0) |
| ***Cost-effectiveness*** | | | | |
| Total change in costs from healthcare perspective (€-millions) | 30-49 | -691 (-1,166 to -317) | -809 (-1,336 to -405) | -1,147 (-1,913 to -550) |
|  | 50-69 | -1,054 (-1,664 to -572) | -1,485 (-2,134 to -920) | -1,812 (-2,796 to -1,015) |
|  | 70-90 | -504 (-811 to -211) | -863 (-1,255 to -517) | -889 (-1,437 to -467) |
| Total change in costs from societal perspective (€-millions) | 30-49 | -4,769 (-8,587 to -2,067) | -5,396 (-9,275 to -2,418) | -7,846 (-13,077 to -3,477) |
|  | 50-69 | -4,224 (-6,738 to -2,130) | -5,470 (-8,219 to -3,170) | -7,211 (-11,493 to -3,957) |
|  | 70-90 | -504 (-811 to -211) | -863 (-1,255 to -517) | -889 (-1,437 to -467) |

*“Ad-valorem tax” refers to a 20% ad-valorem tax on SSBs with a pass-through to consumers of 82% (for details see section “Sugar-sweetened beverage taxation scenarios” in the main text). ^#^“Extended ad-valorem tax” refers to a 20% ad-valorem tax on SSBs and fruit juice with a pass-through to consumers of 82% (for details see section “Sugar-sweetened beverage taxation scenarios” in the main text). ^‡^“Tiered tax” refers to a tiered tax on SSBs similar to the United Kingdom Soft Drinks Industry Levy that leads to a reduction in SSB sugar content by 30% through reformulation (for details see section “Sugar-sweetened beverage taxation scenarios” in the main text). ^†^Cases and case-years prevented/postponed are defined as incident and prevalent cases completely prevented or delayed for one or more years, respectively. ^§^Productivity costs are irrelevant for individuals above retirement age (65+ years) and therefore zero. Abbreviations: CHD, coronary heart disease; QALY, quality-adjusted life year; SSB, sugar-sweetened beverages; T2DM, type 2 diabetes mellitus.

Table Z: Health and economic impact of SSB taxation scenarios in Germany 2023-2043 by sex

| ***Outcome category*** | ***Sex*** | **Change in outcomes compared to baseline without tax (95%-uncertainty intervals)** | | |
| --- | --- | --- | --- | --- |
|  |  | *Ad-valorem* tax* | Extended *ad-valorem* tax^#^ | Tiered tax^‡^ |
| ***Health outcomes*** | | | | |
| **Cases prevented/postponed**^†^ |  |  |  |  |
| T2DM | Male | 104,700 (50,000, 158,900) | 144,700 (84,500, 203,800) | 193,800 (94,600, 287,000) |
|  | Female | 26,600 (10,800, 44,400) | 46,100 (27,900, 66,400) | 50,600 (22,900, 80,000) |
| CHD | Male | 34,300 (18,600, 51,000) | 39,700 (23,400, 57,600) | 61,100 (33,700, 89,600) |
|  | Female | 4,700 (2,000, 8,300) | 6,300 (3,000, 10,900) | 8,600 (4,200, 14,400) |
| Stroke | Male | 1,500 (-100, 3,800) | 3,200 (1,200, 6,600) | 2,700 (500, 5,900) |
|  | Female | 300 (-300, 1300) | 1,200 (300, 3,100) | 600 (-100, 2,100) |
| Obesity | Male | 27,200 (-100, 56,500) | 109,700 (70,400, 162,200) | 57,300 (28,700, 87,700) |
|  | Female | 4,400 (-9,300, 18,600) | 50,500 (22,100, 80,900) | 14,600 (2,700, 26,700) |
| **Case-years prevented/postponed**^†^ |  |  |  |  |
| T2DM | Male | 885,400 (379,400, 1,434,100) | 1,188,900 (646,700, 1,742,600) | 1,542,400 (702,700, 2,463,400) |
|  | Female | 228,500 (91,500, 415,400) | 382,100 (229,000, 586,600) | 411,900 (178,700, 702,600) |
| CHD | Male | 207,200 (96,800, 326,700) | 233,300 (122,200, 361,500) | 353,600 (179,100, 545,900) |
|  | Female | 30,500 (9,400, 60,400) | 38,700 (13,800, 72,100) | 53,200 (20,900, 95,700) |
| Stroke | Male | 3,500 (-7,600, 18,300) | 13,000 (-1,000, 36,200) | 6,400 (-9,400, 27,900) |
|  | Female | 700 (-3,700, 8,000) | 5,900 (-1,600, 18,400) | 2,000 (-3,200, 9,800) |
| Obesity | Male | 625,600 (179,800, 1,147,400) | 2,727,100 (1,622,700, 3,853,300) | 1,381,500 (848,600, 1,912,800) |
|  | Female | 108,500 (-102,100, 290,400) | 1,180,300 (714,200, 1,664,900) | 308,600 (188,200, 430,000) |
| All-cause deaths prevented/postponed | Male | 14,900 (7,300, 23,200) | 18,100 (10,300, 26,800) | 25,500 (13,800, 39,100) |
|  | Female | 2,100 (700, 4,000) | 3,500 (1,600, 5,900) | 3,700 (1,600, 6,600) |
| QALYs gained | Male | 89,100 (51,500, 127,900) | 178,100 (122,000, 232,000) | 158,100 (106,600, 210,300) |
|  | Female | 16,700 (4,000, 28,600) | 74,200 (53,900, 97,500) | 34,300 (24,000, 45,800) |
| Life-years gained | Male | 85,500 (40,400, 144,200) | 99,800 (52,500, 162,200) | 139,800 (70,800, 230,000) |
|  | Female | 9,700 (2,500, 21,500) | 15,500 (5,700, 28,600) | 16,300 (6,300, 32,200) |
| Life expectancy | Male | 0.03 (-0.02, 0.1) | 0.03 (-0.02, 0.11) | 0.05 (-0.01, 0.15) |
|  | Female | 0 (-0.01, 0.03) | 0 (-0.01, 0.03) | 0 (-0.01, 0.04) |
| Life expectancy at age 60 years | Male | 0.01 (-0.01, 0.04) | 0.01 (-0.02, 0.05) | 0.02 (-0.01, 0.07) |
|  | Female | 0 (-0.01, 0.02) | 0 (-0.01, 0.02) | 0 (-0.01, 0.02) |
| ***Health-related cost outcomes (€-millions)*** | | | | |
| **Healthcare costs** |  |  |  |  |
| T2DM | Male | -1,224 (-2,041 to -524) | -1,654 (-2,479 to -903) | -2,084 (-3,420 to -959) |
|  | Female | -390 (-746 to -158) | -648 (-1,025 to -375) | -688 (-1,222 to -292) |
| CHD | Male | -567 (-829 to -282) | -663 (-961 to -373) | -966 (-1,389 to -509) |
|  | Female | -92 (-176 to -34) | -130 (-230 to -59) | -166 (-293 to -76) |
| Stroke | Male | -67 (-176 to 7) | -138 (-304 to -47) | -117 (-280 to -5) |
|  | Female | -19 (-78 to 14) | -62 (-166 to -10) | -36 (-117 to 2) |
| Other | Male | 104 (48 to 180) | 122 (65 to 206) | 167 (84 to 288) |
|  | Female | 14 (3 to 33) | 22 (7 to 45) | 23 (8 to 49) |
| **Productivity costs** |  |  |  |  |
| T2DM early retirement | Male | -17 (-47 to 8) | -25 (-57 to 1) | -30 (-79 to 9) |
|  | Female | -6 (-15 to -1) | -10 (-19 to -4) | -11 (-24 to -3) |
| T2DM sick leave | Male | -965 (-2,291 to -306) | -1,235 (-2,801 to -417) | -1,636 (-3,853 to -505) |
|  | Female | -204 (-527 to -66) | -297 (-679 to -118) | -365 (-874 to -126) |
| Stroke early retirement | Male | -1 (-30 to 6) | -4 (-60 to 1) | -2 (-53 to 4) |
|  | Female | 0 (-7 to 1) | 0 (-16 to 1) | 0 (-10 to 1) |
| Stroke sick leave | Male | 0 (-1 to 0) | 0 (-2 to 0) | 0 (-2 to 0) |
|  | Female | 0 (0 to 0) | 0 (0 to 0) | 0 (0 to 0) |
| Premature death | Male | -3,469 (-6,842 to -1,188) | -3,760 (-7,191 to -1,406) | -5,777 (-10,878 to -2,232) |
|  | Female | -83 (-412 to 0) | -107 (-452 to 0) | -161 (-558 to 0) |
| **Time costs** |  |  |  |  |
| T2DM self-management | Male | -953 (-1,660 to -390) | -1,182 (-1,938 to -595) | -1,612 (-2,753 to -713) |
|  | Female | -196 (-395 to -77) | -282 (-488 to -143) | -339 (-636 to -134) |
| T2DM time for health service use | Male | -1,203 (-2,857 to -415) | -1,521 (-3,576 to -574) | -2,038 (-4,734 to -703) |
|  | Female | -247 (-626 to -89) | -367 (-853 to -143) | -427 (-1,093 to -147) |
| Other time for health service use | Male | 311 (130 to 538) | 387 (188 to 629) | 527 (223 to 885) |
|  | Female | 62 (23 to 121) | 90 (43 to 158) | 107 (42 to 201) |
| ***Cost-effectiveness*** | | | | |
| Total change in costs from healthcare perspective (€-millions) | Male | -1,762 (-2,724 to -927) | -2,348 (-3,320 to -1,436) | -2,980 (-4,574 to -1,639) |
|  | Female | -492 (-877 to -223) | -824 (-1,295 to -503) | -870 (-1,441 to -435) |
| Total change in costs from societal perspective (€-millions) | Male | -8,358 (-12,952 to -4,122) | -9,963 (-15,008 to -5,569) | -13,841 (-21,803 to -7,004) |
|  | Female | -1,207 (-2,241 to -553) | -1,867 (-3,044 to -1,065) | -2,138 (-3,725 to -995) |

*“Ad-valorem tax” refers to a 20% ad-valorem tax on SSBs with a pass-through to consumers of 82% (for details see section “Sugar-sweetened beverage taxation scenarios” in the main text). ^#^“Extended ad-valorem tax” refers to a 20% ad-valorem tax on SSBs and fruit juice with a pass-through to consumers of 82% (for details see section “Sugar-sweetened beverage taxation scenarios” in the main text). ^‡^“Tiered tax” refers to a tiered tax on SSBs similar to the United Kingdom Soft Drinks Industry Levy that leads to a reduction in SSB sugar content by 30% through reformulation (for details see section “Sugar-sweetened beverage taxation scenarios” in the main text). ^†^Cases and case-years prevented/postponed are defined as incident and prevalent cases completely prevented or delayed for one or more years, respectively. Abbreviations: CHD, coronary heart disease; QALY, quality-adjusted life year; SSB, sugar-sweetened beverages; T2DM, type 2 diabetes mellitus.

Table AA: Health and economic impact of SSB taxation scenarios in Germany 2023-2043 by included exposures

| ***Outcome category*** | ***Exposures*** | **Change in outcomes compared to baseline without tax (95%-uncertainty intervals)** | | |
| --- | --- | --- | --- | --- |
|  |  | *Ad-valorem* tax* | Extended *ad-valorem* tax^#^ | Tiered tax^‡^ |
| ***Health outcomes*** | | | | |
| **Cases prevented/postponed**^†^ |  |  |  |  |
| T2DM | SSB & BMI | 132,100 (61,700, 202,900) | 190,800 (112,000, 269,700) | 244,100 (118,200, 365,300) |
|  | BMI | 14,100 (1,800, 27,500) | 72,000 (41,600, 105,300) | 32,400 (18,500, 46,700) |
| CHD | SSB & BMI | 39,200 (21,100, 58,100) | 45,800 (27,500, 66,200) | 69,800 (38,800, 101,900) |
|  | BMI | 1,400 (-200, 3,900) | 7,800 (3,500, 13,900) | 3,300 (1,200, 6,400) |
| Stroke | SSB & BMI | 1,900 (0, 4,500) | 4,500 (1,900, 8,500) | 3,400 (800, 7,100) |
|  | BMI | 600 (-300, 1,700) | 3,400 (1,100, 6,600) | 1,300 (100, 2,900) |
| Obesity | SSB & BMI | 31,600 (-5,400, 72,600) | 159,400 (97,100, 232,400) | 72,300 (36,400, 105,500) |
|  | BMI | 31,900 (-5,300, 70,300) | 158,700 (99,100, 232,200) | 72,100 (38,100, 106,600) |
| **Case-years prevented/postponed**^†^ |  |  |  |  |
| T2DM | SSB & BMI | 1,109,300 (481,700, 1,838,200) | 1,569,600 (876,500, 2,313,800) | 1,940,900 (879,200, 3,106,500) |
|  | BMI | 107,900 (16,900, 227,100) | 552,200 (298,100, 892,500) | 240,900 (131,400, 394,700) |
| CHD | SSB & BMI | 239,700 (112,300, 375,600) | 274,700 (146,600, 415,100) | 408,200 (206,600, 620,900) |
|  | BMI | 8,000 (-3,400, 25,700) | 42,500 (17,600, 83,200) | 18,100 (3,400, 41,200) |
| Stroke | SSB & BMI | 4,300 (-6,600, 22,200) | 18,600 (2,300, 50,200) | 8,900 (-6,600, 32,300) |
|  | BMI | 3,000 (-2,600, 12,600) | 18,700 (3,800, 42,800) | 7,000 (-400, 19,100) |
| Obesity | SSB & BMI | 733,800 (99,600, 1,431,500) | 3,919,200 (2,340,700, 5,490,500) | 1,683,100 (1,035,800, 2,341,000) |
|  | BMI | 780,300 (131,500, 1,473,000) | 3,969,600 (2,383,600, 5,535,100) | 1,752,000 (1,103,400, 2,404,700) |
| All-cause deaths prevented/postponed | SSB & BMI | 17,000 (8,600, 26,100) | 21,600 (12,600, 31,800) | 29,300 (15,900, 44,900) |
|  | BMI | 900 (-300, 2,600) | 5,300 (2,000, 9,900) | 1,900 (500, 4,300) |
| QALYs gained | SSB & BMI | 106,000 (57,200, 153,200) | 252,400 (176,700, 325,800) | 192,300 (130,100, 254,200) |
|  | BMI | 32,600 (3,700, 60,700) | 177,600 (121,500, 242,300) | 73,500 (51,600, 98,400) |
| Life-years gained | SSB & BMI | 954,00 (47,300, 161,000) | 114,200 (61,300, 187,300) | 156,700 (77,900, 255,400) |
|  | BMI | 3,600 (-2,600, 11,800) | 20,800 (7,000, 45,000) | 7,900 (1,500, 18,800) |
| Life expectancy | SSB & BMI | 0.02 (-0.01, 0.05) | 0.02 (-0.01, 0.06) | 0.03 (0, 0.08) |
|  | BMI | 0 (0, 0.01) | 0 (-0.01, 0.02) | 0 (-0.01, 0.01) |
| Life expectancy at age 60 years | SSB & BMI | 0.01 (-0.01, 0.02) | 0.01 (-0.01, 0.03) | 0.01 (-0.01, 0.04) |
|  | BMI | 0 (0, 0.01) | 0 (-0.01, 0.01) | 0 (-0.01, 0.01) |
| ***Health-related cost outcomes (€-millions)*** | | | | |
| **Healthcare costs** |  |  |  |  |
| T2DM | SSB & BMI | -1,613 (-2,750 to -684) | -2,310 (-3,492 to -1,311) | -2,785 (-4,601 to -1,249) |
|  | BMI | -154 (-333 to -21) | -817 (-1,388 to -421) | -341 (-578 to -176) |
| CHD | SSB & BMI | -660 (-1,003 to -345) | -792 (-1,170 to -461) | -1,136 (-1,631 to -619) |
|  | BMI | -28 (-82 to 7) | -160 (-282 to -79) | -63 (-127 to -20) |
| Stroke | SSB & BMI | -89 (-225 to 0) | -204 (-425 to -84) | -153 (-364 to -35) |
|  | BMI | -24 (-83 to 14) | -147 (-313 to -49) | -52 (-139 to -7) |
| Other | SSB & BMI | 118 (59 to 202) | 144 (77 to 236) | 190 (97 to 326) |
|  | BMI | 5 (-5 to 17) | 28 (9 to 65) | 10 (2 to 27) |
| **Productivity costs** |  |  |  |  |
| T2DM early retirement | SSB & BMI | -24 (-60 to 5) | -35 (-73 to -3) | -41 (-101 to 4) |
|  | BMI | -3 (-9 to 0) | -14 (-29 to -6) | -7 (-15 to -3) |
| T2DM sick leave | SSB & BMI | -1,170 (-2,809 to -384) | -1,536 (-3,427 to -541) | -2,013 (-4,707 to -610) |
|  | BMI | -100 (-292 to -22) | -443 (-1,036 to -149) | -220 (-535 to -79) |
| Stroke early retirement | SSB & BMI | -1 (-33 to 5) | -5 (-70 to 0) | -3 (-62 to 3) |
|  | BMI | 0 (-17 to 2) | -3 (-56 to 0) | -1 (-26 to 0) |
| Stroke sick leave | SSB & BMI | 0 (-1 to 0) | 0 (-3 to 0) | 0 (-2 to 0) |
|  | BMI | 0 (0 to 0) | 0 (-2 to 0) | 0 (-1 to 0) |
| Premature death | SSB & BMI | -3,556 (-7,135 to -1,260) | -3,904 (-7,388 to -1,518) | -5,913 (-10,999 to -2,265) |
|  | BMI | -20 (-415 to 24) | -241 (-980 to 0) | -116 (-650 to 0) |
| **Time costs** |  |  |  |  |
| T2DM self-management | SSB & BMI | -1,146 (-2,020 to -475) | -1,461 (-2,420 to -730) | -1,941 (-3,368 to -863) |
|  | BMI | -97 (-224 to -20) | -417 (-772 to -194) | -213 (-402 to -96) |
| T2DM time for health service use | SSB & BMI | -1,446 (-3,508 to -502) | -1,892 (-4,424 to -718) | -2,470 (-5,667 to -847) |
|  | BMI | -124 (-357 to -27) | -537 (-1,344 to -202) | -269 (-651 to -103) |
| Other time for health service use | SSB & BMI | 374 (153 to 651) | 485 (239 to 781) | 633 (269 to 1,068) |
|  | BMI | 32 (6 to 71) | 132 (57 to 239) | 67 (30 to 125) |
| ***Cost-effectiveness*** | | | | |
| Total change in costs from healthcare perspective (€-millions) | SSB & BMI | -2,262 (-3,596 to -1,189) | -3,141 (-4,568 to -1,942) | -3,850 (-6,075 to -2,070) |
|  | BMI | -204 (-448 to -29) | -1,095 (-1,828 to -590) | -455 (-759 to -233) |
| Total change in costs from societal perspective (€-millions) | SSB & BMI | -9,584 (-15,304 to -4,714) | -11,827 (-17,887 to -6,702) | -16,013 (-25,500 to -8,090) |
|  | BMI | -589 (-1,352 to -128) | -2,704 (-5,002 to -1,345) | -1,292 (-2,334 to -620) |

*“Ad-valorem tax” refers to a 20% ad-valorem tax on SSBs with a pass-through to consumers of 82% (for details see section “Sugar-sweetened beverage taxation scenarios” in the main text). ^#^“Extended ad-valorem tax” refers to a 20% ad-valorem tax on SSBs and fruit juice with a pass-through to consumers of 82% (for details see section “Sugar-sweetened beverage taxation scenarios” in the main text). ^‡^“Tiered tax” refers to a tiered tax on SSBs similar to the United Kingdom Soft Drinks Industry Levy that leads to a reduction in SSB sugar content by 30% through reformulation (for details see section “Sugar-sweetened beverage taxation scenarios” in the main text). ^†^Cases and case-years prevented/postponed are defined as incident and prevalent cases completely prevented or delayed for one or more years, respectively. Abbreviations: BMI, body mass index; CHD, coronary heart disease; QALY, quality-adjusted life year; SSB, sugar-sweetened beverages; T2DM, type 2 diabetes mellitus.

Table AB: Health impact of different SSB taxation scenarios in Germany 2023-2043 by simulation model

| ***Health outcomes*** | ***Simulation model*** | **Change in outcomes compared to baseline without tax (95%-uncertainty intervals)** | | |
| --- | --- | --- | --- | --- |
|  |  | *Ad-valorem* tax* | Extended *ad-valorem* tax^#^ | Tiered tax^‡^ |
| **Cases prevented/postponed**^†^ |  |  |  |  |
| CHD | IMPACT_NCD_ | -1,400 (-3,900, 200) | -7,800 (-13,900, -3,500) | -3,300 (-6,400, -1,200) |
|  | PRIMEtime | -1,100 (-1,600, -700) | -6,400 (-7,700, -5,300) | -2,500 (-2,900, -2,100) |
|  | ***Ratio*** | **1.27** | **1.21** | **1.33** |
|  |  |  |  |  |
| Stroke | IMPACT_NCD_ | -600 (-1,700, 300) | -3,400 (-6,600, -1,100) | -1,300 (-2,900, -100) |
|  | PRIMEtime | -500 (-700, -300) | -3,100 (-3,900, -2,400) | -1,100 (-1,400, -900) |
|  | ***Ratio*** | **1.18** | **1.1** | **1.16** |
|  |  |  |  |  |
| T2DM | IMPACT_NCD_ | -14,100 (-27,500, -1,800) | -72,000 (-105,300, -41,600) | -32,400 (-46,700, -18,500) |
|  | PRIMEtime | -19,300 (-27,100, -12,500) | -117,800 (-145,600, -96,000) | -43,900 (-53,300, -35,200) |
|  | ***Ratio*** | **0.73** | **0.61** | **0.74** |
|  |  |  |  |  |
| **QALYs gained** | IMPACT_NCD_ | -32,600 (-60,700, -3700) | -177,600 (-242,300, -121,500) | -73,500 (-98,400, -51,600) |
|  | PRIMEtime | -25,200 (-34,700, -16,900) | -144,100 (-183,500, -110,100) | -55,900 (-69,800, -43,000) |
|  | ***Ratio*** | **1.29** | **1.23** | **1.32** |

*“Ad-valorem tax” refers to a 20% ad-valorem tax on SSBs with a pass-through to consumers of 82% (for details see section “Sugar-sweetened beverage taxation scenarios” in the main text). ^#^“Extended ad-valorem tax” refers to a 20% ad-valorem tax on SSBs and fruit juice with a pass-through to consumers of 82% (for details see section “Sugar-sweetened beverage taxation scenarios” in the main text). ^‡^“Tiered tax” refers to a tiered tax on SSBs similar to the United Kingdom Soft Drinks Industry Levy that leads to a reduction in SSB sugar content by 30% through reformulation (for details see section “Sugar-sweetened beverage taxation scenarios” in the main text). ^†^Cases prevented/postponed are defined as incident cases completely prevented or delayed for one or more years. Abbreviations: CHD, coronary heart disease; NCD, non-communicable disease; QALY, quality-adjusted life year; SSB, sugar-sweetened beverages; T2DM, type 2 diabetes mellitus.

# Supplemental References

1. Collins B, Kypridemos C, PEARSON‐STUTTARD J, Huang Y, Bandosz P, Wilde P, et al. FDA Sodium Reduction Targets and the Food Industry: Are There Incentives to Reformulate? Microsimulation Cost‐Effectiveness Analysis. Milbank Q. 2019;97(3):858-80.

2. Kypridemos C, Guzman-Castillo M, Hyseni L, Hickey GL, Bandosz P, Buchan I, et al. Estimated reductions in cardiovascular and gastric cancer disease burden through salt policies in England: an IMPACTNCD microsimulation study. BMJ Open. 2017;7(1):e013791.

3. Kypridemos C, Collins B, McHale P, Bromley H, Parvulescu P, Capewell S, O'Flaherty M. Future cost-effectiveness and equity of the NHS Health Check cardiovascular disease prevention programme: Microsimulation modelling using data from Liverpool, UK. PLoS Med. 2018;15(5):e1002573.

4. Laverty AA, Kypridemos C, Seferidi P, Vamos EP, Pearson-Stuttard J, Collins B, et al. Quantifying the impact of the Public Health Responsibility Deal on salt intake, cardiovascular disease and gastric cancer burdens: interrupted time series and microsimulation study. J Epidemiol Community Health. 2019;73(9):881-7.

5. Nilson EAF, Pearson-Stuttard J, Collins B, Guzman-Castillo M, Capewell S, O'Flaherty M, et al. Estimating the health and economic effects of the voluntary sodium reduction targets in Brazil: microsimulation analysis. BMC Med. 2021;19(1):225.

6. Pearson-Stuttard J, Kypridemos C, Collins B, Mozaffarian D, Huang Y, Bandosz P, et al. Estimating the health and economic effects of the proposed US Food and Drug Administration voluntary sodium reformulation: Microsimulation cost-effectiveness analysis. PLoS Med. 2018;15(4):e1002551.

7. Collins B, Kypridemos C, Cookson R, Parvulescu P, McHale P, Guzman-Castillo M, et al. Universal or targeted cardiovascular screening? Modelling study using a sector-specific distributional cost effectiveness analysis. Prev Med. 2020;130:105879.

8. Huang Y, Kypridemos C, Liu J, Lee Y, Pearson-Stuttard J, Collins B, et al. Cost-Effectiveness of the US Food and Drug Administration Added Sugar Labeling Policy for Improving Diet and Health. Circulation. 2019;139(23):2613-24.

9. Holle R, Happich M, Löwel H, Wichmann HE, null. KORA - A Research Platform for Population Based Health Research. Gesundheitswesen. 2005;67(S 01):19-25.

10. Heuer T, Krems C, Moon K, Brombach C, Hoffmann I. Food consumption of adults in Germany: results of the German National Nutrition Survey II based on diet history interviews. Br J Nutr. 2015;113(10):1603-14.

11. Schmidt C, Reitzle L, Dress J, Rommel A, Ziese T, Heidemann C. Prevalence and incidence of documented diabetes based on health claims data-reference analysis for diabetes surveillance in Germany. Bundesgesundheitsblatt Gesundheitsforschung Gesundheitsschutz. 2020;63(1):93-102.

12. DeStatis, Robert Koch-Institut. Statistisches Bundesamt (DeStatis). Informationssystem der Gesundheitsberichterstattung des Bundes. 2023. [accessed 19 May 2022]. Available from: <https://www.gbe-bund.de/gbe/>.

13. DeStatis. Statistisches Bundesamt (DeStatis). GENESIS-Online. 2023. [accessed 19 May 2022]. Available from: <https://www-genesis.destatis.de/genesis/online>.

14. DeStatis. Statistisches Bundesamt (DeStatis). 14. koordinierte Bevölkerungsvorausberechnung für Deutschland. 2022. [accessed 19 May 2022]. Available from: <https://www.destatis.de/DE/Themen/Gesellschaft-Umwelt/Bevoelkerung/Bevoelkerungsvorausberechnung/_inhalt.html#_oz53odqfm>.

15. group SOw, collaboration ESCCr. SCORE2-OP risk prediction algorithms: estimating incident cardiovascular event risk in older persons in four geographical risk regions. Eur Heart J. 2021;42(25):2455-67.

16. group Sw, collaboration ESCCr. SCORE2 risk prediction algorithms: new models to estimate 10-year risk of cardiovascular disease in Europe. Eur Heart J. 2021;42(25):2439-54.

17. Ricci C, Wood A, Muller D, Gunter MJ, Agudo A, Boeing H, et al. Alcohol intake in relation to non-fatal and fatal coronary heart disease and stroke: EPIC-CVD case-cohort study. BMJ. 2018;361:k934.

18. UK Data Service. Health Survey for England, 2013. 2015. Available from: <http://doi.org/10.5255/UKDA-SN-7649-1>.

19. Barendregt JJ, Van Oortmarssen GJ, Vos T, Murray CJ. A generic model for the assessment of disease epidemiology: the computational basis of DisMod II. Popul Health Metr. 2003;1(1):4.

20. Hyndman RJ, Shahid Ullah M. Robust forecasting of mortality and fertility rates: A functional data approach. Comput Stat Data Anal. 2007;51(10):4942-56.

21. Micha R, Peñalvo JL, Cudhea F, Imamura F, Rehm CD, Mozaffarian D. Association Between Dietary Factors and Mortality From Heart Disease, Stroke, and Type 2 Diabetes in the United States. JAMA. 2017;317(9):912-24.

22. Xi B, Huang Y, Reilly KH, Li S, Zheng R, Barrio-Lopez MT, et al. Sugar-sweetened beverages and risk of hypertension and CVD: a dose–response meta-analysis. Br J Nutr. 2015;113(5):709-17.

23. Ioannidis JPA. The Challenge of Reforming Nutritional Epidemiologic Research. JAMA. 2018;320(10):969-70.

24. Imamura F, O'Connor L, Ye Z, Mursu J, Hayashino Y, Bhupathiraju SN, Forouhi NG. Consumption of sugar sweetened beverages, artificially sweetened beverages, and fruit juice and incidence of type 2 diabetes: systematic review, meta-analysis, and estimation of population attributable fraction. BMJ. 2015;351:h3576.

25. Lu Y, Hajifathalian K, Ezzati M, Woodward M, Rimm EB, Danaei G. Metabolic mediators of the effects of body-mass index, overweight, and obesity on coronary heart disease and stroke: a pooled analysis of 97 prospective cohorts with 1·8 million participants. Lancet. 2014;383(9921):970-83.

26. Singh GM, Danaei G, Farzadfar F, Stevens GA, Woodward M, Wormser D, et al. The age-specific quantitative effects of metabolic risk factors on cardiovascular diseases and diabetes: a pooled analysis. PLoS One. 2013;8(7):e65174.

27. Collaboration TERF. Diabetes mellitus, fasting blood glucose concentration, and risk of vascular disease: a collaborative meta-analysis of 102 prospective studies. Lancet. 2010;375(9733):2215-22.

28. Stringhini S, Carmeli C, Jokela M, Avendaño M, Muennig P, Guida F, et al. Socioeconomic status and the 25 × 25 risk factors as determinants of premature mortality: a multicohort study and meta-analysis of 1·7 million men and women. Lancet. 2017;389(10075):1229-37.

29. Andreyeva T, Marple K, Marinello S, Moore TE, Powell LM. Outcomes Following Taxation of Sugar-Sweetened Beverages: A Systematic Review and Meta-analysis. JAMA Netw Open. 2022;5(6):e2215276.

30. Organization WH. Taxes on sugary drinks: Why do it? : World Health Organization; 2017. DOI:

31. Emmert-Fees KMF, Karl FM, von Philipsborn P, Rehfuess EA, Laxy M. Simulation Modeling for the Economic Evaluation of Population-Based Dietary Policies: A Systematic Scoping Review. Adv Nutr. 2021;12(5):1957-95.

32. von Philipsborn P, Huizinga O, Leibinger A, Rubin D, Burns J, Emmert-Fees K, et al. Interim Evaluation of Germany's Sugar Reduction Strategy for Soft Drinks: Commitments versus Actual Trends in Sugar Content and Sugar Sales from Soft Drinks. Ann Nutr Metab. 2023.

33. Kahm K, Laxy M, Schneider U, Rogowski WH, Lhachimi SK, Holle R. Health Care Costs Associated With Incident Complications in Patients With Type 2 Diabetes in Germany. Diabetes Care. 2018;41(5):971-8.

34. Kahm K, Stark R, Laxy M, Schneider U, Leidl R. Assessment of excess medical costs for persons with type 2 diabetes according to age groups: an analysis of German health insurance claims data. Diabet Med. 2020;37(10):1752-8.

35. Ulrich S, Holle R, Wacker M, Stark R, Icks A, Thorand B, et al. Cost burden of type 2 diabetes in Germany: results from the population-based KORA studies. BMJ Open. 2016;6(11):e012527.

36. Winter Y, Wolfram C, Schoffski O, Dodel RC, Back T. [Long-term disease-related costs 4 years after stroke or TIA in Germany]. Nervenarzt. 2008;79(8):918-20, 22-4, 26.

37. Icks A, Haastert B, Arend W, Konein J, Thorand B, Holle R, et al. Patient time costs due to self-management in diabetes may be as high as direct medical costs: results from the population-based KORA survey FF4 in Germany. Diabet Med. 2020;37(5):895-7.

38. Icks A, Claessen H, Strassburger K, Waldeyer R, Chernyak N, Julich F, et al. Patient time costs attributable to healthcare use in diabetes: results from the population-based KORA survey in Germany. Diabet Med. 2013;30(10):1245-9.

39. DeStatis. Statistisches Bundesamt (DeStatis). Verdienststrukturerhebung. 2018. Available from: <https://www.destatis.de/DE/Themen/Arbeit/Verdienste/Verdienste-Branche-Berufe/Publikationen/Downloads/verdienststrukturerhebung-heft-1-2162001189004.pdf?__blob=publicationFile>.

40. DeStatis. Statistisches Bundesamt (DeStatis). Verdienste und Arbeitskosten. 2020. [accessed 6 February 2023]. Available from: <https://www.destatis.de/DE/Themen/Arbeit/Arbeitskosten-Lohnnebenkosten/Publikationen/Downloads-Arbeits-und-Lohnnebenkosten/arbeitskosten-bund-2163201209004.pdf?__blob=publicationFile>.

41. DeStatis. Statistisches Bundesamt (DeStatis). Verbraucherpreisindex nach Zwecken des Individualkonsums. 2023. [accessed 6 February 2023]. Available from: <https://www.destatis.de/DE/Themen/Wirtschaft/Preise/Verbraucherpreisindex/_inhalt.html#249532>.

42. DeStatis. Statistisches Bundesamt (DeStatis). Arbeitskostenindizes nach Quartalen. 2023. Available from: <https://www.destatis.de/DE/Themen/Arbeit/Arbeitskosten-Lohnnebenkosten/Tabellen/_tabellen-quartale-index.html#468268>.

43. Laxy M, Becker J, Kahm K, Holle R, Peters A, Thorand B, et al. Utility Decrements Associated With Diabetes and Related Complications: Estimates From a Population-Based Study in Germany. Value Health. 2021;24(2):274-80.

44. Smith E, Scarborough P, Rayner M, Briggs ADM. Should we tax unhealthy food and drink? Proc Nutr Soc. 2018;77(3):314-20.

45. Mitry P, Wawro N, Six-Merker J, Zoller D, Jourdan C, Meisinger C, et al. Usual Dietary Intake Estimation Based on a Combination of Repeated 24-H Food Lists and a Food Frequency Questionnaire in the KORA FF4 Cross-Sectional Study. Front Nutr. 2019;6:145.

46. Han E, Powell LM. Consumption patterns of sugar-sweetened beverages in the United States. J Acad Nutr Diet. 2013;113(1):43-53.

47. Stasinopoulos MD, Rigby RA, Heller GZ, Voudouris V, De Bastiani F. Flexible regression and smoothing: using GAMLSS in R: CRC Press; 2017.

48. Rigby RA, Stasinopoulos MD, Heller GZ, De Bastiani F. Distributions for modeling location, scale, and shape: Using GAMLSS in R: CRC press; 2019.

49. Willett WC, Howe GR, Kushi LH. Adjustment for total energy intake in epidemiologic studies. Am J Clin Nutr. 1997;65(4):1220S-8S.

50. Wirtschaftsvereinigung Alkoholfreie Getränke. Pro-Kopf-Konsum von Erfrischungsgetränken in Deutschland nach Getränkeart in den Jahren 2012 bis 2021. 2022. [accessed 20 June 2022]. Available from: <https://de-statista-com.eaccess.ub.tum.de/statistik/daten/studie/6200/umfrage/pro-kopf-verbrauch-von-erfrischungsgetraenken/>.

51. Getränke WA. Erfrischungsgetränke bleiben beliebt: Wirtschaftsvereinigung Alkoholfreie Getränke; 2010 [Available from: <http://ernaehrungsdenkwerkstatt.de/fileadmin/user_upload/EDWText/TextElemente/Lebensmittel/Wasser-Getraenke/alkoholfreie_Erfrischungsgetraenke_Trend_Afg_Verband_Feb_2010.pdf>.

52. Bleich SN, Vercammen KA, Koma JW, Li Z. Trends in Beverage Consumption Among Children and Adults, 2003-2014. Obesity. 2018;26(2):432-41.

53. Suen SC, Goldhaber-Fiebert JD, Basu S. Matching Microsimulation Risk Factor Correlations to Cross-sectional Data: The Shortest Distance Method. Med Decis Making. 2018;38(4):452-64.

54. Embrechts P, Lindskog F, McNeil A. Chapter 8 - Modelling Dependence with Copulas and Applications to Risk Management. In: Rachev ST, editor. Handbook of Heavy Tailed Distributions in Finance. 1. Amsterdam: North-Holland; 2003. p. 329-84.

55. Briggs A, Sculpher M, Claxton K. Decision modelling for health economic evaluation: OUP Oxford; 2006.

56. Levin ML. The occurrence of lung cancer in man. Acta Unio Int Contra Cancrum. 1953;9(3):531-41.

57. Lee Y, Mozaffarian D, Sy S, Liu J, Wilde PE, Marklund M, et al. Health Impact and Cost-Effectiveness of Volume, Tiered, and Absolute Sugar Content SugarSweetened Beverage Tax Policies in the United States: A Microsimulation Study. Circulation. 2020;142(6):523-34.

58. Christiansen E, Garby L. Prediction of body weight changes caused by changes in energy balance. Eur J Clin Invest. 2002;32(11):826-30.

59. Swinburn BA, Sacks G, Lo SK, Westerterp KR, Rush EC, Rosenbaum M, et al. Estimating the changes in energy flux that characterize the rise in obesity prevalence. Am J Clin Nutr. 2009;89(6):1723-8.

60. Hall KD, Sacks G, Chandramohan D, Chow CC, Wang YC, Gortmaker SL, Swinburn BA. Quantification of the effect of energy imbalance on bodyweight. Lancet. 2011;378(9793):826-37.

61. Ford ES, Ajani UA, Croft JB, Critchley JA, Labarthe DR, Kottke TE, et al. Explaining the Decrease in U.S. Deaths from Coronary Disease, 1980–2000. N Engl J Med. 2007;356(23):2388-98.

62. Unal B, Critchley JA, Capewell S. Explaining the decline in coronary heart disease mortality in England and Wales between 1981 and 2000. Circulation. 2004;109(9):1101-7.

63. Smolina K, Wright FL, Rayner M, Goldacre MJ. Determinants of the decline in mortality from acute myocardial infarction in England between 2002 and 2010: linked national database study. BMJ. 2012;344:d8059.

64. Young F, Capewell S, Ford ES, Critchley JA. Coronary mortality declines in the U.S. between 1980 and 2000 quantifying the contributions from primary and secondary prevention. Am J Prev Med. 2010;39(3):228-34.

65. Vos T, Lim SS, Abbafati C, Abbas KM, Abbasi M, Abbasifard M, et al. Global burden of 369 diseases and injuries in 204 countries and territories, 1990–2019: a systematic analysis for the Global Burden of Disease Study 2019. Lancet. 2020;396(10258):1204-22.

66. Boshuizen HC, Lhachimi SK, van Baal PH, Hoogenveen RT, Smit HA, Mackenbach JP, Nusselder WJ. The DYNAMO-HIA model: an efficient implementation of a risk factor/chronic disease Markov model for use in Health Impact Assessment (HIA). Demography. 2012;49(4):1259-83.

67. Hawkes C, Jewell J, Allen K. A food policy package for healthy diets and the prevention of obesity and diet-related non-communicable diseases: the NOURISHING framework. Obes Rev. 2013;14 Suppl 2:159-68.

68. Cawley J, Thow AM, Wen K, Frisvold D. The Economics of Taxes on Sugar-Sweetened Beverages: A Review of the Effects on Prices, Sales, Cross-Border Shopping, and Consumption. Annu Rev Nutr. 2019;39:317-38.

69. Roosen J, Staudigel M, Rahbauer S. Demand elasticities for fresh meat and welfare effects of meat taxes in Germany. Food Policy. 2022;106.

70. Deaton A, Muellbauer J. An almost ideal demand system. Am Econ Rev. 1980;70(3):312-26.

71. Moschini G. Units of Measurement and the Stone Index in Demand System Estimation. Am J Agric Econ. 1995;77(1):63-8.

72. Cox TL, Wohlgenant MK. Prices and Quality Effects in Cross-Sectional Demand Analysis. Am J Agric Econ. 1986;68:908-19.

73. Shonkwiler J, Yen ST. Two-Step Estimation of a Censored System of Equations. Am J Agric Econ. 1999;81(4):972-82.

74. Green R, Alston JM. Elasticities in AIDS models. Am J Agric Econ. 1990;72(2):442-5.

75. Lee Y, Mozaffarian D, Sy S, Huang Y, Liu J, Wilde PE, et al. Cost-effectiveness of financial incentives for improving diet and health through Medicare and Medicaid: A microsimulation study. PLoS Med. 2019;16(3):e1002761.

76. Neumann PJ, Ganiats TG, Russell LB, Sanders GD, Siegel JE. Cost-Effectiveness in Health and Medicine. 2nd ed. New York: Oxford University Press; 2016.

77. Husereau D, Drummond M, Augustovski F, de Bekker-Grob E, Briggs AH, Carswell C, et al. Consolidated Health Economic Evaluation Reporting Standards 2022 (CHEERS 2022) Statement: Updated Reporting Guidance for Health Economic Evaluations. Value Health. 2022;25(1):3-9.

78. Koerkamp BG, Stijnen T, Weinstein MC, Hunink MGM. The Combined Analysis of Uncertainty and Patient Heterogeneity in Medical Decision Models. Med Decis Making. 2011;31(4):650-61.

79. Briggs AH, Weinstein MC, Fenwick EA, Karnon J, Sculpher MJ, Paltiel AD, Force I-SMGRPT. Model parameter estimation and uncertainty analysis: a report of the ISPOR-SMDM Modeling Good Research Practices Task Force Working Group-6. Med Decis Making. 2012;32(5):722-32.

80. Busch MA, Kuhnert R. 12-Monats-Prävalenz einer koronaren Herzkrankheit in Deutschland. J Health Monit. 2017;2(1):64-9.

81. Busch MA, Kuhnert R. 12-Monats-Prävalenz von Schlaganfall oder chronischen Beschwerden infolge eines Schlaganfalls in Deutschland. J Health Monit. 2017;2(1):70-6.

82. Heidemann C, Kuhnert R, Born S, Scheidt-Nave C. 12-Monats-Prävalenz des bekannten Diabetes mellitus in Deutschland. J Health Monit. 2017;2(1):48-56.

83. Heidemann C, Scheidt-Nave C, Beyer A-K, Baumert J, Thamm R, Maier B, et al. Gesundheitliche Lage von Erwachsenen in Deutschland – Ergebnisse zu ausgewählten Indikatoren der Studie GEDA 2019/2020-EHIS. J Health Monit. 2021;6(3):3-27.

84. Holstiege J, Akmatov MK, Steffen A, J B. Die ischämische Herzerkrankung in der vertragsärztlichen Versorgung – Zeitliche Trends und regionale Variationen. 2020. DOI: <https://doi.org/10.20364/VA-20.04>

85. Stahmeyer JT, Stubenrauch S, Geyer S, Weissenborn K, Eberhard S. The Frequency and Timing of Recurrent Stroke: An Analysis of Routine Health Insurance Data. Dtsch Arztebl Int. 2019;116(42):711-7.

86. Amies-Cull B, Briggs ADM, Scarborough P. Estimating the potential impact of the UK government's sugar reduction programme on child and adult health: modelling study. BMJ. 2019;365:l1417.

87. Briggs ADM, Scarborough P, Wolstenholme J. Estimating comparable English healthcare costs for multiple diseases and unrelated future costs for use in health and public health economic modelling. PLoS One. 2018;13(5):e0197257.

88. Cobiac LJ, Scarborough P. Modelling the health co-benefits of sustainable diets in the UK, France, Finland, Italy and Sweden. Eur J Clin Nutr. 2019;73(4):624-33.

89. Ananthapavan J, Sacks G, Brown V, Moodie M, Nguyen P, Veerman L, et al. Priority-setting for obesity prevention-The Assessing Cost-Effectiveness of obesity prevention policies in Australia (ACE-Obesity Policy) study. PLoS One. 2020;15(6):e0234804.

90. Carter R, Moodie M, Markwick A, Magnus A, Vos T, Swinburn B, Haby MM. Assessing cost-effectiveness in obesity (ACE-obesity): an overview of the ACE approach, economic methods and cost results. BMC Public Health. 2009;9:419.

91. Barendregt JJ, Van Oortmarssen GJ, Van Hout BA, Van Den Bosch JM, Bonneux L. Coping with multiple morbidity in a life table. Math Popul Stud. 1998;7(1):29-49.

92. Scarborough P, Harrington RA, Mizdrak A, Zhou LM, Doherty A. The Preventable Risk Integrated ModEl and Its Use to Estimate the Health Impact of Public Health Policy Scenarios. Scientifica. 2014;2014:21.

93. Briggs ADM, Cobiac LJ, Wolstenholme J, Scarborough P. PRIMEtime CE: a multistate life table model for estimating the cost-effectiveness of interventions affecting diet and physical activity. BMC Health Serv Res. 2019;19(1):485.

94. Briggs ADM, Wolstenholme J, Scarborough P. Estimating the cost-effectiveness of salt reformulation and increasing access to leisure centres in England, with PRIMEtime CE model validation using the AdViSHE tool. BMC Health Serv Res. 2019;19(1):489.

95. Barendregt JJ, Veerman JL. Categorical versus continuous risk factors and the calculation of potential impact fractions. J Epidemiol Community Health. 2010;64(3):209-12.

96. Cobiac LJ, Law C, Scarborough P. PRIMEtime: an epidemiological model for informing diet and obesity policy. 2022.

97. Picard RR, Cook RD. Cross-Validation of Regression Models. J Am Stat Assoc. 1984;79(387):575-83.

98. Vemer P, Corro Ramos I, van Voorn GA, Al MJ, Feenstra TL. AdViSHE: A Validation-Assessment Tool of Health-Economic Models for Decision Makers and Model Users. Pharmacoeconomics. 2016;34(4):349-61.

99. Tsoi B, Goeree R, Jegathisawaran J, Tarride J-E, Blackhouse G, O’Reilly D. Do different decision-analytic modeling approaches produce different results? A systematic review of cross-validation studies. Expert Rev Pharmacoecon Outcomes Res. 2015;15(3):451-63.

100. Malik VS, Hu FB. The role of sugar-sweetened beverages in the global epidemics of obesity and chronic diseases. Nat Rev Endocrinol. 2022;18(4):205-18.
