# Supplementary material for: Projected health and economic impacts of sugar-sweetened beverage taxation in Germany: A cross-validation modelling study
Source: PLoS Med. 2023 Nov 21;20(11):e1004311. doi: 10.1371/journal.pmed.1004311 (PMC10662751; doi:10.1371/journal.pmed.1004311)
Supplement: Attachment — Submitted filename: PLoS_Med_SSB_Tax_Germany_review_reply_final.docx [file pmed.1004311.s003.docx]

**Point-by-point reply to editorial and peer-reviewer comments for the manuscript entitled “The health and economic impacts of sugar-sweetened beverage taxation in Germany: a cross-validation modelling study” by Emmert-Fees et al.**

Thank you for providing us with the opportunity to revise our manuscript. Please find a point-by-point reply to all editorial and peer-review comments below.

GENERAL COMMENTS

Please cite your Supporting Information as outlined here: https://journals.plos.org/plosmedicine/s/supporting-information

Reply: We have adapted our Supporting Information to follow PLoS Medicine guidelines. Changes were applied throughout the manuscript.

Please ensure to be consistent in the use of the term ‘confidence intervals’ or ‘uncertainty intervals’.

Reply: We have made sure that the terms ‘confidence interval’ and ‘uncertainty interval’ are used correctly, depending on the specific context, in the manuscript and Supporting Information.

Please include the completed CHEERS checklist as Supporting Information. When completing the checklist, please use section and paragraph numbers, rather than page numbers.

Reply: We have included the CHEERS checklist as Supporting Information S2 File.

ACADEMIC EDITOR COMMENTS

I focused on the comments of Reviewer 2. I think they are fair. They might require an appendix be added plus in the text explain the key methods points that are missing. It is very hard to evaluate the results of the simulation without understanding these 3 effects.

Reply: We have addressed all comments from reviewers in detail below. Here we summarize our response to the main themes of feedback from Reviewer #2. For references to changes which were made in the manuscript please refer to the replies to the respective comments from Reviewer #2 directly.

First, we have explained that a lack of access to product-level data unfortunately prohibits us from a) separating the price and reformulation effect in the tiered tax scenario and b) performing a standardized comparison between scenarios. However, we argue that these aspects only minimally impact the conclusions of our analysis, as we aimed to evaluate the overall health and economic effects of sugar-sweetened beverage tax recommendations and an implemented tax design. To appropriately accommodate the provided feedback, we have expanded and edited the manuscript in various sections improving precision in our methodological description, conveying our results more accurately and interpreting our findings with more nuance.

Second, we have thoroughly addressed the insufficient description of price elasticities for beverages in the manuscript and the Supporting Information S1 Appendix. We excluded the reference to the forthcoming paper and instead fully describe all data and methods.

Third, we have clarified and incorporated further major comments from Reviewer #2 regarding the interpretation and reporting of results from the simulated scenarios; the relevance of tax implementation costs; and aspects of tax revenue in different taxation scenarios.

EDITOR-IN-CHIEF COMMENTS

Please ensure that you sufficiently address the price elasticity of SSBs in the main text. In addition, please clearly address the choice of Germany for your modelling study and discuss the representation of the German population in your model, as well as which aspects of economic, policy, and/or health care related issues may be unique to Germany. Please clarify how country-specific characteristics were captured by your model and expand your introduction/discussion on the current political/policy background on SSBs in Germany.

Reply: Thank you for these comprehensive suggestions. We have thoroughly addressed the missing methodological information on price elasticities in the main text (lines 340ff in the marked-up copy) and the corresponding Supporting Information S1 Appendix (pages 42ff). We have further strived to make the choice of Germany more salient to readers in the introduction section of the manuscript. For this we added a respective paragraph on obesity and dietary risk factors in Germany to the introduction (lines 165ff in the marked-up copy). We further added more information on the potentially limited representativeness of the German population in our model with regards to exposure data sources for body mass index and dietary intakes to the discussion section (lines 656ff in the marked-up copy). Country-specific characteristics were captured at all stages of our modeling process by using the most reliable and available context-specific, preferably official, data sources for demographics, exposures, dietary intakes, and disease epidemiology. We have detailed this extensively in the Supporting Information S1 Appendix but slightly adjusted our wording in the methods section to better reflect this (throughout methods section in the marked-up copy). Finally, we improved our description of the German policy context and the political feasibility of the modelled scenarios in the introduction and discussion sections (lines 175ff and 700ff in the marked-up copy).

FINANCIAL DISCLOSURE

The funding statement should include: specific grant numbers, initials of authors who received each award, URLs to sponsors’ websites. Also, please state whether any sponsors or funders (other than the named authors) played any role in study design, data collection and analysis, the decision to publish, or preparation of the manuscript. If they had no role in the research, include this sentence: “The funders had no role in study design, data collection and analysis, decision to publish, or preparation of the manuscript.”

Reply: We have adjusted the Financial Disclosure statement accordingly.

DATA AVAILABILITY STATEMENT

PLOS Medicine requires that the de-identified data underlying the specific results in a published article be made available, without restrictions on access, in a public repository or as Supporting Information at the time of article publication, provided it is legal and ethical to do so. Please see the policy at http://journals.plos.org/plosmedicine/s/data-availability and FAQs at http://journals.plos.org/plosmedicine/s/data-availability#loc-faqs-for-data-policy

The Data Availability Statement (DAS) requires revision. For each data source used in your study:

a) If the data are freely or publicly available, note this and state the location of the data: within the paper, in Supporting Information files, or in a public repository (include the DOI or accession number).

b) If the data are owned by a third party but freely available upon request, please note this and state the owner of the data set and contact information for data requests (web or email address). Note that a study author cannot be the contact person for the data.

c) If the data are not freely available, please describe briefly the ethical, legal, or contractual restriction that prevents you from sharing it. Please also include an appropriate contact (web or email address) for inquiries (again, this cannot be a study author).

Reply: We have expanded the Data Availability section based on PLoS Medicine requirements. Unfortunately, not all used data is freely available, but all used data can be accessed after application to the respective institutions which own the data. We hope that the updated Data Availability Statement fulfills the respective requirements.

ABSTRACT

PLOS Medicine requests that main results are quantified with 95% CIs as well as p values. Please include. When reporting p values please report as p<0.001 and where higher as the exact p value p=0.002, for example. For the purposes of transparent data reporting, if not including the aforementioned please clearly state the reasons why not.

Reply: We have included p-values to all results of statistical analyses which conduct formal hypothesis testing. However, almost all numbers reported in this study are the result of microsimulation modeling without the application of hypothesis testing methods. We therefore did not include p-values for the latter.

Please include any important dependent variables that are adjusted for in the analyses.

Reply: We have not included “adjusted” variables since we do not apply regression modeling techniques.

Throughout, suggest reporting statistical information as follows to improve clarity for the reader “22% (95% CI [13%,28%]; p

Reply: We have adjusted the reporting of statistical information as recommended.

Please note the use of commas to separate upper and lower bounds, as opposed to hyphens as these can be confused with reporting of negative values.

Reply: We have adjusted the reporting of statistical information as recommended.

When a p value is given, please specify the statistical test used to determine it. Please report p values as p<0.001 and where higher as 'p=0.002'.

Reply: We have adjusted the reporting of statistical information as recommended.

Please ensure that all numbers presented in the abstract are present and identical to numbers presented in the main manuscript text.

Reply: We have corrected minor mistakes in the results reporting in the abstract (lines 68-69 in the marked-up copy)and made sure all numbers are identical to numbers presented in the main text.

l.42: Please define ‘UK’.

Reply: We have adjusted this as requested (line 42 in the marked-up copy).

l.60: Please change ‘find’ to ‘found’ and ensure to use the same tense for presenting results.

Reply: We have adjusted this as requested (line 63 in the marked-up copy).

l.62-66: For clarity, please add details about the ad-valorem tax and the tiered tax to the numbers presented in your abstract.

Reply: We have added details about the respective taxes to the numbers presented in the abstract as requested (lines 65ff in the marked-up copy).

l.71: Please define ‘NCDs’.

Reply: We have adjusted this as requested (line 81 in the marked-up copy).

Abstract Methods and Findings:

* Please include the study design, population and setting, number of participants, years during which the study took place, length of follow up, and main outcome measures.

Reply: We have adjusted the abstract accordingly (lines 49ff in the marked-up copy). Due to the design of the study, some aspects such as “number of participants” and “length of follow up” which are typically relevant in the case of observational studies do not apply. Please check whether this is sufficient.

* In the last sentence of the Abstract Methods and Findings section, please describe the main limitation(s) of the study's methodology.

Reply: We have adjusted this as requested (lines 77-78 in the marked-up copy).

Abstract Conclusions:

*Please begin your Abstract Conclusions with "In this study, we observed ..." or similar, to summarize the main findings from your study, without overstating your conclusions. Please emphasize what is new and address the implications of your study, being careful to avoid assertions of primacy.

Reply: We have adjusted this as requested (line 80 in the marked-up copy).

AUTHOR SUMMARY

At this stage, we ask that you include a short, non-technical Author Summary of your research to make findings accessible to a wide audience that includes both scientists and non-scientists. The Author Summary should immediately follow the Abstract in your revised manuscript. This text is subject to editorial change and should be distinct from the scientific abstract. Please see our author guidelines for more information: <https://journals>.plos.org/plosmedicine/s/revising-your-manuscript#loc-author-summary.

The summary should includincludede 2-3 single sentence, individual bullet points under each of the questions.

It may be helpful to review currently published articles for examples which can be found on our website here <https://journals.plos.org/plosmedicine/>

Reply: We have included an author summary as requested (lines 86ff in the marked-up copy). Please check if this is sufficient.

INTRODUCTION

l.76-77 suggest: “In Central Europe, about 27% of premature deaths in 2017 were associated with dietary risk factors.”

Reply: We have adjusted this as requested (lines 139ff in the marked-up copy).

l.90: Please change ‘indicates’ to ’indicated’.

Reply: We have adjusted this as requested (lines 156 in the marked-up copy).

l.107: Please define ‘UK’.

Reply: We have adjusted this as requested (line 161 in the marked-up copy).

l.113: Please define ‘WHO’ at first use (l.87).

Reply: We have adjusted this as requested (line 153 in the marked-up copy).

l.118: Please define ‘BMI’.

Reply: We have adjusted this as requested (line 202 in the marked-up copy).

Please conclude the Introduction with a clear description of the study question or hypothesis.

Reply: We have moved the short description of the modelled scenarios to the first paragraph in the methods section to make the study question more salient in the last paragraph of the introduction section (lines 191ff in the marked-up copy).

Please temper assertions of primacy ("We address this gap by developing the first population health microsimulation model […]”) by adding ‘to the best of our knowledge’ or similar.

Reply: We have adjusted this as requested (line 193 in the marked-up copy).

METHODS AND RESULTS

For sensitivity analyses, please present and justify the choice of method. (Based on Geoffrey P Garnett, Simon Cousens, Timothy B Hallett, Richard Steketee, Neff Walker. Mathematical models in the evaluation of health programmes. (2011) Lancet DOI:10.1016/S0140-6736(10)61505-X.)

Reply: We have adjusted the wording in the section “Uncertainty and sensitivity analyses” (lines 438ff in the marked-up copy). In general, we have followed contemporary guidelines from the International Society for Pharmacoeconomics and Outcomes Research (ISPOR) for the conduct of simulation studies, which give specific details on how to distinguish sensitivity and uncertainty analyses (1. DOI: 10.1177/0272989X12454577; 2. DOI: 10.1177/0272989X12458348). These guidelines further provide a rationale for sensitivity analyses in which analysts vary key inputs in model application to understand their impact on the results. In our case these are primarily related to policy-related assumptions. We therefore have implemented an array of sensitivity analyses which modify aspects of the policy scenarios in the main analysis (e.g., such as tax size of the ad valorem tax or the relevance of substitution to fruit juice). We further implemented additional, alternative scenarios which might be of interest to readers and policymakers (e.g., reformulation based on voluntary industry efforts).

We have further implemented extensive uncertainty analyses via the method of probabilistic uncertainty analyses in a Monte Carlo framework as specified in section “Uncertainty and sensitivity analyses”. Here our model incorporates stochastic and parameter uncertainty from all possible sources as well as individual heterogeneity. Additionally, we assess the structural uncertainty that is related to the choice of the model structure by simulating all analyses with and without direct, BMI-independent effects of SSBs. Finally, we cross-validate our results with a second, independently developed Markov cohort model. We have added the suggested paper as a reference to our manuscript in the section on uncertainty and sensitivity analyses and hope to have clarified this sufficiently.

Please discuss the scientific rationale for this choice of model structure and identify points where this choice could influence conclusions drawn. Please also describe the strength of the scientific basis underlying the key model assumptions. (Based on Geoffrey P Garnett, Simon Cousens, Timothy B Hallett, Richard Steketee, Neff Walker. Mathematical models in the evaluation of health programmes. (2011) Lancet DOI:10.1016/S0140-6736(10)61505-X.)

Reply: We have adjusted the wording in the section “Model overview” to address this comment (lines 233ff in the marked-up copy). In constructing the microsimulation used in our study, we have used decades of epidemiological and medical evidence on the modelled risk factors and disease outcomes, which we repeatedly highlight throughout the manuscript and Supporting Information S1 Appendix (e.g., sections “Model overview” and “Effects of exposures on cardiometabolic risk” in main text; sections “Disease Module” and “Epidemiological engine” in Supporting Information S1 Appendix). We believe that this sufficiently justifies our model structure. Additionally, for model structural aspects which might influence conclusions drawn, we explicitly conduct separate structural uncertainty analyses as shown in Figure 1 and described in sections “Introduction”, “Model overview” and “Uncertainty and sensitivity analyses” in the main text. This is further detailed in the Supporting Information S1 Appendix. Finally, we discuss limitations and quality of all data inputs in Supplemental Table 1 in the Supporting Information S1 Appendix and cross-validate our results with a second independently developed Markov cohort model (PRIMEtime). We highlight these latter aspects also repeatedly throughout the main text and Supporting Information S1 Appendix. We have added the suggested paper as a reference to our manuscript in the section on uncertainty and sensitivity analyses (also see comment above) and hope to have clarified this appropriately.

PLOS Medicine requests that main results are quantified with 95% CIs as well as p values. Please include. When reporting p values please report as p<0.001 and where higher as the exact p value p=0.002, for example. For the purposes of transparent data reporting, if not including the aforementioned please clearly state the reasons why not.

Reply: We have included p-values to all results of statistical analyses which conduct formal hypothesis testing. However, almost all numbers reported in this study are the result of microsimulation modeling without the application of hypothesis testing methods. We therefore did not include p-values for the latter.

Please include any important dependent variables that are adjusted for in the analyses.

Reply: We have not included “adjusted” variables since we do not apply regression modeling techniques.

Suggest reporting statistical information as detailed above – see under ABSTRACT

Reply: We have adjusted the reporting of statistical information as recommended.

Please present numerators and denominators for percentages, at least in the Tables [not necessarily each time they're mentioned].

Reply: We have checked to ensure that we fulfill this requirement. However, we have abstained from including denominators for the relative change in sugar consumption in Table 1 to avoid making the table less accessible and harder to read. However, we also report absolute changes in the same table, which contextualizes relative reductions appropriately in our opinion (lines 495ff in the marked-up copy).

Please ensure to use a consistent tense for presenting results. For example, section 3.3. is written in past tense, whereas the introduction part of the results (3. Results) is written in present tense.

Reply: We have adjusted this as requested.

l.150: Please define ‘US’.

Reply: We have adjusted this as requested (line 214 in the marked-up copy).

l.221. Please change ‘consider’ to ‘considered’.

Reply: We have adjusted this as requested (line 343 in the marked-up copy).

p.46: Please change “In IMPACTNCD Germany The calculation of […]” to “In IMPACTNCD Germany, the calculation of […]”.

Reply: We have adjusted this in the Supporting Information S1 Appendix as requested.

DISCUSSION

Please present and organize the Discussion as follows: a short, clear summary of the article's findings; what the study adds to existing research and where and why the results may differ from previous research; strengths and limitations of the study; implications and next steps for research, clinical practice, and/or public policy; one-paragraph conclusion (Please remove the heading “5. Conclusion).

Reply: We have adjusted this as requested (lines 590ff in the marked-up copy).

Please temper assertions of primacy ("We use the best available epidemiological evidence […]”; “However, we are the first to show […]”, “Our study is the first diet policy evaluation […]”. “We are also the first to quantify the positive cardiometabolic health effects of SSB taxation in […]”. “Our study is the first to model the full health […]”) by adding ‘to the best of our knowledge’ or similar.

Reply: We have adjusted the wording in the respective sections throughout the manuscript as requested.

l.540-l-545: Please clarify and explain the Copyright statement mentioned under ‘Declarations’.

Reply: We have removed the Copyright statement which was part of the manuscript by mistake.

p. 23-25: Please remove the Declarations section from your main manuscript. The data should only be included in the corresponding section in the online submission form. The ethical approval details should be included in the Methods section of your manuscript and in the online submission form.

Reply: We have removed the declarations section as requested (lines 807ff in the marked-up copy).

FIGURES

For all Figures, please ensure that you have complied with our figures requirements <http://journals.plos.org/plosmedicine/s/figures>.

Reply: We have checked the requirements and used the PACE tool to ensure that all figures meet them.

Please consider avoiding the use of red and green in order to make your figure more accessible to those with colour blindness.

Reply: The color palette we used for figures where color is meaningful for interpretation (i.e., Figure 2, Figure 3, and Figure 4) is suitable for color blindness of any kind (<https://cran.r-project.org/web/packages/viridis/vignettes/intro-to-viridis.html>).

For Figures, if applicable, please show the axis beginning at zero. If this is not possible, please show a break in the axis.

Reply: We have adjusted this as requested.

Please in the figure legend/description, define abbreviations used in each figure (including those in Supporting Information files).

Note: The comments related to figure formatting and documentation are addressed collectively below.

Figure 1: Please define ‘∆’ in the figure description.

Figure 2: Please define ‘SSB’.

Figure 2: Please check the statement “Horizontal and vertical error bars indicate 95%-confidence intervals of incremental QALYs and costs per scenario, respectively.”

Figure 2: On the y-axis, the unit used is in “€-billions”, whereas in the main manuscript you refer to “millions”. Please revise.

Figure 3: Please define ‘SSB’.

Figure 4: Please define the meaning of the three SSB taxation scenarios in the figure description or clarify the meaning of ‘extended’ on the y-axis.

Please add figure descriptions to all supplementary figures.

Supplemental Figure 2: Please define ‘GAMLSS’ and ‘KORA’.

Supplemental Figure 4: Please explain the meaning of all lines presented in the figure.

Supplemental Figure 4: For clarity and to avoid confusion due to color, we suggest changing the figure legend to “Source; Observed – dashed line; Modelled – solid line”.

Supplemental Figure 4: Please define ‘ml’, ‘GAMLSS’, ‘KORA’, ‘NVS II’.

Supplemental Figure 6: Please define ‘GAMLSS’ and ‘ml’.

Supplemental Figure 7: Please define ‘GAMLSS’.

Supplemental Figure 8: Please explain the meaning of all lines presented in the figure.

Supplemental Figure 8: For clarity and to avoid confusion due to color, we suggest changing the figure legend to “Source; Observed – dashed line; Modelled – solid line”.

Supplemental Figure 8: Please define ‘ml’, ‘GAMLSS’, ‘KORA’, ‘NVS II’.

Supplemental Figure 10: Please define ‘GAMLSS’ and ‘ml’.

Supplemental Figure 11: Please define ‘GAMLSS’ and ‘ml’.

Supplemental Figure 11: Please add a unit (years) for the age groups and change the description from “(blue dashed lines)” to “(blue lines)”.

Supplemental Figure 12: Please define ‘GAMLSS’ and ‘ml’.

Supplemental Figure 12: Please change the description from “(blue dashed lines)” to “(blue lines)”.

Supplemental Figure 13: Please define ‘GAMLSS’ and ‘ml’.

Supplemental Figure 13: Please add a unit (years) for the age groups and change the description from “(blue dashed lines)” to “(blue lines)”.

Supplemental Figure 14: Please define ‘GAMLSS’ and ‘ml’.

Supplemental Figure 14: Please change the description from “(blue dashed lines)” to “(blue lines)”.

Supplemental Figure 15: Please define the meaning of the black dashed line and add a unit for age.

Supplemental Figure 16: Please define ‘∆’ in the figure description and change “BMI-independetn healthe effects” to “BMI-independent health effects”.

Supplemental Figure 17: Please define ‘KORA’ and add a unit for age. Please define the meaning of the dots and lines in the graphs.

Supplemental Figure 19/20/21/22/23/24: Please add a unit for age (age groups).

Supplemental Figure 25/26/27/28/29/30: Please change groups from ‘Women’ and ‘Men’ to ‘Female’ and ‘Male’. Please add a unit for age and add ‘rate’ on the y-axis (incidence/prevalence rate).

Supplemental Figure 31/32/34/35/38/39: Please add a unit for age.

Supplemental Figure 33: For clarity, please add the years to the x-axis on the upper graph (KV 2013-2018). Please add a unit for age.

Supplemental Figure 34: Please add a unit for age.

Supplemental Figure 36/37: Please add a unit for age. Please define ‘AOK’.

Supplemental Figure 41: Please change groups from ‘Women’ and ‘Men’ to ‘Female’ and ‘Male’.

Supplemental Figure 42/43: Please change groups from ‘Women’ and ‘Men’ to ‘Female’ and ‘Male’.

Supplemental Methods 2: Please define ‘NCD’, ‘BMI’, ‘CHD’, ‘QALY’.

Supplemental Methods 3: Please define ‘CHD’, ‘QALY’.

Reply: We have adhered to all the above comments related to figure formatting and adjusted the figures as requested. We have further fixed minor errors in figure formatting to improve consistency.

Supplemental Figure 5: Please define ‘GAMLSS’ and ‘ml’. Please add ‘Age, years’ as a description/unit to the age groups presented on the left side of the figure.

Reply: In our opinion, adding a description/unit for age on the left side of the figure would decrease interpretability. We have thus added a clear description to the figure title and legend.

Supplemental Figure 9: Please define ‘GAMLSS’ and ‘ml’. Please add ‘Age, years’ as a description/unit to the age groups presented on the left side of the figure.

Reply: In our opinion, adding a description/unit for age on the left side of the figure would decrease interpretability. We have thus added a clear description to the figure title and legend.

Supplemental Figure 18: Please ensure to add the reason(s) for record exclusion.

Reply: Unfortunately, we did not document reasons for exclusion at the first stage of screening by title and abstract of the identified records.

TABLES

Please note the use of commas to separate upper and lower bounds, as opposed to hyphens as these can be confused with reporting of negative values. Suggest reporting statistical information as detailed above – see under ABSTRACT

Reply: We have adjusted this as requested.

Please define all abbreviations used in the table below each table (including those in Supporting Information files).

Note: The comments related to table formatting and documentation are addressed collectively below.

For clarity, please add the exact currency (€) to cost-related numbers (€-millions). Please revise throughout your entire manuscript.

Table 1/2: In your table legend, please define the meaning of the three SSB taxation scenarios (at minimum, please define ‘20% extended ad valorem tax’).

Supplemental Table 1: Please define all abbreviations used in the table (e.g., KORA, NVS II) below the table.

Supplemental Table 3: Please define ‘SSB’.

Supplemental Table 4: Please define ‘KORA’, ‘NVS II’ and ‘n/a’.

Supplemental Table 5: Please define ‘KORA’, ‘NVS II’ and ‘n/a’.

Supplemental Table 6: Please define ‘Y’.

Supplemental Table 7: Please define ‘n/a’ and add a unit for age.

Supplemental Table 15b: Please define ‘CHD’.

Supplemental Table 16: Please add a unit for age. Please change the categories under “Sex” from ‘Women’ and ‘Men’ to ‘Female’ and ‘Male’.

Supplemental Table 17: Please add a unit for age. Please change the categories under “Sex” from ‘Women’ and ‘Men’ to ‘Female’ and ‘Male’. Please add a unit (€) to all cost-related numbers.

Supplemental Table 18: Please change the categories from ‘Women’ and ‘Men’ to ‘Female’ and ‘Male’ and add details about the age groups (category and unit). Please define ‘Juice’, ‘ml’, ‘20% extended ad valorem tax’.

Supplemental Table 19/20: For clarity, please add the exact currency (€) to cost-related numbers (€-millions).

Supplemental Table 21: For clarity, please add the exact currency (€) to cost-related numbers (€-millions). Please define ‘20% extended ad valorem tax’.

Supplemental Table 22: Please add a unit for age and costs. Please define ‘20% extended ad valorem tax’.

Supplemental Table 23: Please add a unit for costs. Please change the categories from ‘Women’ and ‘Men’ to ‘Female’ and ‘Male’. Please define ‘20% extended ad valorem tax’. Please remove ‘ICER’ from the abbreviations.

Supplemental Table 24: Please add a unit for costs. Please define ‘20% extended ad valorem tax’ and ‘BMI’. Please remove ‘ICER’ from the abbreviations.

Supplemental Table 25: Please define ‘20% extended ad valorem tax’.

Reply: We have adhered to all the above comments related to table formatting and adjusted the tables as requested.

Supplemental Table 12: Please define ‘EQ-5D-5L’

Reply: “EQ-5D-5L” is a name and not an abbreviation. See: <https://euroqol.org/eq-5d-instruments/>. We have therefore not changed this.

REFERENCES

PLOS uses the numbered citation (citation-sequence) method and first six authors, et al.

Please ensure that journal name abbreviations match those found in the National Center for Biotechnology Information (NCBI) databases (http://www.ncbi.nlm.nih.gov/nlmcatalog/journals), and are appropriately formatted and capitalised.

Please also see https://journals.plos.org/plosmedicine/s/submission-guidelines#loc-references for further details on reference formatting.

Where website addresses are cited, please specify the date of access.

Reply: We have checked that references are formatted correctly.

Supplemental References: RE reference [66], Supplementary Material, listed as under review, papers cannot be listed in the reference list until they have been accepted for publication or are otherwise publicly accessible (for example, in a preprint archive). The information may be cited in the text as a personal communication with the author if the author provides written permission to be named. Alternatively, please provide a different appropriate reference.

Reply: We have removed reference #66 from the Supporting Information S1 Appendix. The corresponding information is now included directly in the Supporting Information S1 Appendix (page 42ff).

Comments from the reviewers:

Reviewer #1: Recognizing a gap in context-specific research, the authors aimed to develop the first population health microsimulation model for Germany, termed IMPACTNCD Germany. This model assessed the potential impact of different SSB taxation on sugar consumption and thus on diseases like stroke, coronary heart disease, and type 2 diabetes, considering morbidity, mortality, healthcare costs, patient time, and productivity costs. Overall, this is a well-designed and conducted study. The methods of the study are rigorous to me, and the results are very useful to inform policies of SSB taxation in Germany. Below are my specific comments.

Reply: Thank you for your kind words and the constructive feedback.

1. The data are not fully publicly available, which may not meet PLoSMED's requirement.

Reply: Thank you for pointing this out. We have expanded the Data Availability Statement based on PLoS Medicine requirements (also see editorial comments above). Unfortunately, not all used data is freely available, but all used data can be accessed after application to the respective institutions which own the data.

2. Line 90: it could be helpful to provide brief introduction of "tiered structure" in the introduction. Not all readers are familiar with this term.

Reply: We have introduced the “tiered structure” in the introduction (lines 158ff in the marked-up copy).

3. Line 76-120: The introduction does a great job of establishing the importance of dietary risk factors in premature deaths and the role of SSBs. However, the connection between SSBs and dietary risk factors could be better emphasized.

Reply: Thank you for this suggestion. We have slightly expanded the introduction to better link sugar-sweetened beverages and dietary risk (lines 142ff in the marked-up copy).

4. Line 76-120: While the authors point out that previous studies did not quantify healthcare cost savings or productivity costs, they could go further in explaining why their approach is novel or important. For instance, what is the specific benefit of using a microsimulation model for Germany? Why are these three policy scenarios chosen for simulation?

Reply: Thanks for raising this point. We have taken the opportunity to clarify the novelty of our study and aimed to formulate the respective sections more concisely (lines 181ff in the marked-up copy).

5. Line 184: "..the distribution of BMI.." Do you mean the distribution of SSB consumption here? The surveys you described in this paragraph were all about dietary consumption not about anthropometric measurements.

Reply: We have checked that our wording is correct. We refer here to body mass index (BMI), which is one of the main exposures of our study. BMI is measured in the surveys used to construct the synthetic population and introduced one paragraph earlier under the subheading “Synthetic German population”. The *Kooperative Gesundheitsforschung in der Region Augsburg* (KORA) survey is used for both anthropometric measures and dietary consumption as indicated in the paragraph “Synthetic German population”.

6. Line 198-199: I think the Global Burden of Disease Study (the GBD) should have better incidence estimates for stroke and CHD for Germany.

Reply: Thank you for this suggestion. In preparation of our study, we have considered Global Burden of Disease (GBD) data to inform estimates of stroke and CHD incidence. However, data from GBD is itself based on extensive modeling which fills missing data with estimates from comparable countries, thus not necessarily capturing the country-specific epidemiological context of each disease. This may lead to suboptimal calibration of GBD to other nationally available epidemiological data (see for example: <https://www.ncbi.nlm.nih.gov/pubmed/27539806>). Additionally, members of the author team had similar experiences when modelling health interventions in other countries. After extensive discussions among the author team, weighing the advantages and disadvantages of using GBD data, we therefore decided to use the best available national data instead. This process ultimately led us to estimate context-specific CHD and stroke incidence rates using the SCORE2 equations as described in the Supporting Information S1 Appendix to our study. We hope to have clarified this appropriately.

7. Line 223-224: was this assumption based on the reference cited here? Should make it clear how the assumption is made.

Reply: You are correct in that this assumption was based on the cited reference. We have made this clearer now (lines 365ff in the marked-up copy).

8. Line 234: what do you mean by conservative aetiologic effects here? Lower bound of the 95% CI? Need to be more specific.

Reply: Thank you for this comment, which we gladly clarify. We have adjusted the respective wording to be more concise (lines 383ff in the marked-up copy). To explain further, “conservative aetiologic effects” were here used to indicate that the applied estimate of a long-term change in BMI based on changes in sugar consumption is from observational studies and more conservative than long-term changes in weight modelled by e.g., energy balance equations. To make the implications of the applied method of long-term body weight reduction more salient to readers, we have added example calculations to the table description of Supplemental Table 7 in the Supporting Information S1 Appendix (also see comments of Reviewer 4).

9. Line 285: I'm still not sure whether discounting QALYs is a good idea.. To me A QALY should equal a QALY no matter whether that is now or 20 years from now.

Reply: Thank you for raising this point. While we are aware of ongoing debates in health economics whether quality-adjusted life years (QALYs) should be discounted, we followed recommendations from the second panel on cost-effectiveness in health and medicine and guidance from the German Institute for Quality and Efficiency in Health Care (IQWiG, the national commissioned health technology assessment body), that suggest to discount QALYs. Additionally, we provide sensitivity analyses with different discount rates for costs and QALYs to reflect that different discount rates might be appropriate.

Reviewer #2: Thanks for allowing me to review the paper entitled "The health and economic impacts of sugar-sweetened beverage taxation in Germany: a cross-validation study". I want to highlight the solid specification of the epidemiological model due to the impressive validation process by the authors by calibrating this model according to observed health information. Due to the strength of this epidemiological model, most of my comments are related to the economic aspects of the SSB tax assessments. I hope the authors find these comments relevant to strengthen the study.

Reply: Thank you for your comments and the constructive feedback, which has strengthened our work further.

Major comments

The main text does not offer enough information to understand/identify the different tax burdens depending on the sugar-content threshold under the tiered tax. For example, what is the expected price increase with/without reformulation under the tiered tax in Germany? For the tiered tax, could the authors explain how they isolated the tax-related reformulation effect from the tax-related price effect?

Reply: Thank you for these comments. Unfortunately, we do not have access to product-level purchasing data of the SSB market in Germany. Therefore, our analysis of different tax design, and especially with regards to the tiered tax, is limited. As a consequence, we have to rely on price elasticities for the ad-valorem tax scenarios and an observed reformulation effect from the United Kingdom (UK) in case of the tiered tax scenario. This means that we can model the tiered tax only indirectly and are further unable to compare specifications of different sugar thresholds which would have been highly interesting.

We therefore are only able to model a tiered tax that would lead to similar reformulation effects as observed in the UK without specifying the respective thresholds or price increases that would lead to such an effect. To make these assumptions more transparent, we have expanded the respective section in the section “Sugar-sweetened beverage taxation scenarios” (lines 252ff in the marked-up copy) and restructured and expanded the section “Effect of SSB taxation scenarios on sugar intake” (lines 329ff in the marked-up copy). For the same reason, we are unfortunately not able to isolate the tax-related reformulation from the tax-related price effect.

However, this is not a problem in our application, as we are interested in the total effect of each SSB taxation scenario. Further information on our rationale is available under Supplemental Methods 1: Policy Module in the Supporting Information S1 Appendix. To improve transparency, we also added this as a specific limitation (lines 671ff in the marked-up copy). We hope to have clarified these points sufficiently.

Moreover, the current approach does not allow a "standardized" comparison between the 20% ad valorem tax and the tiered tax. Thus, it might not be possible to conclude if the higher health effects under the tiered tax are because of reformulation or more significant price increases. I recommend that the authors review the paper by Salgado and Ng (2021) where the authors make volumetric and sugar taxes comparable before reformulation so that it is possible to see the potential health effect linked to reformulation.

Reply: Thank you for raising these important points. We agree that a standardized comparison between our modelled scenarios is not possible and that this question is very relevant. We have reviewed the suggested manuscript by Salgado and Ng (2021) with great interest. However, a detailed comparison and standardization of different SSB tax scenarios similar to Salgado and Ng (2021) is unfortunately not possible without more granular product-level purchasing data to which we do not have access. However, we agree that using German product-level data to model the comparative effectiveness of tax designs in more detail together with our microsimulation is a highly relevant avenue for future research.

Further, we think such an analysis would be beyond the current scope of our study in which we aimed to pragmatically compare SSB taxation scenarios in Germany based on global recommendations and the observed effects of implemented SSB tax designs. In this sense, our aim was not to decompose the health effects in the tiered taxation scenario in reformulation and price effects, but rather to compare the total effects of different internationally enacted taxation approaches in the best context-specific way possible (e.g., ad-valorem tax via national price elasticities and tiered tax via observed reformulation effects). Additionally, based on the study (<https://www.ncbi.nlm.nih.gov/pubmed/36809753>) we use as a source for the reformulation effect in the tiered tax scenario, we assume that sugar-sweetened beverage consumption would not change. This implies only a minor or irrelevant price change in the tiered tax scenario.

To be precise and to avoid confusion, we have further checked our manuscript to make sure that conclusions about the relative effectiveness of different tax designs are phrased moderately. We also have added this as a specific limitation (lines 671ff in the marked-up copy).

Authors gather information on price elasticities from a "forthcoming" paper (see reference #45). Estimations of price elasticities are a demanding task by itself. Thus, if the relevant paper is still under review, estimates of price elasticities can change due to reviewers' comments. Thus, I recommend that the authors use published evidence either from a meta-analysis or another paper for Germany. Or is this point already covered under the sensitivity analysis of "implementing the effect of taxation using a meta-analytic estimate? If so, it is important to highlight the remarkable difference (i.e., 25%) compared to their preferred model specification.

Reply: Thank you for this comment. Within the larger project of using simulation modeling to evaluate diet policies in Germany, we identified a lack of price elasticities for non-alcoholic beverage categories in Germany. For this reason, we included an experienced economist in our team with whom we implemented the analysis for the paper in reference #45, aiming to publish it separately.

We agree that consequently the methods used in our analysis to estimate beverage price elasticities were not reported sufficiently transparent in the manuscript or its Supporting Information S1 Appendix. We have thus excluded reference #45 and instead included all methodological details on the elasticity estimation in the Supporting Information S1 Appendix (page 42ff). We further included specific information on the estimated elasticities in the main text (lines 345ff in the marked-up copy). This allows for full transparency of the applied methods.

The sensitivity analysis “implementing the effect of taxation using a meta-analytic estimate” indeed uses a published SSB own-price elasticity estimate based on observational studies which has been used in other international SSB tax modeling studies. Further information on this sensitivity analysis is provided in Supplemental Table 10 in the Supporting Information S1 Appendix.

The difference between this meta-analytic estimate and our own context-specific estimate is indeed large. However, we believe that our national price elasticity estimates, which were produced using established economic modeling techniques and national representative data, are more relevant for our study. Further, our estimated national elasticities are very similar to other reported SSB own-price elasticity estimates such as in Andreyeva et al. (10.1001/jamanetworkopen.2022.15276).

Regarding the epidemiological model, can a cohort member of the microsimulation get two or more diseases?

Reply: We are happy to clarify this question. Simulants in the microsimulation are tracked through their whole individual life course and can indeed acquire more than one of the modelled diseases. To reflect this, we do not only model exposures (e.g., BMI) as risk factors for diseases, but also diseases as risk factors for diseases (e.g., type 2 diabetes increases the risk for stroke). We hope to have clarified this sufficiently.

Could the authors include estimates of sugar reductions in Table 1 as percentage reductions? This information can be policy-relevant due to a direct interpretation of the tax effectiveness is in terms of percentage reductions.

Reply: Thank you this suggestion. We have included percentage reductions in Table 1 accordingly (lines 495ff in the marked-up copy).

There is a marked reduction (i.e., five times larger) in sugar consumption from SSB and juice when comparing scenarios 1 and 2 in Table 1. Could the authors provide some insights to understand this marked difference between scenarios? For example, is it a result of a high baseline consumption of juices in Germany? Is it a result of SSB and juices being complements?

Reply: Thank you for this question. You are correct that this is due to a very high level of juice consumption in Germany. Using our estimated national elasticities, this leads to a large reduction in beverage consumption and thus sugar intake from fruit juice and sugar-sweetened beverages. Further details, a comparison with different data sources and a discussion of the implications on the results of our study are described in Supplemental Methods 1: Epidemiological Engine – Exposure module.

While Table 1 shows that the "20% extended ad valorem tax" leads to a sugar consumption reduction that more than doubles the reduction under the tiered tax, table 2 shows, in general, larger health effects under the latter tax. These results might seem counterintuitive because we can expect larger health effects under policies that lead to higher sugar reductions, which would be the "20% extended ad valorem tax" in this paper. Could the authors explain this counterintuitive result?

Reply: We are happy to explain these counterintuitive results. In our study we also analyze the relevance of BMI-independent, direct effects of SSBs on health. Likewise, we do not assume that there are direct effects of fruit juice as described in the paragraph “Effects of exposures on cardiometabolic risk”. This leads to larger health gains under the tiered tax than under the extended ad-valorem tax in the main analysis (which includes direct effects of SSBs), despite higher sugar reductions in the latter. We make the resulting uncertainty explicit by re-estimating all analyses without BMI-independent, direct effects of SSBs, which we show in Figure 2 in the main manuscript. We have made sure to report and discuss these results more explicitly and precisely in the manuscript (lines 570ff, 630ff, 714f in the marked-up copy)

According to my best understanding, the authors solely focused on health savings overlooking any cost linked to the tax implementation. Other scholars have included costs. For example, see the papers by (Basto-Abreu et al., 2019; Wilde et al., 2019). Could the authors include any costs linked to the tax implementation?

Reply: Thank you for this comment. We have explicitly not included tax implementation costs as these are hard to quantify, although others have attempted this as you rightfully point out. However, we agree that a discussion of this aspect, specifically with regards to different tax designs, was lacking and have expanded the discussion section accordingly (lines 678ff in the marked-up copy).

A downside for sugar taxes that encourage reformulation is a potential reduction in tax revenue (see findings by Salgado and Ng(2021)). While I do not recommend including tax revenue as part of the cost-effectiveness analyses (because it is a benefit for the government but a cost for consumers), I think it might be helpful to discuss this potential tax revenue reduction as a relevant outcome for the government when weighing benefits and costs across different tax designs.

Reply: Thank you for this suggestion. We agree that a reflection on tax revenue implications of the different scenarios is currently lacking and have expanded the discussion section accordingly, referring to your suggested study (lines 704ff in the marked-up copy).

Minor comments

In the abstract, the tax in Mexico corresponds to 1.00 peso/liter rather than 0.01 peso/liter.

Reply: Thank you for spotting this mistake. We have adjusted the abstract accordingly (line 41 in the marked-up copy).

The authors highlight that their tax-design selection is "based on internationally implemented policies" (see line 498). While this is clear in the case of the tiered tax, which corresponds to the design in the UK, it is not obvious which country is the reference for the 20% tax.

Reply: Thank you for this comment. We have mentioned our rational for choosing the tax designs in the last paragraph of the introduction section (now under Model overview) but agree that this was inconsistently specified throughout the rest of the main text. We have worded these and similar instances throughout the manuscript more carefully to better reflect that the reference for the 20% tax were international scientific recommendations in conjunction with implemented taxes that aimed to follow these recommendations (lines 209ff, 260ff and throughout manuscript in the marked-up copy).

It would be policy-relevant to see a clear justification regarding the selection of tax designs for main scenarios 2) and 3). For example, why did the authors prefer a sugar-tax design as in the UK rather than the sugar tax as in South Africa (which has been associated with a sugar reduction larger than 50% (Stacey et al., 2021))?

Reply: Thank you for this comment. We aimed to select few tax designs that would be most relevant for the German policy context and which we could model with the data we have access to. We therefore opted 1) to estimate ad-valorem taxation based on recommendations from WHO, which many countries that actually implemented SSB taxes followed, that SSB taxes should at least aim to increase prices by 20% (this was also the original target of the South African Health Promotion Levy as mentioned in Stacey et al., 2021); 2) to extend this ad-valorem tax to fruit juice due to the high baseline fruit juice consumption in Germany; and 3) to investigate the SDIL which has been thoroughly evaluated and was implemented in an European economy roughly similar to Germany and might serve as a blueprint for policymakers. We have aimed to make these justifications more precise in the sections “Modelling overview”, “Sugar-sweetened beverage taxation scenarios” and “Effect of SSB taxation scenarios on sugar intake” (lines 209ff, 260ff and 330ff in the marked-up copy).

For Table 2, could the author confirm whether uncertainty intervals (UI) correspond to 95% UI? If so, please include this information as part of the footnote.

Reply: Thank you for spotting this. We have added the respective information to Table 2 (line 507 in the marked-up copy).

Reviewer #3: Dear Authors

Thank you for very well written study. It is one of the best-documented study in the public health literature that I have had the opportunity to review.

I do have a few questions for my own clarification and comments for your kind consideration.

Reply: Thank you for this positive review and the constructive comments.

1. The study has modeled trend in BMI using three surveys. As I am not familiar with public policies in Germany, I would like to know whether there were any obesity/DM related campaigns in Germany during the time covered by these surveys, such that the trends might be affected by it. For example, smoking trend in absence of government push for reduction of smoking would be different as compared to a landmark report about negative consequences of smoking. Similarly if there were school-based campaigns to reduce SSB advertising and consumption in/around school-children, then the entire base of population entering the cohort-simulation model will have different BMI distribution. The external validity of the procedure to determine trends for BMI will depend on this.

Reply: Thank you for this question. To the best of our knowledge there were no large-scale public health campaigns or fundamental governmental nutritional interventions, specifically for sugar-sweetened beverages, to the degree that these might have influenced body mass index (BMI) trends in Germany. However, overall trends in BMI are likely captured in the used surveys and reflect overall changes in behavioral risk factors related to BMI. While it is important to understand what might affect observed BMI trends, this has no relevant implications for our microsimulation model since we want to indeed replicate the observed risk factor trends in the German population. We hope to have clarified this sufficiently.

2. Inflation: Page 44 indicates that CPI-Health has been used to adjust for inflation in health-care sector. Pardon me, but I could not find information on whether the authors have adjusted the prices of SSBs and fruit-juices for inflation over the 20-year time horizon. My reasons for asking are twofold: First - in ad-valorem scenario - the absolute quantum of tax increases if there is inflation and recent macroeconomic data suggests higher food inflation due to Ukraine-Russia conflict. The quantum of tax itself in this case acts as automated brakes on consumption (as compared to specific tax which declines as proportion of price if there is rapid inflation). Second, the market structure of SSB vis-a-vis fruit juice industry is likely to affect the product-specific inflation. In my country, SSBs are manufactured/sold by firms in oligopoly tending towards duopoly structure whereas fruit-juice market is much more diverse. In face of general inflation, the ability of the dominant market players to maintain prices to gain market share is likely to happen for SSBs than for fruit juices. Hence, my question about how did authors address these diverse issues of market structure and inflation for specific products.

Reply: Thank you for this comment. In our microsimulation model, we do not include beverage price inflation because we do not directly observe current individual product prices due to lack of access to product-level data (also see replies to comments of Reviewer #2). We therefore use price elasticities (in scenarios 1 and 2), which translate proportional changes in price to proportional changes in consumption. In this way, taxes are converted to consumption. Due to a lack of nutritional time series data, we are only able to model population level trends due to demographic changes over the time horizon (i.e., consumption depends on age and sex). We extensively describe this in the main text and the Supporting Information S1 Appendix while acknowledging the resulting limitations.

Indeed, the health gains from an ad-valorem tax may be greater if a secular trend of inflation in beverage prices is already leading to reduced consumption. It is important to emphasize that the tax in scenarios 1 and 2 is specified as a relative change. In our analysis including inflation would thus be equivalent to an additional “tax” which would further reduce consumption. This makes our results conservative considering current food price inflation. We have discussed this in the discussion section of our manuscript in the paragraph describing study limitations. Further, although we did not include inflation of beverage prices, we also did not include wage inflation which may counteract the described effects somewhat if disposable income keeps up with beverage prices. We implicitly also assume that the incentives of the tiered tax (scenario 3) would stay stable. This implies that the tiered tax amounts would be uprated periodically by the government if prices increase substantially.

We included many other forms of uncertainty in the modelling in terms of tax pass-through rate, price elasticity of demand, and relationship between SSB consumption and health outcomes which may have a larger effect on the results. Despite not including aspects of market structure and inflation, we believe the results are still robust.

3. Market structure and political feasibility. I understand the study documents the costs/benefits of 3 scenarios. As a reader I would be interested to know the political feasibility and interest in implementing (a) ad-valorem vs (c) taxes/incentives for product-reformulation. Again I am not familiar with German context and hence general audience would benefit from authors' discussion of these issues. Developing countries or governments with lower bargaining power as compared to industry would prefer (a) as it signals to the politic that action has been taken, provides stable revenue; while other countries might prefer (c). Could the authors' discuss whether (a) or (c) is more likely to be feasible in their country?

Reply: Thank you for this suggestion, which we have gladly expanded on in the discussion section (lines 700ff in the marked-up copy).

4. Elasticity estimates. Page 93 indicates estimation of own- and cross-price elasticity estimates. Were these calculated separately by age- gender- or age-by-gender groups or did the authors calculate a single elasticity estimate? If a single elasticity estimate was calculated did the authors apply it uniformly to all age-gender groups or did they scale it different factors to take into account consumption preferences?

Reply: Thank you for this question. We have used marginal unstratified elasticities because the estimated elasticities were based on household consumption data which does not easily allow further stratification by age and sex. Additionally, scaling elasticities based on various factors assuming to affect consumption preferences is not trivial and beyond the scope of this study. We have, however, acknowledged this as a limitation of our study (lines 665ff in the marked-up copy).

Reviewer #4: Overall comments

Thank you for the opportunity to review this manuscript. This is an exceptionally thorough (due to all the Supplementary Material provided) and impressive piece of work that is an important contribution to the SSB tax academic literature. The study examines the potential impacts of an SSB tax in Germany, a country that could potentially benefit, and policymakers would also likely read this paper with great interest. There are also highly detailed methods that could benefit researchers.

The main findings of the paper are that (1) implementing an SSB tax could have health and economic benefits in Germany, and (2) a tiered tax was modelled to be more effective (of the tax structures considered). It is a strength that the authors explored a few possible SSB tax structures, which allows for even stronger policy recommendations by comparing their relative modelled impacts. However, it should be noted there are other possible tax structures, such as the South Africa Health Promotion Levy, which taxes based on sugar concentration, and is different from the UK SDIL tiered tax.

I have few critiques as many of the questions I had were answered in the Supplementary Materials.

Reply: Thank you for your feedback and the constructive comments.

Specific comments

Abstract

Lines 67-68: If word count is not an issue, it could help the reader understand the magnitude of the difference between including/excluding fruit juice by putting the absolute or relative difference in the findings between the two approaches. The same could be done for analyses excluding direct health effects of SSBs.

Reply: Thank you for this suggestion. We have weighed the possibility to include the suggested results in the abstract considering editorial requirements that all numbers presented in the abstract must be present identically in the main text. Including these numbers in the abstract would necessitate the addition of another table in the main text, which we think might make the manuscript less focused. We have therefore decided to report key results for the ad-valorem tax scenario including fruit juice, which are already present in the main text, in the abstract as well (lines 74ff in the marked-up copy). However, we had to compromise and decided against including quantitative results for the analysis excluding direct health effects of SSBs in the abstract because we think this would unnecessarily inflate abstract word count further.

Introduction

Clear and concise Intro. Provides a strong rationale for the study and its potential use in informing policy.

Reply: Thank you!

Methods

Lines 156-165: Although it's possible it is mentioned somewhere, it was not clear to me why an effort was clearly made to simulate an ad valorem tax with and without fruit juice, but fruit juice is not discussed in the context of a tiered tax. I see that the assumption made is simply a 30% reduction in sugar consumption across the board, without changing SSB intake. I still think for clarity it could be worth mentioning whether the 30% reduction does/does not include fruit drinks (with added sugar). As you've defined terms, it would seem not. Presumably if this does not include fruit drinks (with added sugar that could be reformulated), then including them along with other SSBs could lead to an even larger impact, given the impact was greatly increased in the ad valorem scenario + fruit drinks. This is very minor, but it seems worth explaining to fully understand how the tiered scenario compares to the other two.

Reply: Thank you for this comment. Our definition of sugar-sweetened beverages indeed includes fruit drinks which thus would be affected by the 30% reduction due to reformulation. We have specified this in the section “Sugar-sweetened beverage taxation scenarios”.

However, we are unfortunately not able to distinguish 100% fruit juice (which does not contain added sugars) and nectars (which might contain added sugars). Generally, nectars are only a small proportion of juice consumption in Germany, though (Reference 50 in the Supporting Information S1 Appendix; requires Statista access). We thus consider the resulting bias very small. We have added this information to the section “Sugar-sweetened beverage taxation scenarios” (lines 253ff in the marked-up copy). Moreover, fruit juice is not discussed in context of a tiered tax because reformulation is not relevant for fruit juice without added sugars. We hope to have clarified this sufficiently.

Lines 232-236: can you add the quantitative details for the "conservative aetiologic effect estimates" (i.e. why it is considered conservative) and "predicted reductions in weight are generally lower than using traditional energy balance equations." As an example for comparison, the above quotation from Lines 213-214 was easy to follow as it included the assumption for price pass through: "We assumed that the proportion of the SSB price increase passed on to consumers was 82% (66 to 98) based on a recent meta-analysis [4]." Some of the referencing without including quantitative details requires the reader to do a lot of tracking down of references or tables elsewhere in the document. However, I did find Supplementary Table 7 helpful in answering a lot of these questions.

Reply: Thank you for this comment. Unfortunately, many of the calculations in the process of our study involve complex steps and weighing of different approaches. This makes a full presentation of all quantitative information in the main text infeasible in our opinion and would decrease the readability of the manuscript. However, to make the applied method of long-term body weight reduction more salient to readers, we have added example calculations to the table description of Supplemental Table 7 in the Supporting Information S1 Appendix.

Line 266: please also provide quantitative details for "medical costs for the treatment of stroke, CHD, and T2DM."

Reply: Thank you for this suggestion. We have considered how quantitative details for the medical costs could be accommodated in the main text. However, we use highly stratified information, which is infeasible to be conveyed in the main text. We show all quantitative details of our health economic analysis in Supplemental Table 16 in the Supporting Information S1 Appendix.

Results

Lines 392-393: I understand the results are all available in Tables referenced, but this description ("smaller, however still large") is too vague. I would suggest adding quantitative details regarding how much smaller, or why it should still be considered large.

Reply: Thank you for this suggestion. We have extended the section reporting on results from the analysis excluding direct effects of SSBs to provide more quantitative information (lines 570ff in the marked-up copy). We additionally ensured a more careful wording.

Discussion

Line 413: "(~€9,600 to ~€16,000) over the next 20 years" are you missing the word "million" as was included in line 412? It might be implied but seems clearer to include.

Reply: Thank you for your spotting this. We adjusted based on your suggestion (line 598 in the marked-up copy).

Lines 486-488: In the section on future research, there are useful suggestions. Could you please add more detail about future research directions that address actual policy impacts? There is a mention of assessing equity, but Reference 89 still takes measured changes in SSB intake and then models expected changes in QALYs, which is common practice but may require real world evaluation.

Reply: Thank you for this suggestion. We have expanded the respective paragraph in the discussion section to emphasize potential future research directions on effects estimates of actual policy impacts and their usefulness for simulation modeling applications (line 741ff in the marked-up copy).

Line 527-531: "Finally, as with all population health modelling studies, our results are subject to the validity of the underlying epidemiological evidence…" Although these appear to be reliable risk estimates, using them in these types of causal models (SSB tax would lead to) is potentially pushing them to their limit. How have other modelling studies performed when compared to real world evaluations of impacts of SSB taxes on BMI and health outcomes? Although there might be decent confidence in the potential health and therefore also economic benefits, it seems conceivable that a few grams per capita per day reduction does not translate to changes in outcomes of those magnitudes, unless real impacts are measured.

Reply: Thank you for this comment. To our knowledge, a comparison between simulation modeling studies and real-world evaluations of SSB taxes has so far not been attempted. This is partially because long-term benefits of SSB taxation will only materialize over the coming years. Additionally, the real-world evaluation of some relevant outcomes is likely not possible due to empirical identification problems. However, in the future, studies comparing intermediate outcomes (e.g., SSB consumption, BMI, type 2 diabetes incidence) between real-world evaluations and simulation modeling studies could be conducted. Such analyses could help adjust simulation modeling studies for unobserved mechanisms that affect policy impact (see also a recent publication on this topic from our team: <https://doi.org/10.1093/eurpub/ckac051>).

On the other hand, if we believe in the validity of currently available estimates of dietary risk factors such as added sugars, there is also reason to believe that a meaningful change in these risk factors will introduce a meaningful change in outcomes such as BMI and type 2 diabetes. However here, three factors need to be considered. First, we report population averages in the reduction of sugar consumption from beverages, but in the microsimulation the health benefits are of course larger for population subgroups that have a high baseline consumption since these groups also have a larger proportional reduction in consumption. Second, to be able to adequately simulate the many complex dimensions of policy impacts which ultimately determine the actual impact, we need good evidence and causal estimates on these dimensions. Thus, a difference in simulated and observed outcomes might also be related to relevant non-modelled/unobserved policy impact dimensions. In the case of SSB taxation these dimensions might for example be relevant changing dietary consumption patterns (including out-of-home consumption) or substitution to other goods (including changes in physical activity). Third, different methods to estimate long-term weight change on a population-level based on changes in energy intake (in our case induced by changes in added sugar intake) lead to vastly heterogenous results (as shown in the expanded footnote of Supplemental Table 7). To be able to better understand observed impacts (or non-impacts) of policies such as SSB taxes on relevant health outcomes like BMI; identify new approaches to non-communicable disease prevention; and perform reliable simulation of respective preventive policies, better evidence on the mechanisms, barriers, and facilitators of long-term weight reduction in humans and the aforementioned, complex policy impact dimensions is needed. This includes the relevance of threshold effects.

We have slightly expanded the discussion section to better reflect these issues (line 740ff in the marked-up copy).
