# Supplementary material for: Projected health and economic impacts of sugar-sweetened beverage taxation in Germany: A cross-validation modelling study
Source: PLoS Med. 2023 Nov 21;20(11):e1004311. doi: 10.1371/journal.pmed.1004311 (PMC10662751; doi:10.1371/journal.pmed.1004311)
Supplement: Attachment — Submitted filename: 09_27_2023_PLoS_Med_SSB_Tax_Germany_review_reply_2.docx [file pmed.1004311.s004.docx]

**Point-by-point reply to editorial and peer-reviewer comments for the manuscript entitled “Projected health and economic impacts of sugar-sweetened beverage taxation in Germany: a cross-validation modelling study” by Emmert-Fees et al.**

Thank you for providing us with the opportunity to revise our manuscript. Please find a point-by-point reply to all editorial and peer-review comments below.

GENERAL COMMENTS

1) We note that you switch between the descriptions “ad-valorem tax” and “20% ad-valorem tax”. Please note that you introduced this scenario as “ad-valorem tax” in line 252 and we suggest using a consistent format following this.

Reply: Thank you for pointing this out. We have now implemented a consistent wording for the scenarios throughout the manuscript. However, in some instances, we refer to ad-valorem taxation in general and not the specific scenario name as defined in the methods section of our manuscript. We have kept the original wording in these instances or changed to a more precise wording. Because scenarios are not already defined in the abstract and author summary, we have not changed the wording in these parts of the manuscript. Additionally, we ensured a consistent wording throughout the supplementary material.

2) Please note that it is necessary that you define the numbers presented in tables and figures indicating that in parentheses you are presenting, e.g. 95%-uncertainty intervals. Please thoroughly revise your manuscript including the Supplementary material regarding this comment.

Reply: Thanks for this comment. We have revised all relevant tables in the main manuscript and the supplementary material accordingly.

3) Please use a consistent format when using ‘Female’ and ‘Male’ as titles in figures and table. We suggest starting these words with a capitalized letter. Please revise thoroughly.

Reply: We have ensured a consistent format when describing results by sex in tables and figures in the manuscript and its supplement. We now use “Female” and “Male”.

4) Any mentions of age must have a unit (e.g., years). Please revise thoroughly.

Reply: We have ensured that all mentions of age in the main manuscript and its supplementary material have an appropriate unit.

5) Please revise your manuscript thoroughly regarding the introduction of abbreviations (at first use) and when used in figures and tables.

Reply: We thoroughly checked the manuscript regarding the use and introduction of abbreviations in the text, figures, and tables. We ensured that all abbreviations are defined at first use and have removed abbreviations that are only used once.

EDITORIAL COMMENTS

We suggest changing the title to “Predicted health and economic impacts of sugar-sweetened beverage taxation in Germany: a cross-validation modelling study” and re-wording “save billions of societal costs” in the abstract.

Reply: Thank you for these suggestions. We have discussed a change of the title according to your suggestion within the author team. However, we feel that including the word “predicted” is not appropriate for our study because we do not report the results from a prediction modeling study. We would prefer the inclusion of the word “projected”, which bears a similar meaning, and have revised the title accordingly. We are happy to follow the original suggestion, should the editors not agree with our proposal. Additionally, we have re-phrased to “save a substantial amount of societal costs”.

DATA AVAILABILITY

Thank you for the updated Data Availability Statement (DAS). Please note that a study author cannot be the contact person for the data so the statement “Additional code used for data cleaning and preparation is available upon request from the corresponding author.” is not acceptable in its current form.

Reply: We have removed the respective sentence from the DAS as we do not deem it necessary for the statement. The source code of the simulation model is available in an online repository and we think this is sufficient.

ABSTRACT

l.50: Please change to ‘aged 30-90 years’.

Reply: We have made the appropriate change.

l.59: Please define ‘BMI’.

Reply: We have made the appropriate change.

ll.65-70: Please revise this section. It reads confusingly – are there only numbers included for obesity and type 2 diabetes? Why not for cardiovascular disease? It might be clearer to insert the numbers following the disease.

Reply: We have revised this section of the abstract. To increase the readability of the abstract, we have removed the reporting of cases prevented/postponed and instead made the overall reporting of QALYs and costs saved more precise and consistent. Otherwise, because consistent reporting of cases prevented/postponed would be necessary for all three scenarios and three main disease (stroke, coronary heart disease and type 2 diabetes), the abstract would be considerably longer if included. We hope this change is sufficient. We have further made small edits to accommodate the word count of 500 words.

AUTHOR SUMMARY

Thank you for providing the Author Summary. The Author Summary is very long and needs to be shortened. It should contain only the most important details for each of the questions, in a condensed form. For example, in the first bullet point of “Why Was This Study Done?”, we suggest removing the examples in parentheses as this goes beyond the scope of the bullet points. Similarly, the first bullet point of “What Did the Researchers Do and Find?“ could be shortened to “We evaluated three SSB taxation scenarios in Germany with a simulation model: 1) 20% ad-valorem tax on SSBs; 2) extended 20% ad-valorem tax on SSBs and fruit juice; 3) tiered tax leading to reformulation of SSBs towards 30% lower sugar content.” or similar.

It may be helpful to review currently published articles for examples which can be found on our website here https://journals.plos.org/plosmedicine/

Reply: We have shortened the Author Summary as suggested and hope it is appropriate in its revised form.

INTRODUCTION

l.155: Please define ‘₤’.

Reply: We have made the appropriate change.

l.156: Please define ‘g’ and ‘ml’ (at first use).

Reply: We have made the appropriate change.

METHODS AND RESULTS

Please remove the numbering of the subheadings.

Reply: We have removed the numbering of subheadings but kept it for the original section headings as removal was not requested.

l.211: Please change to ‘age 30 to 90 years’. Please ensure to add a unit for age and revise throughout the entire manuscript.

Reply: We have made the appropriate change.

l.245: Please change ‘Thes’ to ‘The’.

Reply: We have made the appropriate change.

l.282: Please change to “we used data from the three KORA waves to incorporate time trends”.

Reply: We have made the appropriate change.

l.307: Please provide a reference.

Reply: We have provided an appropriate reference.

l.369: Please define ‘CVD’ (at first use).

Reply: We have made the appropriate change.

l.380: Please change to ‘age of 65 years’.

Reply: We have made the appropriate change.

l.435: Please define ‘PSA’.

Reply: This was an error. We have changed the abbreviation accordingly.

l.478: Please note that in most instances you have written ‘20% ad-valorem tax’ in quotation marks (e.g., see l.316 “20% ad-valorem tax”). Please use a consistent format and revise throughout the entire manuscript.

Reply: Thank you for pointing this out. We have now implemented a consistent wording for the scenarios throughout the manuscript. However, in some instances, we refer to ad-valorem taxation in general and not the specific scenario name as defined in the methods section of our manuscript. We have kept the original wording in these instances or changed to a more precise wording. Because scenarios are not already defined in the abstract and author summary, we have not changed the wording in these parts of the manuscript.

ll.486-488: You state that “[…] the largest impact on obesity, particularly among women due to their relatively high fruit juice consumption (Table 2).” and reference Table 2, whereas Table 2 only shows the influence on all individuals and not grouped by sex. Please add an appropriate reference.

Reply: We have moved the reference to Table 2 to the appropriate location in the sentence and added an additional reference for the mentioned statement.

l.498: Please change to ‘below 70 years’.

Reply: We have made the appropriate change.

ll.533-537: Why do you start presenting the costs in billions at this point, when you have consistently used millions before? Please keep the format consistent (we suggest millions).

Reply: We have changed the format consistently to millions in the main text. However, we still report billions in the abstract and the author summary.

DISCUSSION

l.557: Please re-phrase ‘save billions of from both healthcare […]’.

Reply: We have re-phrased the statement to “save a substantial amount of […]”.

TABLES

Table 1: The table requires revision. The table is missing a description of the changes in sugar consumption being presented by sex and different age groups. The age groups lack a unit. The titles ‘Absolute change from 2023 to 2043 in g/day’ and ‘Relative change from 2023 to 2043 in %’ should be positioned in the middle of the three columns containing the actual numbers (in the current form the titles read as a title for the sex and age groups listed below). It would also be helpful to add footnotes to the different policy scenarios guiding the reader to the table description.

Reply: We have revised the table according to the suggestions.

Table 1: The terms gender and sex are not interchangeable (as discussed in https://www.who.int/health-topics/gender); please use the appropriate term.

Reply: We have consistently used the term sex throughout our study referring to physiological sex as designated at birth.

Table 2: We suggest adding footnotes to the different policy scenarios guiding the reader to the table description. Please define ‘Diff.’ or use the full-length word.

Reply: We have revised the table according to the suggestions.

Table 2: We suggest changing the unit to ‘millions in €’ and removing the ‘€’ from the numbers presented in the table.

Reply: We have revised the table according to the suggestion. However we chose the wording “€-millions” so that the table is consistent with the wording in figures.

FIGURES

Please in the figure legend/description, define abbreviations used in each figure (including those in Supporting Information files).

Reply: We have made the appropriate changes.

Figure 1: Please define ‘ml’.

Reply: We have made the appropriate change.

Figure 2: Please define ‘BMI’.

Reply: We have made the appropriate change. Additionally, we have re-phrased the scenarios to be consistent with the manuscript text and the results tables.

Figure 3: Please change the unit on the y-axis to ‘in €-millions’ (as in Figure 2).

Reply: We have made the appropriate change. Additionally, we have re-phrased the scenarios to be consistent with the manuscript text and the results tables.

Figure 3: You stated that the plots are ‘stratified by sex and health economic perspective’ while there is no stratification by sex. Please revise.

Reply: We have corrected this error and removed mentioning of the stratification by sex.

Figure 4: You have divided the four graphs into A, B, C, D and not used this in the figure description. Please revise. We suggest changing the description to “Horizontal bar chart comparing cross-validation outcomes for cases prevented/postponed for A) coronary heart disease, B) stroke, C) type 2 diabetes and D) quality adjusted life years gained between the IMPACTNCD microsimulation (purple) and the PRIMEtime cohort model (green).” or similar.

Reply: We have revised the figure legend according to the suggestion.

REFERENCES

Where website addresses are cited, please use ‘accessed’ instead of ‘cited’ when specifying the date of access.

Reply: We have made the appropriate changes.

Please thoroughly revise all references including those in the S1 Appendix and ensure that journal name abbreviations match those found in the National Center for Biotechnology Information (NCBI) databases (http://www.ncbi.nlm.nih.gov/nlmcatalog/journals), and are appropriately formatted and capitalised (e.g., PloS medicine should be PloS Med, BMC public health should be BMC Public Health, Diabetic medicine : a journal of the British Diabetic Association should be Diabet Med).

Reply: We have made sure that all journal abbreviations match the NCBI databases. However, some of the cited journals are not in the NCBI databases and we have thus kept the original journal name. Additionally, our reference manager did not allow to mark these changes as tracked changes in Word. We hope this is fine.

SUPPLEMENTARY MATERIALS

Please revise the supplementary material thoroughly regarding the definition of abbreviations (including those in figures and tables).

Reply: We have thoroughly checked the use of abbreviations in the supplementary material.

Please ensure that all mentions of age (including axis labels) have a unit.

Reply: We have checked that all mentions of age have a unit and made the appropriate changes.

Please note that column titles should include the meaning of the numbers in parentheses, e.g. Sensitivity analysis 1: Ad-valorem tax with 10% tax rate (95%-uncertainty intervals).

Reply: We have made the appropriate changes.

Supplemental Methods 1, p.6, 2nd paragraph: Please define ‘US’.

Reply: We have made the appropriate change.

Supplemental Methods 1: p.15, 2nd paragraph: Please add a unit for age (‘aged 30 to 90 years’) and revise throughout the entire S1 Appendix.

Reply: We have made the appropriate changes.

Supplemental Figure 2: Please add an axis label for the axis of the age groups (top) and the years (right). Please define ‘kg’ and ‘m2’. We suggest changing the description to “[…] BMI distribution by 10-years age groups and year based […]” and would suggesting changing the title accordingly (like Supplemental Figure 5).

Reply: We have made the appropriate changes.

Supplemental Figure 3: Please add an axis label for the axis of the years (right). Please define ‘kg’ and ‘m2’.

Reply: We have made the appropriate change.

Supplemental Figure 4: Please explain the meaning of the light-colored lines in the figure description (dashed and solid). Please add a unit for age.

Reply: We have added the needed description and added the unit.

Supplemental Figure 4: Why is there no modelled line (solid line) for the NSV II data, but a observed line (dashed line) for the combined data??

Reply: We did not perform GAMLSS modeling on only the NVS II data because it was primarily used to supplement the KORA data. The observed line for the combined data is produced by plotting the observed values after combining the data. It is presented for comparison with the finally modelled and used combined data. The figure is correct and appropriate in its current form.

Supplemental Figure 5: Please add an axis label for the axis of the age groups.

Reply: We have made the appropriate change.

Supplemental Figure 7: Please add a unit for age. Please define ‘KORA’.

Reply: We have made the appropriate change.

Supplemental Figure 8: Please explain the meaning of the light-colored lines in the figure description (dashed and solid). Please add a unit for age.

Reply: We have added the needed description and added the unit.

Supplemental Figure 8: Why is there no modelled line for the NSV II data?

Reply: See reply to comment regarding Supplemental Figure 4 above. The figure is correct and appropriate in its current form.

Supplemental Figure 9: Please add an axis label for the axis of the age groups. Please change the x-axis label to ‘Fruit juice consumption in ml’.

Reply: We have made the appropriate change.

Supplemental Figure 10: Please change the x-axis label to ‘Fruit juice consumption in ml’.

Reply: We have made the appropriate change.

Supplemental Figure 11 onward: Why did you change the data presentation from “blue dashed lines red lines” to “(solid) blue lines and red lines”? We suggest using a consistent style.

Reply: We have made the appropriate change.

Supplemental Figure 11/12/13/14 We suggest changing the x-axis label to ‘Sugar consumption per ml of SSB’.

Reply: We have made the appropriate changes.

Supplemental Figure 15: Please change ‘x-axes’ to ‘x-axis’. In the title, please add a unit for age (‘for ages 30 and 50 years’).

Reply: We have made the appropriate change.

Supplemental Table 7: Please add a unit for age in the figure description (‘of age 35 years’). For the first outcome (BMI (baseline BMI <25)), please add a unit. We suggest changing the fifth column title to include the meaning of the numbers in parentheses – are these uncertainty intervals (e.g., Risk estimate by age group* (95%-uncertainty intervals))?

Reply: We have made the appropriate change.

Supplemental Table 8: For clarity, we suggest adding some of the information provided in Step 1 (p.33) to the table description. Please add a unit for the numbers presented in the table (years?).

Reply: We have made the appropriate change.

Supplemental Methods 1: p.37: Please change to “Where RR_(i1 … ik) are the relative risks that are related to the specific risk exposures of the synthetic individual, as in step 1.”

Reply: We have made the appropriate change.

Supplemental Methods 1: p.37: Please define ‘HDL’.

Reply: We have made the appropriate change.

Supplemental Methods 1: p.38: Please write ’17,594’ instead of ‘17594’ and ensure using a consistent number format throughout the entire Appendix (e.g., also see p.56).

Reply: We have checked the number formatting and made the appropriate changes throughout the entire Appendix.

Supplemental Figure 17: Please consider avoiding the use of red and green in order to make your figure more accessible to those with colour blindness. We suggest adding the years of the follow-up studies to be included in the description.

Reply: We have added the years of the follow-up to the description and changed the color palette of the figure to the Viridis palette which is appropriate for color blindness.

Supplemental Table 9: Please add a unit for age in the figure description (‘of age 35 years’). When describing BMI, please add a unit.

Reply: We have added a unit to BMI where appropriate. However, we are not sure how to address the first part of the comment as there is no mentioning of “of age 35…”.

Supplemental Methods 1: p.43: Please change ‘Hessia’ to ‘Hesse’, ‘Saxonia-Anhalt’ to ‘Saxony-Anhalt’ and ‘Mecklenburg-Vorpommern’ to ‘Mecklenburg-Western Pomerania’.

Reply: We have made the appropriate changes.

Supplemental Methods 1: p.45: Please define ‘QALYs’ at first use.

Reply: We have made the appropriate change.

Supplemental Table 12: In ‘Comments & assumptions’, please either exchange ‘Kähm et al. (2018)’ with the according reference ([33]) or add the according reference to it in the description.

Reply: We have removed the reference completely as it is already present in the “Source” column.

Supplemental Table 13: Please explain in a footnote what the ‘EQ-5D-5L’ is.

Reply: We have made the appropriate change.

Supplemental Methods 1: p.57: Please change ‘60.000’ to ’60,000’ and see the comment about using a consistent number format.

Reply: We have made the appropriate change.

Supplemental Table 17/18: Please see the comment about using a consistent number format.

Reply: We have made the appropriate changes.

Supplemental Methods 1: p.61: Please change to ‘at age 60 years’.

Reply: We have made the appropriate change.

Supplemental Figure 19: Please note that the graphs are difficult to compare due to the varying y-axis. Please ensure that the y-axis is identical for all figures to facilitate comparison. The observed mortality rates start in 2013 whereas the y-axis and black dots (presenting the observed mortality rates) start at year 1990 – please explain. From the figure description, the discrepancy between time points will not be clear to the reader.

Reply: We have adjusted the figure legend to be more precise. We did not adjust the y-axes because this would make interpretation of the figure impossible. The aim of the figure is not to compare mortality rates between age groups but to compare observed, forecasted, and simulated mortality rates for each age group. This comparison enables an assessment of calibration and internal validity of the model. Fixing the y-axis would make this comparison impossible due to the large differences in mortality rate by age group. This and similar figures are correct in their current form.

Supplemental Figure 20: See comment for Supp. Figure 19. Also, please change to “[…] coronary heart disease (CHD) mortality rates in Germany in women”.

Reply: We have adjusted the figure title. Regarding the y-axes see reply to comment for Supplemental Figure 19 above.

Supplemental Figure 21/22/23/24: See comment for Supp. Figure 19.

Reply: See reply to comment for Supplemental Figure 19 above.

Supplemental Figure 25-29: We suggest changing the x-axis label to ‘Age group (years)’. Please explain the meaning of the point clouds and the violin plot.

Reply: We have adjusted the x-axis labels. We have slightly adjusted the explanation in the figure legends but otherwise think the existing explanation and description is sufficient. All relevant information is present for interested readers.

Supplemental Figure 25-27: Please ensure that the y-axis is identical for all figures to facilitate comparison.

Reply: Similar to the reply to the comment for Supplementary Figure 19, we did not adjust the y-axis because the aim of the plots is to enable an assessment of internal validity with regards to the input prevalence and incidence of the modelled diseases compared to the output of these measures from the model. These figures are correct in their current form.

Supplemental Figure 28-29: Please ensure that the y-axis is identical for all figures to facilitate comparison.

Reply: Similar to the reply to the comment for Supplementary Figure 19, we did not adjust the y-axis because the aim of the plots is to enable an assessment of internal validity with regards to the input prevalence and incidence of the modelled diseases compared to the output of these measures from the model. These figures are correct in their current form.

Supplemental Figure 31/32/33/34/35/36/37/38/39: Please mention in the figure description that data is presented by sex and age group.

Reply: We have made the appropriate changes.

Supplemental Figure 33: Please correct the x-axis label for the two top graphs to ‘Year’. Why does the x-axis start before 2010? Please mention in the figure description that data is presented by sex and age group. Please note that the shaded areas are hardly recognizable.

Reply: We have made the appropriate changes.

Supplemental Figure 36: Please define ‘GEDA’.

Reply: We have made the appropriate change.

Supplemental Methods 3: p.85: We suggest defining ‘SSB’ anew as this is a new segment of the S1 Appendix.

Reply: We have made the appropriate change.

Supplemental Methods 3: p.86: Please define ‘BMI’.

Reply: We have made the appropriate change.

Supplemental Figure 40/41: Please define the meaning of the three SSB taxation scenarios in the figure description. You have divided the four graphs into A, B, C, D and not used this in the figure description. Please revise (see comment Figure 4). Please define ‘CE’ in PRIMEtime CE.

Reply: We have made the appropriate changes.

Supplemental Figure 41: Where is the horizontal bar for CHD and stroke for females in the ad-valorem tax group?

Reply: We have checked this before the original first submission. The mean is zero and the figure therefore correct.

Supplemental Figure 42/43: Please ensure that the y-axis is identical for figures of the same group to facilitate comparison. Please define ‘SSB’ (add in the description following ‘sugar-sweetened beverage’).

Reply: We have made the appropriate changes and made the scenario descriptions consistent.

Supplemental Table 19: Please change the column title to include the meaning of the numbers in parentheses – are these uncertainty intervals (e.g., Difference to baseline in 2043 (95%-uncertainty intervals))?

Reply: We have made the appropriate change.

Supplemental Table 20/21: Please change the column titles to include the meaning of the numbers in parentheses – are these uncertainty intervals (e.g., Sensitivity analysis 1: Ad-valorem tax with 10% tax rate (95%-uncertainty intervals))? Please add a unit for age.

Reply: We have made the appropriate changes.

Supplemental Table 22: Please change the column titles to include the meaning of the numbers in parentheses – are these uncertainty intervals (see comment Supplemental Table 20/21)? Also, consider this for the QALYs data.

Reply: We have made the appropriate changes.

Supplemental Table 23/24/25/26: Please change the column titles to include the meaning of the numbers in parentheses – are these uncertainty intervals (see comment Supplemental Table 20/21)?

Reply: We have made the appropriate changes.

Comments from Reviewers:

Reviewer #1: I think the authors have adequately addressed all the comments and concerns from me and other reviewers. I would recommend for acceptance. Congratulations to the authors.

Reply: Thank you for your kind words and recommendation for acceptance.

Reviewer #2: The authors have addressed all my previous comments satisfactorily. I enjoyed reviewing this paper, and congrats to the authors for their study.

Reply: Thank you for your constructive feedback.

Reviewer #3: The comments and revisions are satisfactory.

Reply: Thank you for your comments.
