# Supplementary material for: Projected health and economic impacts of sugar-sweetened beverage taxation in Germany: A cross-validation modelling study
Source: PLoS Med. 2023 Nov 21;20(11):e1004311. doi: 10.1371/journal.pmed.1004311 (PMC10662751; doi:10.1371/journal.pmed.1004311)
Supplement: Attachment — Submitted filename: Author summary_PMEDICINE-D-23-01506R3.docx [file pmed.1004311.s005.docx]

Why Was This Study Done?

- Taxation of sugar-sweetened beverages (SSBs), recommended by the World Health Organization and implemented in many jurisdictions globally, aims to reduce the non-communicable disease burden by disincentivizing consumption through increased consumer prices or incentivizing industry reformulation to reduce SSB sugar content.
- No tax on SSBs is currently enacted in Germany and the national government is preparing a new national strategy on food seeking evidence-based recommendations to establish policy priorities until 2050.
- In Germany, the potential long-term health and economic impacts of SSB taxation has not been evaluated.

What Did the Researchers Do and Find?

- We developed and validated a microsimulation model based on national data and international evidence to model the impact of SSB taxation on dietary exposure of added sugar from beverages, body mass index (BMI), cardiometabolic diseases and related economic costs.
- We evaluated three SSB taxation scenarios in Germany with a simulation model: 1) 20% ad-valorem tax on SSBs; 2) extended 20% ad-valorem tax on SSBs and fruit juice; 3) tiered tax leading to reformulation of SSBs towards 30% lower sugar content.
- Taxation of SSBs in Germany could prevent or postpone 132,100 to 244,100 cases of type 2 diabetes, gain 106,000 to 192,300 quality-adjusted life years and save €10.8 to €16.0 billion in societal cost from 2023 to 2043 with the highest impacts estimated for tiered taxation.
- The absolute long-term health impacts are largely dependent on the relevance of direct, BMI-independent cardiometabolic effects of SSBs.

What Do These Findings Mean?

- All modelled SSB taxation scenarios are likely to improve population health and reduce societal costs in Germany by preventing cardiometabolic disease.
- Considering all sources of uncertainty, modelled SSB taxation scenarios leading to reformulation towards less sugar might have a larger population-level health and economic impact than those that incentivize consumer behaviour change only through increased prices.
- From a public health perspective, taxation of SSBs should be considered as a policy option for German decision makers to reduce consumption of added sugar and improve population health.
